# *Naja annulifera* Snake: New insights into the venom components and pathogenesis of envenomation

**DOI:** 10.1371/journal.pntd.0007017

**Published:** 2019-01-18

**Authors:** Felipe Silva-de-França, Isadora Maria Villas-Boas, Solange Maria de Toledo Serrano, Bruno Cogliati, Sonia Aparecida de Andrade Chudzinski, Priscila Hess Lopes, Eduardo Shigueo Kitano, Cinthya Kimori Okamoto, Denise V. Tambourgi

**Affiliations:** 1 Immunochemistry Laboratory, Butantan Institute, São Paulo, Brazil; 2 Special Laboratory of Applied Toxinology, Butantan Institute, São Paulo, Brazil; 3 Department of Pathology, School of Veterinary Medicine and Animal Science, University of São Paulo, São Paulo, Brazil; Liverpool School of Tropical Medicine, UNITED KINGDOM

## Abstract

**Background:**

*Naja annulifera* is a medically important venomous snake occurring in some of the countries in Sub-Saharan Africa. Accidental bites result in severe coagulation disturbances, systemic inflammation and heart damage, as reported in dogs, and death, by respiratory arrest, in humans. Despite the medical importance of *N*. *annulifera*, little is known about its venom composition and the pathogenesis of envenomation. In this paper, the toxic, inflammatory and immunogenic properties of *N*. *annulifera* venom were analyzed.

**Methodology/Principal findings:**

Venom proteomic analysis identified 79 different proteins, including Three Finger Toxins, Cysteine Rich Secretory Proteins, Metalloproteinases, Phospholipases A_2_ (PLA_2_), Hyaluronidase, L-amino-acid oxidase, Cobra Venom Factor and Serine Proteinase. The presence of PLA_2_, hyaluronidase, fibrinogenolytic and anticoagulant activities was detected using functional assays. The venom was cytotoxic to human keratinocytes. In an experimental murine model of envenomation, it was found that the venom induced local changes, such as swelling, which was controlled by anti-inflammatory drugs. Moreover, the venom caused death, which was preceded by systemic inflammation and pulmonary hemorrhage. The venom was shown to be immunogenic, inducing a strong humoral immune response, with the production of antibodies able to recognize venom components with high molecular weight and to neutralize its lethal activity.

**Conclusions/Significance:**

The results obtained in this study demonstrate that *N*. *annulifera* venom contains toxins able to induce local and systemic inflammation, which can contribute to lung damage and death. Moreover, the venom is immunogenic, an important feature that must be considered during the production of a therapeutic anti-*N*. *annulifera* antivenom.

## Author summary

*N*. *annulifera* is a dangerous snake that belongs to the Elapidae family. It is found in some of the countries in Sub-Saharan Africa and has caused accidents in humans and dogs. In this study, we characterized some of the biochemical, toxic and immunogenic properties of *N*. *annulifera* venom. We showed that the venom is composed of several proteins, some of which display enzymatic activities, such as phospholipase A2, hyaluronidase, metalloproteinases and serine proteinases. The venom promoted disturbances in the human coagulation system and was cytotoxic to human epidermal cells. Using a mouse model, we showed that the venom promotes local reactions that were reduced with anti-inflammatory drugs. The venom caused systemic inflammation, lung hemorrhage and death. Further, the venom stimulated production of high antibody titers when injected into mice and the antiserum produced was able to inhibit venom-induced death. This study demonstrated that *N*. *annulifera* venom contains toxins that trigger inflammatory process, which may contribute to the envenomation pathology. Moreover, the venom is immunogenic, an important aspect for the production of an efficient *N*. *annulifera* antivenom.

## Introduction

Envenoming from snakebites is a public health problem in rural areas of the tropical and subtropical countries in Africa, Latin America, Asia and Oceania [1, 2]. This medical condition kills more than 95,000 people per year and leads over 300,000 victims to live with permanent sequelae [3].

It is also estimated that there are approximately 3,700 species of snakes worldwide [4]. Of these, approximately 15% are venomous and have caused serious accidents involving humans and other animals [5]. Venomous snakes belong to the Colubroidea superfamily, which is composed of several families, such as Colubridae, Viperidae, Lamprophiidae and Elapidae [6, 7]. The Elapidae family consists of 61 genera and includes 365 species [8], which are distributed in the tropical and temperate regions of Africa, America, Asia and Australia. These snakes can live in terrestrial or aquatic environments and present variable diet, including small vertebrates, such as birds, rodents, reptiles and fishes or invertebrates [9, 10].

The venom of Elapidae is well known to contain powerful neurotoxins that play a role in the snake defense against predators and prey capture. These neurotoxins may also be responsible for some of the clinical manifestations observed in human envenomation, such as respiratory arrest [11, 12]. However, several studies have noted the presence of different components in these venoms, including phospholipases A_2_ (PLA_2_), hyaluronidases (HYA) [13, 14, 15], metallo- (SVMP) and serine proteinases (SVSP) [16, 17, 18], inhibitors [19], peptides [20] and cytotoxins [21, 22]. All of these components may have cytotoxic [14, 23, 24], hemorrhagic, anticoagulant [25], pro-inflammatory [26, 27] or immunogenic [28] properties.

The genus Naja presents specimens that cause a large and serious number of accidents [3, 8, 23, 29]. One dangerous representative of this genus is *Naja annulifera*, which is popularly called the Banded or Snouted Cobra [30, 31]. It is found in Sub-Saharan Africa countries, including Zambia, Malawi, Mozambique, Swaziland, Zimbabwe, Botswana and South Africa, in savannah grasslands, deserts, and rocky areas and near human habitations. This ranging of habits is associated to its broad diet, once it feeds on frogs, lizards, birds and it eggs, snakes and rats.

Accidents involving *N*. *annulifera* are considered severe. The envenomed individuals experience swelling, pain and local burning at the site of the bite, followed by pain throughout their entire body. Beside these clinical findings, affected individuals can present with dizziness and palpebral ptosis. Some can progress to respiratory arrest and, without a specific treatment, death. The treatment for envenomated individuals is serum therapy and in respiratory arrest cases, mechanical ventilation. Some studies have also reported that envenomed individuals in South Africa may develop necrosis at the site of the bite as well as hematologic disturbances [23, 31, 32, 33].

Veterinary epidemiologic data demonstrated that approximately 60% of dogs poisoned by snakebites in South Africa were bitten by *N*. *annulifera*. These dogs presented with various clinical findings, including hematologic alterations, such as leukocytosis and thrombocytopenia [34], increased plasma levels of Cardiac Troponin I and C Reactive Protein (CRP) [35] and disturbances in the coagulation system [36].

Information about the components of *N*. *annulifera* venom is scarce. Some authors have noted the presence of several cyto/cardiotoxins and neurotoxins [37, 38, 39, 40, 41, 42]. Despite its medical importance, epidemiologic, clinical and experimental studies of *N*. *annulifera* venom are limited and the mechanisms by which it causes toxicity remains poorly understood. The goal of the present study was therefore to describe the components of *N*. *annulifera* venom and its toxic activities, utilizing *in vitro* and *in vivo* models to better understand the pathology of envenomation by this snake.

## Methods

### Reagents

Bovine serum albumin (BSA), Concanavalin A from *Canavalia ensiforms* (ConA), Wheat Germ Agglutinin from *Triticutun vulgaris* (WGA), Tween 20, Triton X-100, 3’3’-Diaminobenzidine (DAB), Hyaluronic Acid, anti-mouse IgG horseradish labeled with Peroxidase (IgG-HRPO), Ortho-phenylenediamine (OPD), cetyltrimethylammonium bromide (CTAB), Gelatin, Comassie Brilliant Blue R-250, Human Fibrinogen (Fb), Human Thrombin, 1,10 Phenantroline (1,10 Phe), Phenylmethasulfonyl Fluoride (PMSF), Dimethyl Sulfoxide (DMSO), Cromolyn, Dexamethasone, Trypsin, Solid Phase Extraction Disks (SDB-XC membranes), Hepes, urea, iodoacetamide and trifluoroacetic acid (TFA) were purchased from Sigma-Aldrich (Missouri, USA). The Bicinchoninic Acid (BCA) Protein Assay Kit was purchased from Pierce Biotechnology, Inc. (Wisconsin, USA). SDS-PAGE (Amersham ECL Gel 8–16%, 10 wells) gels were obtained from GE Healthcare Life Sciences (Uppsala, Sweden). Dithiothreitol (DTT) was from Calbiochem (Darmstadt, Germany). The nitrocellulose membrane and EnzChek Phospholipase A2 Assay Kit were purchased from Thermo Fisher Scientific (Massachusetts, USA). Aluminum Hydroxide (Al(OH)_3_) was purchased from Prati Donaduzzi (Sao Paulo, Brazil). Anti-mouse IgG labeled with alkaline phosphatase (IgG-AP), Nitroblue Tetrazolium chloride (NBT), 5-bromo-4-chloro-3-indolyl-phosphatase (BCIP) and CytoTox 96 Non-Radioactive Cytotoxicity Assay Kit were obtained from Promega Corp. (Madison, Wisconsin, USA). ELISA plates were purchased from Costar Corning, Inc. (New York, USA). Calcium chloride, Cephalin and Thromboplastin were obtained from Stago (Saint-Quen-I’Aumône, France). Indomethacin, MK-886 (Sodium Salt) and WEB-2086 were purchased from Cayman Chemical (Michigan, USA). The BD Cytometric Bead Array (CBA) Mouse Inflammation and Mouse IL-1β ELISA kits were obtained from BD Biosciences (New Jersey, USA). The mouse IL-17 DuoSet3.2.4.3 ELISA kit was obtained from R&D Systems (Minnesota, USA). The Panótico Rápido kit was purchased from Laborclin (Parana, Brazil). The HaCat human keratinocyte lineage was obtained from the Rio de Janeiro Cell Bank (Rio de Janeiro, Brazil). Dulbecco's Modified Medium Eagle—Gibco (DMEM) medium, penicillin and streptomycin were purchased from Invitrogen Corp. (California, USA). Fetal Bovine Serum was obtained from Cultilab (São Paulo, Brazil). 3-(4,5-dimethylthiazol-2-yl)2,5-di-phenyltetrazolium bromide (MTT) was obtained from Merk (Darmstadt, Germany). Frits for SPE cartridges were obtained from Agilent (California, USA) 10 μm Jupiter C-18 beads were purchased from Phenomenex (Torrance, USA). 3 μm ReproSil-Pur C-18 beads were obtained from Dr. Maisch (Ammerbuch, Germany). 75 μm I.D. or 100 μm I.D. x 360 μm O.D. polyimide coated capillary tubing were purchased from Molex (Lisle, USA).

### Venoms

*N*. *annulifera* venom (South Africa specimens) was purchased from Latoxan Natural Active Ingredients (Valence, France). The lyophilized venom was reconstituted in sterile saline solution at 5 mg/mL. The protein content was assessed with the BCA Protein Assay Kit according to the manufacturer’s recommendations. The samples were aliquoted and stored at -80°C until use. *Bothrops jararaca*, *Crotalus durissus terrificus* and *Tityus serrulatus* venoms were supplied by Butantan Institute, SP, Brazil, and their use was approved by the Brazilian Institute of Environment and Renewable Resources (IBAMA), an enforcement agency of the Brazilian Ministry of the Environment (protocol number: 010035/2015-0), and by SisGen (Sistema Nacional do Patrimônio Genético e do Conhecimento Tradicional Associado (protocol numbers AD50761 and AEE9AEA).

### Ethics statement

High_III_ (H_III_) female mice weighing 18–22 g were obtained from the Immunogenetics laboratory, while Balb/c male mice weighing 18–22 g were obtained from the Center for Animal Breeding, both from Butantan Institute. All procedures involving animals were in accordance with the ethical principles for animal research adopted by the Brazilian Society of Animal Science and the National Brazilian Legislation n°.11.794/08. The protocols used in the present study were approved by the Institutional Animal Care and Use Committee of the Butantan Institute (protocols approved n° 01092/13 and 01262/14).

Experiments using samples obtained from humans were previously approved by the Human Research Ethics Committee of the Municipal Health Secretary of São Paulo. Human blood samples were obtained from healthy donors who knew of the purposes of this study and signed the corresponding informed consent form (protocol approved n° 974.312).

### Characterization of the components of *N*. *annulifera* venom

#### Electrophoresis and lectin Western blot

Samples of *N*. *annulifera* venom (15 μg) were separated by SDS-PAGE on 8–16% or 12% gels [43], under reducing or non-reducing conditions, and were silver stained [44] or blotted on nitrocellulose membranes [45]. After the transfer, the membranes were blocked with 5% BSA in Phosphate Buffered Saline (PBS) (8.1 mM sodium phosphate, 1.5 mM potassium phosphate, 137 mM sodium chloride and 2.7 potassium chloride, p.H. 7.2) and then incubated with peroxidase labeled lectins, ConA (1:1000 dilution) or WGA (1:2000 dilution) to detect residues of Mannose and N-acetylglucosamine [46]. Glycosylated proteins were detected using a solution that contained 0.1% hydrogen peroxide plus 0.5 mg/mL DAB.

#### Mass spectrometric protein identification

For the proteomic analysis, *N*. *annulifera* venom was submitted to trypsin digestion as described by Kinter and Sherman [47]. Briefly, 200 μg venom (three replicates) were denatured with 6 M urea in 100 mM Tris-HCl, pH 8, and reduced with dithiothreitol (DTT) 10 mM for 1 h at room temperature. Cysteine carbamidomethylation was performed by incubation with iodoacetamide 40 mM for 1 h at room temperature, followed by addition of DTT 40 mM to consume excess of alkylating agent. Protein samples were diluted with water until the concentration of urea decreased to about 0.7 M and trypsin was added using an enzyme: substrate ratio of 1:50 (w/w) at 37°C for 18 h. The reaction was stopped with 20% TFA until pH 3 and desalting was carried out by loading individual samples to p-1000 StageTip SDB-XC [48]. After drying using a vacuum centrifuge, peptide mixtures were dissolved in 100 μL of 0.1% formic acid (solution A) and peptide concentration was estimated. For that, a peptide standard curve was prepared using a mixture of trypsin-digested bovine serum albumin (MassPREP BSA Digestion Standard; Waters) ranging from 0.2 to 1.2 μg/μL (in solution A). The absorbance was measured at 214 nm using a NanoDrop spectrophotometer (Thermo Scientific), and peptide sample concentration was determined using the standard curve. For analysis, each peptide sample (5 μg) was automatically injected using an EASY Nano LCII system (Thermo Scientific) into a 5 cm of 10 μm Jupiter C-18 trap column (100 μm I.D. × 360 μm O.D.) coupled to an LTQ-Orbitrap Velos mass spectrometer (Thermo Scientific). Chromatographic separation of tryptic peptides was performed on a 15-cm long column (75 μm I.D. x 360 μm O.D.) packed in-house with 3 μm ReproSil-Pur C-18 beads (Dr. Maisch, Germany). Peptides were eluted with a linear gradient of acetonitrile in 0.1% formic acid (solution B) at 200 nL/min: the linear step used for the elution was 5–35% in 75 min. Spray voltage was set at 2.2 kV and the mass spectrometer was operated in data dependent mode, in which one full MS scan was acquired in the m/z range of 300–1,600 followed by MS/MS acquisition using collision induced dissociation of the ten most intense ions from the MS scan. MS spectra were acquired in the Orbitrap analyzer at 30,000 resolution (at 400 m/z) whereas the MS/MS scans were acquired in the linear ion trap. The minimum signal threshold to trigger fragmentation event, isolation window, activation time and normalized collision energy were set to, respectively, 1,000 cps, 2 m/z, 10 ms and 35%. A dynamic peak exclusion (list size of 500) was applied to avoid the same m/z of being selected for the next 20 seconds. Two independent LC-MS/MS runs were performed for each sample.

#### Database search

Protein identification was performed using the MaxQuant software suite (v 1.5.3.12) [49] using the theoretical trypsin fragments produced from the database restricted to the taxonomy “Serpentes” (Uniprot release September 14, 2016; 63,114 sequences). The database search considered the precursor mass tolerance of 20 ppm, in the first search, and the nonlinear mass recalibration was made in the masses of first pass identifications. The second search considered the mass tolerance of 4.5 ppm and tolerance for fragments was set to 0.5 Da. The following search parameters were used: iodoacetamide derivatives of cysteine were considered as fixed modification, oxidation of methionine was specified as variable modification, and up to two missed cleavages by trypsin were accepted. Assignments for peptides and proteins were accepted at a false discovery rate < 1%. For the identification, only proteins identified in at least two experimental replicates, with at least 1 unique peptide and posterior error probability ≤ 0.01 were accepted. “Match between runs” feature was enabled. For each protein group in the MaxQuant’s ‘proteinGroups.txt’ file, the first protein entry was selected as representative. The mass spectrometry proteomics data have been deposited to the ProteomeXchange Consortium (http://proteomecentral.proteomexchange.org), via the PRIDE partner repository [50], with the dataset identifier: PXD010962 (Username: reviewer27828@ebi.ac.uk; Password: BNxFCLRG).

### Toxic-Enzymatic properties of the venom

#### Gelatinolytic activity

The gelatinolytic activity of the *N*. *annulifera* snake venom was evaluated with zymography using gelatin as the substrate [51]. Samples of the venom (30 μg), prepared under non-reducing conditions, were separated via 10% SDS-PAGE with 1 mg/ml gelatin. The gels were washed for 2 hours at room temperature in 2.5% Triton X-100 solution and incubated overnight at 37°C in substrate buffer (50 mM Tris-HCl, 200 mM NaCl, 10 mM CaCl_2_, 0.05% Brij-35, p.H. 8.3). After this period, the gels were stained with Coomassie Brilliant Blue solution (40% methanol, 10% acetic acid and 0.1% Coomassie Brilliant Blue).

#### Fibrinogen cleavage

Human fibrinogen samples (30 μg) were incubated with 5 μg of *N*. *annulifera* venom or saline for 1 hour at 37°C under constant agitation. In parallel, venom samples were pre-incubated with 1.10 Phe (10 mM) or PMSF (10 mM) at room temperature for 30 minutes. After incubation, the mixtures were submitted to SDS-PAGE using a 8–16% gradient gel under reducing conditions and stained with Coomassie Brilliant Blue solution. The positive control of the reaction was human Thrombin (1U).

#### Hyaluronidase activity

Hyaluronidase activity was analyzed according to the methodology described by Purkrittayakamee et al. [52] with slight modifications. Briefly, samples of *N*. *annulifera* venom (20 μg) were incubated with a mixture that contained hyaluronic acid (1 mg/mL) (100 μL) and acetate buffer (200 mM sodium acetate and 0.15 M of NaCl, p.H. 6.0) (400 μL) at 37°C for 15 minutes. After incubation, the reactions were stopped with 1 mL of CTAB (2.5% CTAB and 2% NaOH). The absorbances were measured at λ405 nm in a spectrophotometer (Multiskan EX, Labsystems, Finland) against a blank that contained hyaluronic acid, acetate buffer and CTAB. The results are expressed as units of turbidity reduction (UTR) *per* mg of venom. *T*. *serrulatus* scorpion venom (20 μg) was used as a positive control.

#### PLA_2_ activity

PLA_2_ activity was evaluated using the EnzChek^TM^ Phospholipase A2 Assay Kit according to the manufacturer’s recommendations. Briefly, samples of *N*. *annulifera* venom (0.5 μg) was incubated with a phospholipid mix that contained 10 mM Dioleoylphosphatidylcholine and 10 mM Dioleoylphosphatidylglycerol in 96-well microtiter plates. Increased fluorescence was evaluated using a spectrometer FLUOstar Omega (BMG Labtech, Ortenberg, Germany) at λ_EM_ 460 and λ_EX_ 515 nm and 37°C for 10 minutes. Specific activity was expressed as Units of Fluorescence (UF) *per* minute *per* microgram of venom. *C*. *d*. *terrificus* (0.5 μg) venom was used as positive control.

#### Cytotoxic activity

The HaCat human keratinocyte cell lineage was cultured in DMEM supplemented with 10% fetal bovine serum and 1% penicillin-streptomycin at 37°C and 5% CO_2_. The action of *N*. *annulifera* venom on the viability of human keratinocytes was evaluated using the MTT (3- (4,5-dimethylthiazol-2-yl) -2,5-diphenyltetrazolium bromide) method [53] with minor modifications. Keratinocytes (5×10^4^ cells/well) were grown in 96-well plates that contained supplemented DMEM medium and were then maintained for 24 hours in DMEM medium without SFB. After this period, cells were incubated for 72 hours in increasing venom concentrations at 37°C and 5% CO_2_. The supernatant from the wells was aspirated and 60 μl of the MTT solution (0.83 μg/μL) in incomplete DMEM medium was added to the wells. The plates were then incubated for 30 minutes at 37° C and 5% CO_2_. Thereafter, the supernatant was aspirated, 100 μL/well of DMSO was added, and the plates were allowed to stand at room temperature for five minutes. The spectrophotometric analysis of the reactions (Multiskan-EX, Labsystems, Helsinki, Finland) was performed at λ 540 nm. Cell viability was calculated as: [O.D. experimental sample _(540nm)_*100]/[O.D. control sample_(540nm)_]. The results are expressed as cell viability (%).

Additionally, the action of *N*. *annulifera* venom on the viability of human keratinocytes was evaluated by Lactate Dehydrogenase (LDH) release assay. Supernatants of keratinocyte cultures, treated for 72 hours with increasing venom concentrations, were centrifuged at 405× *g* for 10 min, and tested for the presence of LDH, using the CytoTox 96 Non-Radioactive Cytotoxicity Assay Kit, according to the manufacturer's instructions.

### The impact of venom on hemostatic parameters

Blood samples from healthy donors were placed into tubes containing sodium citrate (3.2%) and centrifuged at 260× *g* at room temperature to obtain platelet-poor plasma (PPP). The PPP samples were aliquoted and stored at -20°C until their use.

#### Activated Partial Thromboplastin Time (APTT)

PPP samples (50 μL) were treated with saline or increasing concentrations of venom and incubated for 3 minutes at 37°C with 50 μL of Cephalin. PPP was then recalcified with CaCl_2_ (0.025 M), and the coagulation time was measured over 240 seconds in a Stago Start System (Asnières-sur-Seine, France). The results are expressed in seconds and *R-time*, which was obtained from the experimental sample/control sample. Disorders in coagulation time were considered significant when the *R-time* was over 1.3 [54].

#### Prothrombin Time (PT)

PPP samples (50 μL) were treated with saline or increasing concentrations of venom and incubated for 1 minute at 37°C. PPP was then incubated with CaCl_2_ (0.025 M) that contained thromboplastin (100 μL), and the coagulation time was followed for 60 seconds using the Stago Start System (Asnières-sur-Seine, France). The results are expressed in seconds and *R-time*, which was obtained from the experimental sample/control sample. Abnormalities in coagulation time were considered significant when the *R-time* was over 1.3 [54].

### *In vivo* assays

#### Lethal Activity of the venom (LD_50_)

Balb/c mice groups (n = 6) were intraperitoneally (i.p) inoculated with increasing concentrations of venom (20, 40, 60, 80, 100 and 120 μg) [55] or sterile saline. After inoculation, animals were monitored for 72 hours and LD_50_ was calculated using the Probit transformation [56]. The results are expressed as μg of venom *per* mouse.

#### Local reactions: Edematogenic activity and pharmacological modulation

The venom edematogenic activity was assessed via the method previously published by Yamakawa et al. [57] with some modifications. Balb/c mice (n = 6) were inoculated with 10 μg of *N*. *annulifera* venom at a volume of 50 μL (sterile saline) in the subcutaneous (s.c) tissue of the plantar region of the animal’s left hindpaw. The contralateral hindpaw (control) was inoculated with 50 μL of sterile saline. To assess edematogenic activity, the thickness of the hindpaw was evaluated with a caliper rule (Mitutoyo, Suzano-SP, Brazil; sensibility of 0.01 mm) at different time points before (T0) and over the 24 h, following either venom or saline inoculation (Te). Increases in paw volume are expressed in percentage form (%), calculated with the following formula: (Te-T0)/T0*100.

To evaluate the contribution of mast cells and different lipid mediators to the hindpaw edema induced by *N*. *annulifera* venom, mice (n = 6 *per* drug) were pretreated with different anti-inflammatory compounds: a) Sodium Cromoglycate, a mast cell degranulation inhibitor (10 mg/kg), administered i.p. for 3 days before edema induction [58]; b) dexamethasone, an indirect cPLA_2_ inhibitor, was given to mice (2 mg/kg) i.p. 2 h before venom inoculation [59]; c) the non-selective cyclooxygenase inhibitor Indomethacin, was administered (10 mg/kg) i.p. 30 minutes [60] before venom; d) MK-886, a 5-lipoxigenase-activating protein inhibitor, was administered (5 mg/kg) i.p. 30 minutes [61] before edema induction; and e) WEB-2086, a Platelet Activating Factor Receptor antagonist, was administered (5 mg/kg) s.c. 1 hour before venom administration [62, 63]. Dexamethasone and Cromolyn were diluted in saline, while Indomethacin, MK-886, WEB-2086 were diluted in DMSO. Following these pretreatments, edema was induced and monitored for 24 h.

#### Systemic reactions

To analyze the possible systemic reactions induced by *N*. *annulifera* venom, two experimental groups were established. The first measured systemic alterations over time. LD_50_ assays were performed to define the sublethal dosing level that was able to induce the deleterious effects of the venom, including an increase in inflammatory parameters, but not kill the animals. LD_50_ values that varied between 50 and 75% were tested, and the selected dose for the inflammatory assays was 60% of LD_50_. Balb/c mice (n = 6) were then inoculated with 60% of LD_50_ or sterile saline i.p. and euthanized at different time points to obtain blood and collect the organs.

In another experimental set, it was assessed whether death promoted by venom was preceded by systemic reactions. Animal groups (n = 6) were inoculated with saline or 2 LD_50_ of *N*. *annulifera* venom i.p. Immediately after animals’ death, their blood and organs were collected. Blood samples were obtained by cardiac puncture and were immediately incubated with EDTA (2.5 mg/mL). Aliquots of these samples were used to measure systemic leukocyte changes, while other blood samples were centrifuged at 2800× *g* at 4°C for 10 minutes to isolate plasma. Plasma samples were stored at -80°C and used to measure inflammatory mediators.

#### Leukocyte alterations

To analyze changes in the total number of leukocytes, blood samples were diluted 1:10 in Turk’s solution (0.1% crystal violet dye in 2% acetic acid), and the cells were counted in a Neubauer chamber. Blood samples were also submitted for blood smear and stained with a Fast Panoptic stain kit. Differential cell counts were performed by analyzing 100 cells that were identified as lymphocytes, neutrophils or monocytes based on morphologic criteria.

#### Detection of inflammatory mediators

IL-6, TNF-α, MCP-1, IL-12, IFN-γ and IL-10 detection was performed using the BD Cytometric Bead Array (CBA) Mouse Inflammation Kit (BD Bioscience). IL-1β and IL-17 quantification was performed using the Mouse IL-1β ELISA (BD Bioscience) and Mouse IL-17 Duo3.2.43 ELISA (R&D Systems) kits, respectively. All assays were performed according to the manufacturer’s instructions. The results are expressed as pg/mL.

#### Histopathologic analysis

To analyze systemic histopathologic changes, Balb/c mice (n = 6) were inoculated i.p. with a sublethal dose or 2DL_50_ of *N*. *annulifera* venom. After euthanasia or death, animals were exsanguinated, and their brains, lungs, hearts, kidneys, spleens and livers were collected. To analyze local histopathological changes, Balb/c mice (n = 3) were injected subcutaneously with 10 μg of *N*. *annulifera* venom at a volume of 50 μL (sterile saline) in the plantar region of their left hindpaws. The contralateral hindpaws (control) were inoculated with 50 μL of sterile saline. Animals were euthanized at different time points (0, 20, 60, 240 and 1440 minutes) and had their hindpaws removed. The hindpaws and organs were fixed in a 10% formaldehyde solution for 24 hours, submitted for routine histology and stained with hematoxylin and eosin (HE). All samples were analyzed with a light microscope and were examined for the presence of necrosis, edema, inflammatory infiltrates or hemorrhagic foci.

### Experimental antivenom production

#### Immunogenicity

H_III_ mice (n = 12) were immunized subcutaneously with 10 μg of *N*. *annulifera* venom diluted 1:25 on Al(OH)_3_ to achieve a final volume of 200 μL. These animals received three booster doses (5 μg) at 20-day intervals. Control animals were inoculated with sterile saline. Bleeding was performed 10 days after each venom inoculation. Blood was allowed to clot at room temperature for 15 minutes and was then left at 4° C for 6 hours. After centrifugation at 252× *g* and 4° C, the sera were collected and immediately frozen at -20° C.

#### Sera antibody titers

To evaluate the sera antibody titers of immunized animals, ELISA plates (Costar) were coated with 100 μL of *N*. *annulifera* venom (10 μg/mL) and incubated overnight at 4° C. The plates were then blocked with 5% BSA in PBS for 2 hours at 37° C and incubated with crescent dilutions of non-immune or experimental sera samples for 1 hour at room temperature. After incubation, the plates were washed with PBS-Tween 20 0.05%, and incubated with the IgG-HRPO (1:5000 diluted) secondary antibody in 1% BSA/PBS for 1 hour at 37° C. The plates were then washed, and the reactions were developed with OPD substrate according to the manufacturer’s instructions. The optical densities were obtained using spectrophotometry (Multiskan EX, Labsystems, Finland) at λ492 nm.

#### Recognition of venom components using experimental antivenom

Samples of *N*. *annulifera* venom (15 μg) were separated using 8–16% gradient SDS-PAGE [43] and blotted on nitrocellulose membranes [45]. The membranes were blocked for 2 hours with 5% BSA in PBS, washed three times with PBS, incubated with normal or experimental sera pools, and diluted 1:5000 in 0.01% PBS/BSA for 1 hour at room temperature. After incubation, the membranes were washed with PBS-0.05% Tween 20, incubated with IgG-AP secondary antibodies, and diluted 1:5000 in 1% BSA/PBS for 1 hour at room temperature. After incubation, the membranes were washed three times in PBS-0.05% Tween 20. Antigenic reactivity was measured by the addition of NBT and BCIP substrates according to the manufacturer’s recommendations.

#### Serum neutralization of the venom’s lethal activity

To determine the neutralizing potential of the experimental antivenom, samples of the venom, equivalent to 2LD_50_ (188.28 μg), were incubated with saline or crescent dilutions of the experimental serum for 30 minutes at 37° C. These mixtures were then i.p. administered to Balb/c mice (n = 6) (200 μL). Animals were observed for 72 hours and the death/survival ratio recorded. The neutralizing potential was calculated by Probit analysis and the results expressed as potency (mg of venom neutralized by mL of experimental serum) [56].

### Statistical analysis

Statistical analysis was performed using one-way ANOVA followed by Tukey’s Multiple comparison test, two-way ANOVA followed by a Bonferroni multiple comparison or *t-*tests. All statistical analyses were performed using Graphpad Prism 5 software (La Jolla, California, USA). Differences were considered significant when p ≤ 0.05.

## Results

### *N*. *annulifera* venom composition

**Fig 1:**
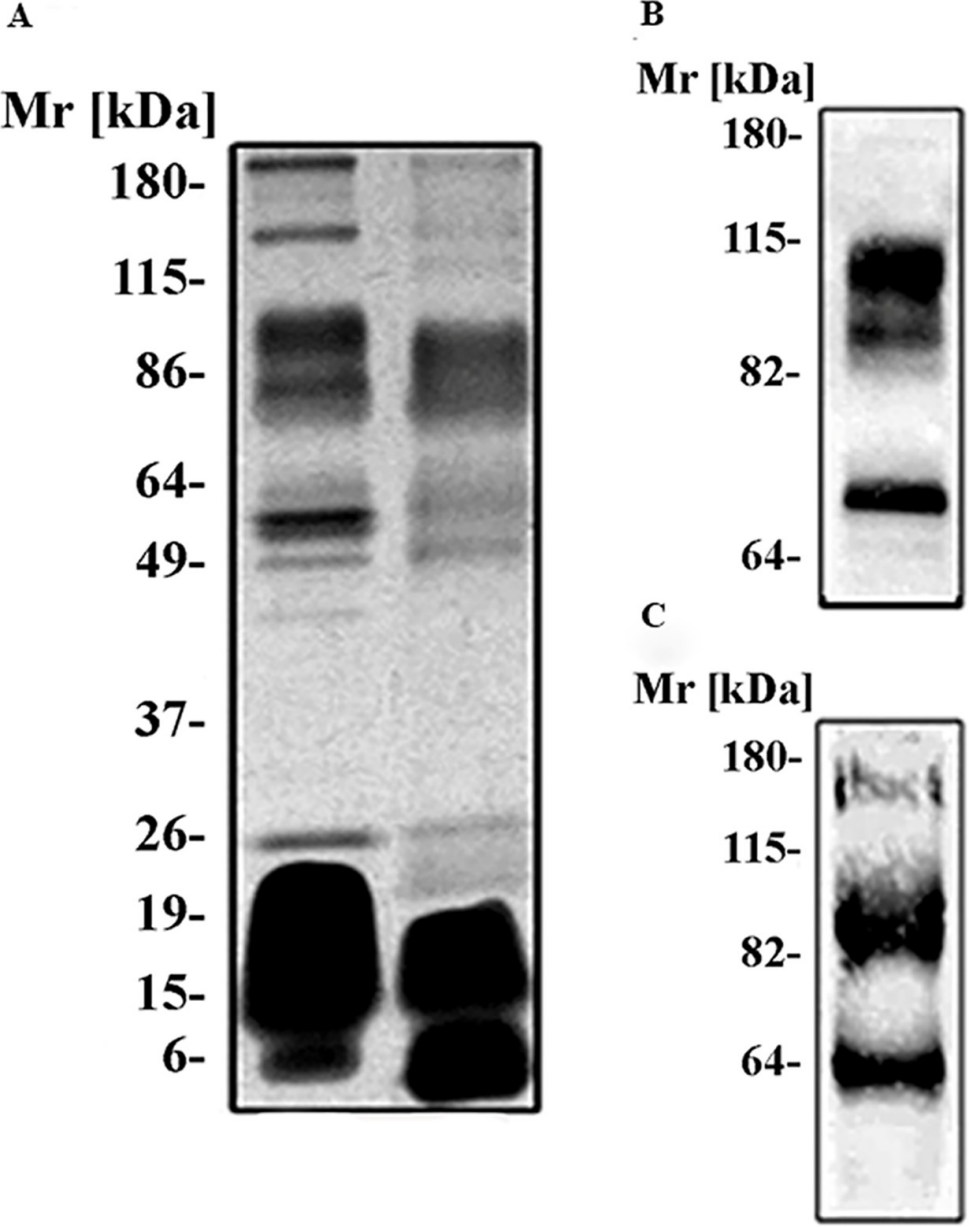
Characterization of the venom components. [A] SDS-polyacrilamide gel electrophoresis of *N*. *annulifera* venom. Venom samples (15 μg) were separated using a 8–16% SDS-polyacrylamide gel under non-reducing [NR] or reducing [R] conditions and silver stained. Analysis of the presence of [B] N-acetylglucosamine and [C] Mannan residues in the venom. Samples of *N*. *annulifera* venom (15 μg) were separated via SDS-PAGE and blotted onto a nitrocellulose membrane. The membranes were then incubated with the lectins WGA [B] or ConA [C], and the reactions were revealed with DAB plus H_2_O_2_.

The analysis of the electrophoretic profile of *N*. *annulifera* venom using a 8–16% SDS-polyacrylamide gel, under non-reducing conditions, revealed protein bands with relative molecular masses between ~10 kDa and ~190 kDa. Under reducing conditions, the venom displayed a more simple profile, with main protein bands ranging from ~6 kDa to ~100 kDa (Fig 1A), indicating that the venom contains protein in oligomeric state. To better characterize venom proteins, nitrocellulose membranes to which venom samples had been transferred were incubated with the ConA and WGA lectins, which recognize mannose [64] and N-acetylglucosamine [65] residues, respectively. Fig 1B shows three protein bands (~70, ~86 and ~103 kDa) that interacted with WGA indicating the presence of N-acetylglucosamine. Moreover, three bands that interacted with ConA (~63, ~81 and ~170 kDa) indicated that these proteins contain mannose residues in their carbohydrate moieties (Fig 1C).

The venom proteomic analysis using in-solution trypsin digestion and LC-MS/MS resulted in the identification of 79 proteins, including 24 types of venom components with or without enzymatic function, and six proteins of unknown function. Despite the variety of proteins identified in the venom, it is dominated by the presence of 3FTx (27 proteins) (Table 1; S1 and S2 Tables; S1 Spectra). Other protein classes identified in the venom include cysteine-rich secretory protein (CRISP), Kunitz-type protease inhibitor, snake venom metalloproteinase (SVMP), snake venom serine proteinase (SVSP), phospholipase A2 (PLA_2_), hyaluronidase (glycosyl hydrolase 56 family) and Cobra Venom Factor (venom complement C3 homolog).

**Table 1:** List of proteins identified in *Naja annulifera* venom by in-solution trypsin digestion and LC-MS/MS analysis*.

Protein family	Protein ID**	Protein	# Peptides
Snake three-finger toxin family	P01454	Cytotoxin 9	14
	P62394	Cytotoxin 11	6
	P01421	Short neurotoxin 4	10
	P68419	Short neurotoxin 1	12
	P01456	Cytotoxin 1	13
	Q9W717	Neurotoxin-like protein NTL2	2
	P01463	Cytotoxin 2	7
	P01453	Cytotoxin 10	10
	P29181	Weak neurotoxin 7	2
	P01401	Weak toxin CM-11	8
	P01399	Weak toxin CM-13b	4
	P01462	Cytotoxin 2	11
	P81782	Bucandin	3
	P01400	Weak toxin S4C11	6
	P25674	Long neurotoxin 1	7
	P25678	Weak toxin CM-2a	3
	P01457	Cytotoxin 5	6
	Q9W6W6	Cytotoxin 10	5
	P01422	Short neurotoxin 2	7
	P82462	Muscarinic toxin-like protein 1	2
	R4FK68	3FTx-Pse-105	1
	P01461	Cytotoxin 4	7
	R4G7H8	3FTx-Fur-10	1
	P01464	Cytotoxin 5	6
	P01388	Long neurotoxin 2	3
	P01389	Long neurotoxin 1	5
	P01426	Short neurotoxin 1	5
CRISP family	Q7T1K6	Cysteine-rich venom protein natrin-1	6
	P84808	Cysteine-rich venom protein kaouthin-2	5
	P0DL15	Cysteine-rich venom protein annuliferin-b (Fragment)	5
	P84807	Cysteine-rich venom protein 25-A (Fragment)	1
	Q8JI38	Cysteine-rich venom protein latisemin	2
	F2Q6F2	Cysteine-rich seceretory protein Dr-CRPK	2
	C1JZW4	Opharin	2
	Q7ZT98	Cysteine-rich venom protein ophanin	5
Phospholipase A2 family	B2BRS4	Truncated putative phospholipase A2	1
	P25498	Acidic phospholipase A2 E	1
	Q92085	Neutral phospholipase A2 B	1
	P00605	Phospholipase A2 basic	2
	P00600	Acidic phospholipase A2 DE-II	4
SVMP family	D6PXE8	Zinc metalloproteinase-disintegrin-like atrase-B	5
	D5LMJ3	Zinc metalloproteinase-disintegrin-like atrase-A	7
	Q10749	Snake venom metalloproteinase-disintegrin-like mocarhagin	6
	P82942	Hemorrhagic metalloproteinase-disintegrin-like kaouthiagin	4
	D3TTC2	Zinc metalloproteinase-disintegrin-like atragin	6
Type-B carboxylesterase/lipase family	A0A098LYB5	Carboxylic ester hydrolase	5
	R4FKE6	Carboxylic ester hydrolase (Fragment)	4
	Q92035	Acetylcholinesterase	3
	A0A098LY86	Carboxylic ester hydrolase (Fragment)	2
Phosphodiesterase family	A0A2D0TC04	Snake venom phosphodiesterase (PDE)	14
	A0A0F7YYZ8	Phosphodiesterase	9
Lectin family	U3FVL3	Vesicular integral-membrane protein VIP36	1
	R4G314	LP-Pse-6	1
Flavin monoamine oxidase family	A0A2R4N4Q6	Amine oxidase (Fragment)	14
	R4FID0	Amine oxidase	8
NGF family	P61899	Venom nerve growth factor	7
	Q5YF89	Venom nerve growth factor 2	4
Ohanin/vesprin family	P82885	Thaicobrin	6
	A0A182C6D0	Ohanin	4
5'-nucleotidase family	U3FYP9	Ecto-5-nucleotidase 1c	13
Ankyrin repeat	V8NWT6	Ankyrin repeat domain-containing protein 34B (Fragment)	1
Carbohydrate/starch-binding module (family 21)	V8N6R7	Protein phosphatase 1 regulatory subunit 3A (Fragment)	1
DEATH domain, DD	V8P0T5	Tumor necrosis factor receptor superfamily member 11B	3
Endonuclease family	V8N4Y2	Endonuclease domain-containing 1 protein	5
Glutathione peroxidase family	V8P395	Glutathione peroxidase (Fragment)	10
Glycosyl hydrolase 56 family (hyaluronidase)	U3FYQ4	Hyaluronidase	3
LDH/MDH superfamily	V8P6K5	L-lactate dehydrogenase	1
Pyruvate kinase family	V8P2C8	Pyruvate kinase	1
SVSP family	A8QL57	Snake venom serine protease BmSP (Fragment)	2
Thiolase family	V8NMW3	Non-specific lipid-transfer protein	1
Tudor domain	V8NEV9	Tudor domain-containing protein 6 (Fragment)	1
Venom complement C3 homolog family	Q91132	Cobra venom factor	18
Venom Kunitz-type family	P00986	Kunitz-type serine protease inhibitor 2	5
Unknown	A0A2D4GN98	Uncharacterized protein (Fragment)	1
	V8NUN2	Transmembrane protein 14C	1
	A0A2D4JG71	Uncharacterized protein (Fragment)	1
	A0A2D4KYJ8	Uncharacterized protein (Fragment)	1
	A0A2D4H759	Uncharacterized protein (Fragment)	1
	A0A2D4IS21	Uncharacterized protein (Fragment)	1

*Only proteins identified in at least two experimental replicates, with at least 1 unique peptide and posterior error probability ≤ 0.01 are listed.

**The first protein entry was selected as a representative of each Protein Group as identified using the MaxQuant software package.

### Toxic-Enzymatic properties of *N*. *annulifera* venom

**Fig 2:**
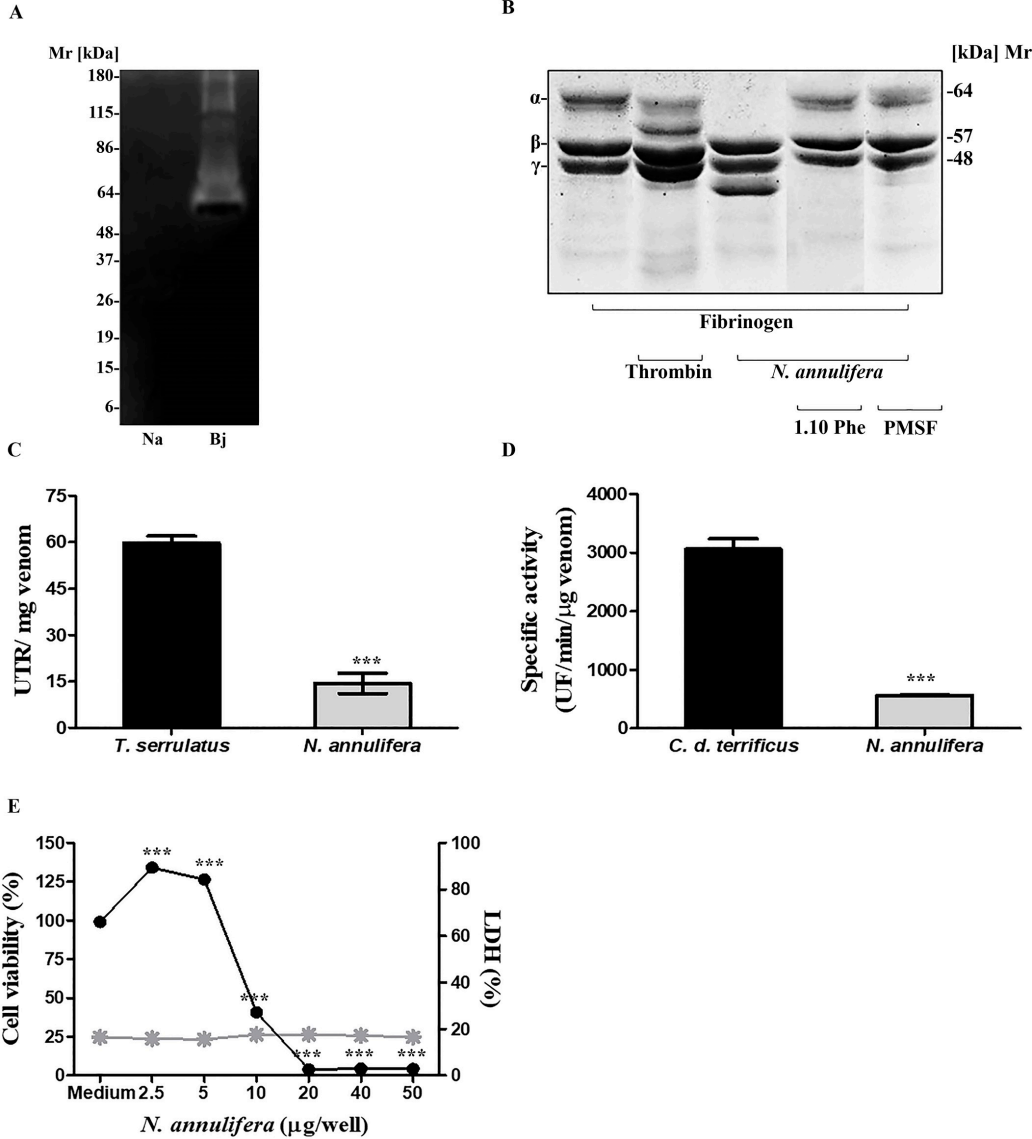
Toxic-enzyme properties of *N*. *annulifera* venom. [A] Zymography: Samples of *N*. *annulifera* venom (30 μg) were assessed via 10% SDS-PAGE in the presence of 1 mg/mL gelatin and then incubated with the substrate buffer. Gels were stained with *Comassie Brilliant Blue*. The venom of *B*. *jararaca* (10 μg) was used as a positive control. [B] Fb cleavage: Fb samples (30 μg) were incubated with *N*. *annulifera* venom (5 μg) with or without metallo- (10 mM) and serine proteinase (10 mM) inhibitors for 1 hour. Samples were then analyzed via SDS-PAGE (8–16% gradient gel) and stained with *Comassie Brilliant Blue*. [C] Hyaluronidase activity: samples of *N*. *annulifera* venom (20 μg) were incubated at 37°C for 15 minutes in a solution containing hyaluronic acid. After the incubation, the reactions were stopped with CTAB and the absorbances were measured at λ 405 nm with a spectrophotometer. As a positive control, *T*. *serrulatus* (20 μg) scorpion venom was used. The results are representative of three separate experiments and expressed as UTR/mg of venom ± SD. Statistical analysis was performed using the *t-test* (*** *p≤* 0.05). [D] PLA_2_ activity: Samples of *N*. *annulifera* venom (0.5 μg) were incubated at 37°C with a phospholipid mix that contained 10 mM phosphatidylcholine and 10 mM phosphatidylglycerol. Increased fluorescence was measured for 10 minutes. As a positive control, *C*. *d*. *terrificus* venom (0.5 μg) was used. The results are representative of three separate experiments and expressed as specific activity (UF *per* μg of venom *per* minute) ± SD. Statistical analysis was performed using *t-test* (*** *p≤* 0.05). [E] Cytotoxic activity: The HaCat human keratinocyte cell lineage was cultured in DMEM medium and incubated during 72 hours with increasing amounts of *N*. *annulifera* venom. The effect on cell viability was evaluated using the MTT method and by measuring LDH release from human keratinocytes exposed to the venom, using the CytoTox 96 Non-Radioactive Cytotoxicity Assay Kit. Statistical analysis was performed using One Way ANOVA (*** *p≤* 0.05) ± SD.

*N*. *annulifera* venom showed no proteolytic activity on zymography using gelatin as substrate. Under the same experimental conditions, proteins from the positive control of *B*. *jararaca* venom showed gelatinolytic activity, as demonstrated by clear regions in the gel (bands with molecular mass above 60 kDa) (Fig 2A). However, when proteolytic activity on fibrinogen was assessed, it was observed that the venom contained proteinases that were able to cleave this protein at the alpha chain, generating a fragment with a molecular mass of ~ 40 kDa (Fig 2B). In addition, when inhibitors were added to the reactions, cleavage was inhibited by 1, 10 Phe and PMSF, demonstrating the contributions of SVMP and SVSP to this hydrolysis (Fig 2B).

The hyaluronidase activity of *N*. *annulifera* snake venom was assessed via a turbidimetric assay, and the results are expressed in UTR. It was demonstrated that *N*. *annulifera* venom has a low hyaluronidase activity (UTR = 14.4) compared to the positive control *T*. *serrulatus* scorpion venom (UTR = 52.8) (Fig 2C).

PLA_2_ activity was measured with a fluorimetric assay, and the results obtained are expressed as specific activity (SA: UF/min/μg venom). Fig 2D shows that *N*. *annulifera* snake venom has significantly lower phospholipase activity (SA: 566.2) than the *C*. *d*. *terrificus* venom positive controls (SA: 3061.3).

Cytotoxic properties of the venom were evaluated by cell viability and LHD release assays. *N*. *annulifera* venom was able to reduce human keratinocyte viability in a dose-dependent manner but was not able to promote LDH release by the cells (Fig 2E).

### The impact of *N*. *annulifera* on hemostasis

*N*. *annulifera* venom induced disturbances in the Activated Partial Thromboplastin Time in a dose dependent manner (Table 2). Platelet-poor plasma (PPP) samples were incoagulable when the highest venom concentrations were used (12.5 to 50 μg), while the coagulation time (seconds) was significantly prolonged at lower concentrations (2.5 to 6.25 μg) (*p≤* 0.05). Moreover, the *R-time* shows that the disturbances promoted by *N*. *annulifera* venom were severe, as they were much higher than normal. Prothrombin Time assays demonstrated that the venom led to a significant prolongation in the coagulation time of PPP at the highest venom concentrations, *i*.*e*., 25 and 50 μg. Besides that, both venom concentrations were able to cause a significant alteration in the *R-time* (Table 2).

**Table 2:** Alterations in aTTP and PT induced by *N*. *annulifera* venom.

Assay	C	2.5 μg	3.12 μg	6.25 μg	12.5 μg	25 μg	50 μg
**aTTP**	38	78.4***	92.8***	98.9***	-***	-***	-***
***R-time***	1	2.1***	2.3***	2.3***	6.3***	6.3***	6.3***
**PT**	15.5	15.5	15.5	15.5	15.3	18.3**	33.6***
***R-time***	1	1	1	1	1	1.33**	2.2***

aTTP: Activated Partial Thromboplastin Time; PT: Prothrombin Time; -: Incoagulable; C: Control (PPP samples treated with sterile saline); *R-time*: scale of abnormalities in coagulation time. Statistical analysis was performed using Two Way ANOVA (** and *** *p≤* 0.05) ± SD.

### Toxicity of *N*. *annulifera* venom in the murine model

#### Local reactions

**Fig 3:**
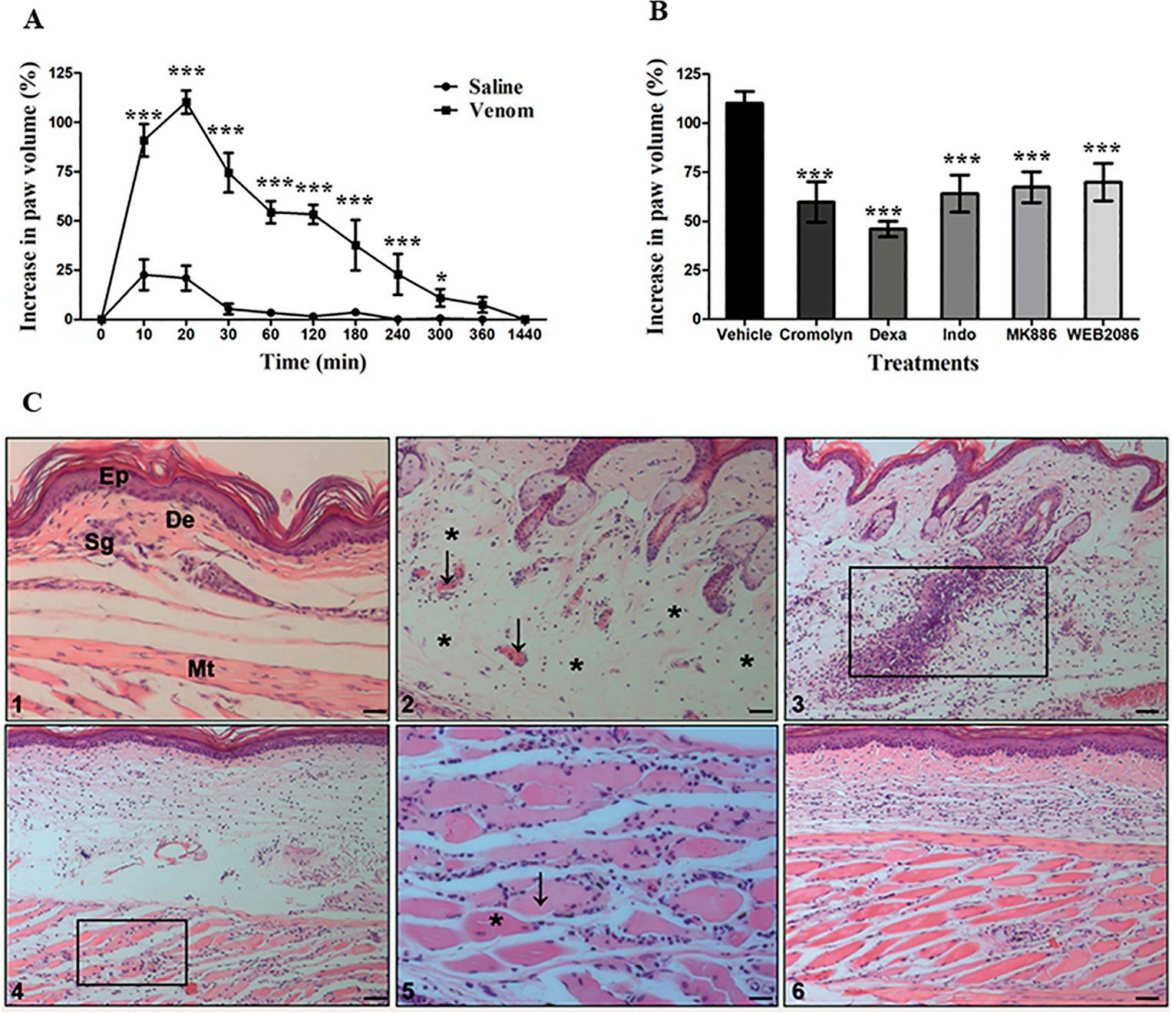
Local alterations induced by *N*. *annulifera* venom. [A] Edema: samples of venom (10 μg/50 μL) or sterile saline were injected into the left or right hind footpad, respectively, of Balb/c mice (n = 6). Paw edema was assessed by measuring the paw thickness using a caliper rule before (T0) and after inoculation with venom or saline (Te). The increased paw volume was expressed as percentage (%) and was calculated with the following formula: (Te-T0)/T0*100. Statistical analysis was performed using two-way ANOVA, followed by the Bonferroni multiple comparison test (***p≤ 0.05) ± SD. [B] The effect of inhibitors on the paw edema induced by *N*. *annulifera* venom: groups of Balb/c mice (n = 6) were treated with different inhibitors before induction of edema with venom: Cromolyn, a mast cell degranulation inhibitor (10 mg/kg, three days consecutively before the edema, i.p. route); dexamethasone (Dexa), a cPLA_2_ inhibitor (2 mg/kg, 2 hours before the edema, i.p. route); indomethacin (Indo), a COX isoform inhibitor (10 mg/kg, 30 minutes before the edema, i.p. route); MK886, a 5-lipoxygenase-activating protein inhibitor (5 mg/kg, 30 minutes before the edema, i.p. route); and WEB2086, a PAFR antagonist (5 mg/kg, 1 hour before the edema, s.c. route). After treatment, edema was induced, and the paws were measured with a caliper rule at several time points. The increased paw volume was expressed as a percentage. Statistical analysis was performed using two-way ANOVA, followed by the Bonferroni multiple comparison test (***p≤ 0.05) ± SD. [C] Histopathological changes promoted by *N*. *annulifera* venom: samples of *N*. *annulifera* venom (10 μg/50 μL) were injected into the left hind paw of Balb/c mice (n = 6). The contralateral paws were injected with sterile saline (50 μL) to serve as controls. The animals were euthanized at different time points, and their paws were removed and submitted for histopathological analysis. Control group [Panel 1]: epidermis (Ep), dermis (De), sweat glands (Sg) and muscle tissue (Mt). Experimental group [Panel 2]: arrows highlight vascular congestion and a perivascular inflammatory infiltrate; asterisks indicate areas of edema. The rectangle indicates areas of neutrophil infiltration [Panel 3] and myonecrosis [Panels 4, 5]. The arrow and asterisk in Panel 5 indicate dead and living cells, respectively. Scale = 10μm.

To evaluate the edematogenic activity of *N*. *annulifera* venom, Balb/c mice were inoculated with 10 μg of venom into their left hind footpads, and their paws were measured at different time points. The venom was able to induce a significant rapid onset of edema, with its maximum peak (110.13% of increase on paw volume) reached 20 minutes after inoculation. It returned to basal level 24 hours after venom inoculation (Fig 3A).

In addition to the evaluation of edematogenic activity, the contribution of mast cell and lipid mediators to edema development was also assessed. It was found that mast cell degranulation is important for the earliest development (10–30 minutes) of edema as treatment with Cromolyn, a mast cell degranulation inhibitor, was able to reduce swelling (S1A Fig). Dexamethasone, a synthetic corticosteroid compound with a potent anti-inflammatory activity, had a strong effect on the paw edema induced by *N*. *annulifera* venom. It was able to decrease hindpaw thickness throughout the duration of edema, mainly during the early stages (10–30 minutes), suggesting that lipid mediators participate in the development of edema (S1B Fig). Indomethacin, a non-selective COX inhibitor, was also able to reduce the degree of edema (10 to 120 minutes), suggesting that prostaglandins and thromboxanes may contribute to the local reaction to the venom. However, treatment with Indomethacin was not able to control swelling during the late phase (180 to 1440 minutes) (S1C Fig). A similar effect was observed in animals treated with a FLAP inhibitor (S1D Fig). The PAFR blocker WEB-2086 demonstrated inhibitory action only in the earliest phases of the edema (20–30 minutes) (S1E Fig). Although all inhibitors showed different modulation profiles, they were able to significantly decrease the edema at its peak [Fig 3B].

Histopathologic analyses showed that the venom promotes several changes, ranging from mild to intense, at all time periods [Fig 3C]. After inoculation with the venom, it was observed that it promotes an acute inflammatory response at the inoculation site, with a disorganized dermal collagen matrix, subcutaneous edema, vascular congestion [Panel 2], mild erythrocyte extravasation and neutrophil migration to the dermis and muscle [Panels 3, 4, 5]. Myonecrosis was also observed [Panels 4, 5] along with polymorphonuclear cell infiltration. These alterations persisted for several hours. However, after 24 hours of inoculation, a decrease in the subcutaneous edema was observed, along with the absence of vascular congestion and few remaining neutrophils in the tissue. By contrast, an increased number of mononuclear cells and reorganization of the dermal collagen matrix was observed [Panel 6].

### Lethal dose

LD_50_ was determined 72 hours after venom administration. Prior to their death, animals presented with several clinical findings of envenomation, including apathy, bending at the column, a rough hair coat, dyspnea and paralysis of their hind limbs. LD_50_ was calculated with a probit analysis with a 95% confidence interval and was found to be 94.14 μg (68.78–115.74).

### Systemic reactions induced by *N*. *annulifera* venom

**Fig 4:**
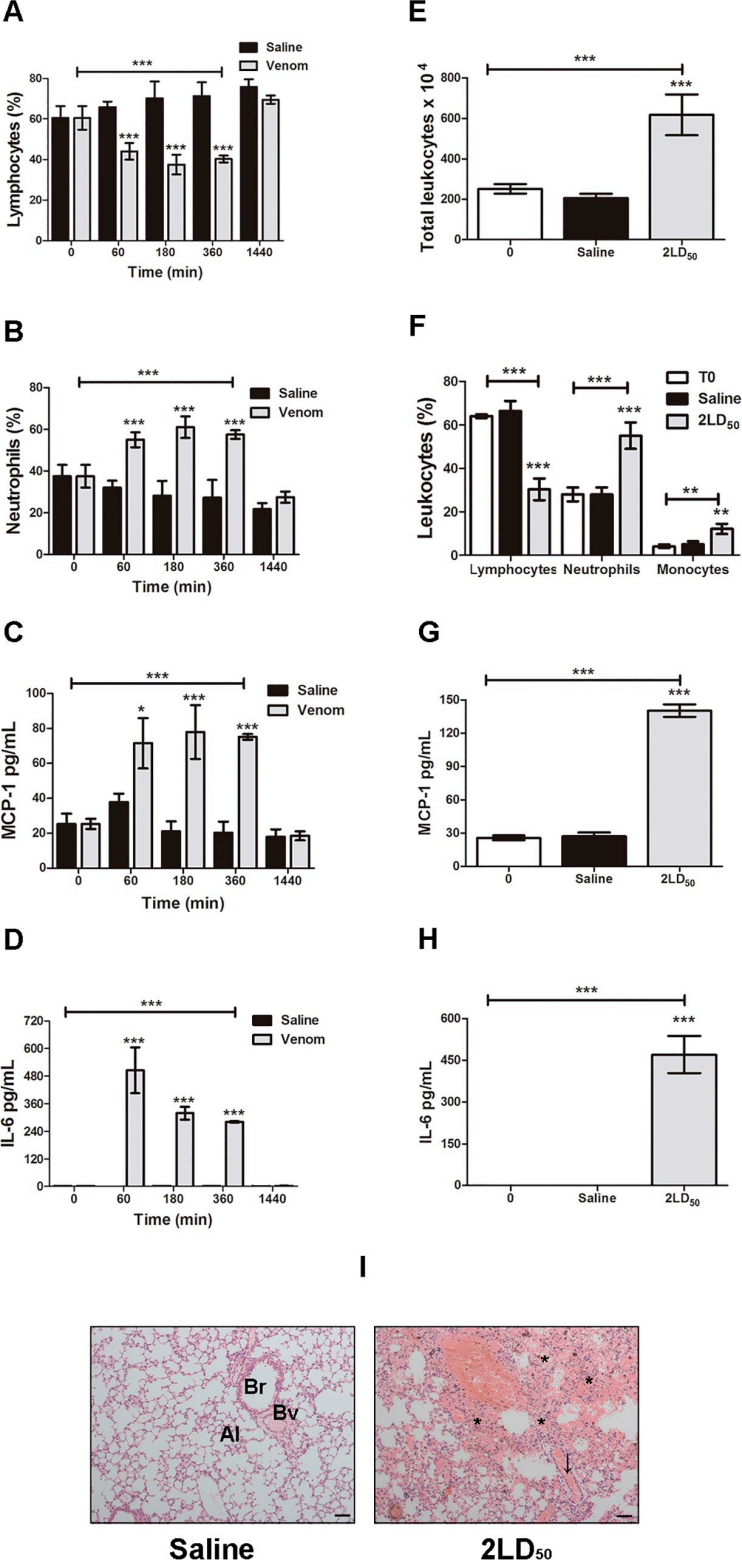
Systemic changes promoted by *N*. *annulifera* venom. Balb/c mice were injected with either a [A-D] sublethal (56.48 μg) dose or [E-I] 2LD_50_ (188.28 μg) of venom intraperitoneally. After death, animals were exsanguinated and some organs were fixed in a 10% formaldehyde solution and submitted for histologic analysis. [A, B, F] Leukocyte alterations: blood samples obtained from the animals were diluted in Turk’s solution or submitted for either a blood smear or Fast Panoptic stain to analyze total and differential leukocyte alterations. [I] Histopathological analysis: tissues were analyzed under a light microscope to detect histological alterations. Lungs: bronchiole (Br), blood vessel (Bv), alveoli (Al). Arrow and asterisk indicate vascular congestion and multifocal hemorrhage, respectively. Scale = 10μm. [C, D, G, H] Increased plasma levels of inflammatory mediators: plasma samples were submitted for CBA (BD Bioscience PharMingen, EUA) or ELISA (BD Bioscience PharMingen, EUA) (R&D Systems) according to the manufacturer’s instructions. The results are expressed as pg/mL. Statistical analyses were performed using Graphpad Prism by means of a 2-way ANOVA followed by the Bonferroni multiple comparison test (***p≤ 0.05) ± SD.

The systemic reactions promoted by the venom were evaluated using two different experimental protocols. By injecting 60% (56.48 μg) (Sublethal dose) of LD_50_ of *N*. *annulifera* venom, it was observed that animals presented several clinical findings of envenomation, including apathy, bending at the column, a rough hair coat, dyspnea and difficulty walking. This dose was also able to cause alterations in some systemic parameters, such as a decrease of circulating lymphocytes [Fig 4A] and an increased number of circulating neutrophils [Fig 4B]. Nonetheless, this dose was not able to lead to changes in the total number of leukocytes and histological organs damage. However, a sublethal dose promoted an increase in the plasma levels of MCP-1 [Fig 4C] and IL-6 [Fig 4D]. These alterations persisted for several hours. However, 24 hours after experimental envenomation, all of these parameters returned to their normal values.

Injection of 2LD_50_ led to several clinical findings of envenomation, such as apathy, bending at the column, a rough hair coat, dyspnea, hind limb paralysis and death. In addition to these clinical findings, histopathologic alterations were observed in the lungs, among these, moderate vascular congestion and multiple hemorrhagic foci [Fig 4I]. Furthermore, the animals demonstrated leukocytosis [Fig 4E] characterized by neutrophilia and monocytosis [Fig 4F], as well as increased plasma levels of MCP-1 [Fig 4G] and IL-6 [Fig 4H].

### *N*. *annulifera* venom immunogenicity and antivenom production

**Fig 5:**
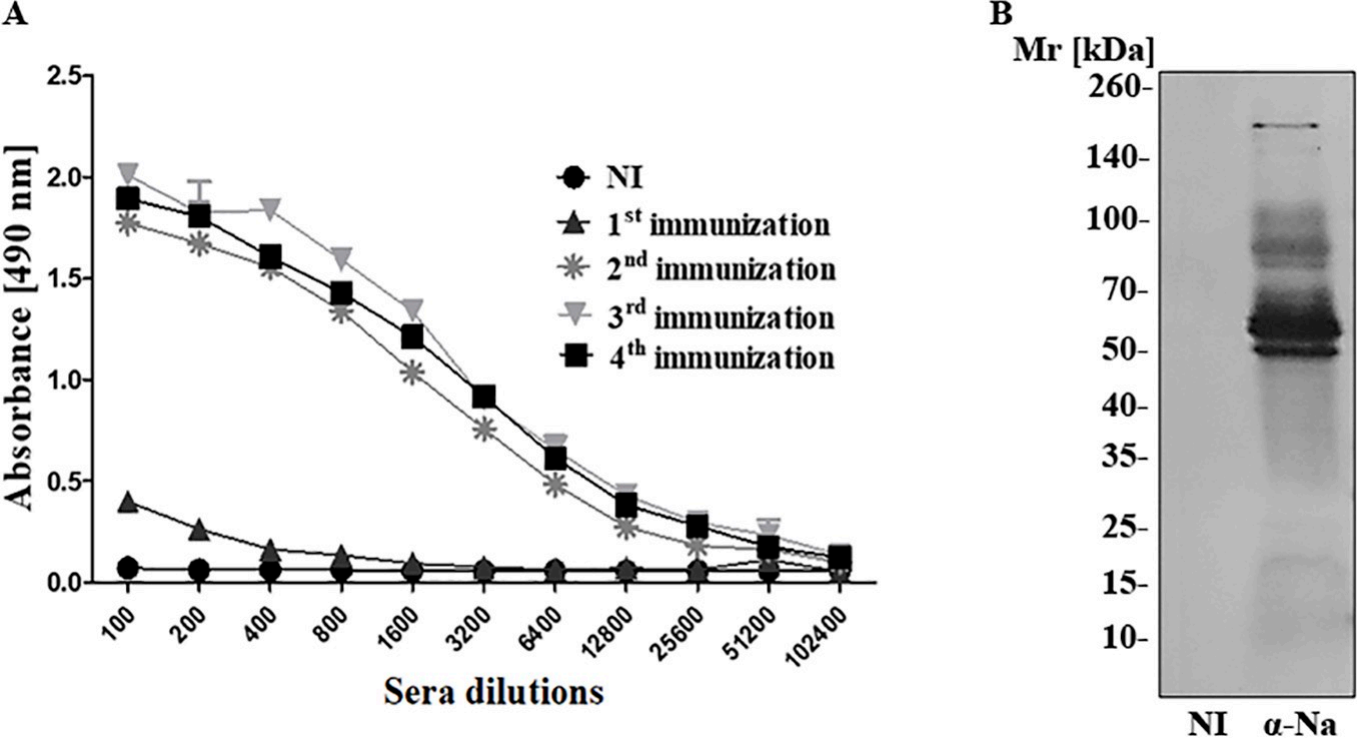
*N*. *annulifera* venom immunogenicity, antivenom production and serum neutralization. [A] Antibody titers: ELISA plates were coated with 10 μg/mL venom (100 μL/well), incubated with increasing dilutions of non-immune (NI) or experimental sera obtained from H_III_ mice, and incubated with anti-mouse HRPO-conjugated IgG (1:5000). The reaction was performed with the addition of OPD and H_2_O_2_, and spectrophotometric readings were taken at λ 492 nm. [B] Western Blot: samples of the venom (15 μg) were separated via 8–16% gradient SDS-PAGE, blotted onto nitrocellulose membranes and incubated with the experimental serum diluted to 1:5,000 (α Na). The membranes were incubated with conjugated anti-mouse IgG-AP (1:7,500), and the reactions were started by adding NBT/BCIP.

The immunogenicity of *N*. *annulifera* snake venom was evaluated in H_III_ mice immunized with 10 μg of venom. The antibody titers were determined by ELISA. Fig 5A presents the antibody response over time. *N*. *annulifera* venom is highly immunogenic, promoting the production of high antibody titers, already after the second immunization dose. The recognition profile of the venom proteins by the experimental *N*. *annulifera* mouse antiserum was determined by Western Blot. Fig 5B shows that the experimental serum was able to recognize venom components, mainly proteins with Mr above 50 kDa. Moreover, as determined by Probit analysis, it was shown that this antivenom was able to neutralize the *N*. *annulifera* venom lethal activity with a high potency, *i*.*e*., 1 ml of antivenom was able to neutralize 4.5 mg of venom.

## Discussion

In this study, we characterized some of the biochemical, toxic, immunogenic and physiopathologic properties of the venom from *N*. *annulifera*, which is a medically important snake related to accidental bites in the countries of Sub-Saharan Africa. The results show that *N*. *annulifera* venom contains several toxic components able to induce systemic inflammation, which may contribute to the pathology observed in envenomed individuals. Moreover, the venom is immunogenic, an important feature that must be considered during the production of a therapeutic anti-*N*. *annulifera* antivenom.

Electrophoretic analysis *of N*. *annulifera* venom showed that it contains several components, including low molecular mass proteins, which suggested the presence of neurotoxins [18], among these, 3FTx [14, 15] and PLA_2_ [16]. The presence of these components in the venom was confirmed by trypsin digestion of proteins and LC-MS/MS analysis. These components may be responsible for some of the clinical findings observed during envenomation, such as heart damage and systemic inflammation in dogs [35], as well as respiratory arrest in humans [23]. The data from this study corroborate the results of other studies, which showed the presence of these components in *N*. *annulifera* venom [37, 38, 39, 40, 41, 42]. Envenomed individuals from South Africa showed local dermonecrotic injury after a bite, which could be caused by the high content of cytotoxins, as shown in our LC-MS/MS analysis. In accordance with data from Panagides and colleagues [66], here we also showed that *N*. *annulifera* venom could promote decrease in human epidermal cells viability, as evaluated by the MTT method. However, it was not possible to detect release of LDH by these cells, which possibly indicate that the keratinocyte membranes were not damaged and that the cell death promoted by *N*. *annulifera* could be due to apoptosis [67] and not necrosis. Alternatively, it is possible to consider that *N*. *annulifera* venom contains components able to reduce mitochondrial activity, since MTT method evaluates cell viability as enzymatic conversion of the tetrazolium compound to water insoluble formazan crystals by dehydrogenases occurring in the mitochondria of living cells.

Western Blot lectin analysis demonstrated the presence of mannose and N-acetylglucosamine residues in *N*. *annulifera* venom proteins. These carbohydrate residues were found in the venom proteins of different genera of snakes [46, 68, 69, 70], including in important toxic components, such as SVSP and SVMP [69, 70]. Although we have not identified the families of these glycosylated components, it is possible that some of them are linked to SVMPs or SVSPs, as they have predicted molecular masses similar to some of the previously described glycosylated proteolytic enzymes from viperid venoms [69, 70]. Moreover, SVMPs and SVSPs were detected in *N*. *annulifera* venom by LC-MS/MS. Proteins containing these carbohydrate residues are also present in many pathogens, such as bacteria and fungi, and can be recognized by different immune cells and molecules, triggering inflammatory and immune responses [71, 72]. This recognition could contribute to the clinical manifestations observed during envenomation, such as the systemic inflammation observed in dogs [35].

Functional biochemical assays were performed to confirm the presence of some of the toxic-enzymatic components found in the LC-MS/MS analysis. The presence of proteinases in animal venoms can contribute to different clinical manifestations during envenomation, such as inflammation, tissue damage, disturbances in coagulation and bleeding [73]. In elapid venoms, proteolytic activity is usually low or nonexistent [26, 74, 75]. In contrast to the data shown by Phillips et al. [76], under the experimental conditions used in this paper, *N*. *annulifera* venom did not show any proteolytic activity on either gelatin. However, although detected at low abundance in the proteomic analysis, the venom contains SVMP and SVSP able to cleave the fibrinogen alpha chain, which suggests that these proteinases can contribute to the hemostatic alterations as observed in dogs envenomated by *N*. *annulifera* [36].

Hyaluronic acid is cellular cement that, together with other components of the extracellular matrix, forms a protective gel that prevents the entry of foreign agents. A variety of animal venoms contain hyaluronidases, which cleave hyaluronic acid molecules to facilitate access of the venom from the tissue to the bloodstream. This enzyme is also called “spreading factor” [77], and its action can promote local and systemic inflammation by increasing tissue permeability [78]. Moreover, the products derived from hyaluronic acid cleavage, which can be recognized by immune receptors, such as TLR2 and 4, trigger the production of inflammatory mediators, such as cytokines and chemokines [79]. *N*. *annulifera* venom showed evidence of hyaluronidase activity, but it was relatively low compared with other elapid venoms from the *Naja* [74] and *Micrurus* [16] genera, and accordingly, only one hyaluronidase was identified by mass spectrometry in the venom.

PLA_2_ belongs to a superfamily of lipolytic enzymes that catalyze specific hydrolysis of the ester linkage at the *sn-2* position of glycerophospholipids, generating arachidonic acid and lysophospholipids. PLA_2_-like proteins found in snake venoms may be devoid of catalytic activity, although it may exhibit myotoxic or neurotoxic activities [80, 81]. Moreover, these enzymes may present with other toxic properties during envenomation, such as the ability to cause cytotoxicity and inflammation [82, 83]. As predicted by LC-MS/MS, the presence of PLA_2_ activity in *N*. *annulifera* venom was also observed in enzymatic assays. Nonetheless, *N*. *annulifera* venom exhibits a very lower content of PLA2 /enzymatic activity when compared to other *Naja* venoms [84, 85, 86, 87, 88], suggesting a strong interspecific variation associated to this particular toxin.

As observed in dogs, we showed here that *N*. *annulifera* venom promoted hemostatic disturbances in human plasma, making it incoagulable. These disturbances in the hemostatic system can be attributed to the fibrinogenolytic proteinases detected in the venom, or to PLA_2_ found in the LC-MS/MS analysis, since these components can promote plasma incoagulability via direct binding to FXa, thereby preventing thrombin generation [89, 90, 91, 92]. This phenomenon, promoted by several species of *Naja* venom, has been observed in different clinical and experimental studies [93, 94]. It is therefore very important to evaluate the mechanisms involved in these alterations and their consequence, since the current literature on the topic is scarce. In addition, these alterations may be a therapeutic target for *Naja* envenomation.

In our *in vivo* experimental model, the venom was able to induce swelling and several histopathologic changes in the hind paws of mice, that decreased only after 24 hours. Among these tissue alterations, myonecrosis associated with inflammation was observed, an event that is commonly found in experimental models of Elapidae envenomation, which is attributed to cytotoxins and PLA_2_ [95, 96].

The inflammatory events promoted by the venom may be attributed to hyaluronidase, PLA_2_, glycosylated proteins, SVSP and SVMP since they can promote different inflammatory events, which include complement activation, mast cell degranulation, release of eicosanoids and cytokines and leukocyte homing [16, 26, 27, 58, 59, 60, 61, 83]. Knowing that *N*. *annulifera* venom promotes inflammation and pain in humans and dogs [31, 32, 33, 34, 35, 36], which were also observed in our experimental model, pharmacologic studies were performed to analyze the role of some inflammatory mediators in the edema process. Pre-treating mice with different compounds that were able to modulate different steps of the inflammatory process, including, mast cell degranulation (Cromolyn), lipid mediator production (Dexamethasone, Indomethacin and MK-886) and action (WEB-2086) significantly decreased the edema promoted by *N*. *annulifera* venom. All of the compounds showed a similar pattern of inhibiting peak edema. However, cPLA_2_, COX isoforms and FLAP inhibitors controlled the edema for a long time, suggesting a stronger contribution of eicosanoids, such as prostaglandins, thromboxanes and leukotrienes, to this process. Moreover, it is possible that these lipid mediators may contribute to the pain observed in humans after a bite, making them good therapeutic targets for the local reactions promoted by *N*. *annulifera* venom.

The LD_50_ of *N*. *annulifera*, established here via i.p. route in Balb/c mice, was 94.14 μg. Ramos-Cerrillo and collaborators [56] showed that the *N*. *annulifera* venom LD_50_ when administered intravenously was 53.9 μg. To evaluate whether *N*. *annulifera* venom was able to promote systemic changes, a sublethal dose of the venom was established, and different inflammatory parameters were evaluated. As in dogs envenomed by *N*. *annulifera* [34], it was observed that a sublethal dose of the venom promotes acute systemic inflammation, which was characterized by neutrophilia and increased levels of IL-6 and MCP-1 in the plasma. However, unlike dogs envenomed by *N*. *annulifera* the sublethal dose was not able to cause organ injury, as observed by Langhorn et al. [35] in dogs.

By administering a superdose (2LD_50_) of venom to mice, it was possible to observe systemic inflammation, which was characterized by an increase in the plasma levels of IL-6 and MCP-1. However, in contrast with the sublethal dose, the 2DL_50_ dose caused leukocytosis, which was characterized by neutrophilia and monocytosis. In addition, dead animals showed multifocal hemorrhaging in their lungs.

These data suggest that the systemic inflammatory process induced by high doses of venom may be associated with the lung alterations observed in humans. In fact, in different models of hemorrhagic shock, plasma, pulmonary and hepatic increases in IL-6 and MCP-1 were observed along with inflammation and lung injury, which may culminate in acute respiratory distress syndrome [97, 98, 99]. It is important to emphasize that in addition to cytokines, some other factors may be associated with the pulmonary hemorrhaging and death caused by a venom overdose. Further, the hemostatic alterations promoted by SVMP, SVSP and PLA_2_ can also contribute to lung hemorrhage.

The treatment indicated for envenomation by *N*. *annulifera* is serum therapy. *N*. *annulifera* venom is part of the antigenic mixture used for the production of polyvalent serum by the South African Vaccine Producers (SAVP) (Pty) Ltd [28], although its immunogenicity and neutralizing potential have been poorly investigated. Here, we show that *N*. *annulifera* venom is highly immunogenic in murine model. Although with different intensities, this mouse antivenom was able to recognize venom components by Western Blot, mainly the ones with high molecular weight, which includes components as HYA, LAAO, CVF and SVMP. Moreover, this monovalent antivenom was able to protect the animals from death induced by venom, with high potency. In contrast, other studies have shown that horse antivenoms produced against venom mixtures, in which *N*. *annulifera* was included, were not able to neutralize the lethal effects of this venom [56, 100, 101]. This may be due to differences related to the animals used (mouse *versus* horse) or to a low level of neutralizing antibodies generated by other venoms present in the immunization pool.

In conclusion, here, we show that *N*. *annulifera* snake venom contains several components with toxic and pro-inflammatory properties. Some of these toxins promote coagulation disturbances, local and systemic inflammatory reactions, which may contribute to the pathologic events, observed in our murine model and possibly in dogs and humans envenomated by *N*. *annulifera*. Moreover, the venom promoted lung haemorrhage, an event that may also occur in cases of human envenomation, since death by respiratory arrest can be the result of the sum of neurotoxin activity and lung haemorrhage. High levels of IL-6 and MCP-1, as detected in the plasma of the envenomated animals, may be associated with pulmonary damage, since systemic inflammatory conditions can be deleterious and affect several organs, including the lungs. Thus, inflammation may be considered as target for the development of new therapeutic strategies in cases of *N*. *annulifera* human envenomation. Moreover, we showed that the venom is highly immunogenic and that the experimental serum was able to neutralize its lethal activity in the murine model. These data encourage further studies to characterize and produce monospecific therapeutic antivenom against *N*. *annulifera*.

## Supporting information

S1 Table Identification of proteins in *N. annulifera* snake venom by trypsin digestion and LC-MS/MS analysis. (XLSX)

S2 Table Identification of protein families in *N. annulifera* snake venom by trypsin digestion and LC-MS/MS analysis. (XLSX)

S1 Spectra Unique peptides identified in the *N. annulifera* snake venom. (DOCX)

S1 Fig Pharmacologic modulation of the edema induced by *N. annulifera* snake venom. To evaluate the contribution of the different inflammatory mediators classes in hindpaw edema induced by *N*. *annulifera*’ s venom, Balb/c mice groups were treated with [A] Cromolyn, a mast cell degranulation inhibitor (10 mg/kg, three days consecutively before the edema, i.p. route); [B] dexamethasone (Dexa), a cPLA_2_ inhibitor (2 mg/kg, 2 hours before the edema, i.p. route); [C] indomethacin (Indo), a COX isoform inhibitor (10 mg/kg, 30 minutes before the edema, i.p. route); [D] MK886, a 5-lipoxygenase-activating protein inhibitor (5 mg/kg, 30 minutes before the edema, i.p. route) and [E] WEB2086, a PAFR antagonist (5 mg/kg, 1 hour before the edema, s.c. route). After treatment, edema was induced, and the paws were measured with a caliper rule at several time points. The increased paw volume was expressed as percentage. Statistical analysis was performed using two-way ANOVA, followed by the Bonferroni multiple comparison test (***p≤ 0.05) ± SD.
(TIF)

## References

[1] Gutiérrez JM, Theakston RDG, Warrel DA. (2006) Confronting the neglected problem of snake bite envenoming: the need for a global partnership. PloS Med 3:150. 10.1371/journal.pmed.0030150 PMC1472552 16729843

[2] Kasturiratne A, Wickremasinghe AR, de Silva N, Gunawardena NK, Pathmeswaram A, Premaratna R, et al (2008) The global burden of snakebite: a literature analysis and modeling based on regional estimates of envenoming and deaths. PloS Med 5: 218. 10.1371/journal.pmed.0050218 PMC2577696 18986210

[3] Harrison RA, Gutiérrez JM. (2016) Priority actions and progress to substantially and sustainably reduce the mortality, morbidity and socioeconomic burden of tropical snakebite. Toxins 24: 1–14. 10.3390/toxins8120351 PMC5198546 27886134

[4] The Reptile Database. Species number. 2018 [accessed Oct 18, 2018]. Available from: http://www.reptile-database.org/db-info/SpeciesStat.html

[5] Gold KS, Dart RC, Barish RA. (2002) Bites of venomous snakes. N Engl J Med. 10.1056/NEJMra013477 12151473

[6] Pough FH, Janis CM, Heiser JB. A vida dos vertebrados. 4^th^ ed São Paulo: Atheneu; 2008.

[7] The Reptile Database. Higher taxa in extant reptiles. 2018 [accessed Oct 18, 2018]. Available from: http://www.reptile-database.org/db-info/taxa.html

[8] The Reptile Database. Higher taxa: Elapidae. 2018 [accessed Oct 18, 2018]. Available from: http://reptile-database.reptarium.cz/advanced_search?taxon=Elapidae&submit=Search

[9] Slowinski JB, Knight A, Rooney AP. (1997) Inferring species trees from trees: a phylogenetic analysis of the Elapidae (Serpentes) based on the amino acid sequences of venom proteins. Mol Phylogenet Evol 8: 349–362. 10.1006/mpev.1997.0434 9417893

[10] Phui Yee JS, Nanling G, Afifiyan F, Donghui M, Siew Lay P, et al (2003) Snake postsynaptic neurotoxins: gene structure, phylogeny and applications in research and therapy. Biochimie 86: 137–149. 10.1016/j.biochi.2003.11.012 15016453

[11] Tytgat J, Vanderbeghe I, Ulens C, Beeumen JV. (2001) New polypeptide components purified from mamba venom. FEBS Lett 491: 282–283. 10.1016/s0014-5793(01)02201-3 11240130

[12] Takacs Z, Wilhelmsen KC, Sorota S. (2004) Cobra (*Naja spp*) nicotinic acetylcholine receptor exhibits resistance to erabu sea snake (Laticauda semifasciata) short-chain α- neurotoxin. J Mol Evol 58: 516–526. 10.1007/s00239-003-2573-8 15170255

[13] Tan NH, Tan CSA. (1988) A comparative study of cobra (*Naja*) venom enzymes. Comp Biochem Physiol 90: 745–750. 10.1016/0305-0491(88)90329-x 2854766

[14] Petras D, Sanz L, Segura A, Herrera M, Villalta M, et al (2010) Snake venomics of African spitting cobras: toxin composition and assessment of congeneric cross-reactivity of the pan- african echitab-plus-ICP antivenom by antivenomics and neutralization approaches. J Proteome Res 10: 1266–1280. 10.1021/pr101040f 21171584

[15] Tanaka GD, Furtado MFD, Portaro FCV, Sant’anna OA, Tambourgi DV. (2010) Diversity of *Micrurus* snake species related to their venom toxic effects and the prospective of antivenom neutralization. Plos Negl Trop Dis 4: 1–12. 10.1371/journal.pntd.0000622 PMC2834742 20231886

[16] Tanaka GD, Pidde-Queiroz G, Furtado MFD, Van Den Berg C, Tambourgi D. (2012) *Micrurus* snake venoms activate human complement system and generate anaphylatoxins. BCM Immunol 13: 1–7. 10.1186/1471-2172-13-4 PMC3398285 22248157

[17] Malih I, Ahmad Rusmili MR, Tee TY, Saile R, Ghalim N, Othman I. (2014) Proteomic analysis of Moroccan cobra *Naja haje legionis* venom using tandem mass spectrometry. J Proteomics 16: 240–252. 10.1016/j.jprot.2013.11.012 24269350

[18] Vejayan J, Khoon TL, Ibrahim H. Comparative analysis of the venom proteome of four Malaysian snake species. J Venom Anim Toxins Incl Trop Dis 20: 1–9. 10.1186/1678-9199-20-1 PMC4015498 24593956

[19] Oyama E, Takashi H. (2006) Distribuition of low molecular weight platelet aggregation inhibitors from snake venoms. Toxicon 49: 293–298. 10.1016/j.toxicon.2006.09.027 17141819

[20] Shweitz H, Vigne P, Moinier D, Frelin C, Lazdunski M. (1992) A new member of the natriuretic peptide family is present in the venom of the green mamba (*Dendroaspis angusticeps*). J Biol Chem 267: 13928–13932. 1352773

[21] Tonsing L, Potgieter DJJ, Louw AI, Visser L. (1983) The binding of snake venom cardiotoxins to heart cell membranes. Biochimt Biophysa Acta 732: 282–288. 10.1016/0005-2736(83)90213-4 6871194

[22] Konshina AG, Boldyrev IA, Utkin YN, Omel’kov AV, Efremov RG. (2011) Snake cytotoxins bind to membranes via interactions with phosphatidylserine head groups of lipids. Plos One 6: 1–13. 10.1371/journal.pone.0019064 PMC3084733 21559494

[23] World Health Organization. Guidelines for the prevention and clinical management of snakebite in África. Brazaville: World Health Organization Regional Office for Africa; 2010.

[24] Mendez I, Gutiérrez JM, Ângulo Y, Calvete JJ, Lomonte B. (2011) Comparative study of the cytolytic activity of snake venoms African spitting cobras (*Naja* spp., Elapidae) and its neutralization by a polyspecific antivenom. Toxicon 58: 558–564. 10.1016/j.toxicon.2011.08.018 21924279

[25] Berling I, Brown SGA, Miteff F, Levi C, Isbister GK. (2015) Intracranial haemorrhages associated with Venom induced consumption coagulophaty in Australian snakebites. Toxicon 102: 8–13. 10.1016/j.toxicon.2015.05.012 26003794

[26] Tambourgi DV, Santos MC, Furtado MFD, Freitas MCW, Silva WD, Kipnis TL. (1994) Pro-inflammatory activities in elapid snake venoms. Br J Pharmacol 112: 723–727. 7921595 10.1111/j.1476-5381.1994.tb13137.x PMC1910224

[27] Cher CDN, Armugam A, Lachumanan R, Coghlan MW, Jeyaseelan K. (2003) Pulmonary inflammation and edema induced by Phospolipase A2: Global gene analysis on aquaporins and Na+/K+-ATPase. J Biol Chem 278: 31352–31360. 10.1074/jbc.M302446200 12746451

[28] Dong VL, Quyen LK, Eng KH, Gopalakrishnakone P. (2003) Immunogenicity of venoms from four common snakes in the South of Vietnam and development of ELISA kit for venom detection. J Immunol Methods 282: 13–31. 14604537 10.1016/s0022-1759(03)00277-1

[29] Koen TL, Williams D. (2011) Snake antivenoms in southern Africa. C M E 29: 75–79.

[30] Broadley DG, Wüster W. (2004) A review of the southern African “*non spitting*” cobras (Serpentes: Elapidae: *Naja*). African J Herpet 53: 101–122.

[31] Clinical Toxinology Resource. Snakes Search Page; 2018 [accessed Oct 18 2018]. Available from: http://www.toxinology.com/fusebox.cfm?fuseaction=main.snakes.display&id=SN0231

[32] Eangelbrecht A. (2012) Management of common animal bites in the emergency centre: The most common animal bites to present in emergencies are dog and snake bites. Continuing Medical Education 30: 401–406.

[33] Westrin L, Von Rahmel P. (2011) The Snouted Cobra, *Naja annulifera*, Peters 1854. Berus 6: 1–22.

[34] Lobetti RG, Joubert K. (2004) Retrospective study of snake envenomation in 155 dogs from the onderstepoort area of South Africa. J S Afr Vet Assoc 75: 169–172. 15830600 10.4102/jsava.v75i4.477

[35] Langhorn R, Persson F, Ablad B, Goddard A, Schoeman JP, Willesen JL, Tarnow I, Kejelgaard-Hansen M. (2014) Myocardial injury in dogs with snake envenomation and its relation to systemic inflammation. J Vet Emerg Crit Care (San Antonio) 24: 1006–1010. 10.1111/vec.12127 24304906

[36] Nagel SS, Schoeman JP, Thompson P N, Wiinberg B, Goddard A. (2014) Hemostatic analysis of dogs naturally envenomed by the African Puffader (*Bitis arietans*) and Snouted Cobra (*Naja annulifera*). J Vet Emerg Crit Care (San Antonio) 24: 662–671. 25351524 10.1111/vec.12236

[37] Weise KHK, Carlsson FHH, Joubert FJ, Strydom DJ. (1973) The purification of toxins V^II^1 and V^II^2, two cytotoxin homologues from banded egyptian cobra (*Naja haje annulifera*) venom, and the complete amino acid sequence of toxin V^II^1. Hoppe Seylers Z Physiol Chem 354:1317–1326. 4803830 10.1515/bchm2.1973.354.2.1317

[38] Joubert FJ. (1975) The amino acid sequences of three toxins (CM-10, CM-12 and CM-14) from *Naja haje annulifera* (Egyptian Cobra) venom. Hoppe Seylers Z Physiol Chem 356:0 53–72. 1975. 1213685

[39] Joubert FJ. (1975) The amino acid sequences of toxin V^II^2, a cytotoxin homologue from banded egyptian cobra (*Naja haje annulifera*) venom. Hoppe Seylers Z Physiol Chem 356: 1893–1900. 1213684 10.1515/bchm2.1975.356.2.1893

[40] Joubert FJ. (1975) The purification and amino acid sequence of toxin CM-13b from *Naja haje annulifera* (Egyptian Cobra) venom. Hoppe Seylers Z Physiol Chem 356: 1901–1908. 1213685

[41] Joubert FJ. (1976) The amino acid sequences of three toxins (CM-8, CM-11 and CM-13a) from *Naja haje annulifera* (Egyptian Cobra) venom. Eur J Biochem 64: 219–232. 1278155 10.1111/j.1432-1033.1976.tb10291.x

[42] Heus F, Vonk F, Otvos RA, Bruyneel B, Smit AB, et al (2013) An efficient analytical platform for on-line microfluidic profiling of neuroactive snake venoms towards nicotinic receptor affinity. Toxicon 61: 112–124. 10.1016/j.toxicon.2012.11.002 23159399

[43] Laemmli UK. (1970) Cleavage of structural proteins during the assembly of the head of bacteriophage T4. Nature 15: 680–685. 10.1038/227680a0 5432063

[44] Morrissey JH. (1980) Silver stain for proteins in polyacrilamide gels: a modified procedure with enhanced uniform sensitivity. Anal Bioch 117: 307–310. 10.1016/0003-2697(81)90783-1 6172996

[45] Towbin H, Staehelin T, Gordon J. (1979) Eletrophoretic transfer of proteins from polyacrilamide gels to nitrocellulose sheets: procedure and some applications. PNAS 76: 4350–4354. 388439 10.1073/pnas.76.9.4350 PMC411572

[46] Paixão-Cavalcante D, Kuniyoshi AK, Portaro FC, Da Silva WD, Tambourgi DV. (2015) African adders: partial characterization of snake venoms from three *Bitis* species of medical importance and their neutralization by experimental equine antivenoms. Plos Negl Trop Dis 9: 1–18. 10.1371/journal.pntd.0003419 PMC4340965 25643358

[47] Kinter M, Sherman NE. Protein sequencing and identification using tandem mass spectrometry. John Wiley & Sons, Inc 2000: 161–163.

[48] Rappsilber J, Mann M, Ishihama Y. (2007) Protocol for micro-purification, enrichment, pre-fractionation and storage of peptides for proteomics using StageTips. Nat Protoc. 2(8):1896–906. 10.1038/nprot.2007.261 17703201

[49] Cox J. and Mann M. (2008) MaxQuant enables high peptide identification rates, individualized p.p.b.-range mass accuracies and proteome-wide protein quantification. Nature Biotechnology 26(12) 1367–1372. 10.1038/nbt.1511 19029910

[50] Vizcaíno J.; Deutsch E.; Wang R. (2014) ProteomeXchange provides globally coordinated proteomics data submission and dissemination. Nat. Biotechnol. 32, 223–226. 10.1038/nbt.2839 24727771 PMC3986813

[51] Heussen C, Dowdle EB. (1980) Electrophoretic analysis of plasminogen activators in polyacrylamide gels containing sodium dodecyl sulfate and copolymerized substrates. Anal Biochem 102: 196–202. 7188842 10.1016/0003-2697(80)90338-3

[52] Pukrittayakamee S, Warrell DA, Desakorn V, McMichael AJ, White NJ, Bunnag D. (1988) The hyaluronidase activities of some southeast asian snake venoms. Toxicon 26: 629–637. 3176052 10.1016/0041-0101(88)90245-0

[53] Mosmann T. (1980) Rapid colorimetric assay for cellular growth and survival: application to proliferation and cytotoxicity assays. J Immunol Methods 65: 55–63. 10.1016/0022-1759(83)90303-4 6606682

[54] Gulati G, Hevelow M, George M, Behling E, Siegel J. International normalized ratio versus plasma levels of coagulation factors in patients on vitamin L antagonist therapy. Arch Pathol Lab Med 135: 490–494. 10.1043/2009-0474-OA.1 21466367

[55] Ramos-Cerrillo B, De Roodt A, Chippaux JP, Olguín L, Casasola A, et al (2008) Characterization of a new polyvalent antivenom (Antivipmyn Africa) against African vipers e elapids. Toxicon 52: 881–888. 10.1016/j.toxicon.2008.09.002 18926842

[56] Finney PJ. Probit Analysis. 3^rd^ ed Cambridge: Cambridge University Press; 1971.

[57] Yamakawa M, Nozaki M, Hokama Z. (1976) Fractionation of sakishima habu (*Trimeresurus elegans*) venom and lethal hemorrhagic, and edema forming activities of fractions. In Animal plant and microbial toxins 48: 97–109.

[58] Galvão Nascimento N, Sampaio MC, Amaral Olivo R, Teixeira C. (2010) Contribution of mast cells to the oedema induced by *Bothrops moojeni* snake venom and pharmacological assessment of the inflammatory mediator involved. Toxicon 55: 343–352. 10.1016/j.toxicon.2009.08.009 19703484

[59] Olivo R, Teixeira CF, Wallace JL, Gutierrez JM, Zamuner SR. (2007) Role of cyclooxigenases in oedema-forming activity of bothropic venoms. Toxicon 49: 670–677. 10.1016/j.toxicon.2006.11.006 17204299

[60] Wanderley CW, Silva CM, Wong DV, Ximenes RM, Morelo DF, Coeker F, Aragão KS, Fernandes C, Palheta-Júnior RC, Havt A, Brito GA, Cunha FQ, Ribeiro RA, Lima-Júnior. (2014) *Bothrops jararacussu* snake venom-induces a local inflammatory response in a prostanoid- and neutrophil-dependent manner. Toxicon 90: 134–147. 10.1016/j.toxicon.2014.08.001 25127849

[61] Zoccal KF, Sorgi CA, Hori JI, Paula-Silva FW, Arantes EC, Serezani CH, Zamboni DS, Faccioli LH. (2016) Opposing roles of LTB4 and PGE2 in regulating the inflammasome-dependent scorpion venom-induced mortality. Nat Commun 23: 1–13. 10.1038/ncomms10760 PMC4766425 26907476

[62] Cirino G, Peers SH, Wallace JL, Flower RJ. (1989) A study of phospholipase A_2_-induced oedema in rat paw. Eur J Pharmacol 166: 505–510. 2806373 10.1016/0014-2999(89)90364-6

[63] Koga MM, Bizzarro B, Sá-Nunes A, Rios JF, Jancar S. (2016) Boosting adaptive immunity: A new role for PAFR antagonists. Sci Rep 14: 1–9. 10.1038/srep39146 PMC5155422 27966635

[64] Goldstein IJ. (1975) Studies on the combining sites of concanavalin A. Adv Exp Med Biol 55: 35–53. 1155247 10.1007/978-1-4684-0949-9_3

[65] Kubota Y, Fujiota K, Takekawa M. (2017) WGA-based lectin affinity gel electrophoresis: A novel method for the detection of O-GlcNAc-modified proteins. PLoS One 7: 1–12 10.1371/journal.pone.0180714 PMC5501588 28686627

[66] Panagides N, Jackson TN, Ikonomopoulou MP, Arbuckle K, Pretzler R, yang DC, Ali SA, Koludarov I, Dobson J, Sanker B, Asselin A, Santana RC, Hendrikx I, Van der Ploeg H, Tai-A-Pin J, Van den Bergh R, Kerkkamp HM, Vonk FJ, Naude A, Strydom MA, Jacobsz L, Dunstan N, Jaeger M, Hodgson WC, Miles J, Fry BG. (2017) How the cobra got its flesh-eating venom: Cytotoxicity as a defensive innovation and its co-evolutaion with hooding, aposematic marking, and spitting. Toxins (Basel) 13: 1–22. 10.3390/toxins9030103 PMC5371858 28335411

[67] Ebrahim K, Shirazi FH, Mirakabadi AZ, Vatanpour H. (2015) Cobra venom cytotoxins: apoptotic or necrotic agents. Toxicon 108: 108–134. 10.1016/j.toxicon.2015.10.004 26482932

[68] Andrade-Silva D, Zelanis A, Kitano ES, Junqueira-de-Azevedo IL, Reis MS, Lopes AS, Serrano SM. Proteomic and glycoproteomic profilings reveal that post-translational modifications of toxins contribute to venom phenotype in snakes. J Proteome Res. 2016 15(8):2658–75. 10.1021/acs.jproteome.6b00217 27297130

[69] Zelanis A, Serrano SM, Reinhold VN. (2012) N-glycome profiling of *Bothrops jararaca* newborn and adults venoms. J Proteome Res 75: 774–782. 10.1016/j.jprot.2011.09.017 21989267

[70] Zelanis A, Tashima AK, Rocha MM, Furtado MF, Camargo AC, Serrano SM. (2010) Analysis of the ontogenetic variation in the venom proteome/peptidome of *Bothrops jararaca* reveals different strategies to deal with prey. J Proteome Res 9: 2278–2291. 10.1021/pr901027r 20146532

[71] Fujita T. (2002) Evolution of the lectin-complement pathway and its role in innate immunity. Nat Rev Immunol 2:346–353. 10.1038/nri800 12033740

[72] Ricklin D, Reis ES, Lambris JD. (2016) Complement in disease: a defence system turning offensive. Nat Rev Nephrol 12: 383–401. 10.1038/nrneph.2016.70 27211870 PMC4974115

[73] Kang TS, Georgieva D, Genov N, Murakami MT, Sinha M, Kumar RP, et al (2011) Enzymatic toxins from snake venom: structural characterization and mechanisms of catalysis. FEBS J 278:4544–4576. 10.1111/j.1742-4658.2011.08115.x 21470368

[74] Tan NH, Tan CS. (1988) A comparative study of cobra (*Naja*) venom enzymes. Comp Biochem Physiol B 90: 745–750. 2854766 10.1016/0305-0491(88)90329-x

[75] Tan NH, Ponnudurai G. (1992) The biological properties of venoms of some American coral snakes (Genus *Micrurus*). Comp Biochem Physiol B 101: 471–474. 1582185 10.1016/0305-0491(92)90029-q

[76] Phillips MA, Waterman JM, Du Plessis P, Smit M, Bennett NC. (2012) No evidence for proteolytic venom resistance in southern African ground squirrels. Toxicon 60: 760–763. 10.1016/j.toxicon.2012.06.004 22728461

[77] Kemparaju K, Girish KS. (2006) Snake venom hyaluronidase: a therapeutic target. Cell Biochem Funct 24: 7–12. 10.1002/cbf.1261 16245359

[78] Bitencourt CD, Gelfuso GM, Pereira PAT, De Assis PA, Tefé-Silva C, Ramos SG et al (2015) Hyaluronidase-Loaded PLGA Microparticles as a new strategy for the treatment of pulmonary fibrosis. Tissue Eng Part A 21: 246–256. 10.1089/ten.TEA.2013.0403 25037276

[79] Jiang D, Liang J, Fan J, Yu S, Chen S, Luo Y et al (2005) Regulation of lung injury and repair by Toll-like receptors and hyaluronan. Nat Med 11: 1173–1779. 10.1038/nm1315 16244651

[80] Burke JE, Dennis EA. (2009) Phospholipase A_2_ structure/function, mechanisms, and signaling. J Lipid Res 50: 237–242. 10.1194/jlr.R800033-JLR200 PMC2674709 19011112

[81] Pirolla RAS, Baldasso PA, Marangoni S, Moran PJ, Rodrigues JAR. (2011) Evaluation of snake Venom Phospholipase A_2_: hydrolysis of non-natural esters. J Braz Chem Soc 22: 300–307.

[82] Borges RJ, Lemke N, Fontes MRM. (2017) PLA2-like proteins myotoxic mechanism: a dynamic model description. Sci Rep. Nov 14; 7(1):15514. 10.1038/s41598-017-15614-z PMC5686144 29138410

[83] Leiguez E, Giannotti KC, Moreira V, Matsubara MH, Gutiérrez JM, Lomonte B, Rodríguez JP, Balsinde J, Teixeira C. (2014) Critical role of TLR2 and MyD88 for functional response of macrophages to a group IIA-secreted phospholipase A2 from snake venom. PLoS One 9: 1–11. 10.1371/journal.pone.0093741 PMC3981733 24718259

[84] Wong KY, Tan CH, Tan KY, Quraishi NH, Tan NH. (2018) Elucidating the biogeographical variation of the venom of *Naja naja* (spectacled cobra) from Pakistan through a venom-decomplexing proteomic study. J Proteomics 175: 153–173. 10.1016/j.jprot.2017.12.012 29278784

[85] Tan KY, Tan CH, Fung SY, Tan NH. (2015) Venomics, lethality and neutralization of *Naja kaouthia* (monocloned cobra) venoms from three different geographical regions of Southeast Asia. 10.1016/j.jprot.2015.02.012 25748141

[86] Lauridsen LP, Laustsen AH, Lomonte B, Gutiérrez JM. (2016) Exploring the venom of the forest cobra: Toxicovenomics and antivenom profiling of *Naja melanoleuca*. J Proteomics 150: 98–107. 10.1016/j.jprot.2016.08.024 27593527

[87] Malih I, Rusmili MRA, Tee TY, Saile R, Ghalim N, Othman I. (2014) Proteomic analysis of Moroccan cobra *Naja haje legionis* venom using tandem mass spectrometry. J Proteomics 96: 2040–251. 10.1016/j.jprot.2013.11.012 24269350

[88] Simões de Oliveira PR, França FS, Villas Boas IM, Teixeira da Rocha MM, Sant’Anna SS, Bastos ML, Tambourgi DV. (2018) Snake venoms from Angola: Intra-specific variations and immunogenicity. Toxicon 148: 85–94. 10.1016/j.toxicon.2018.04.013 29673703

[89] Stefansson S, Kini RM, Evans HJ. (1989) The inhibition of clotting complexes of the extrinsic cascade by the phospholipase A_2_ isoenzymes from *Naja nigricollis* venom. Thromb Res 15: 481–491. J Proteomics 120: 105–173. 10.1016/0049-3848(89)90056-x 2814939

[90] Stefansson S, Kini RM, Evans HJ. (1990) The basic phospholipase A_2_ from *Naja nigricollis* venom inhibits the prothrombinase complex by a novel nonenzymatic mechanism. Biochemistry 21: 7742–7746. 10.1021/bi00485a024 2271532

[91] Kini RM, Evans HJ. (1995) The role of enzymatic activity in inhibition of the extrinsic tenase complex by phospholipase A_2_ isoenzymes from *Naja nigricollis* venom. Toxicon 33: 1585–1590. 8866616 10.1016/0041-0101(95)00103-4

[92] Kerns RT, Kini RM, Stefansson S, Evans HJ. (1999) Targeting of venom phospholipase: the strongly anticoagulant Phospholipase A_2_ from *Naja nigricollis* venom binds to coagulation factor Xa to inhibit the protrombinase complex. Arch Biochem Biophys 369: 107–113. 10.1006/abbi.1999.1345 10462445

[93] MacKay N, Ferguson JC, McNicol GP. (1969) Effects three cobra venoms on blood coagulation, platelet aggregation, and fibrinolysis. J clin Path 22: 304–311. 5814735 10.1136/jcp.22.3.304 PMC474066

[94] White J. International symposium on coral snakes: Elapidae snakebite in Africa and Asia. 2016 [accessed Oct 18, 2018]. Available from: http://sites.pucgoias.edu.br/eventos/isc/wp-content/uploads/sites/34/2016/10/anais_miolo-1.pdf

[95] Ali SA, Alam JM, Abbasi A, Zaidi ZH, Stoeva S, Voelter W. (2000) Sea snake *Hydrophis cyanocinctus* venom. II. Histopathological changes, induced by a myotoxic phospholipase A_2_ (PLA_2_-H1). Toxicon 38: 687–705. 10673160 10.1016/s0041-0101(99)00184-1

[96] Ownby CL, Fletcher JE, Colberg TR. (1993) Cardiotoxin 1 from cobra (*Naja naja atra*) venom causes necrosis of skeletal muscle in vivo. Toxicon 31: 697–709. 8342169 10.1016/0041-0101(93)90376-t

[97] Hierholzer C, Kalff JC, Laurel O, Katsuhiko T, Loeffert JE, Watkins SC, Billiar TR, Tweardy DJ. (1998) Interleukin-6 production in hemorrhagic shock is accompanied by neutrophil recruitment and lung injury. Am J Physiol 275: 611–621. 10.1152/ajplung.1998.275.3.L611 9728057

[98] Frink M, Lu A, Thobe BM, Hsieh YC, Choudhry, Shwacha MG, Kunkel SL, Chaudry IH. (2006) Monocyte chemoattractant protein-1 influences trauma-hemorrhage-induced distal organ damage via regulation of keratinocyte-derived chemokine production. Am J Physiol Regul Integr Comp Physiol 292: 1110–1116. 10.1152/ajpregu.00650.2006 17095647

[99] Mommsen P, Barkhausen T, Frink M, Zeckey C, Probst C, Krettek C, Hildebrand F. (2011) Productive capacity of alveolar macrophages and pulmonar organ damage after femoral fracture and hemorrhage in IL-6 knockout mice. Cytokine 53: 60–65. 10.1016/j.cyto.2010.09.005 20934884

[100] Guidolin FR, Caricati CP, Marcelino JR, Da Silva WD. (2016) Development of equine IgG antivenoms against major snake groups in Mozambique. PLoS Negl Trop Dis 10: 1–17. 10.1371/journal.pntd.0004325 PMC4701360 26730709

[101] Sánchez A, Segura A, Vargas M, Herrera M, Villalta M, Estrada R, Wu F, Litschka-Koen T, Perry MA, Alape-Girón A, León G. (2017) Expanding the neutralization scope of the EchiTAb-plus-ICP antivenom to include venoms of elapids from Southern Africa. Toxicon 125: 59–64. 10.1016/j.toxicon.2016.11.259 27890775

