# Supplementary material for: Naja annulifera Snake: New insights into the venom components and pathogenesis of envenomation
Source: PLoS Negl Trop Dis. 2019 Jan 18;13(1):e0007017. doi: 10.1371/journal.pntd.0007017 (PMC6338361; doi:10.1371/journal.pntd.0007017)
Supplement: S1 Spectra — (DOCX) [file pntd.0007017.s003.docx]

**Supplementary Information.** Annotated MS/MS spectra of unique peptides identified in *Naja annulifera* venom proteins (listed in Supplementary Table S2).**Protein ID – B2BRS4**

**Protein name:** Truncated putative phospholipase A2 OS=Austrelaps labialis OX=471292 PE=2 SV=1

**Number of Unique Peptides:** 1

**m/z:** 819.7

**MS/MS ID:**320

**Score:** 164.5

**Spectrum:** 1/1


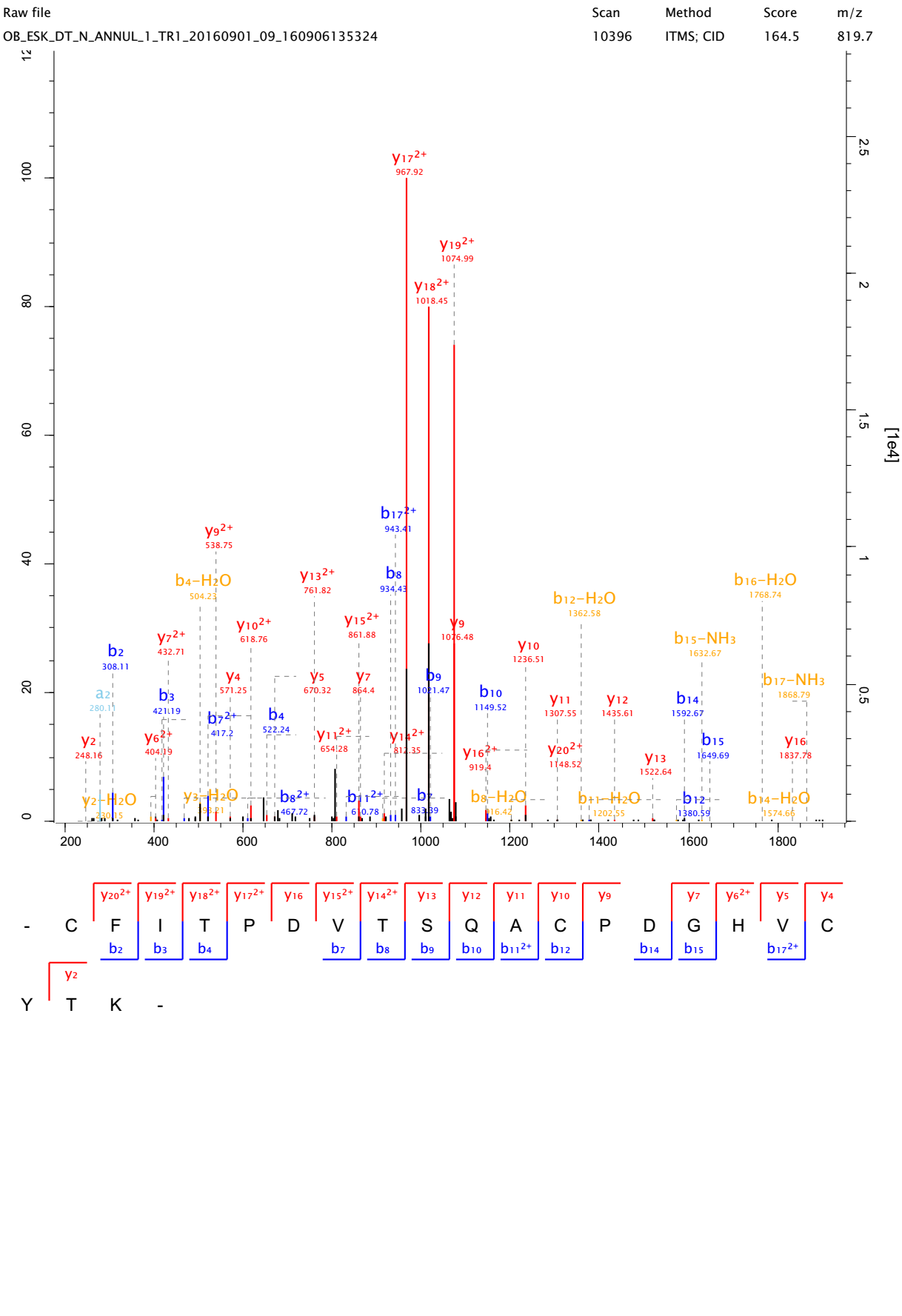


**Protein ID – P84808**

**Protein name:** Cysteine-rich venom protein kaouthin-2 OS=Naja kaouthia OX=8649 PE=1 SV=2

**Number of Unique Peptides:** 4

**m/z:** 516.2

**MS/MS ID:** 310

**Score:** 166.68

**Spectrum:** 1/4


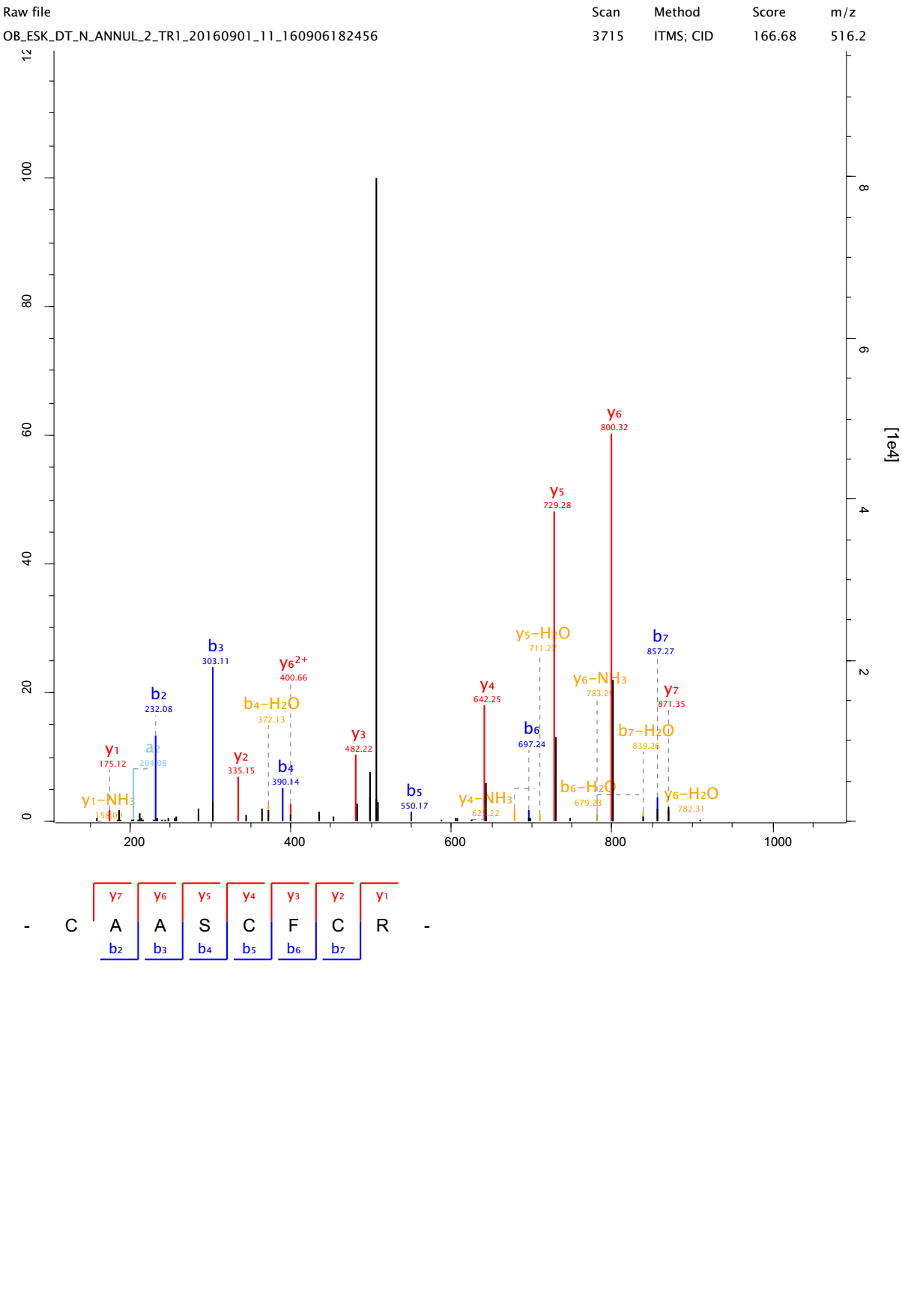


**Protein ID – P84808**

**Protein name:** Cysteine-rich venom protein kaouthin-2 OS=Naja kaouthia OX=8649 PE=1 SV=2

**Number of Unique Peptides:** 4

**m/z:** 513.94

**MS/MS ID:** 1763

**Score:** 178.95

**Spectrum:** 2/4


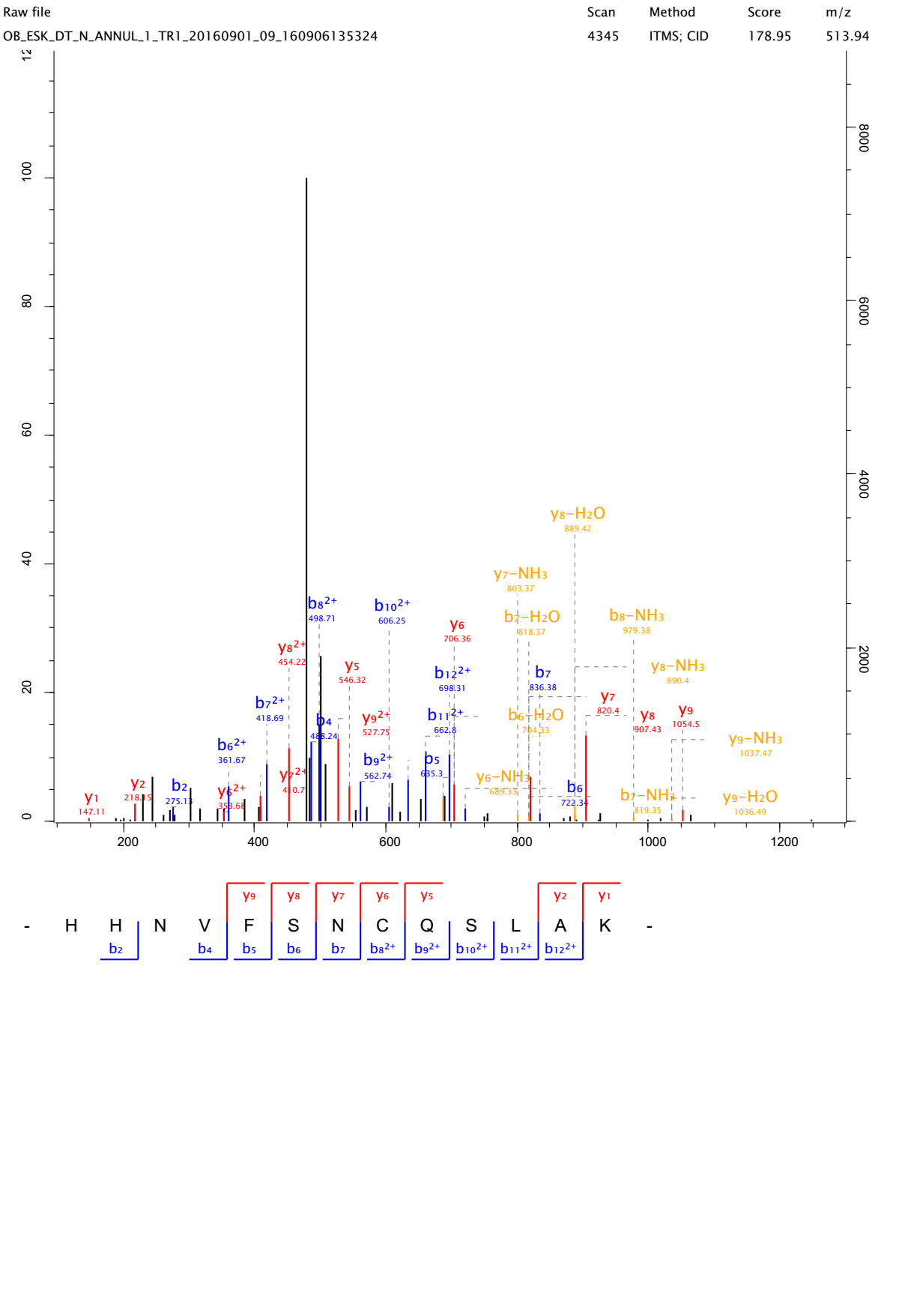


**Protein ID – P84808**

**Protein name:** Cysteine-rich venom protein kaouthin-2 OS=Naja kaouthia OX=8649 PE=1 SV=2

**Number of Unique Peptides:** 4

**m/z:** 623.76

**MS/MS ID:** 3081

**Score:** 169.21

**Spectrum:** 3/4


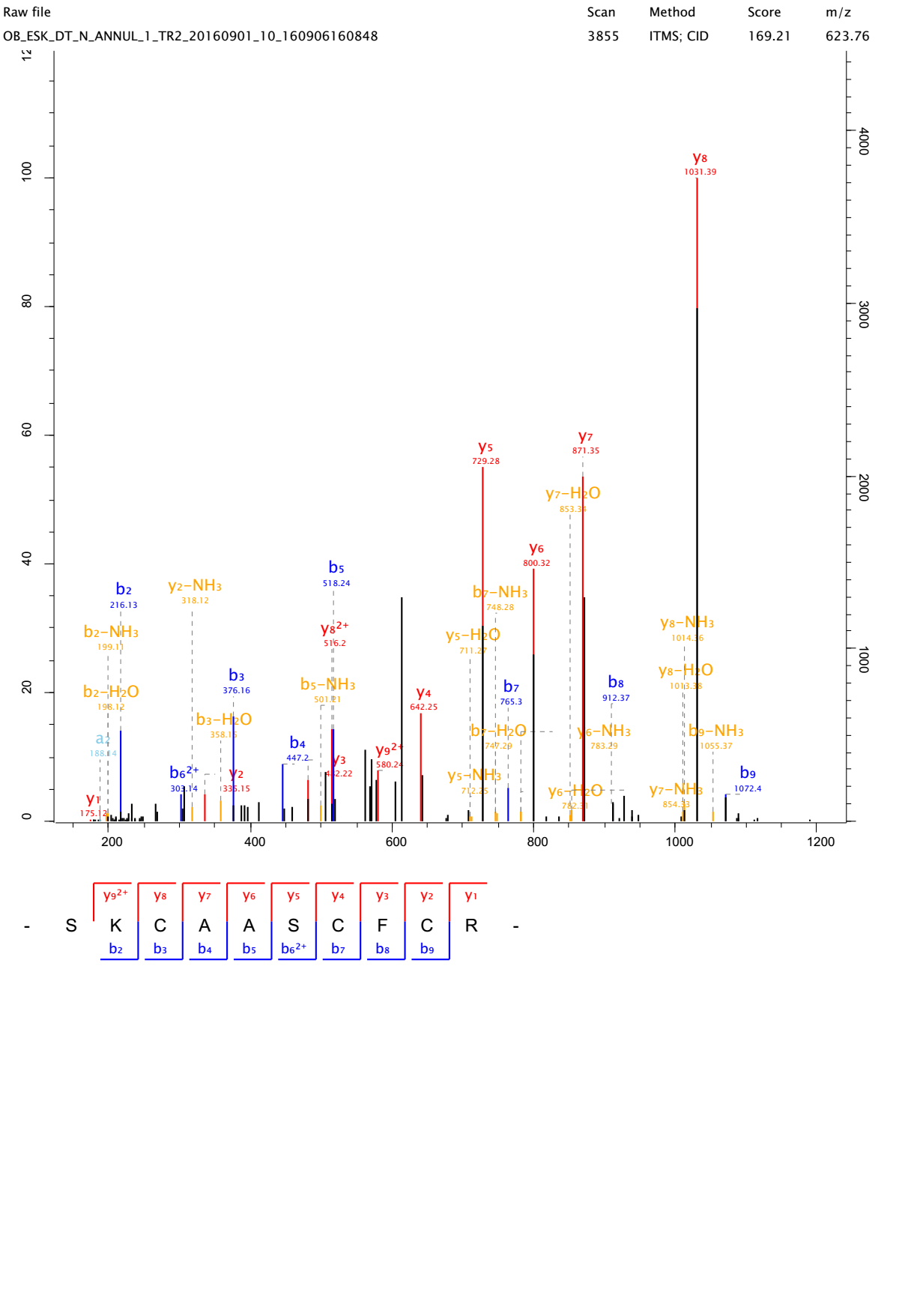


**Protein ID – P84808**

**Protein name:** Cysteine-rich venom protein kaouthin-2 OS=Naja kaouthia OX=8649 PE=1 SV=2

**Number of Unique Peptides:** 4

**m/z:** 1276.62

**MS/MS ID:** 3957

**Score:** 178.56

**Spectrum:** 4/4


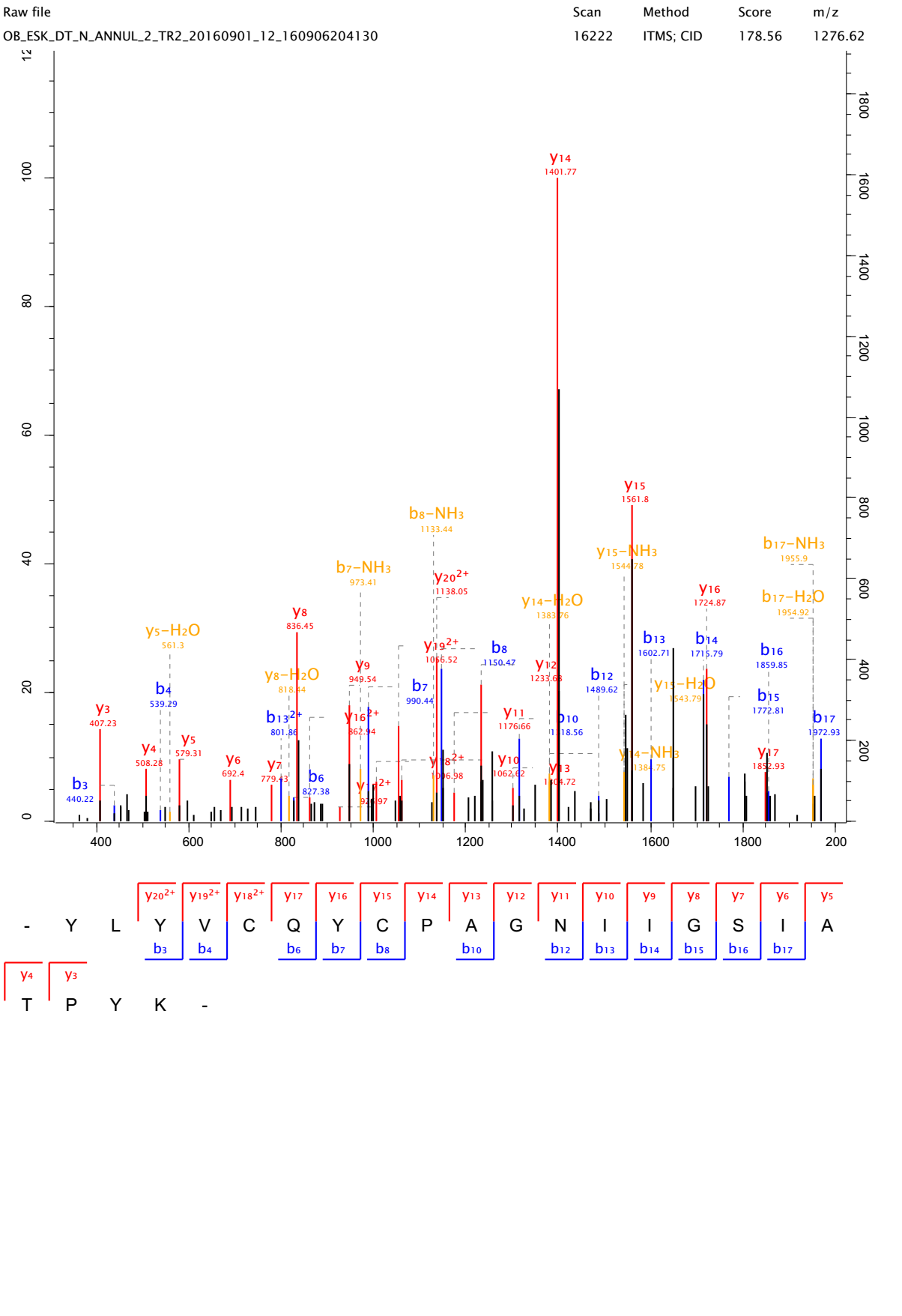


**Protein ID – A0A098LY86**

**Protein name:** Carboxylic ester hydrolase (Fragment) OS=Python regius OX=51751 PE=2 SV=1

**Number of Unique Peptides:** 1

**m/z:** 765.41

**MS/MS ID:** 204

**Score:** 62.59

**Spectrum:** 1/1


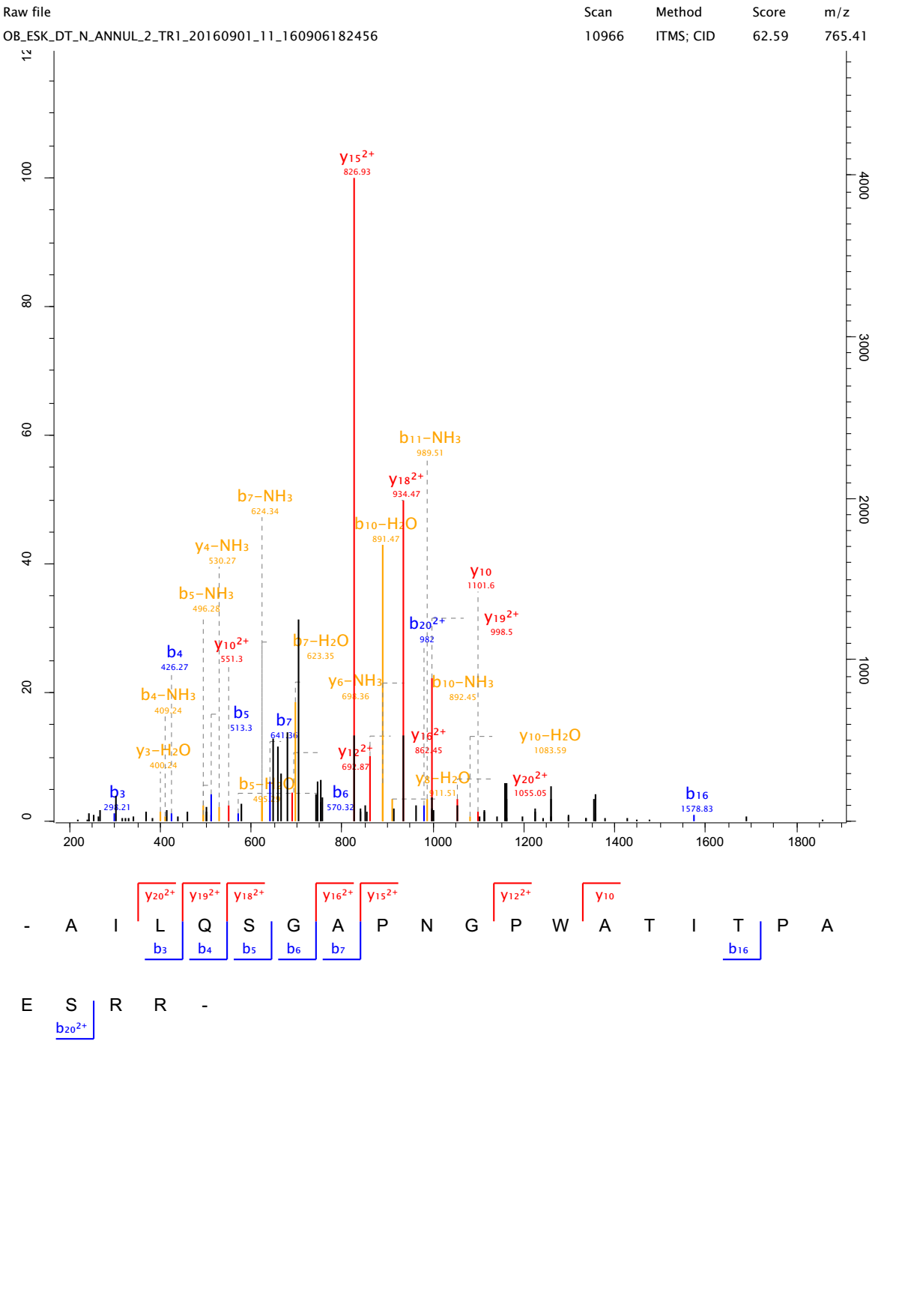


**Protein ID – A0A098LYB5**

**Protein name:** Carboxylic ester hydrolase OS=Opheodrys aestivus OX=186591 PE=2 SV=1

**Number of Unique Peptides:** 3

**m/z:** 1069.56

**MS/MS ID:** 200

**Score:** 208.89

**Spectrum:** 1/3


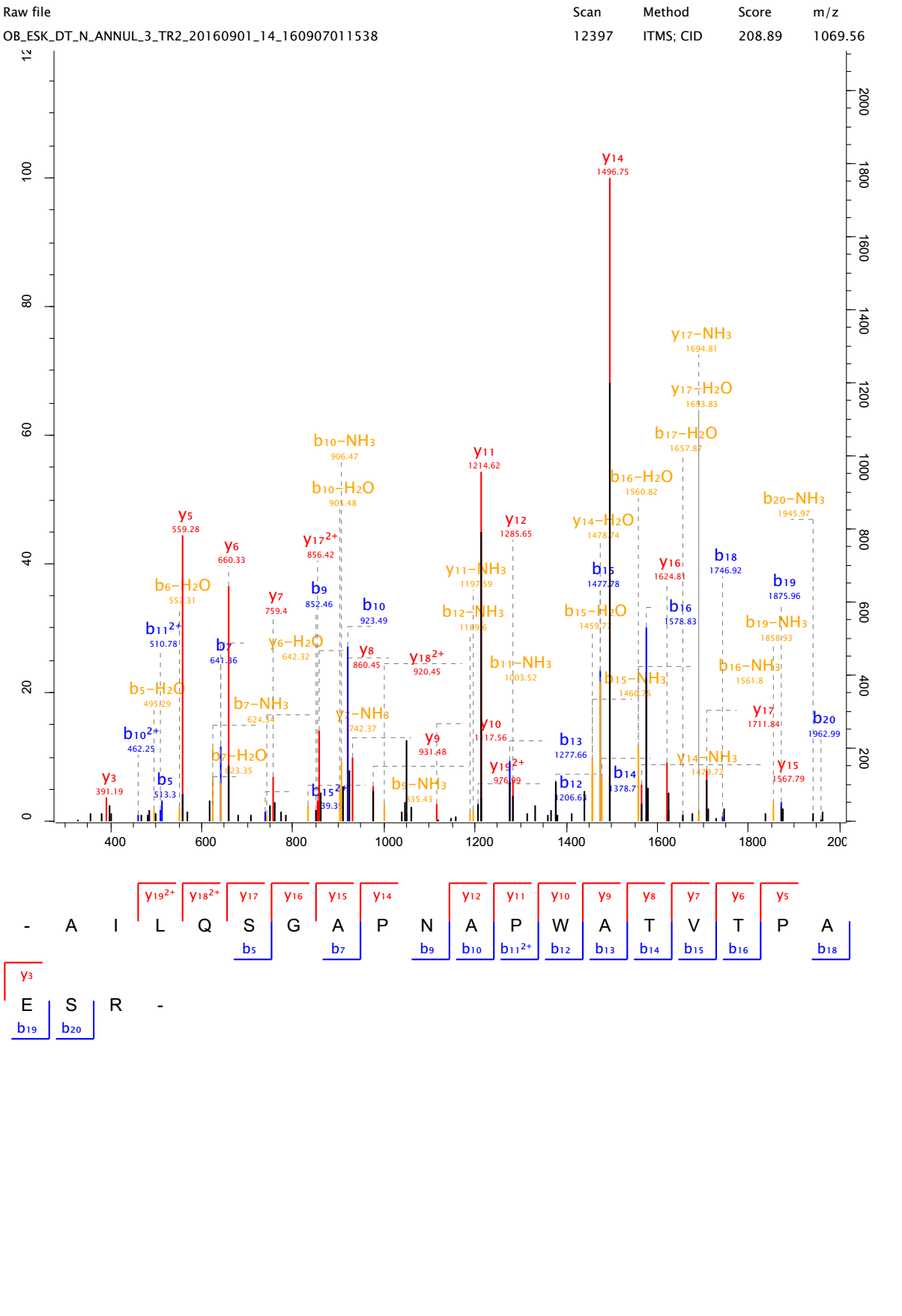


**Protein ID – A0A098LYB5**

**Protein name:** Carboxylic ester hydrolase OS=Opheodrys aestivus OX=186591 PE=2 SV=1

**Number of Unique Peptides:** 3

**m/z:** 765.41

**MS/MS ID:** 203

**Score:** 88.57


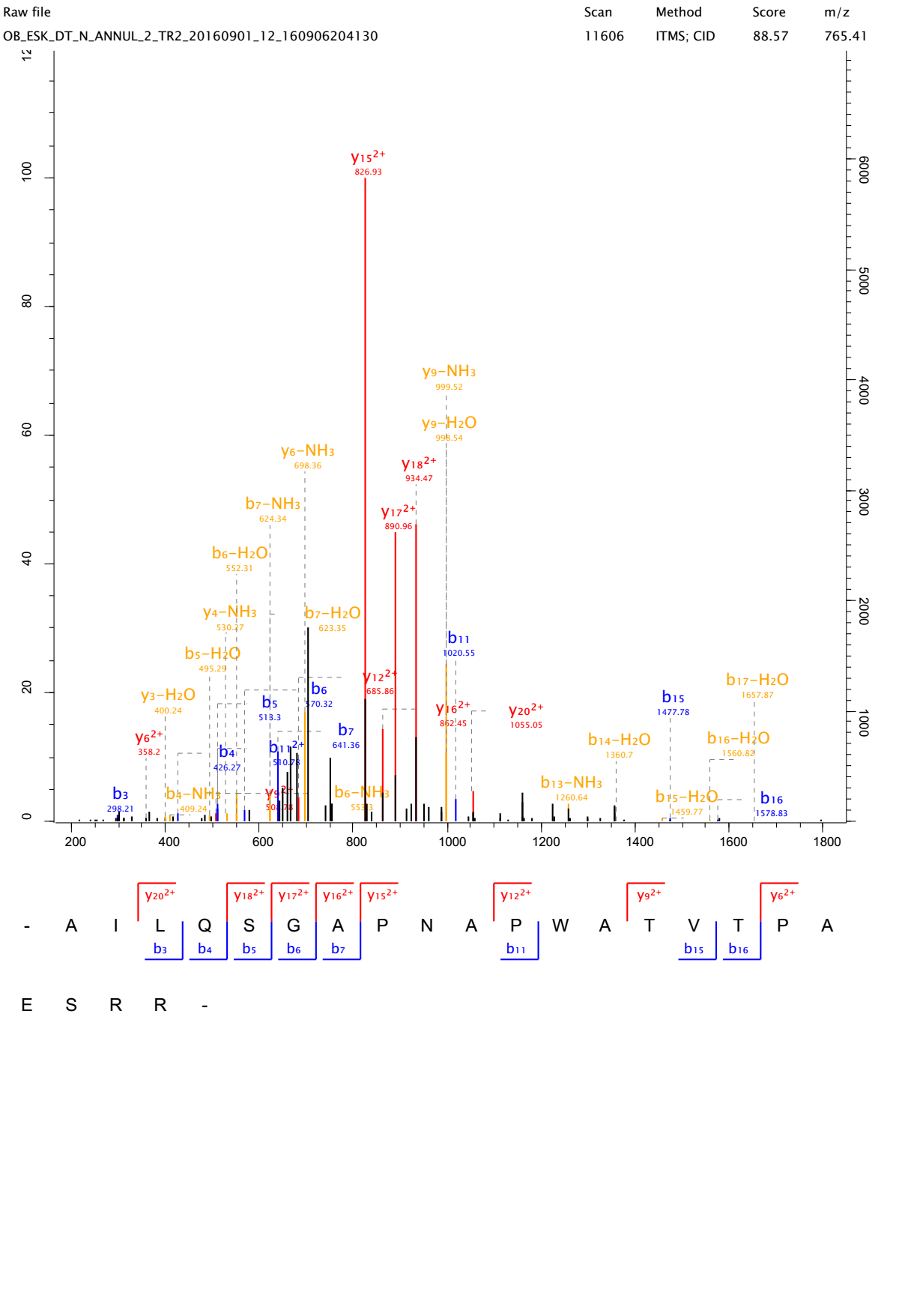
**Spectrum:** 2/3

**Protein ID – A0A098LYB5**

**Protein name:** Carboxylic ester hydrolase OS=Opheodrys aestivus OX=186591 PE=2 SV=1

**Number of Unique Peptides:** 3

**m/z:** 797.74

**MS/MS ID:** 298

**Score:** 70.84

**Spectrum:** 3/3


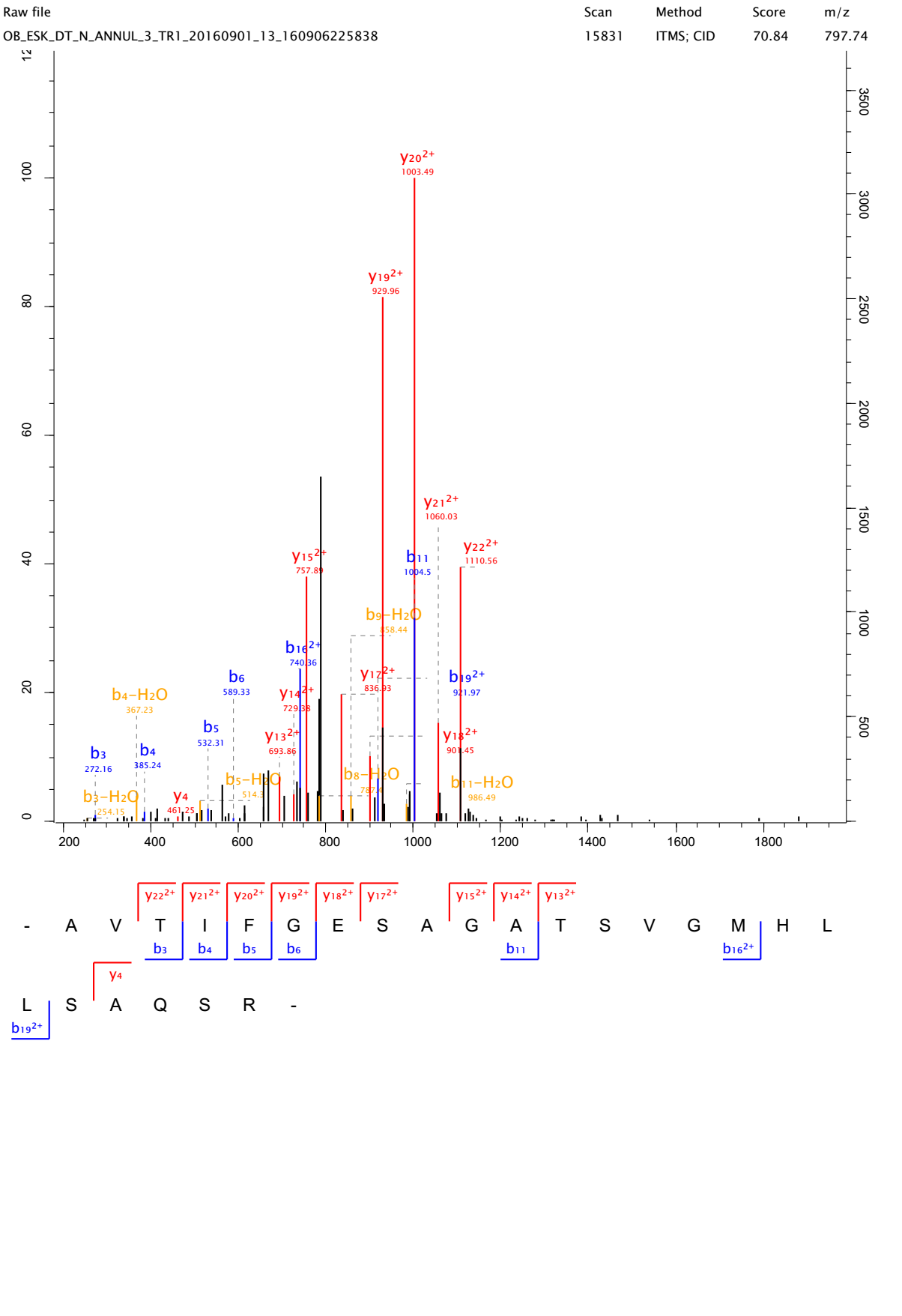


**Protein ID – A0A0F7YYZ8**

**Protein name:** Phosphodiesterase OS=Micrurus fulvius OX=8637 PE=2 SV=1

**Number of Unique Peptides:** 4

**m/z:** 941.06

**MS/MS ID:** 533

**Score:** 181.6


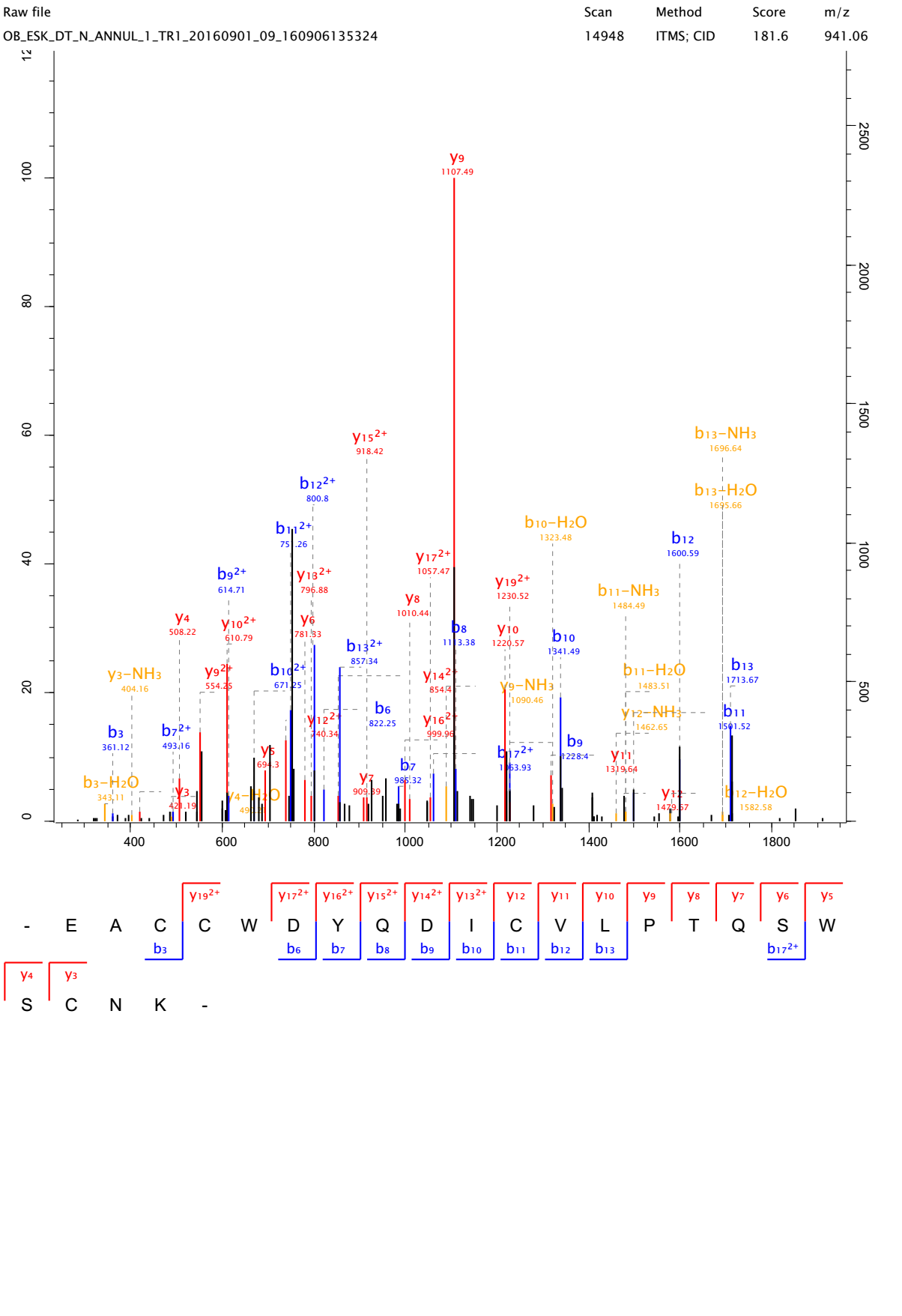
**Spectrum:** 1/4

**Protein ID – A0A0F7YYZ8**

**Protein name:** Phosphodiesterase OS=Micrurus fulvius OX=8637 PE=2 SV=1

**Number of Unique Peptides:** 4

**m/z:** 1031.12

**MS/MS ID:** 550

**Score:** 132.62

**Spectrum:** 2/4


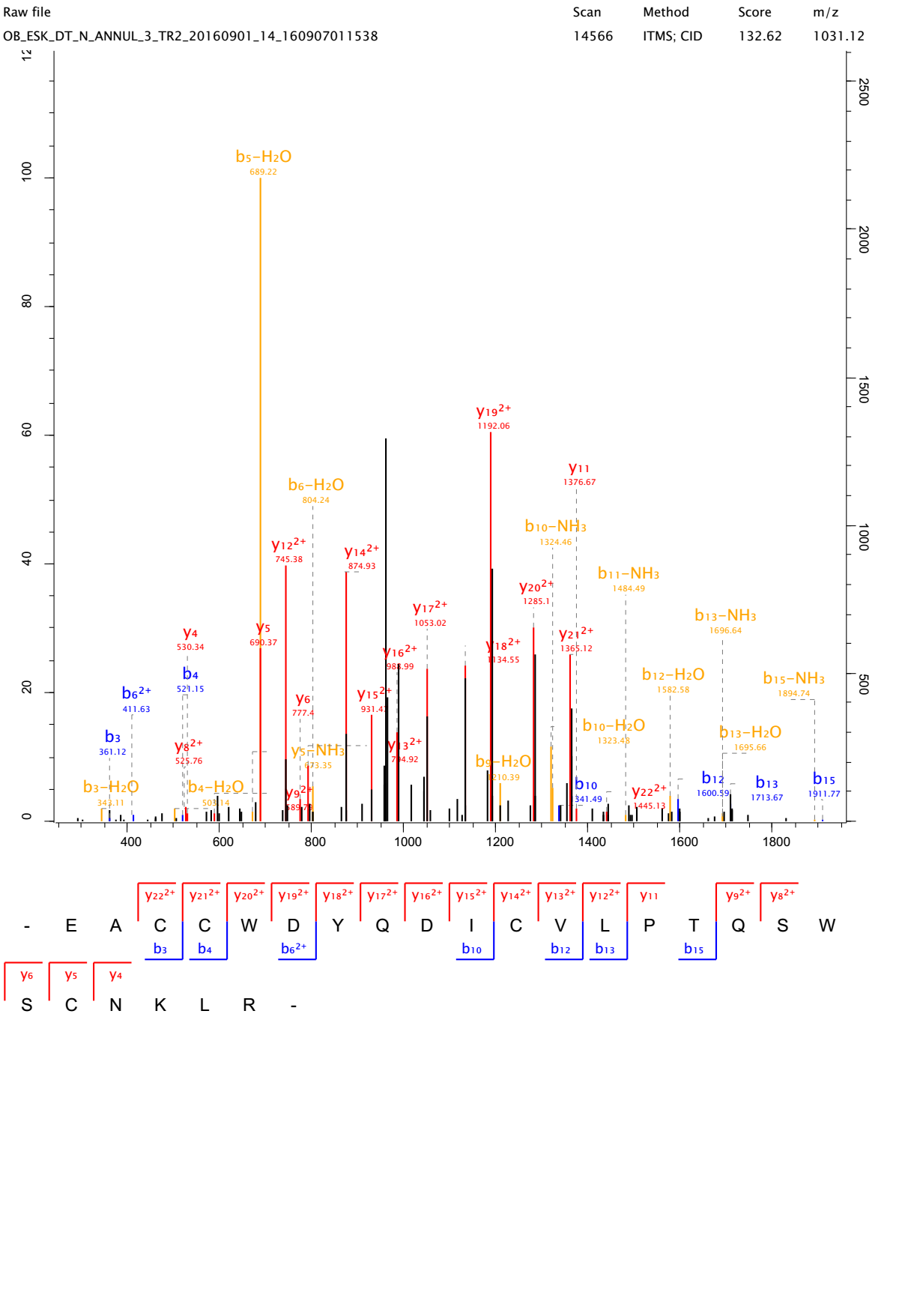


**Protein ID – A0A0F7YYZ8**

**Protein name:** Phosphodiesterase OS=Micrurus fulvius OX=8637 PE=2 SV=1

**Number of Unique Peptides:** 4

**m/z:** 746.43

**MS/MS ID:** 2369

**Score:** 152.11

**Spectrum:** 3/4


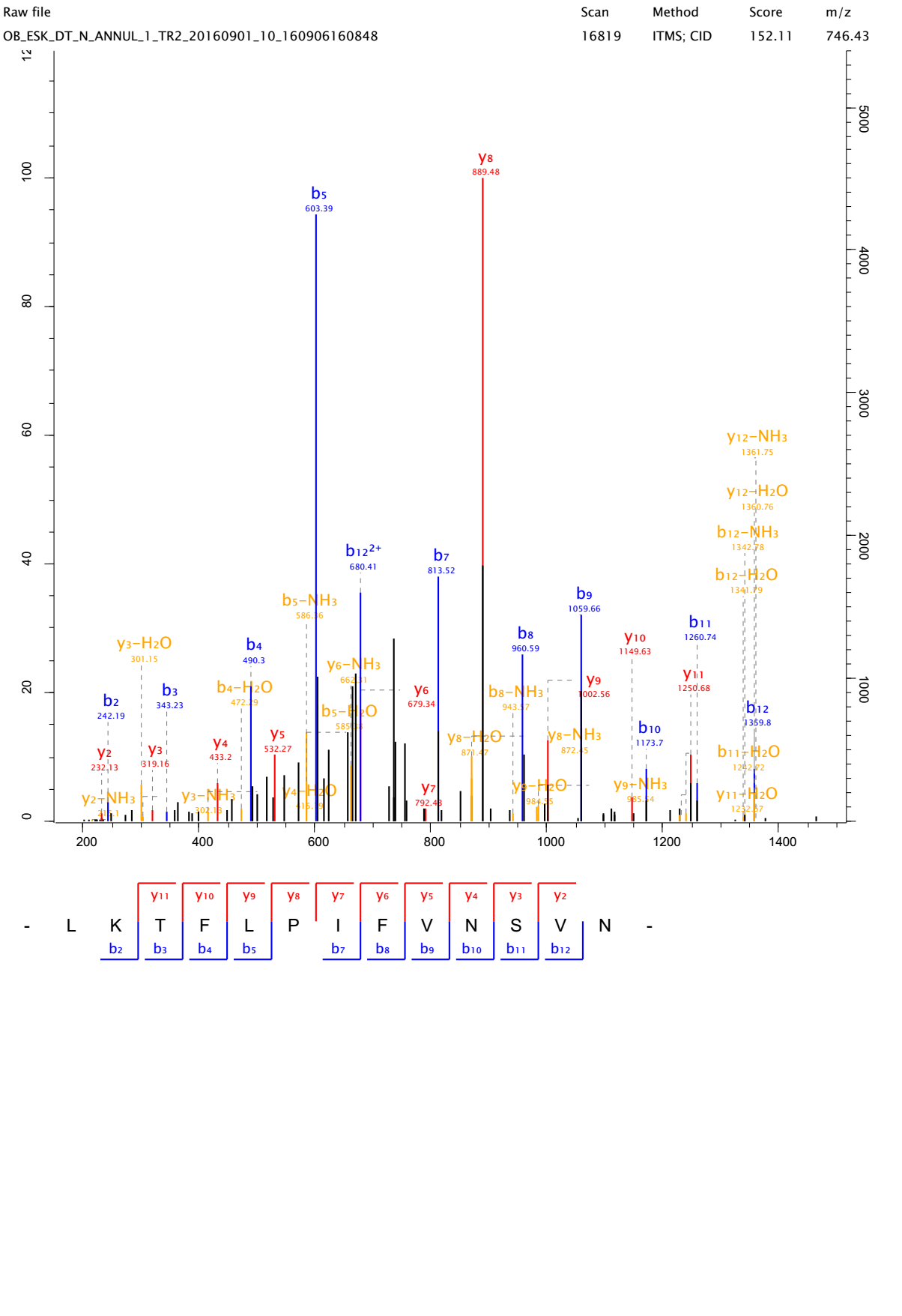


**Protein ID – A0A0F7YYZ8**

**Protein name:** Phosphodiesterase OS=Micrurus fulvius OX=8637 PE=2 SV=1

**Number of Unique Peptides:** 4

**m/z:** 625.84

**MS/MS ID:** 3473

**Score:** 144.1

**Spectrum:** 4/4


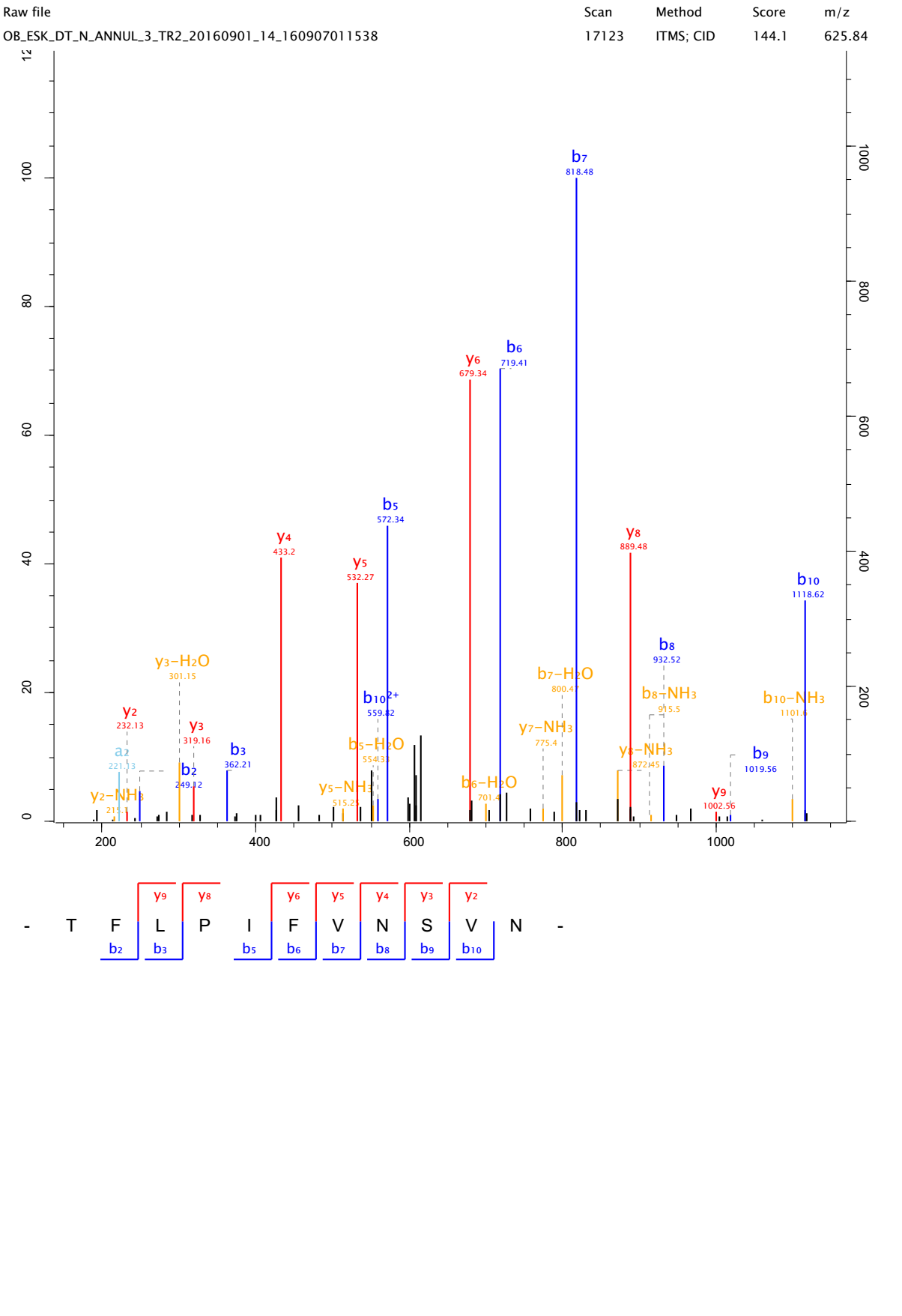


**Protein ID – U3FYP9**

**Protein name:** Ecto-5-nucleotidase 1c OS=Micrurus fulvius OX=8637 PE=2 SV=1

**Number of Unique Peptides:** 1

**m/z:** 598.01

**MS/MS ID:** 1803

**Score:** 123.29

**Spectrum:** 1/1


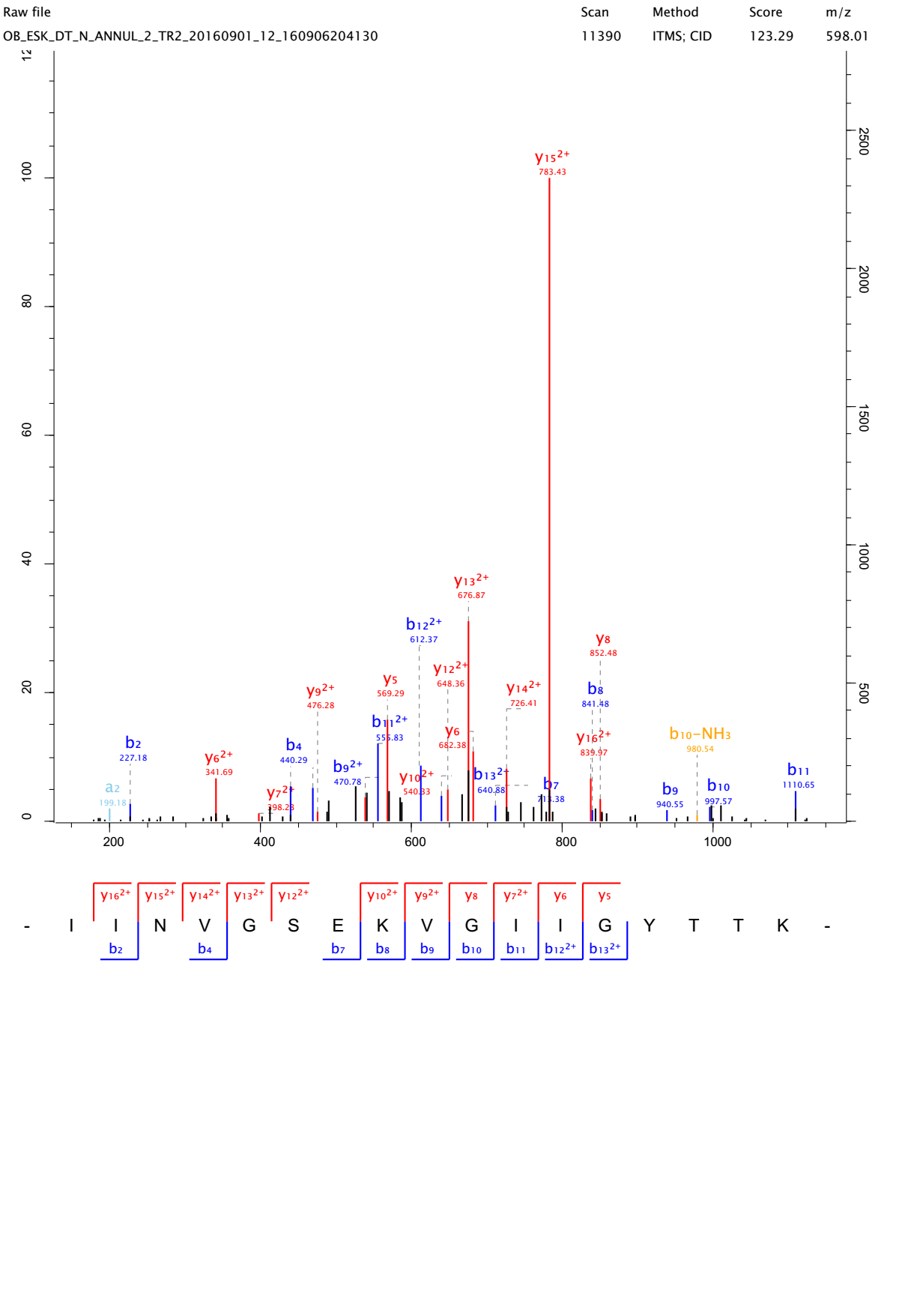


**Protein ID – A0A182C6D0**

**Protein name:** Ohanin OS=Phalotris mertensi OX=1260334 PE=4 SV=1

**Number of Unique Peptides:** 1

**m/z:** 762.87

**MS/MS ID:** 711

**Score:** 153.14

**Spectrum:** 1/1


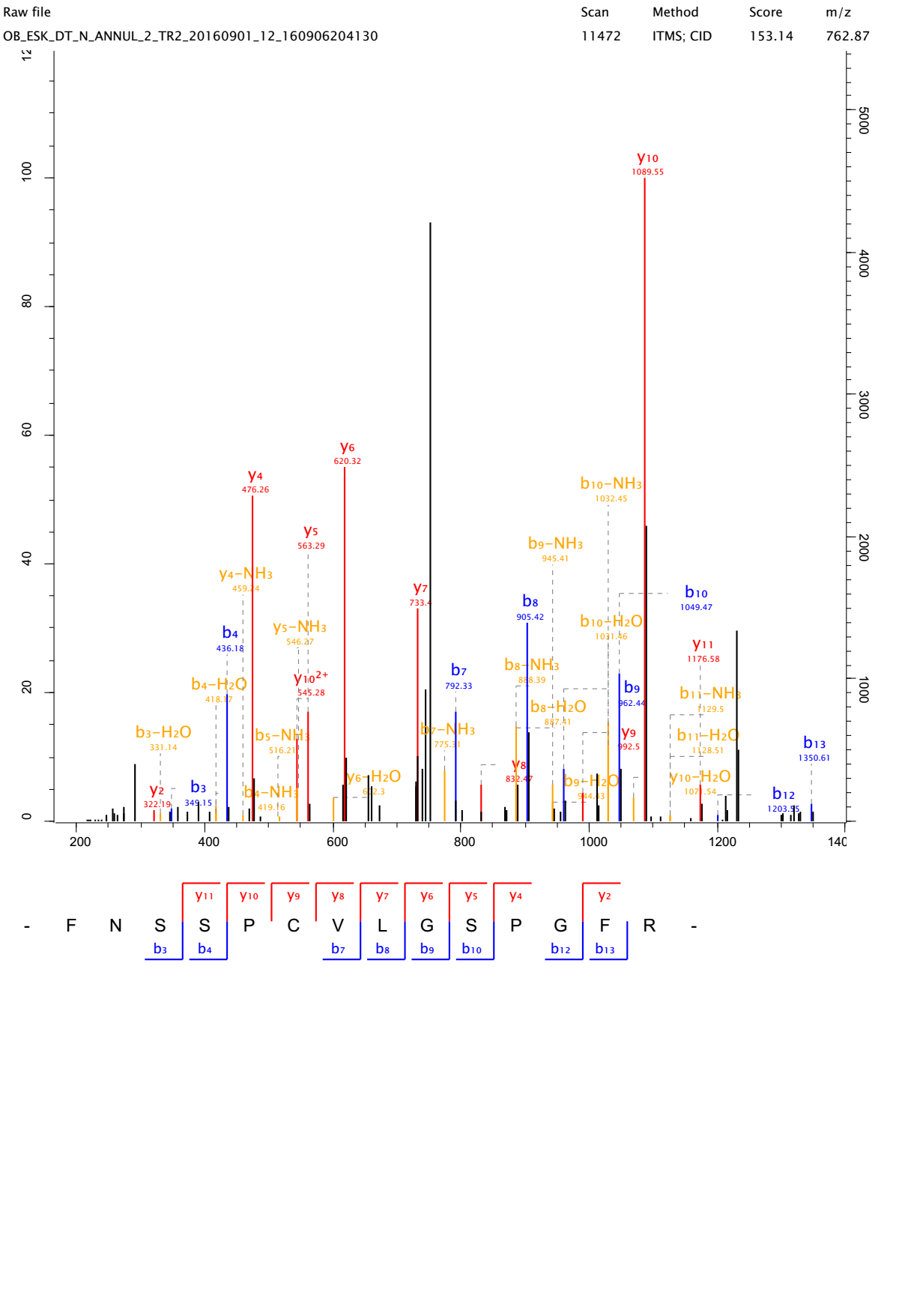


**Protein ID – U3FYQ4**

**Protein name:** Hyaluronidase OS=Micrurus fulvius OX=8637 PE=2 SV=1

**Number of Unique Peptides:** 2

**m/z:** 635.64

**MS/MS ID:** 1769

**Score:** 82.58

**Spectrum:** 1/2


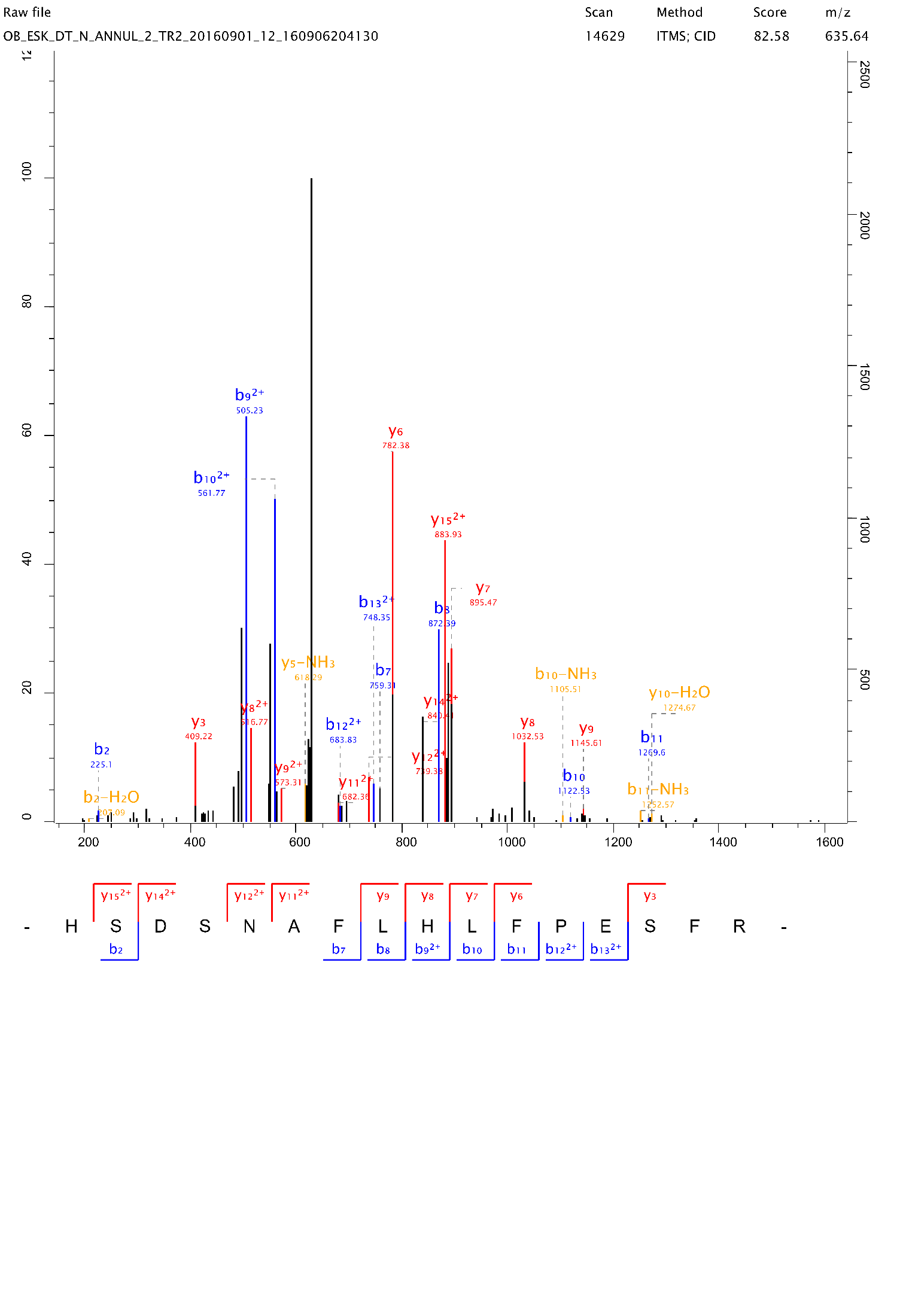


**Protein ID – U3FYQ4**

**Protein name:** Hyaluronidase OS=Micrurus fulvius OX=8637 PE=2 SV=1

**Number of Unique Peptides:** 2

**m/z:** 604.3

**MS/MS ID:** 3464

**Score:** 67.33

**Spectrum:** 2/2


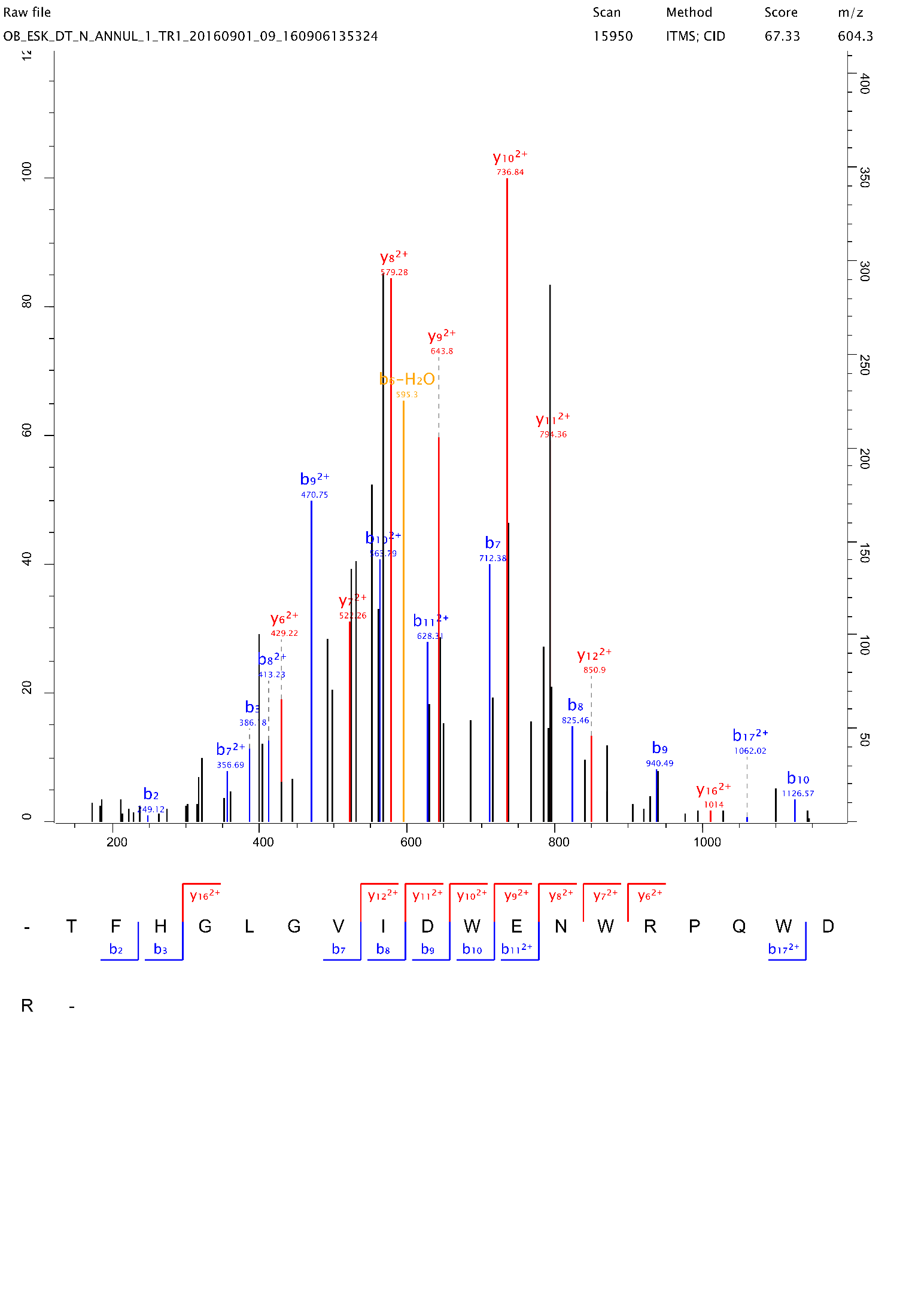


**Protein ID – Q9W717**

**Protein name:** Neurotoxin-like protein NTL2 OS=Naja atra OX=8656 PE=3 SV=1

**Number of Unique Peptides:** 1

**m/z:** 605.87

**MS/MS ID:** 1731

**Score:** 112.3

**Spectrum:** 1/1


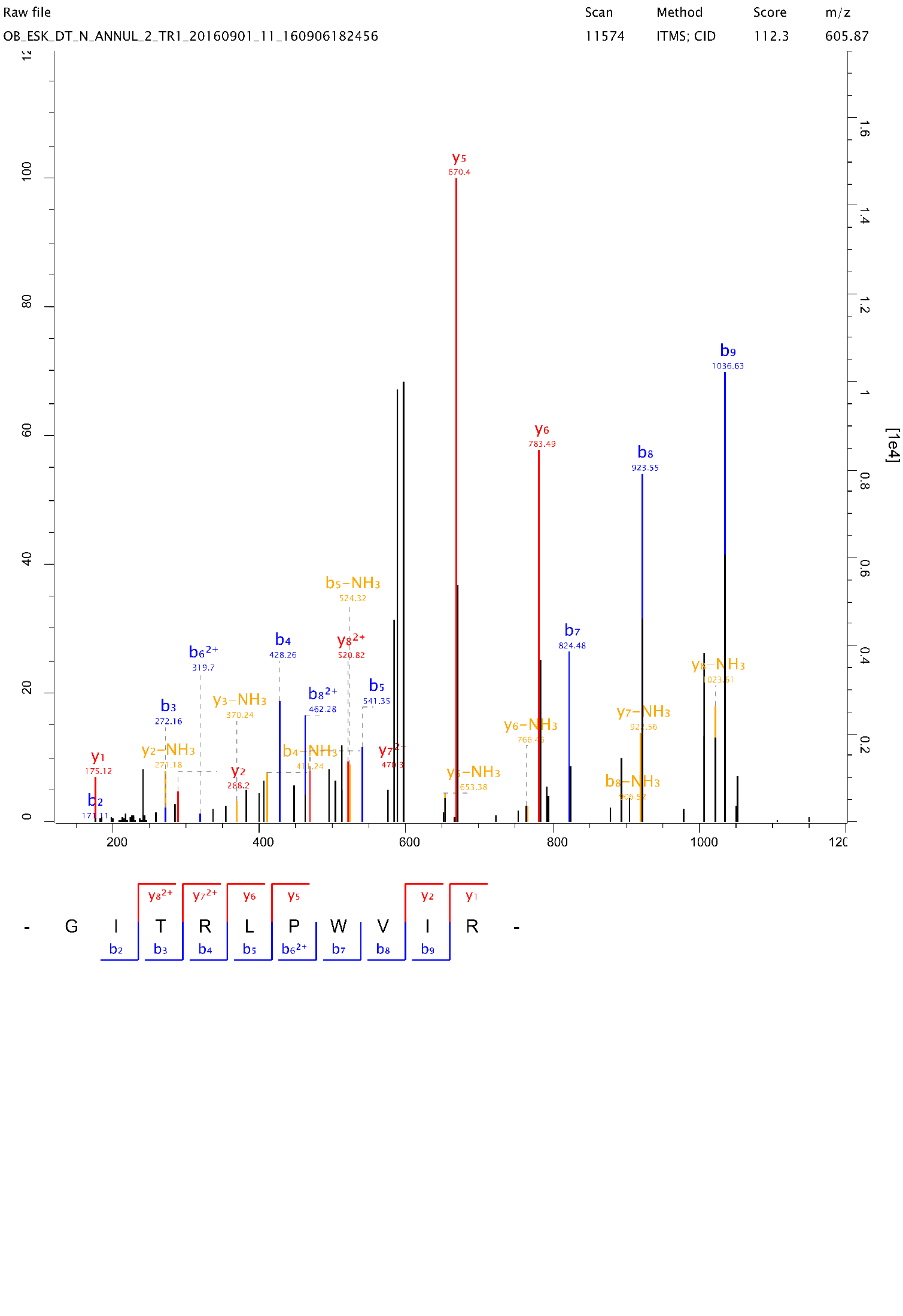


**Protein ID – V8P6K5**

**Protein name:** L-lactate dehydrogenase OS=Ophiophagus hannah OX=8665 GN=LDHA PE=3 SV=1

**Number of Unique Peptides:** 1

**m/z:** 664.03

**MS/MS ID:** 2023

**Score:** 95.55

**Spectrum:** 1/1


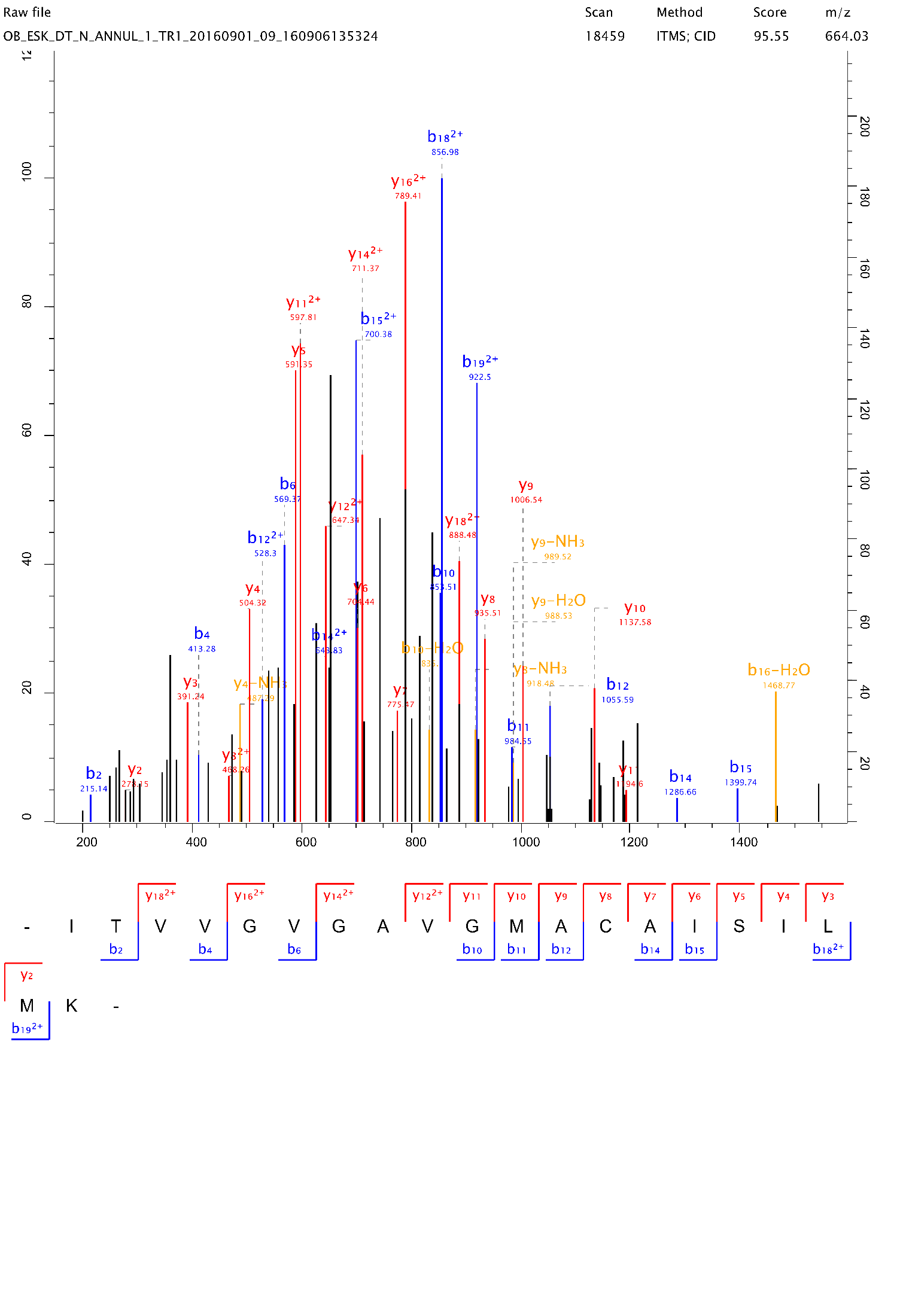


**Protein ID – F2Q6F2**

**Protein name:** Cysteine-rich seceretory protein Dr-CRPK OS=Daboia russelii OX=8707 PE=2 SV=1

**Number of Unique Peptides:** 1

**m/z:** 652.85

**MS/MS ID:** 3033

**Score:** 161.25

**Spectrum:** 1/1


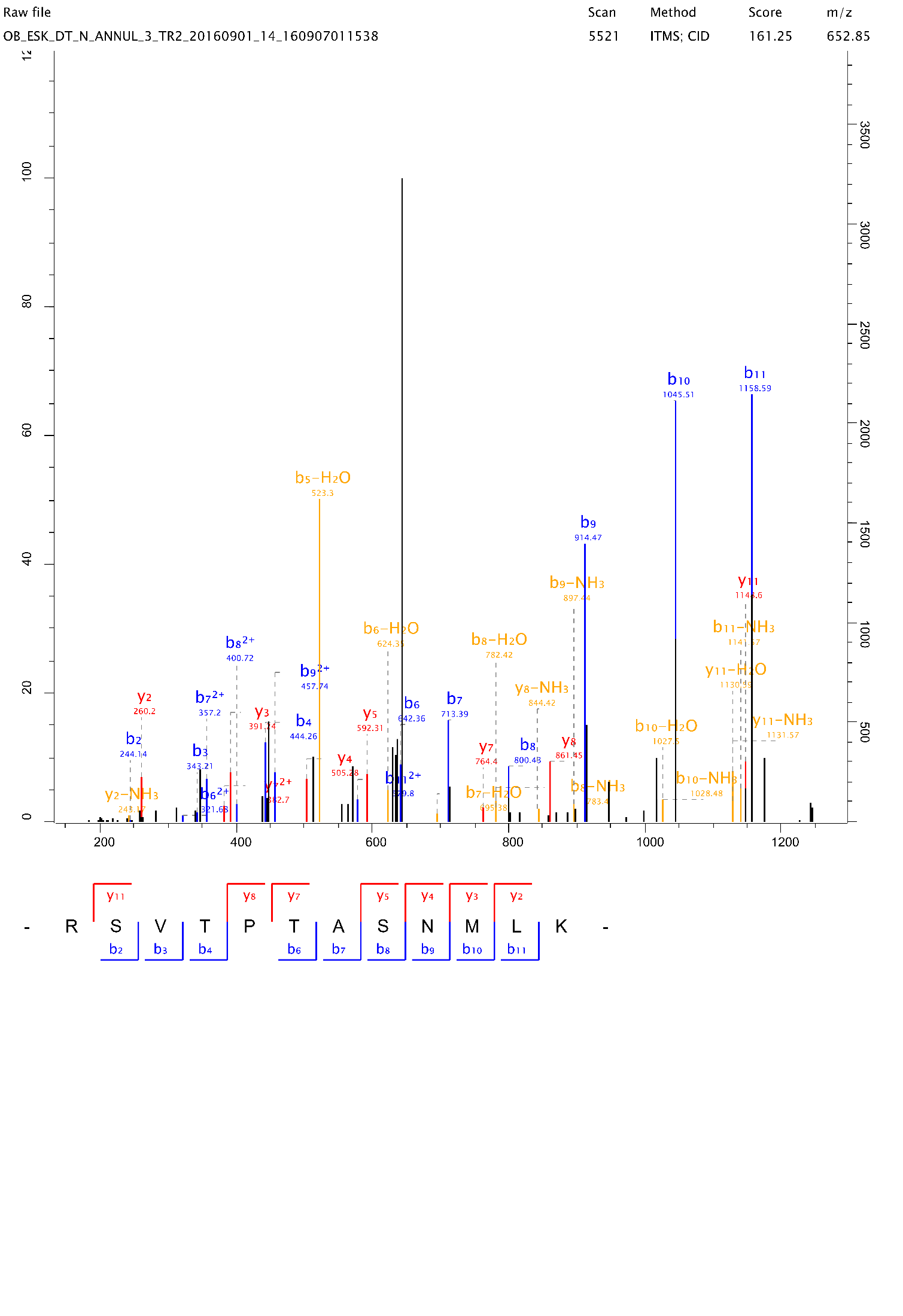


**Protein ID – A0A2D0TC04**

**Protein name:** Snake venom phosphodiesterase (PDE) OS=Naja atra OX=8656 PE=1 SV=1

**Number of Unique Peptides:** 9

**m/z:** 830.43

**MS/MS ID:** 217

**Score:** 62.03

**Spectrum:** 1/9


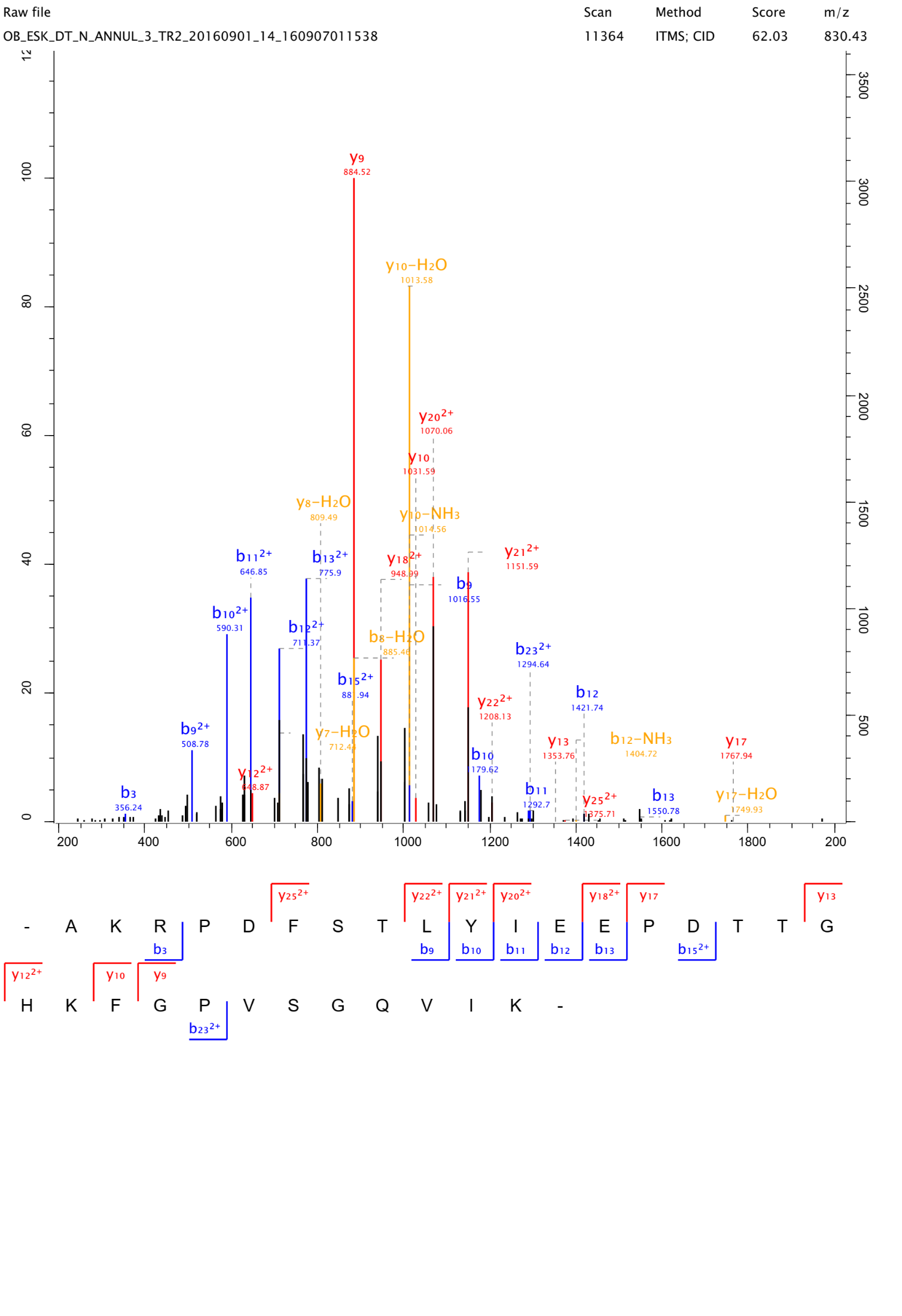


**Protein ID – A0A2D0TC04**

**Protein name:** Snake venom phosphodiesterase (PDE) OS=Naja atra OX=8656 PE=1 SV=1

**Number of Unique Peptides:** 9

**m/z:** 797.08

**MS/MS ID:** 499

**Score:** 123.14

**Spectrum:** 2/9


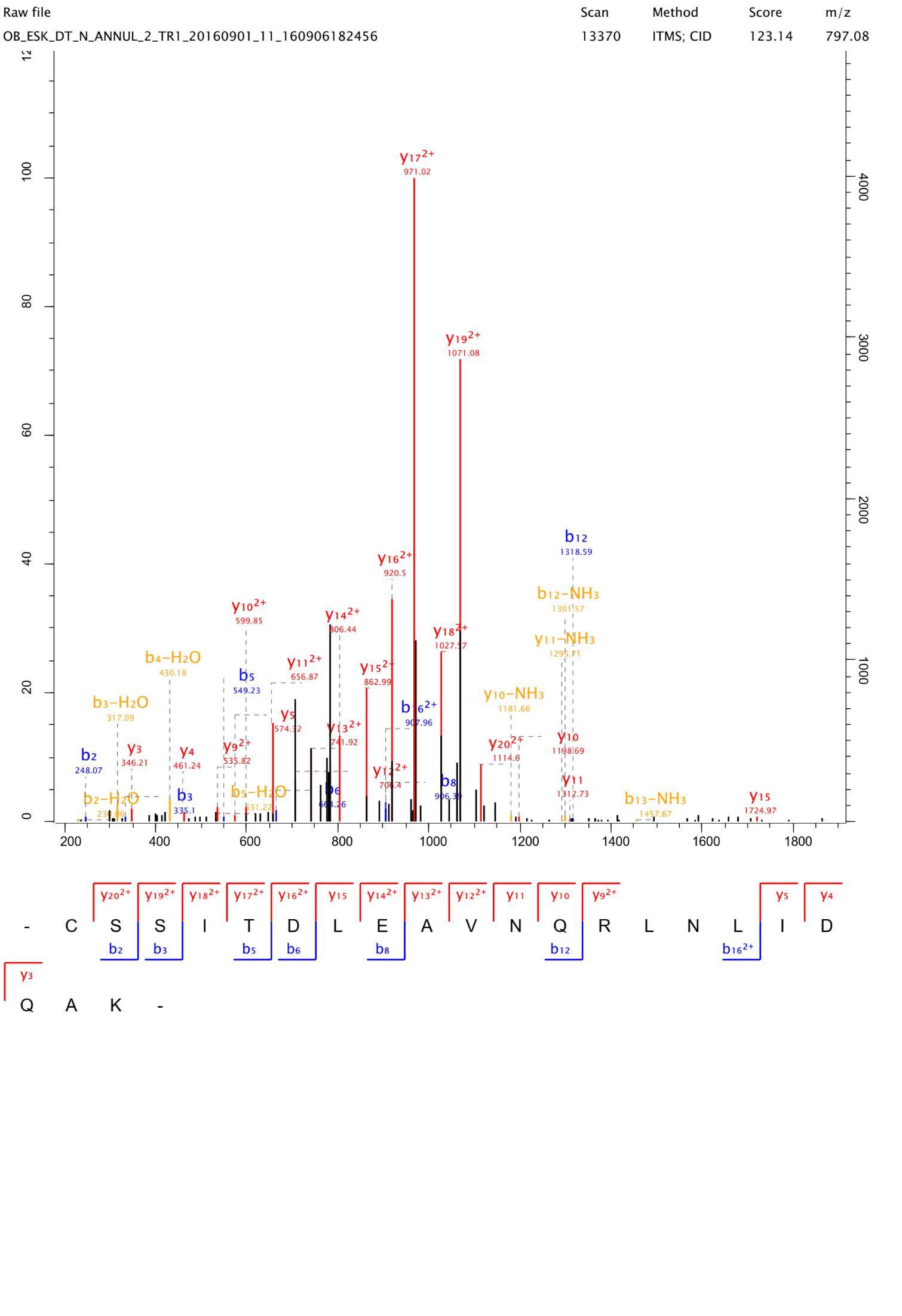


**Protein ID – A0A2D0TC04**

**Protein name:** Snake venom phosphodiesterase (PDE) OS=Naja atra OX=8656 PE=1 SV=1

**Number of Unique Peptides:** 9

**m/z:** 516.3

**MS/MS ID:** 646

**Score:** 141.88

**Spectrum:** 3/9


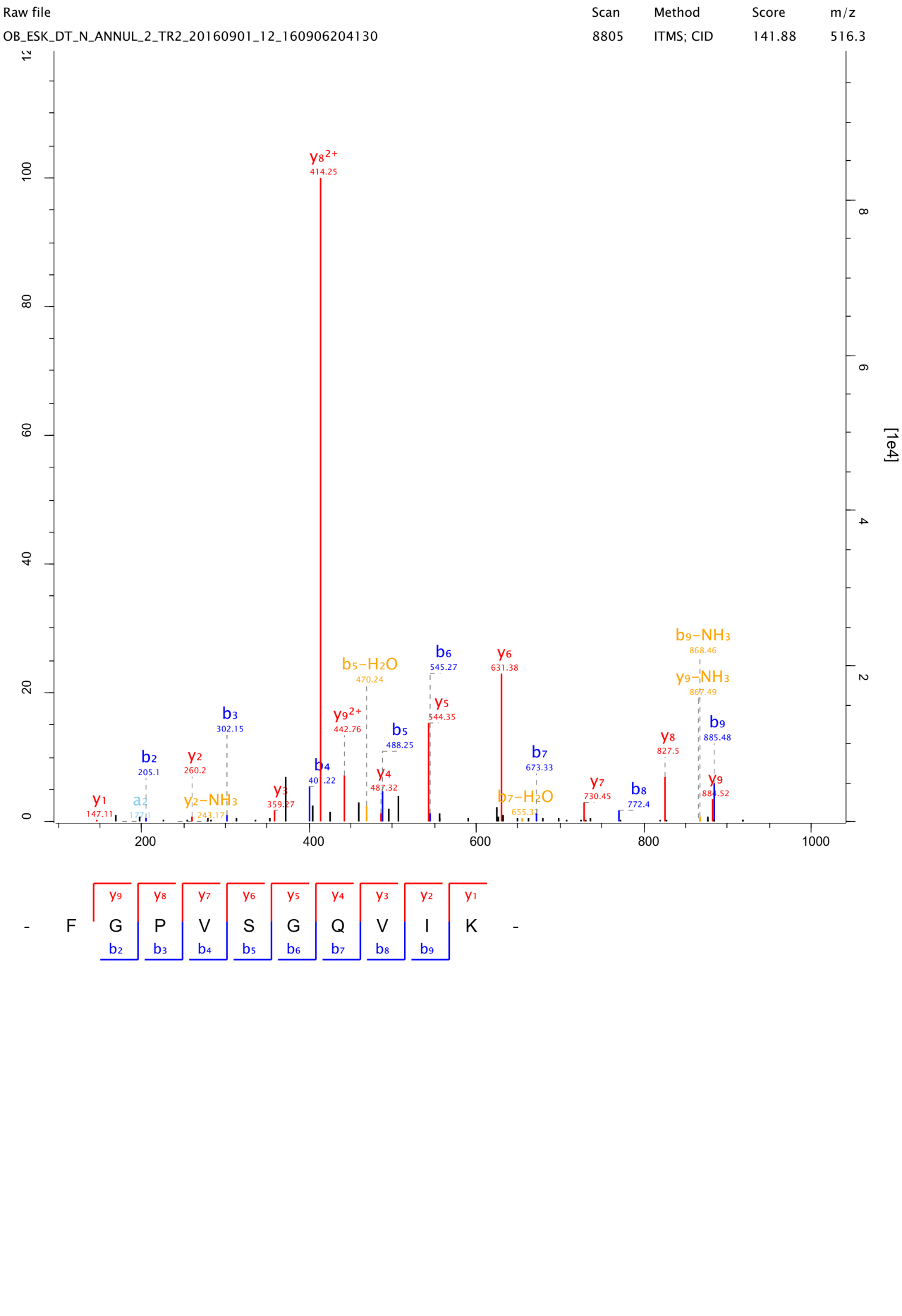


**Protein ID – A0A2D0TC04**

**Protein name:** Snake venom phosphodiesterase (PDE) OS=Naja atra OX=8656 PE=1 SV=1

**Number of Unique Peptides:** 9

**m/z:** 457.77

**MS/MS ID:** 2405

**Score:** 158.87

**Spectrum:** 4/9


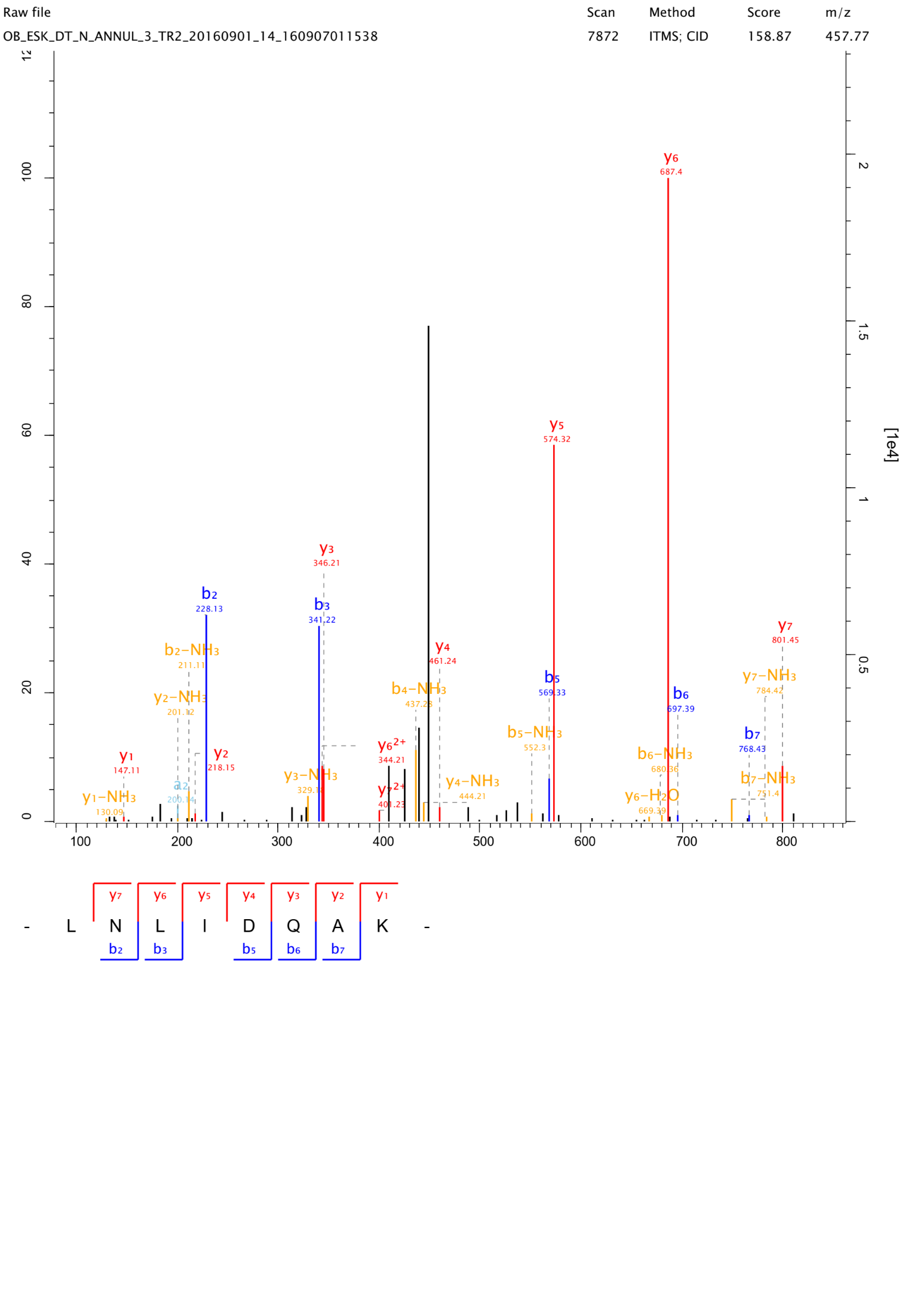


**Protein ID – A0A2D0TC04**

**Protein name:** Snake venom phosphodiesterase (PDE) OS=Naja atra OX=8656 PE=1 SV=1

**Number of Unique Peptides:** 9

**m/z:** 758.04

**MS/MS ID:** 2802

**Score:** 74.14

**Spectrum:** 5/9


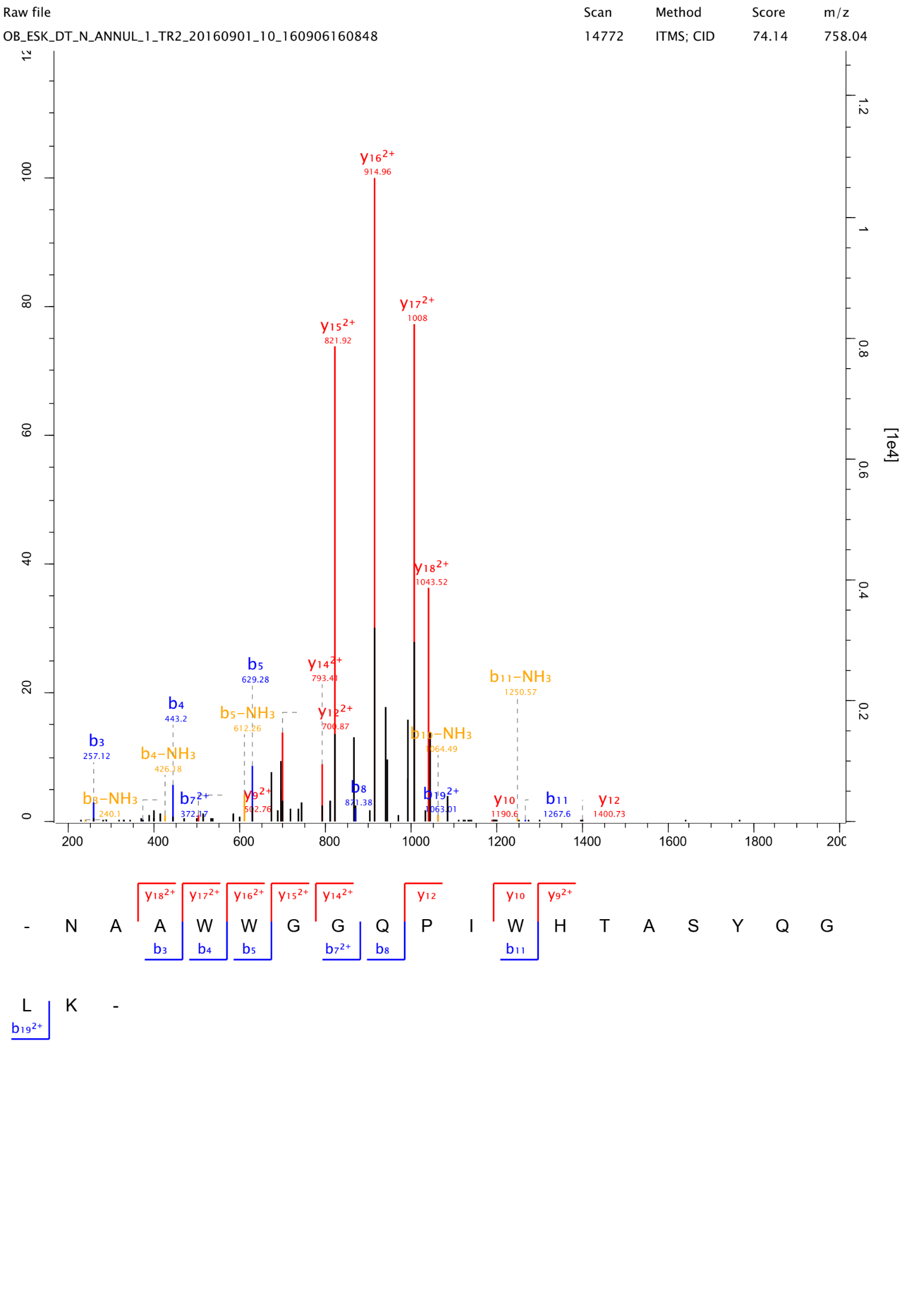


**Protein ID – A0A2D0TC04**

**Protein name:** Snake venom phosphodiesterase (PDE) OS=Naja atra OX=8656 PE=1 SV=1

**Number of Unique Peptides:** 9

**m/z:** 668.02

**MS/MS ID:** 2881

**Score:** 156.57

**Spectrum:** 6/9


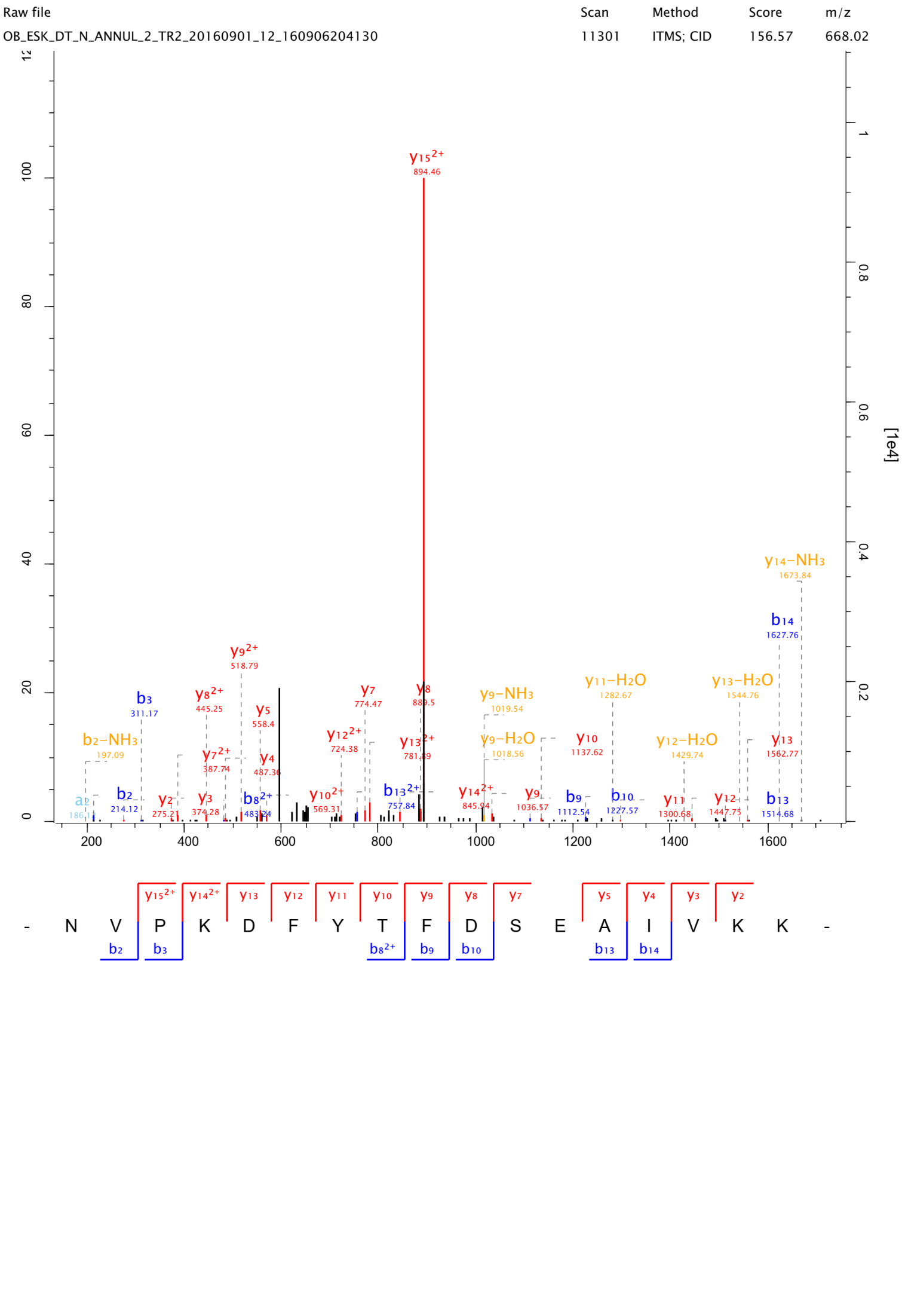


**Protein ID – A0A2D0TC04**

**Protein name:** Snake venom phosphodiesterase (PDE) OS=Naja atra OX=8656 PE=1 SV=1

**Number of Unique Peptides:** 9

**m/z:** 780.9

**MS/MS ID:** 3020

**Score:** 76.15

**Spectrum:** 7/9


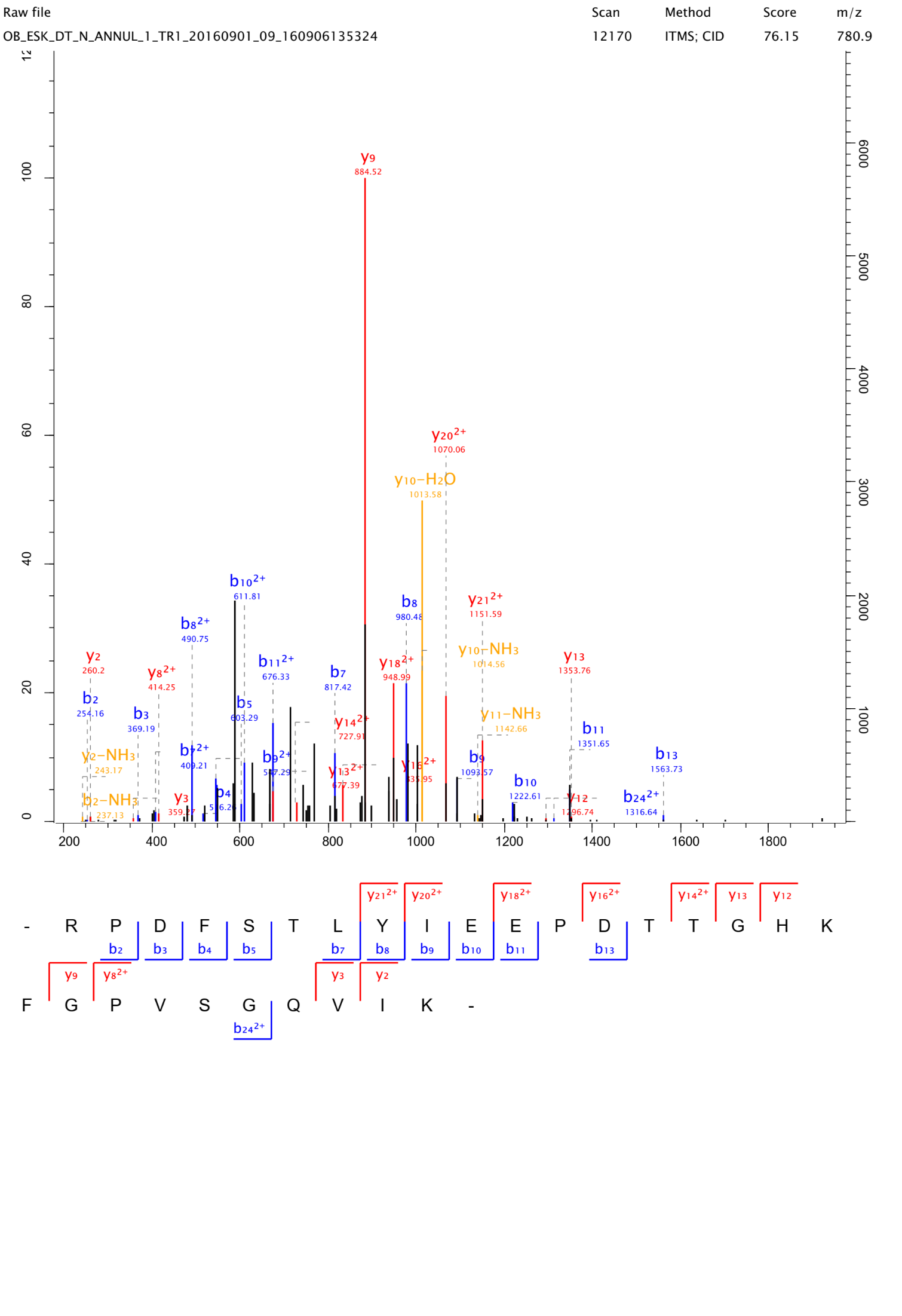


**Protein ID – A0A2D0TC04**

**Protein name:** Snake venom phosphodiesterase (PDE) OS=Naja atra OX=8656 PE=1 SV=1

**Number of Unique Peptides:** 9

**m/z:** 647.36

**MS/MS ID:** 3568

**Score:** 97.13

**Spectrum:** 8/9


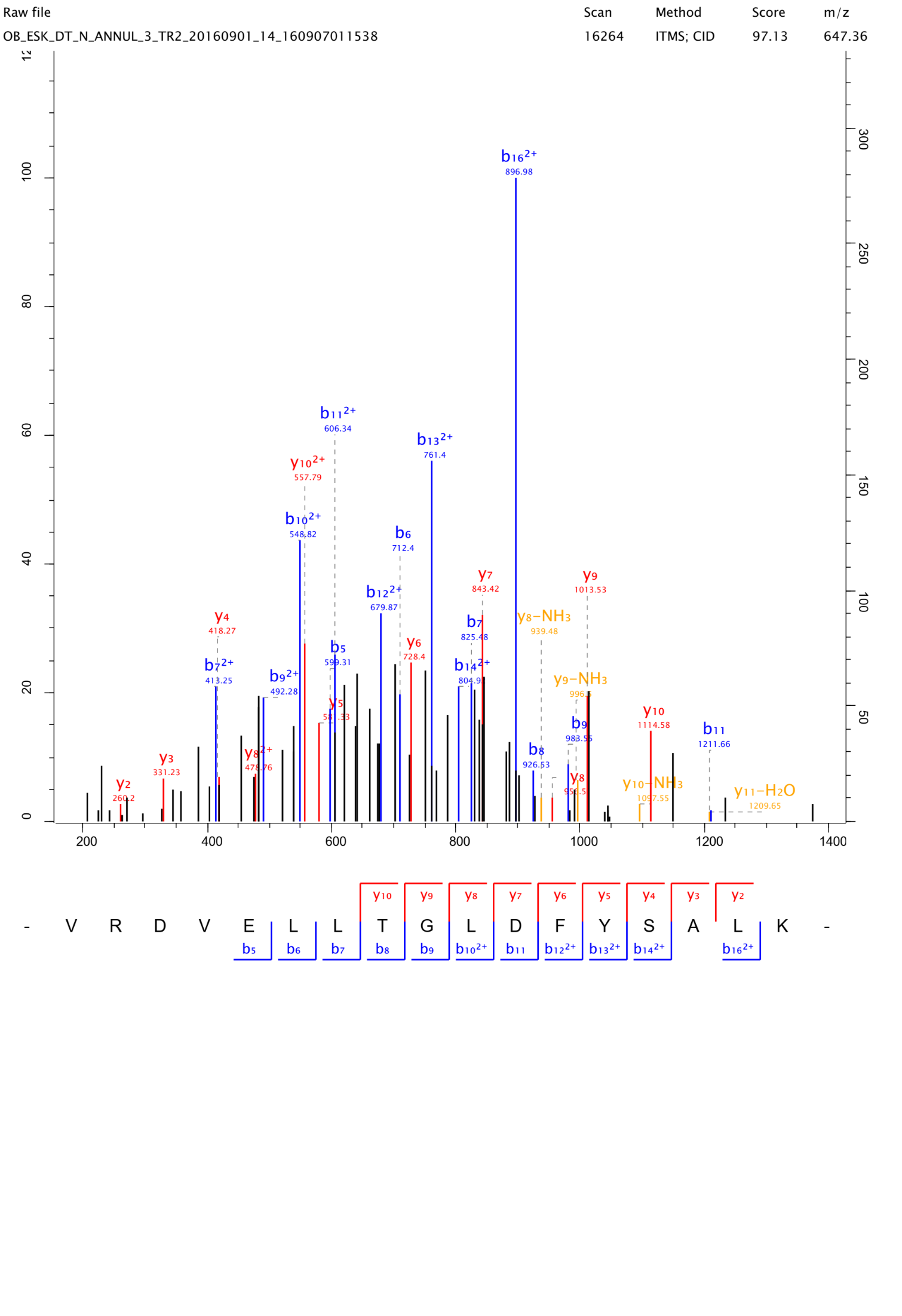


**Protein ID – A0A2D0TC04**

**Protein name:** Snake venom phosphodiesterase (PDE) OS=Naja atra OX=8656 PE=1 SV=1

**Number of Unique Peptides:** 9

**m/z:** 716.9

**MS/MS ID:** 3573

**Score:** 161.55

**Spectrum:** 9/9


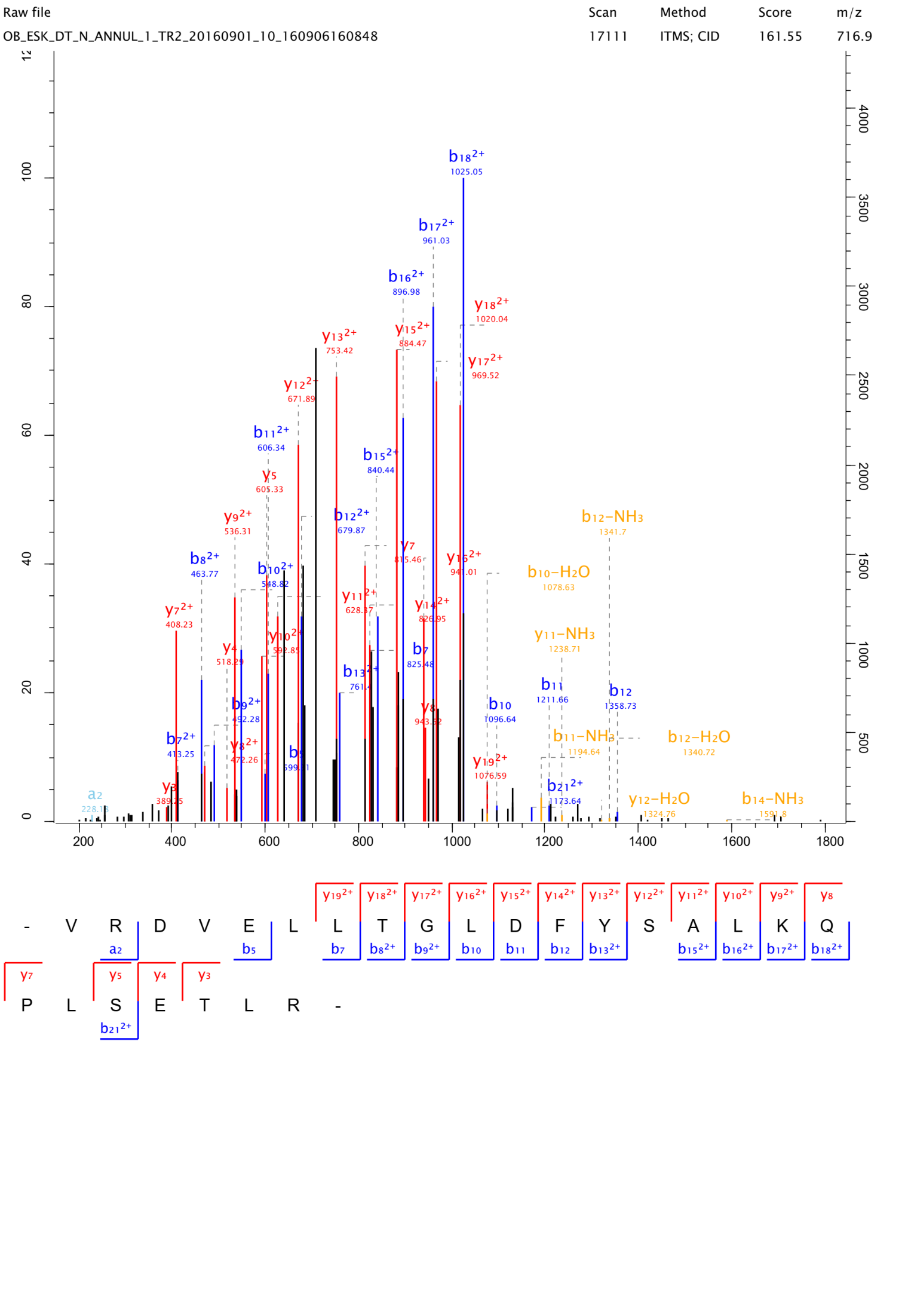


**Protein ID – A0A2D4GN98**

**Protein name:** Uncharacterized protein (Fragment) OS=Micrurus corallinus OX=54390 PE=4 SV=1

**Number of Unique Peptides:** 1

**m/z:** 591.33

**MS/MS ID:** 273

**Score:** 169.82

**Spectrum:** 1/1


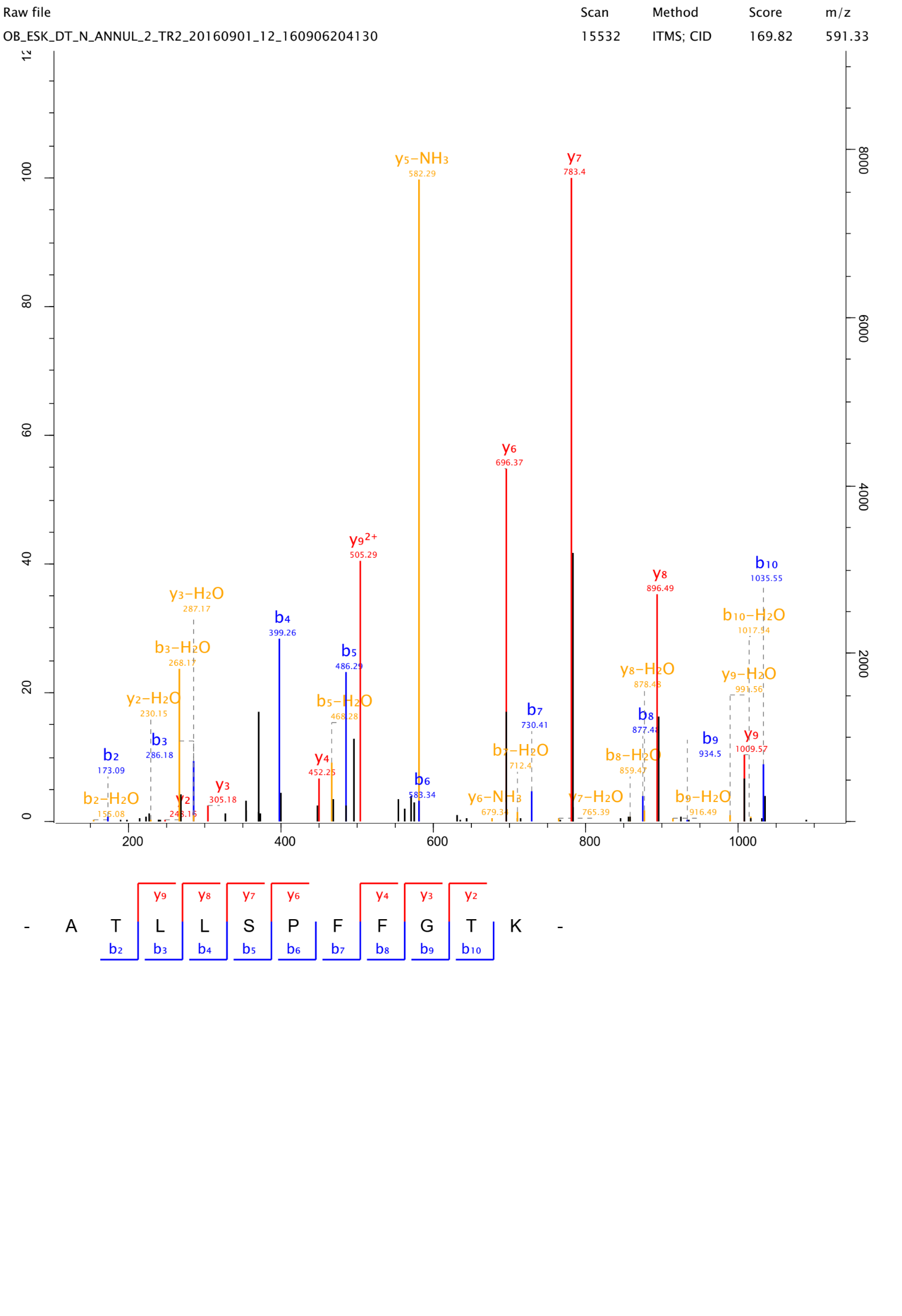


**Protein ID – A0A2D4H759**

**Protein name:** Uncharacterized protein (Fragment) OS=Micrurus lemniscatus lemniscatus OX=129467 PE=4 SV=1

**Number of Unique Peptides:** 1

**m/z:** 618.35

**MS/MS ID:** 3589

**Score:** 103.26

**Spectrum:** 1/1


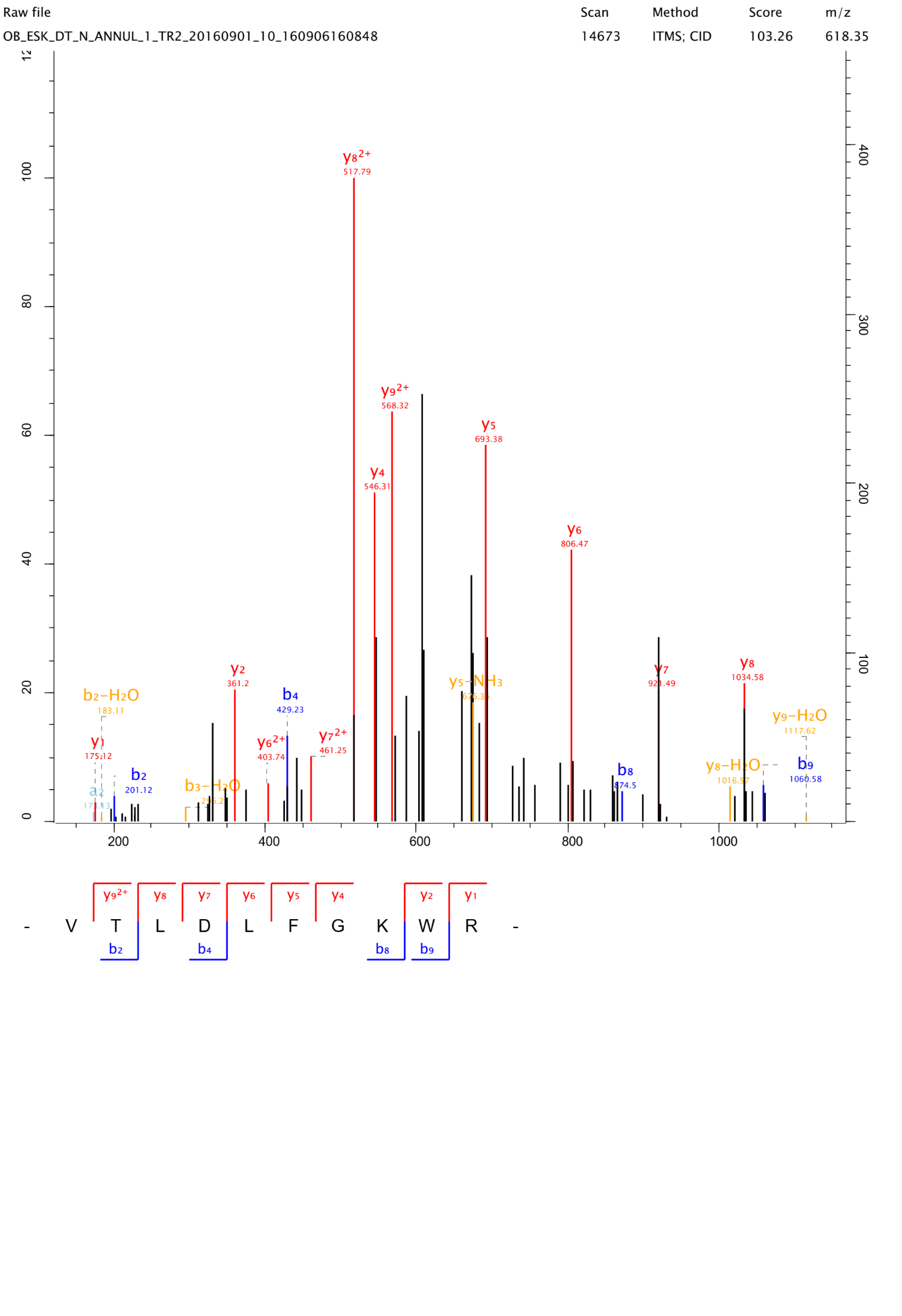


**Protein ID – A0A2D4KYJ8**

**Protein name:** Uncharacterized protein (Fragment) OS=Micrurus paraensis OX=1970185 PE=4 SV=1

**Number of Unique Peptides:** 1

**m/z:** 677.85

**MS/MS ID:** 3058

**Score:** 243.37

**Spectrum:** 1/1


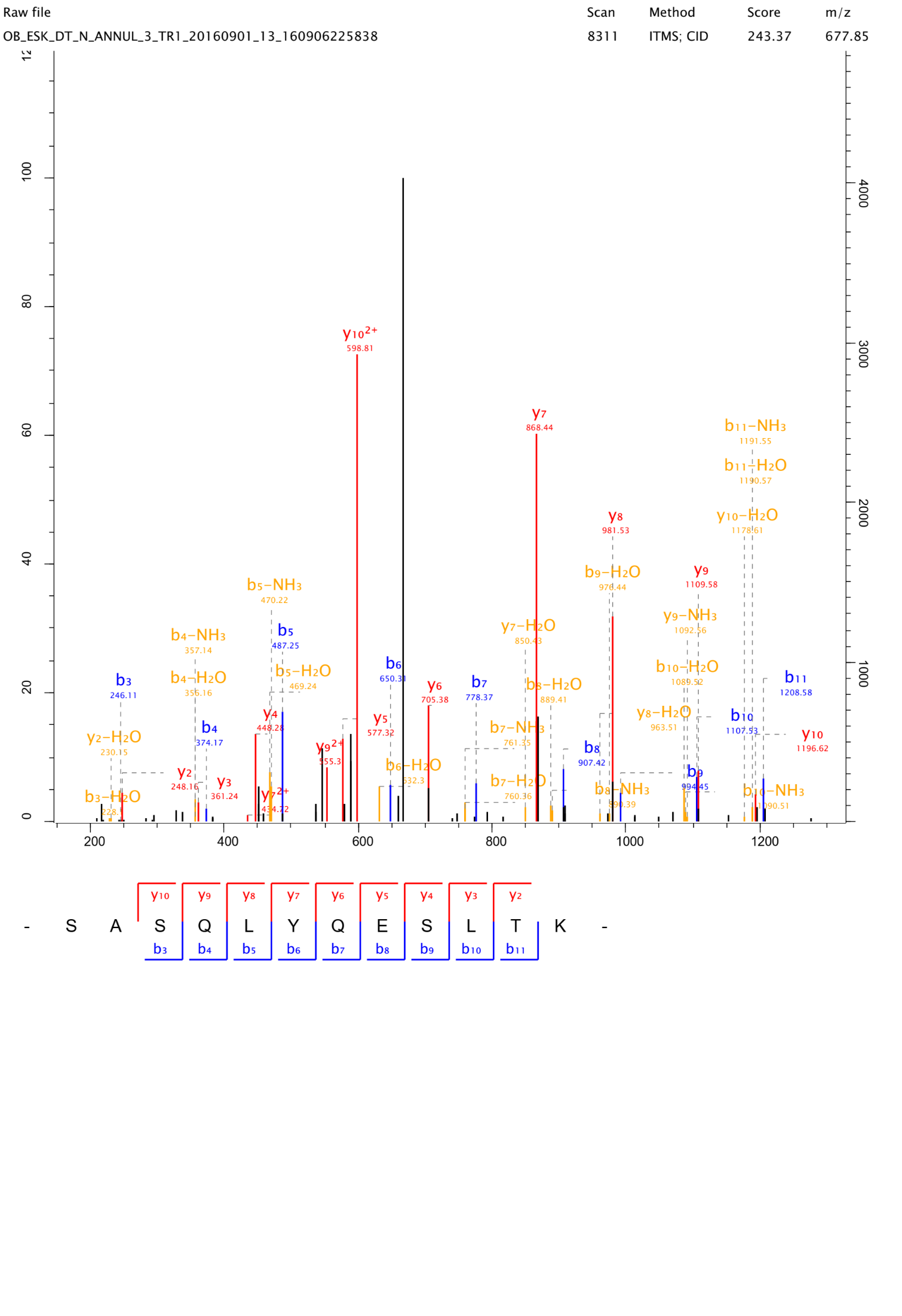


**Protein ID – A0A2R4N4Q6**

**Protein name:** Amine oxidase (Fragment) OS=Naja atra OX=8656 PE=2 SV=1

**Number of Unique Peptides:** 10

**m/z:** 661.34

**MS/MS ID:** 561

**Score:** 142.1

**Spectrum:** 1/10


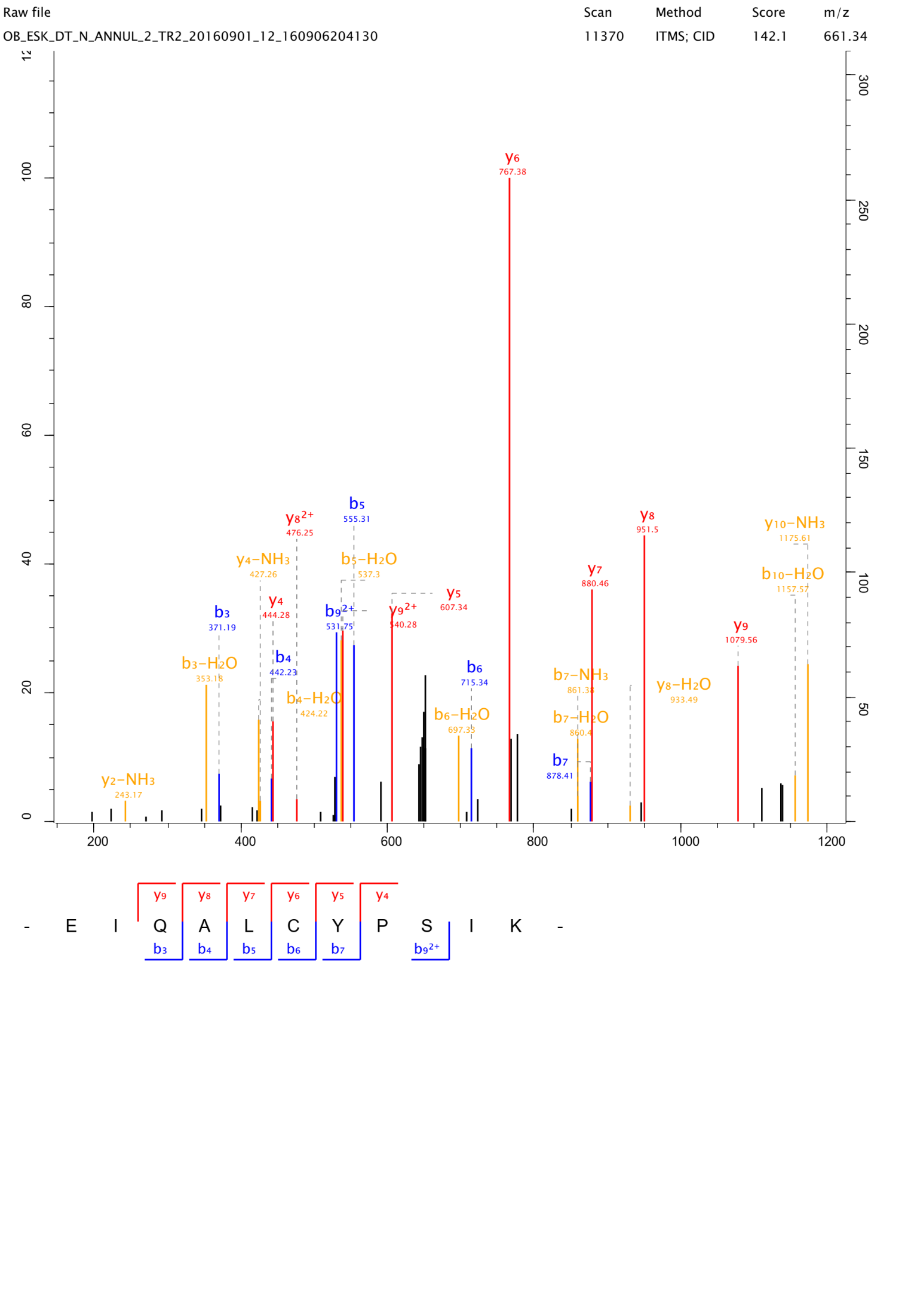


**Protein ID – A0A2R4N4Q6**

**Protein name:** Amine oxidase (Fragment) OS=Naja atra OX=8656 PE=2 SV=1

**Number of Unique Peptides:** 10

**m/z:** 567.3

**MS/MS ID:** 564

**Score:** 166.66

**Spectrum:** 2/10


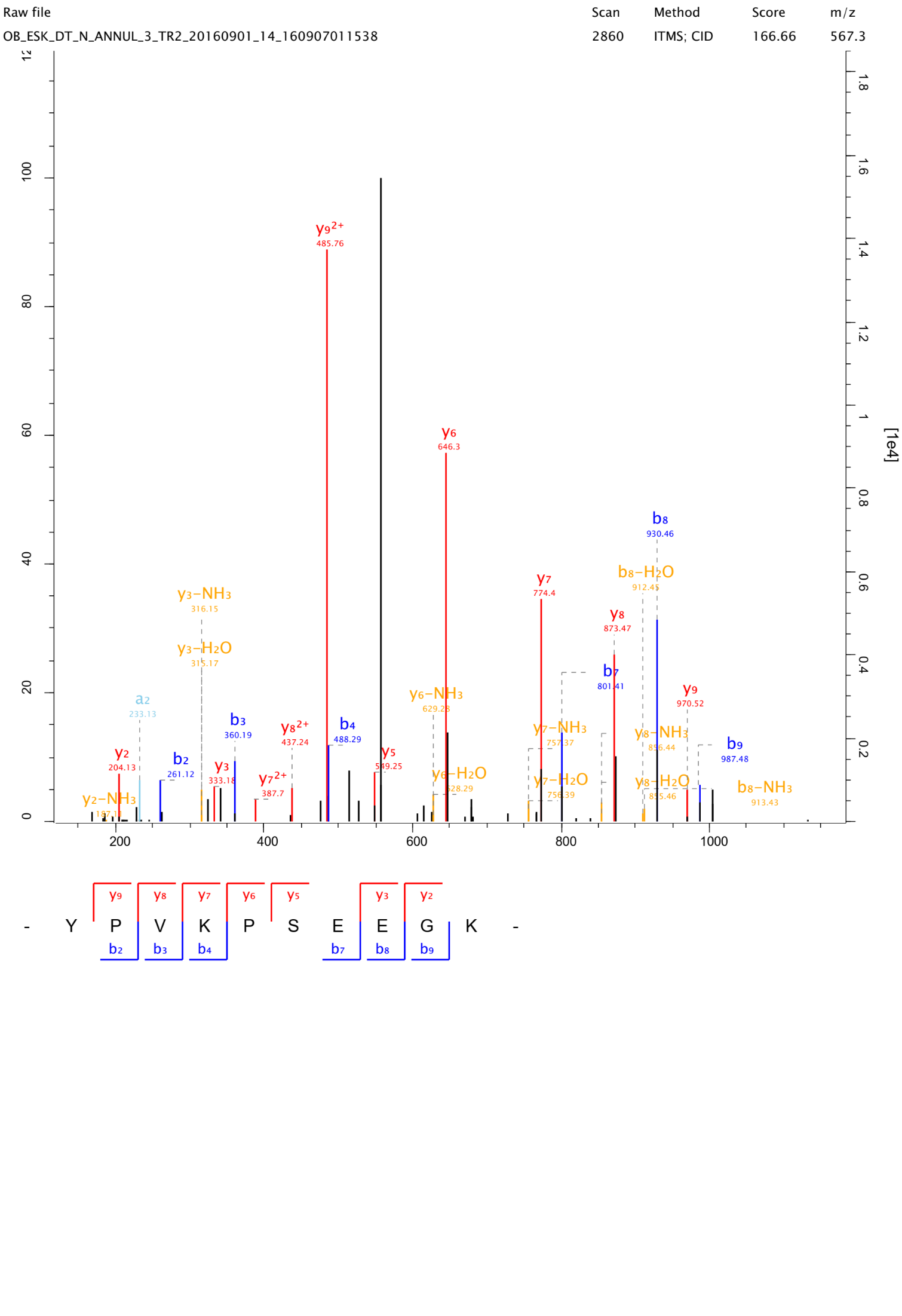


**Protein ID – A0A2R4N4Q6**

**Protein name:** Amine oxidase (Fragment) OS=Naja atra OX=8656 PE=2 SV=1

**Number of Unique Peptides:** 10

**m/z:** 725.39

**MS/MS ID:** 659

**Score:** 123.28

**Spectrum:** 3/10


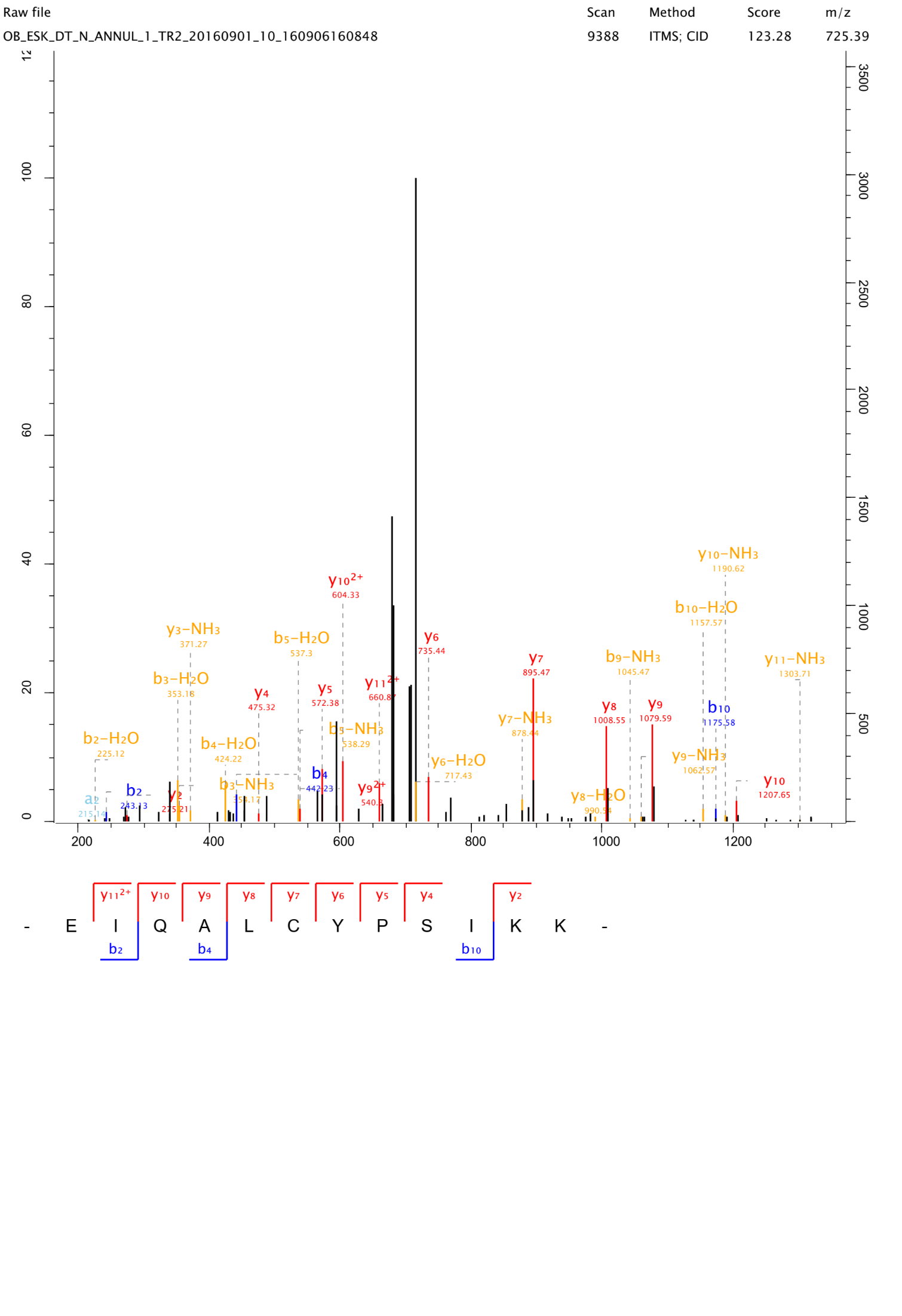


**Protein ID – A0A2R4N4Q6**

**Protein name:** Amine oxidase (Fragment) OS=Naja atra OX=8656 PE=2 SV=1

**Number of Unique Peptides:** 10

**m/z:** 826.72

**MS/MS ID:** 2393

**Score:** 193.32

**Spectrum:** 4/10


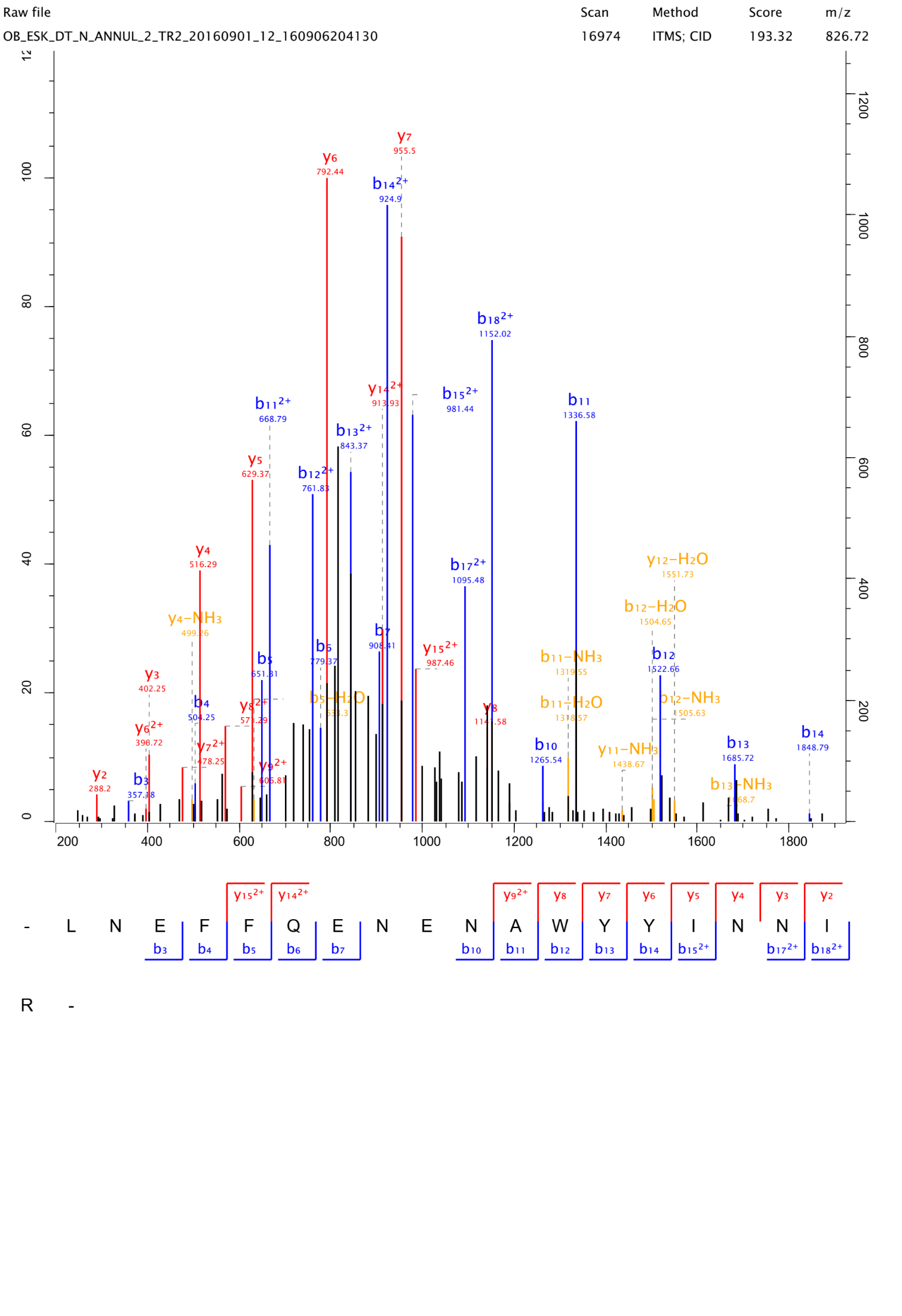


**Protein ID – A0A2R4N4Q6**

**Protein name:** Amine oxidase (Fragment) OS=Naja atra OX=8656 PE=2 SV=1

**Number of Unique Peptides:** 10

**m/z:** 869.42

**MS/MS ID:** 2399

**Score:** 118.31

**Spectrum:** 5/10


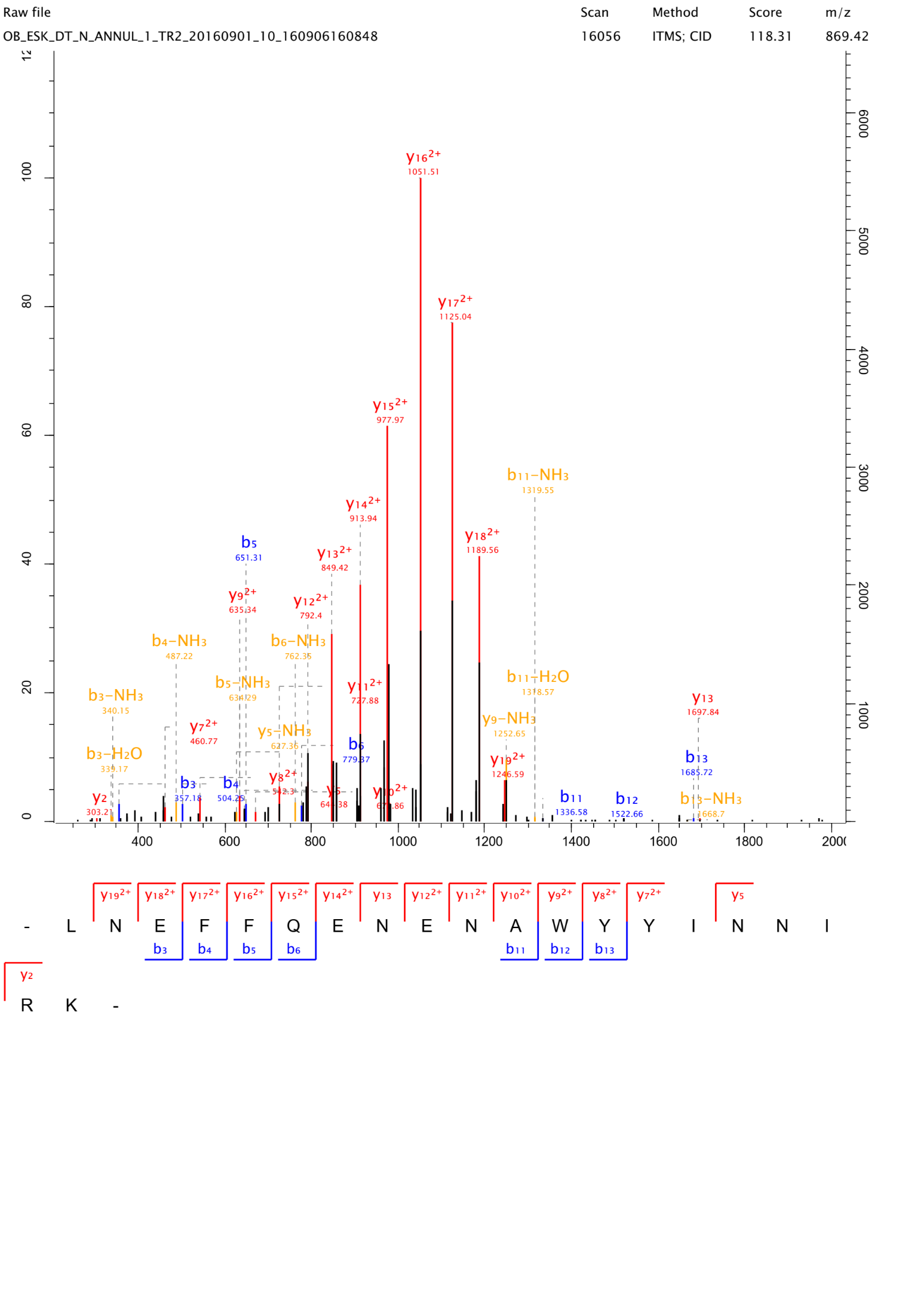


**Protein ID – A0A2R4N4Q6**

**Protein name:** Amine oxidase (Fragment) OS=Naja atra OX=8656 PE=2 SV=1

**Number of Unique Peptides:** 10

**m/z:** 680.03

**MS/MS ID:** 3424

**Score:** 143.88

**Spectrum:** 6/10


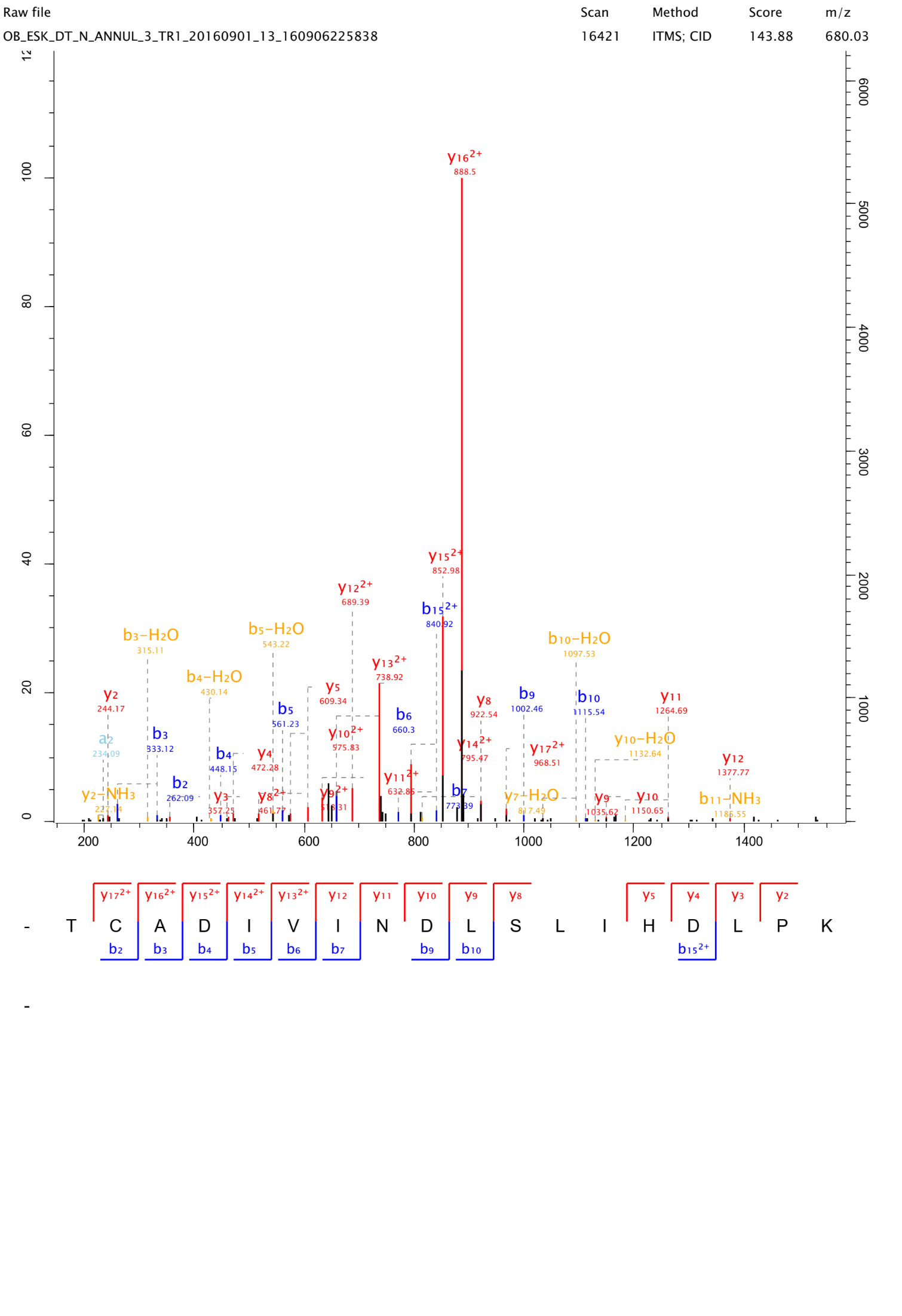


**Protein ID – A0A2R4N4Q6**

**Protein name:** Amine oxidase (Fragment) OS=Naja atra OX=8656 PE=2 SV=1

**Number of Unique Peptides:** 10

**m/z:** 509.28

**MS/MS ID:** 3596

**Score:** 167.67

**Spectrum:** 7/10


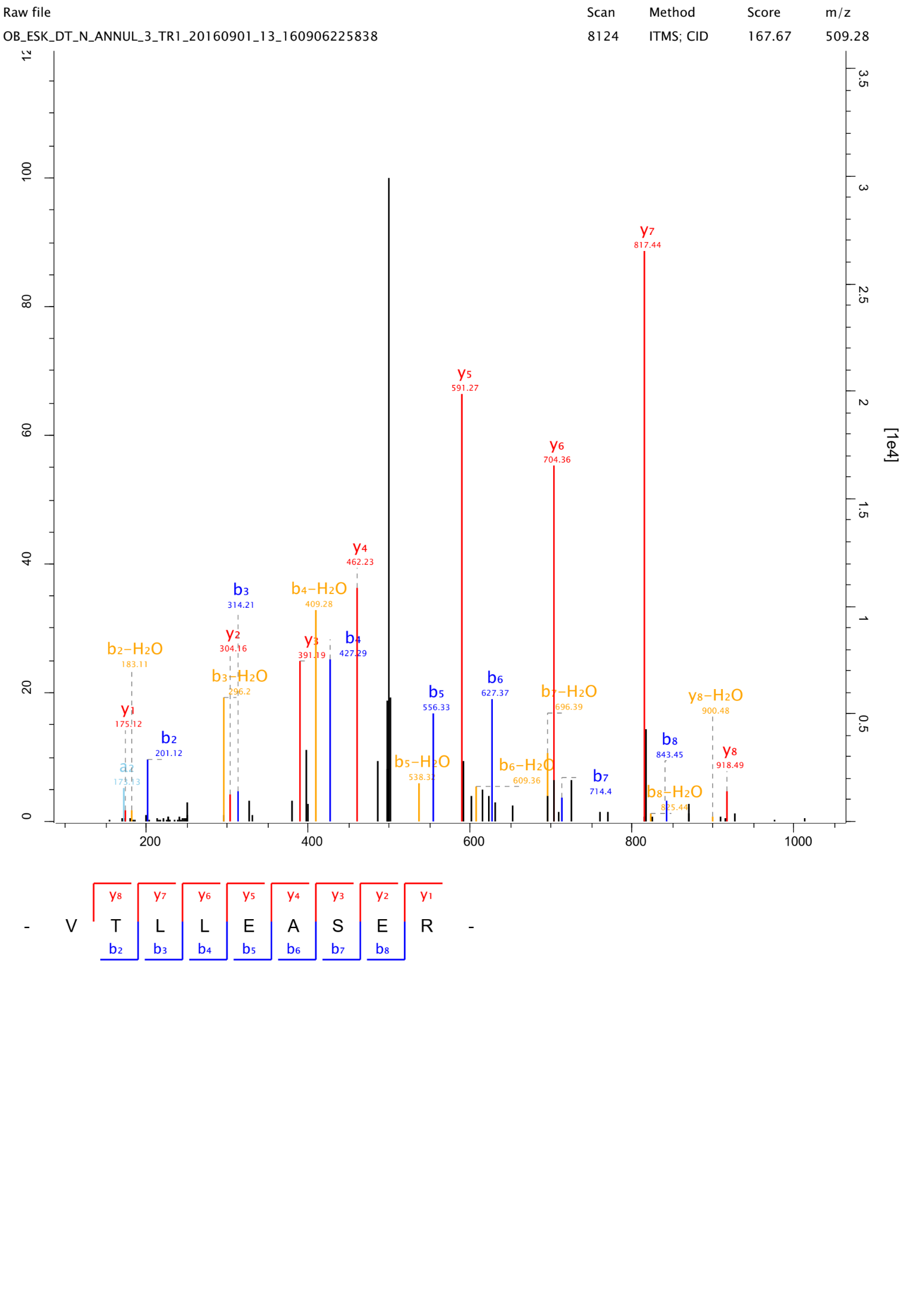


**Protein ID – A0A2R4N4Q6**

**Protein name:** Amine oxidase (Fragment) OS=Naja atra OX=8656 PE=2 SV=1

**Number of Unique Peptides:** 10

**m/z:** 732.06

**MS/MS ID:** 3431

**Score:** 109.55

**Spectrum:** 8/10


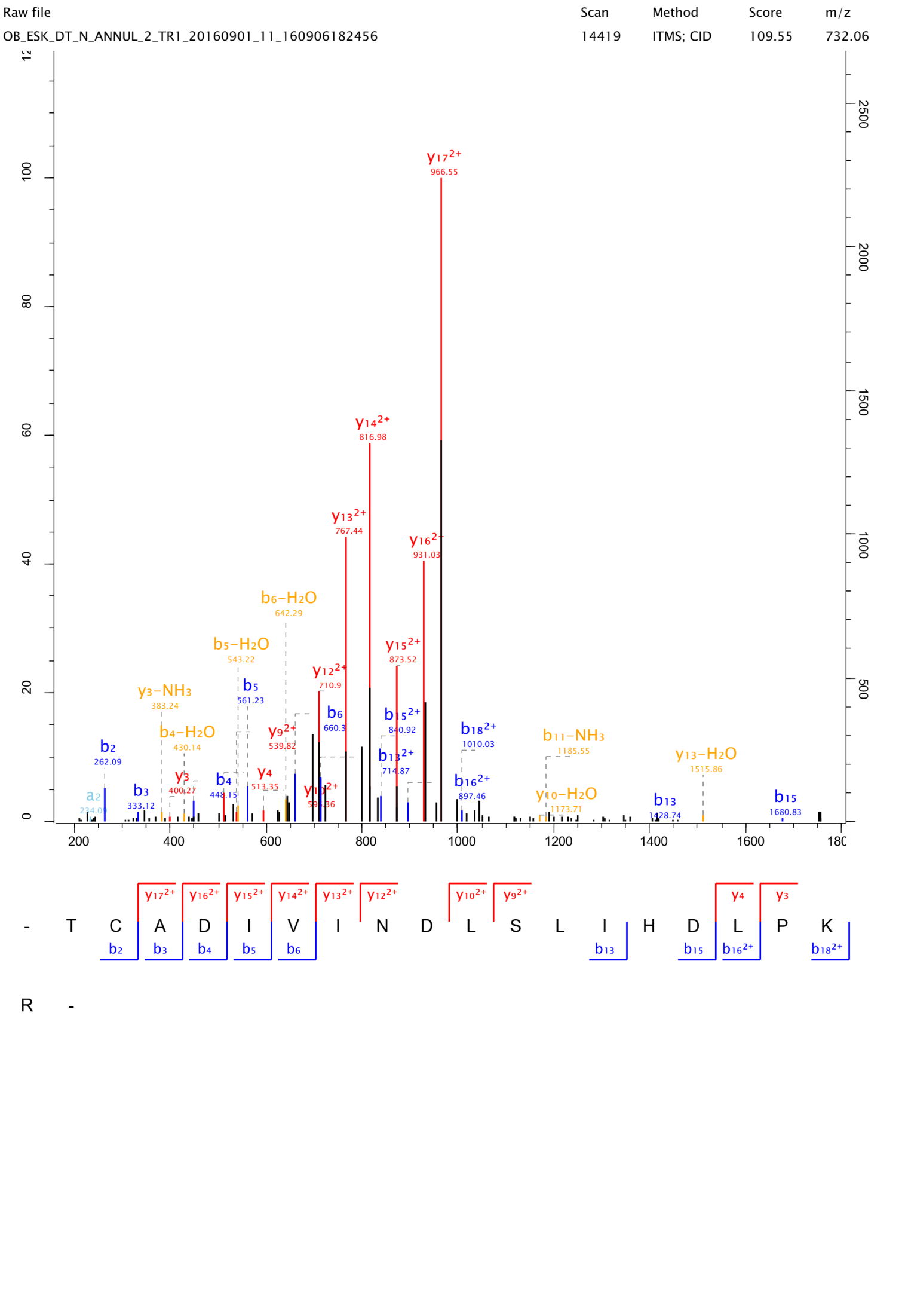


**Protein ID – A0A2R4N4Q6**

**Protein name:** Amine oxidase (Fragment) OS=Naja atra OX=8656 PE=2 SV=1

**Number of Unique Peptides:** 10

**m/z:** 454.25

**MS/MS ID:** 3598

**Score:** 143.37

**Spectrum:** 9/10


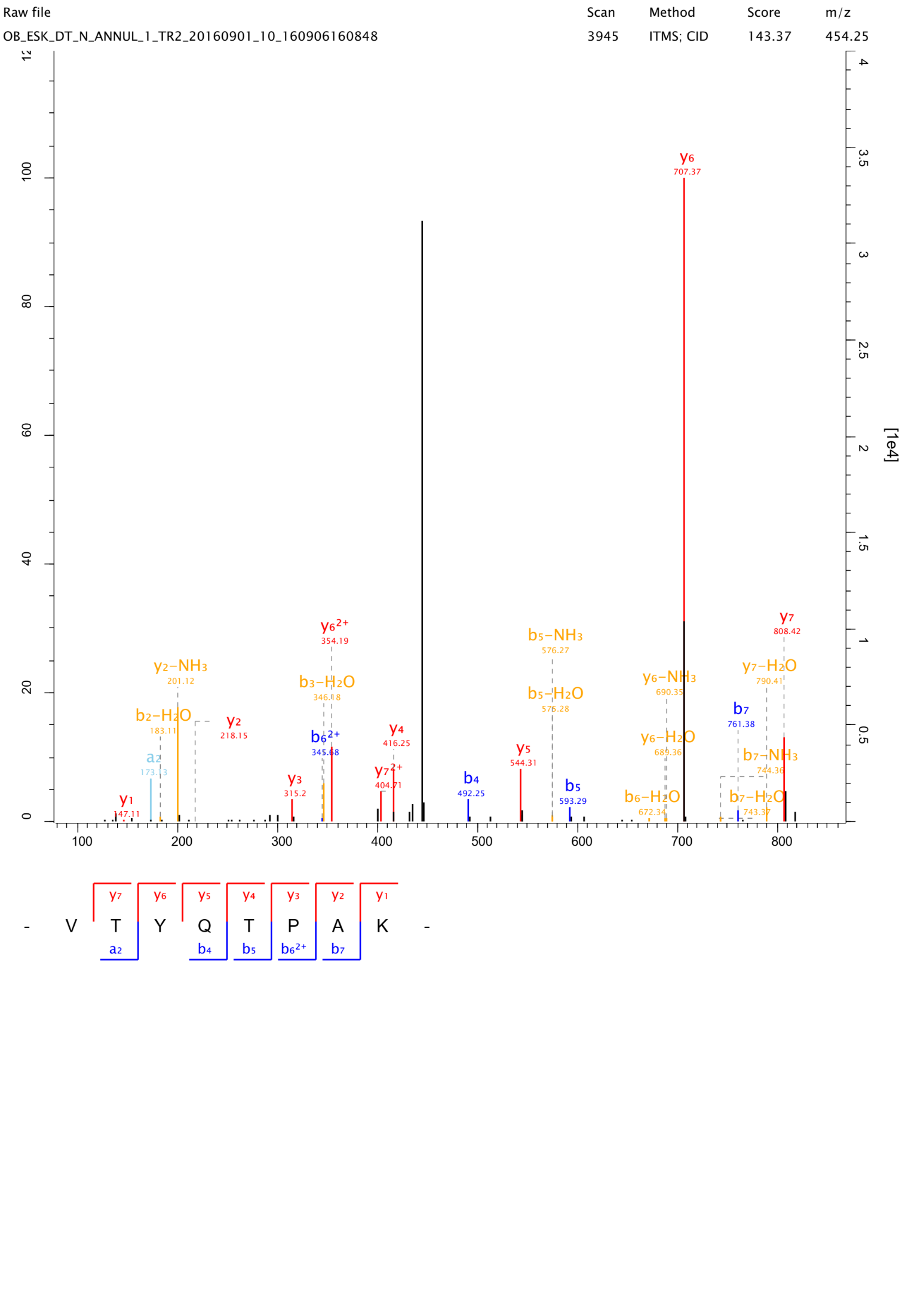


**Protein ID – A0A2R4N4Q6**

**Protein name:** Amine oxidase (Fragment) OS=Naja atra OX=8656 PE=2 SV=1

**Number of Unique Peptides:** 10

**m/z:** 567.3

**MS/MS ID:** 4011

**Score:** 166.66

**Spectrum:** 10/10


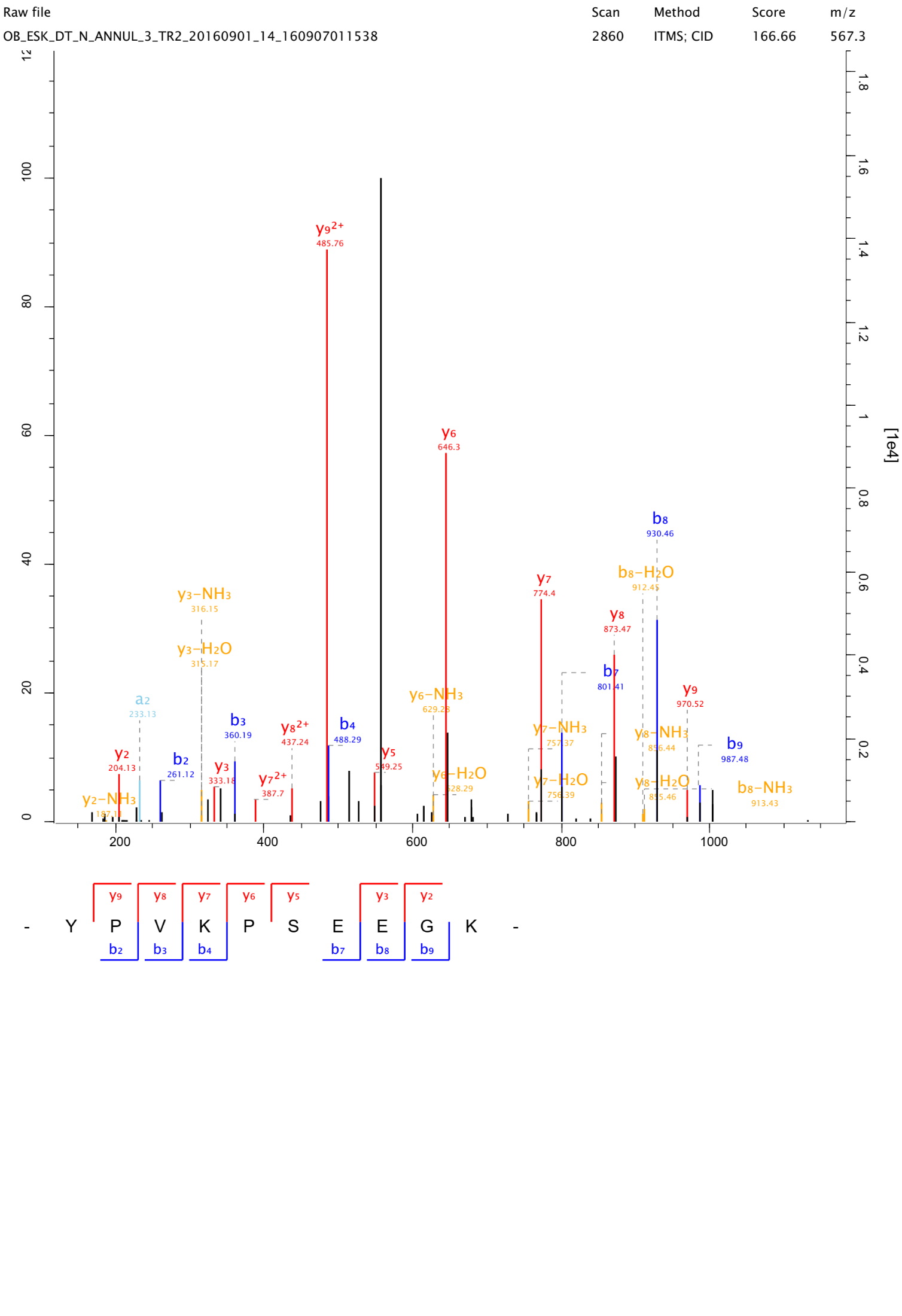


**Protein ID – P25498**

**Protein name:** Acidic phospholipase A2 E OS=Naja oxiana OX=8657 PE=1 SV=1

**Number of Unique Peptides:** 1

**m/z:** 579.28

**MS/MS ID:** 1859

**Score:** 176.6

**Spectrum:** 1/1


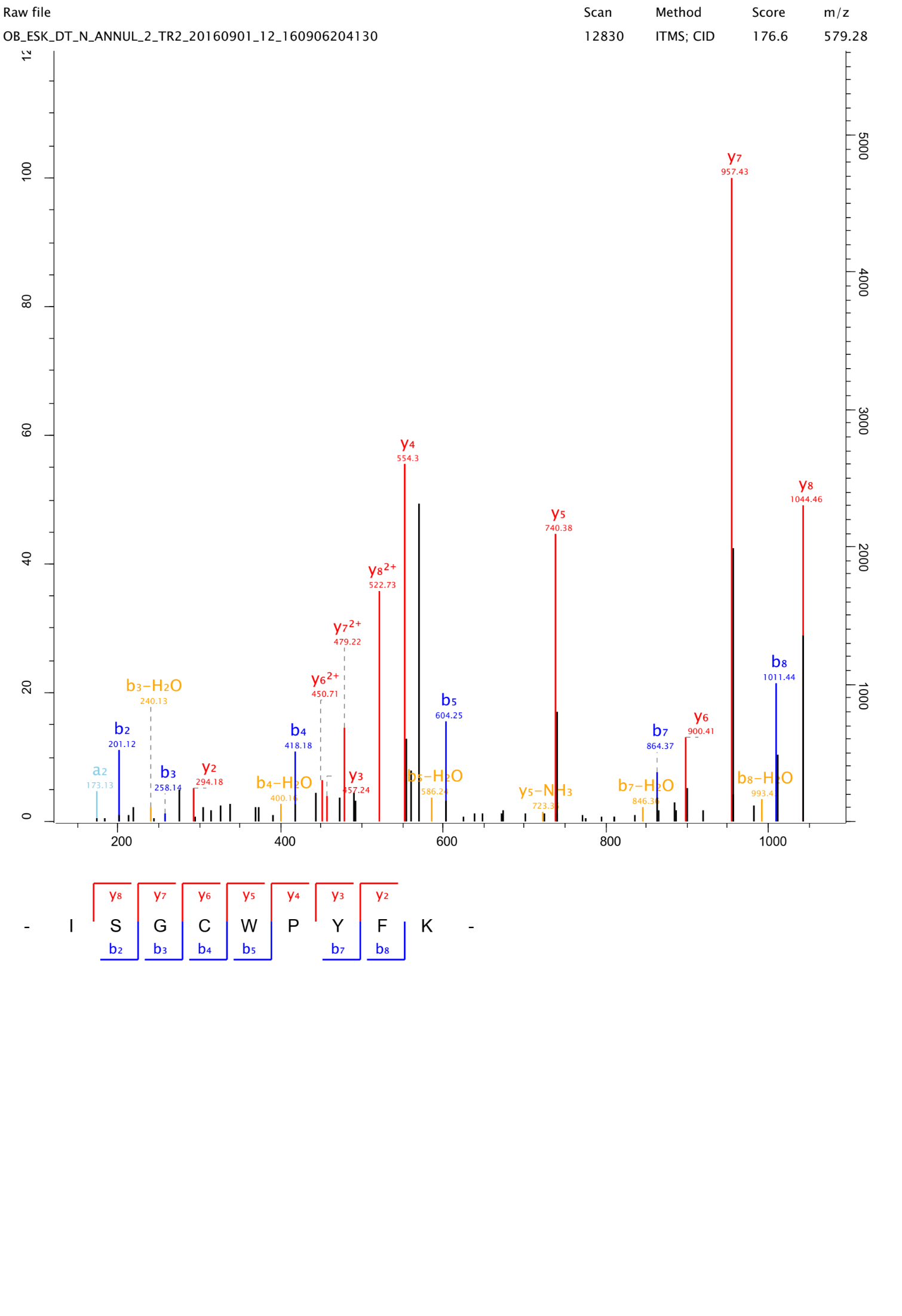


**Protein ID – A8QL57**

**Protein name:** Snake venom serine protease BmSP (Fragment) OS=Bungarus multicinctus OX=8616 PE=2 SV=1

**Number of Unique Peptides:** 2

**m/z:** 811.91

**MS/MS ID:** 719

**Score:** 143.05

**Spectrum:** 1/2


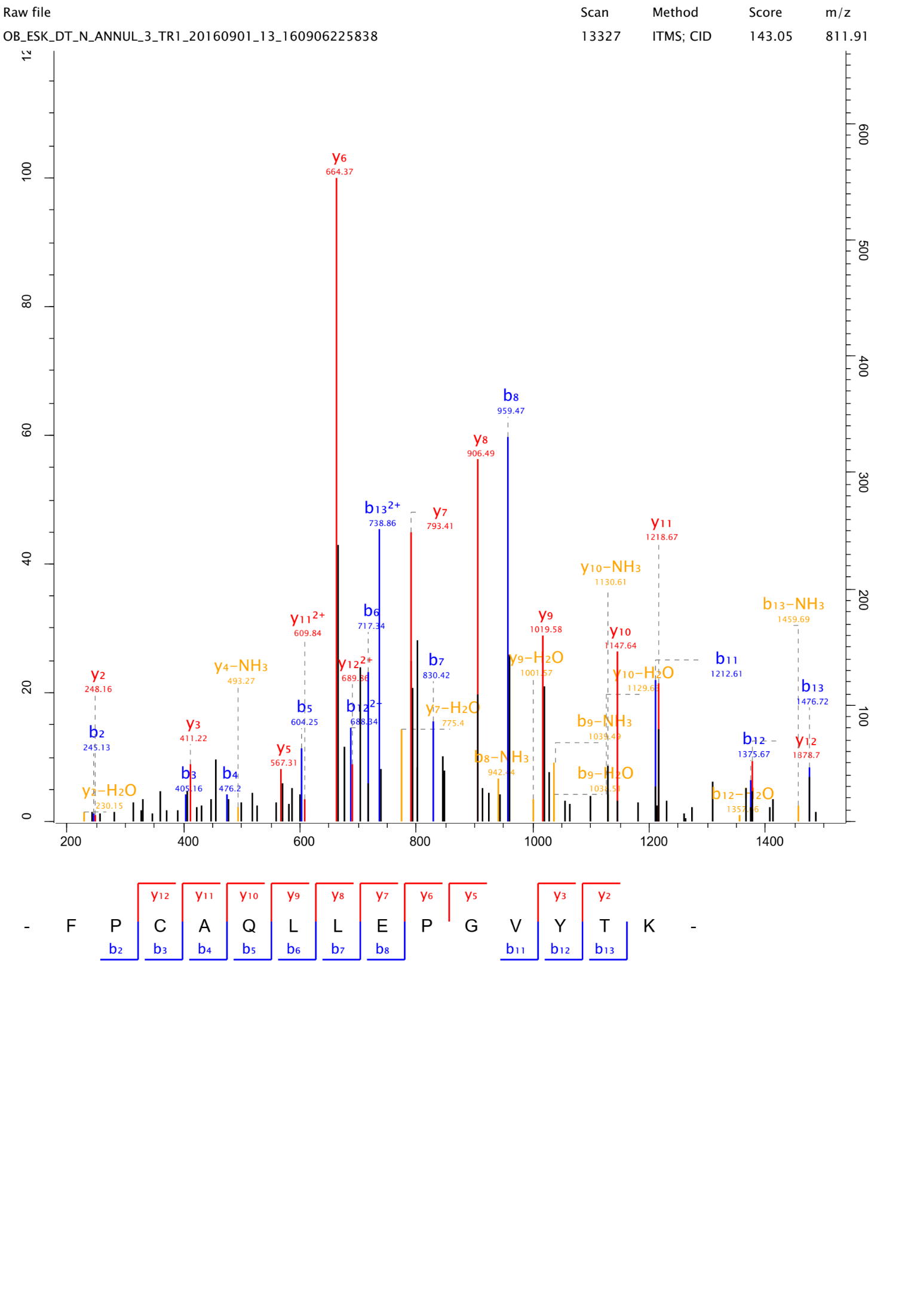


**Protein ID – A8QL57**

**Protein name:** Snake venom serine protease BmSP (Fragment) OS=Bungarus multicinctus OX=8616 PE=2 SV=1

**Number of Unique Peptides:** 2

**m/z:** 568.3

**MS/MS ID:** 2249

**Score:** 76.82

**Spectrum:** 2/2


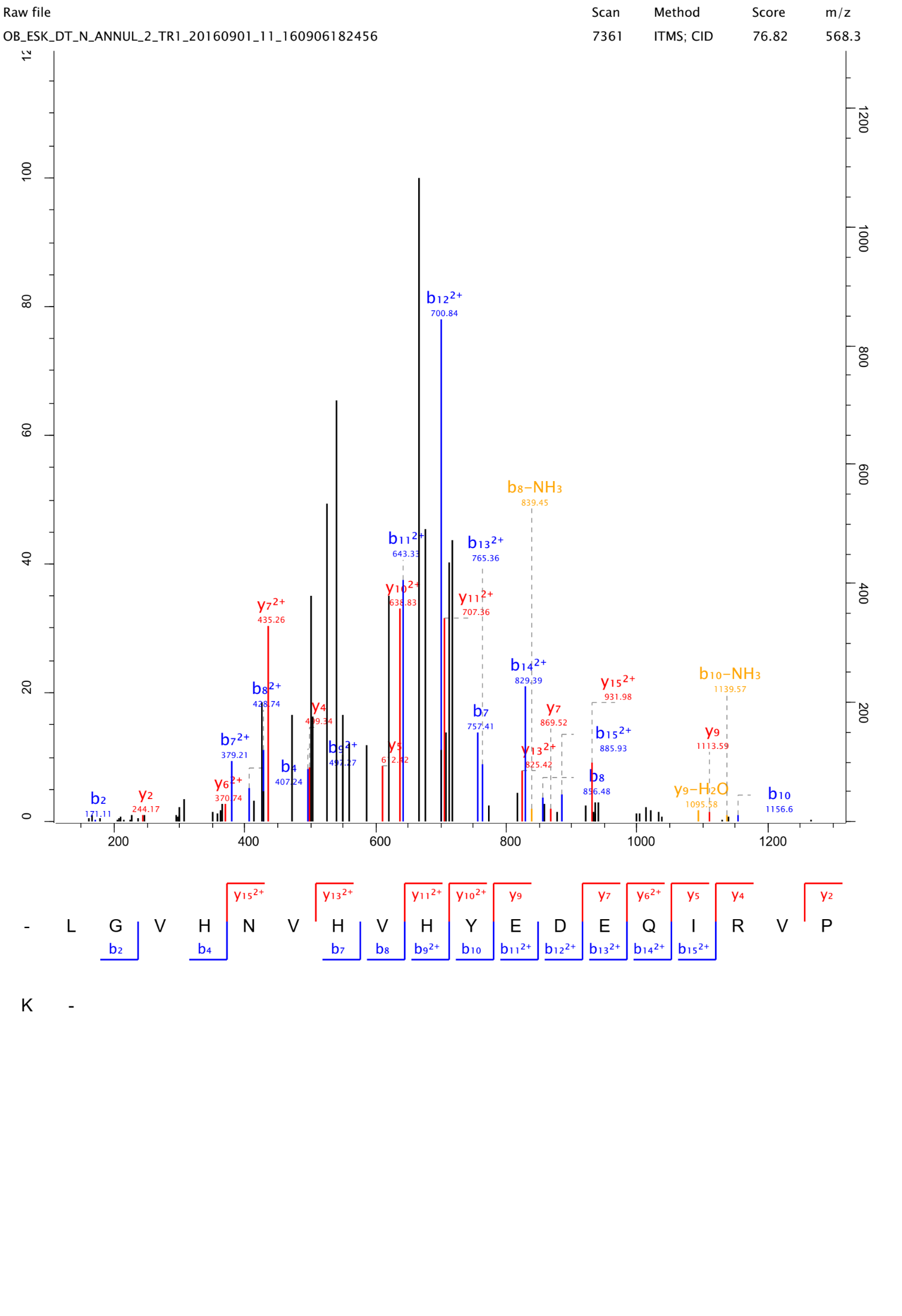


**Protein ID – C1JZW4**

**Protein name:** Opharin OS=Ophiophagus hannah OX=8665 PE=2 SV=1

**Number of Unique Peptides:** 1

**m/z:** 885.13

**MS/MS ID:** 731

**Score:** 85.05

**Spectrum:** 1/1


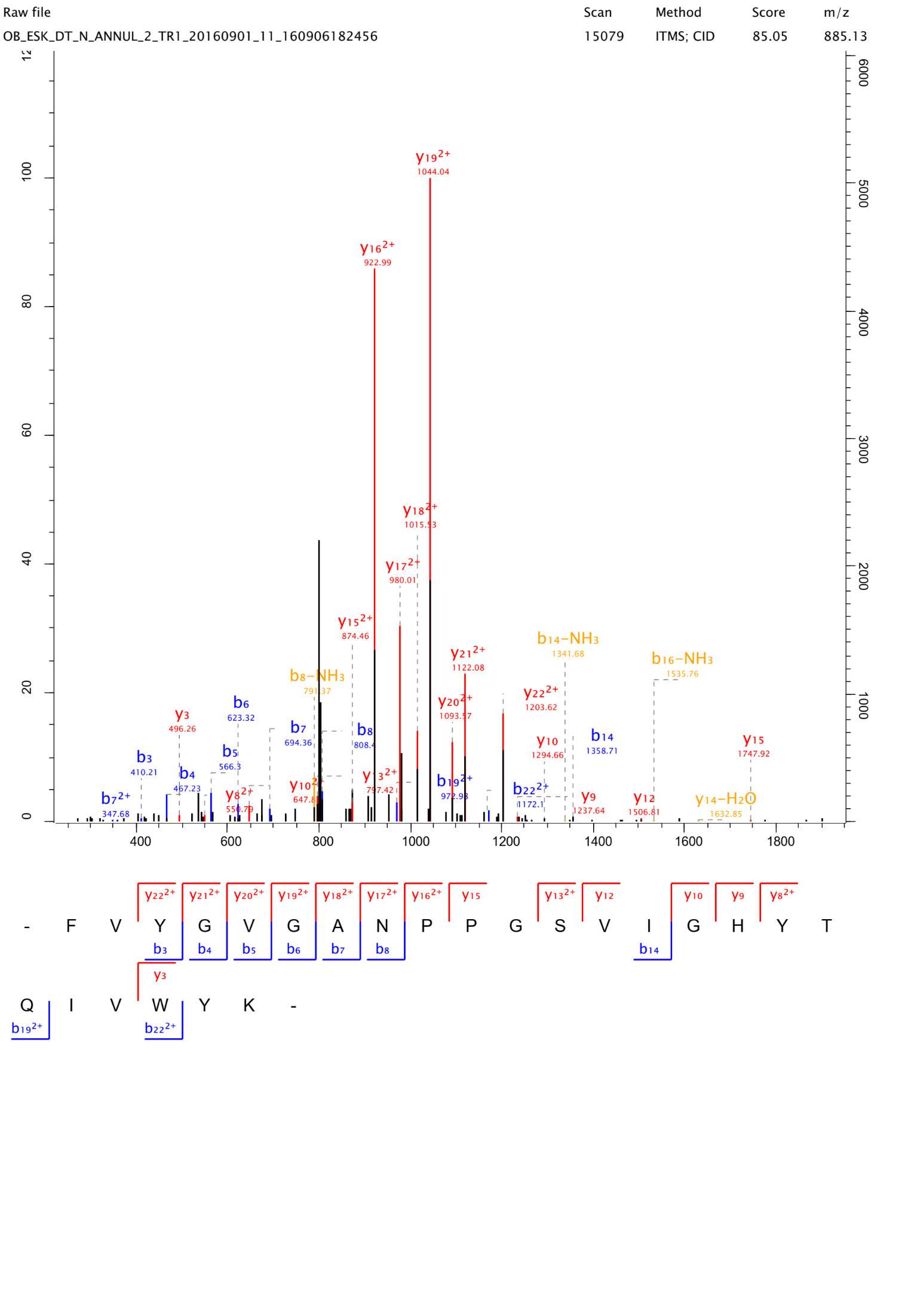


**Protein ID – R4G314**

**Protein name:** LP-Pse-6 OS=Pseudonaja modesta OX=340912 PE=2 SV=1

**Number of Unique Peptides:** 1

**m/z:** 584.77

**MS/MS ID:** 3938

**Score:** 128.38

**Spectrum:** 1/1


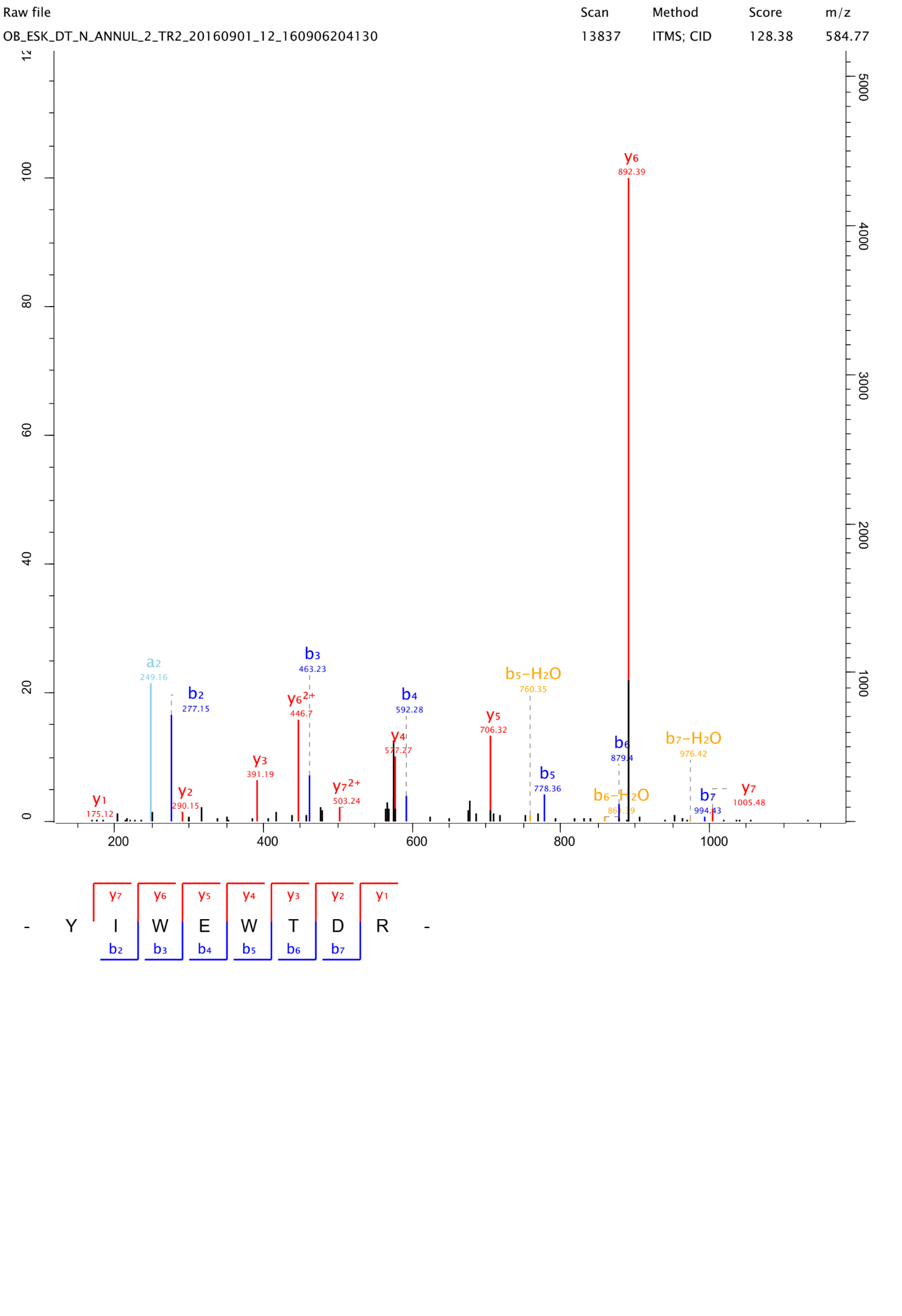


**Protein ID – D6PXE8**

**Protein name:** Zinc metalloproteinase-disintegrin-like atrase-B OS=Naja atra OX=8656 PE=1 SV=1

**Number of Unique Peptides:** 1

**m/z:** 530.62

**MS/MS ID:** 3067

**Score:** 148.13

**Spectrum:** 1/1


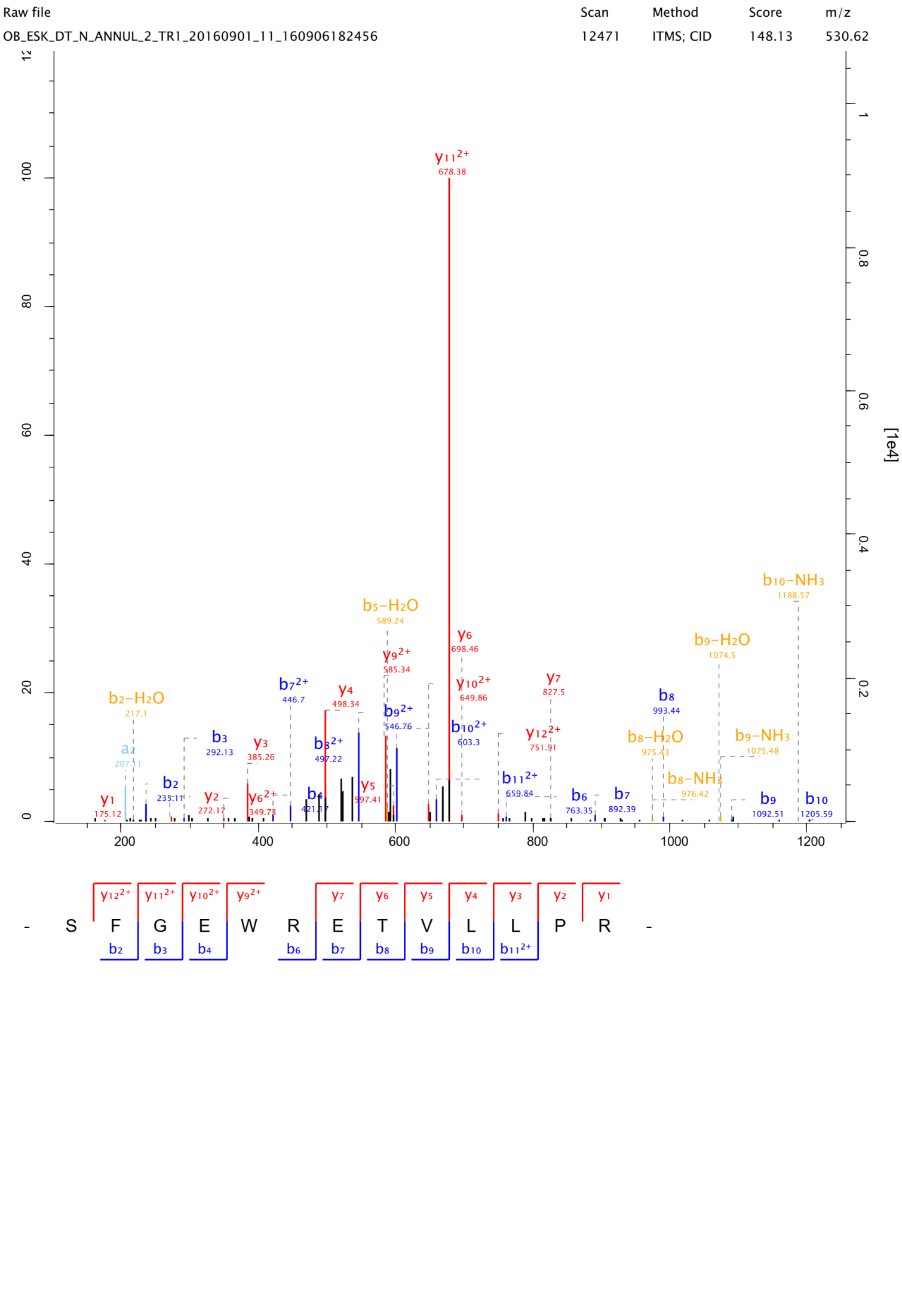


**Protein ID – D3TTC2**

**Protein name:** Zinc metalloproteinase-disintegrin-like atragin OS=Naja atra OX=8656 PE=1 SV=1

**Number of Unique Peptides:** 1

**m/z:** 651.67

**MS/MS ID:** 2116

**Score:** 124.47

**Spectrum:** 1/1


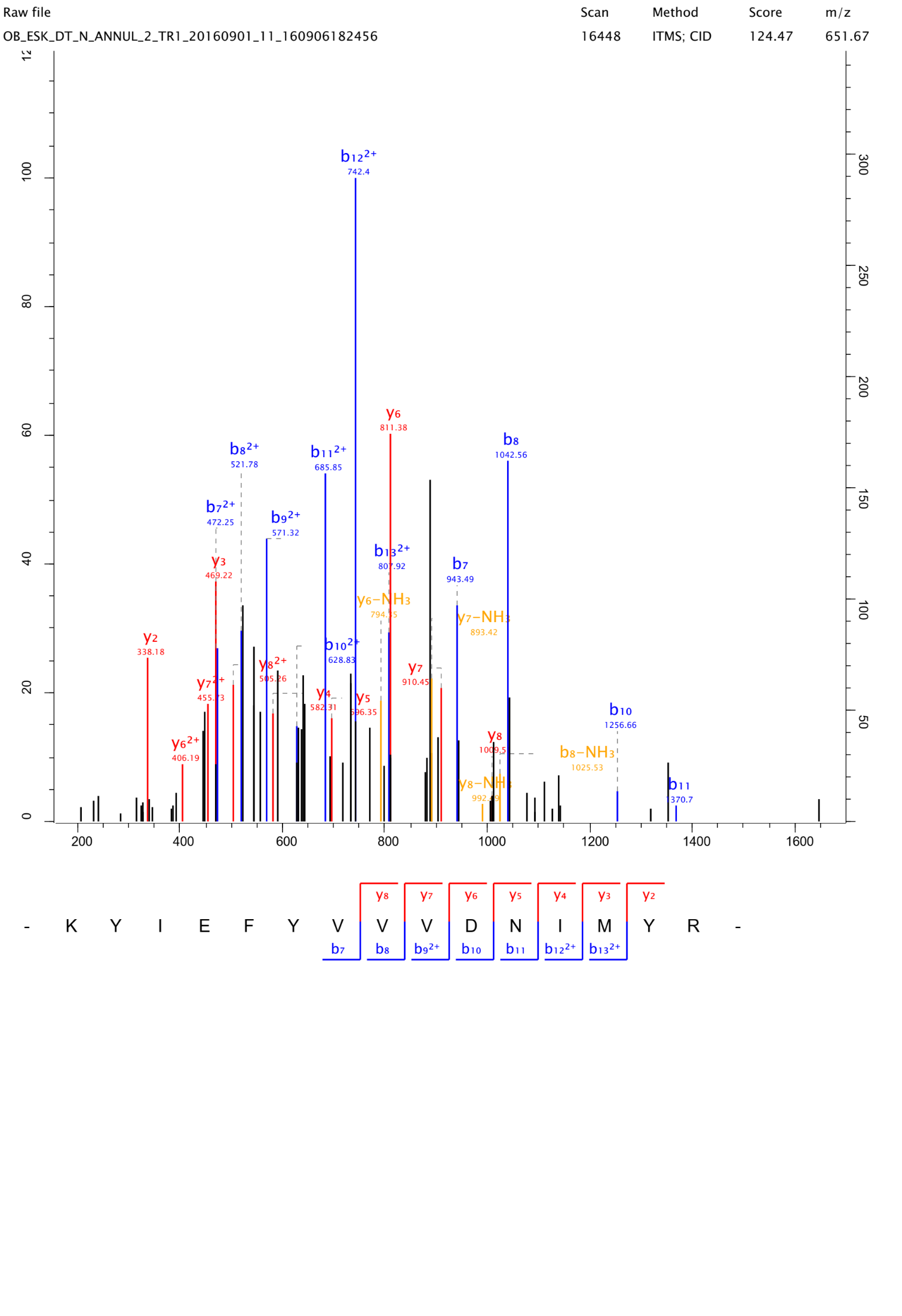


**Protein ID – D5LMJ3**

**Protein name:** Zinc metalloproteinase-disintegrin-like atrase-A OS=Naja atra OX=8656 PE=2 SV=1

**Number of Unique Peptides:** 6

**m/z:** 693.32

**MS/MS ID:** 463

**Score:** 182.92

**Spectrum:** 1/6


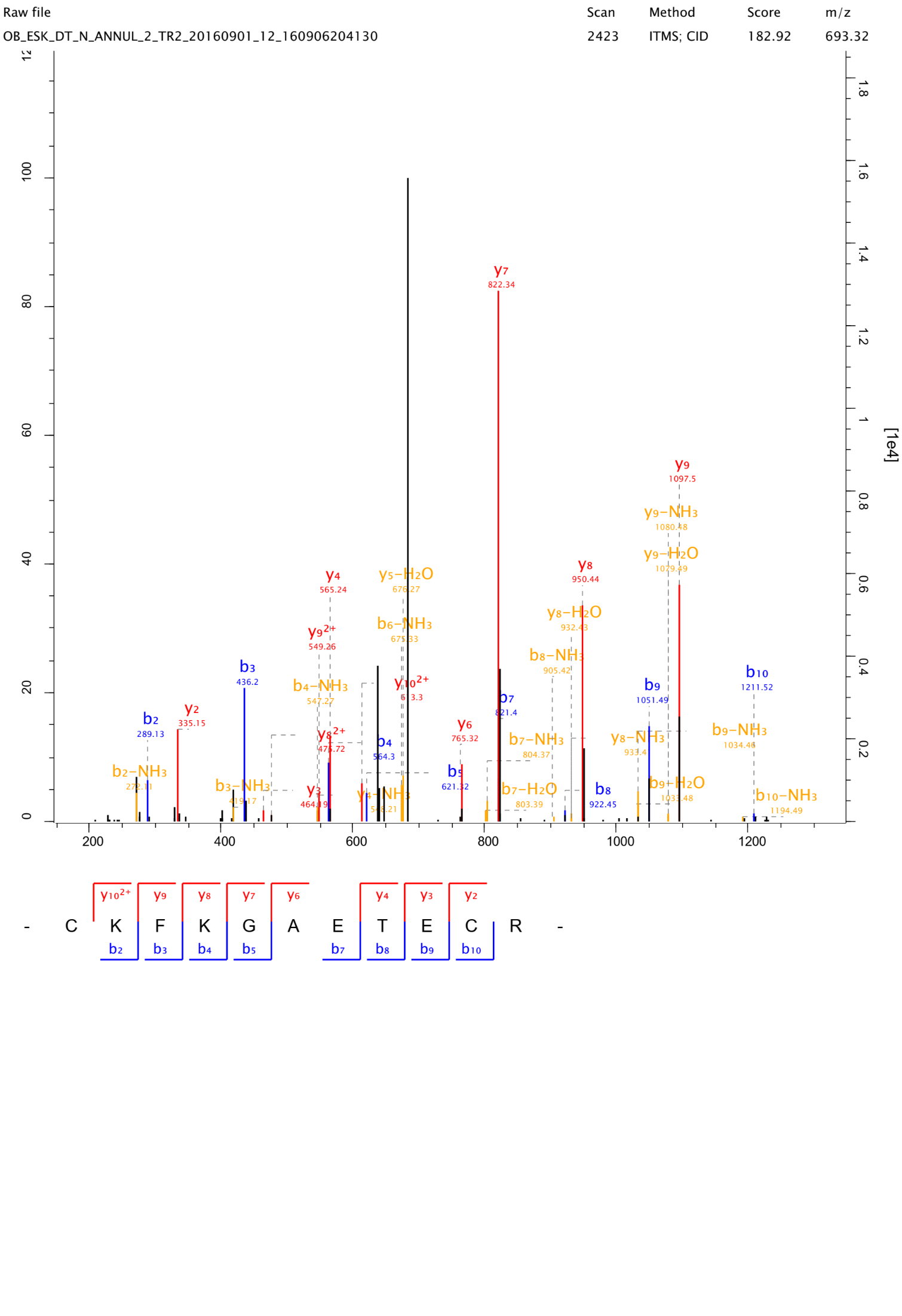


**Protein ID – D5LMJ3**

**Protein name:** Zinc metalloproteinase-disintegrin-like atrase-A OS=Naja atra OX=8656 PE=2 SV=1

**Number of Unique Peptides:** 6

**m/z:** 544.28

**MS/MS ID:** 555

**Score:** 229.33

**Spectrum:** 2/6


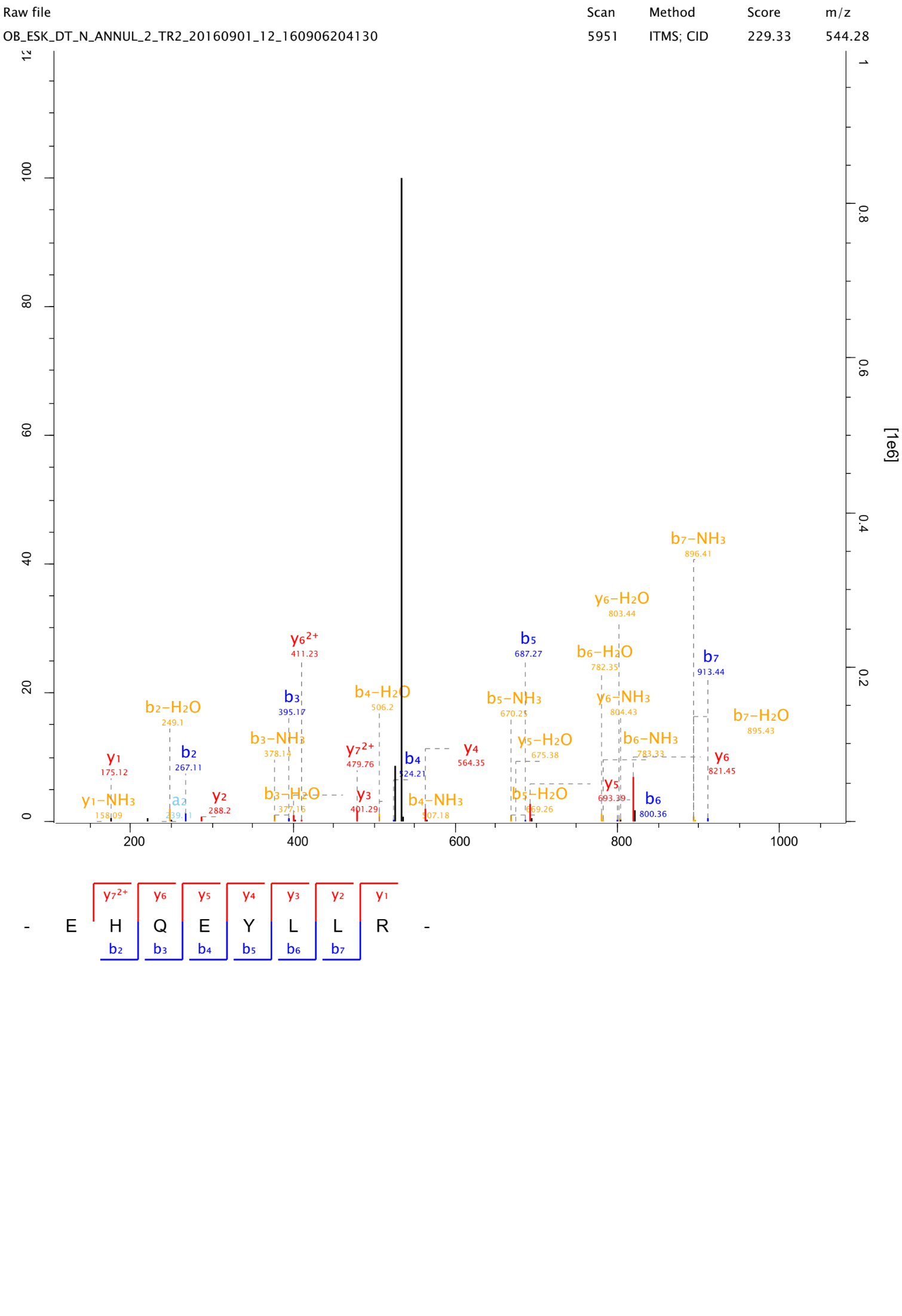


**Protein ID – D5LMJ3**

**Protein name:** Zinc metalloproteinase-disintegrin-like atrase-A OS=Naja atra OX=8656 PE=2 SV=1

**Number of Unique Peptides:** 6

**m/z:** 549.26

**MS/MS ID:** 676

**Score:** 167.09


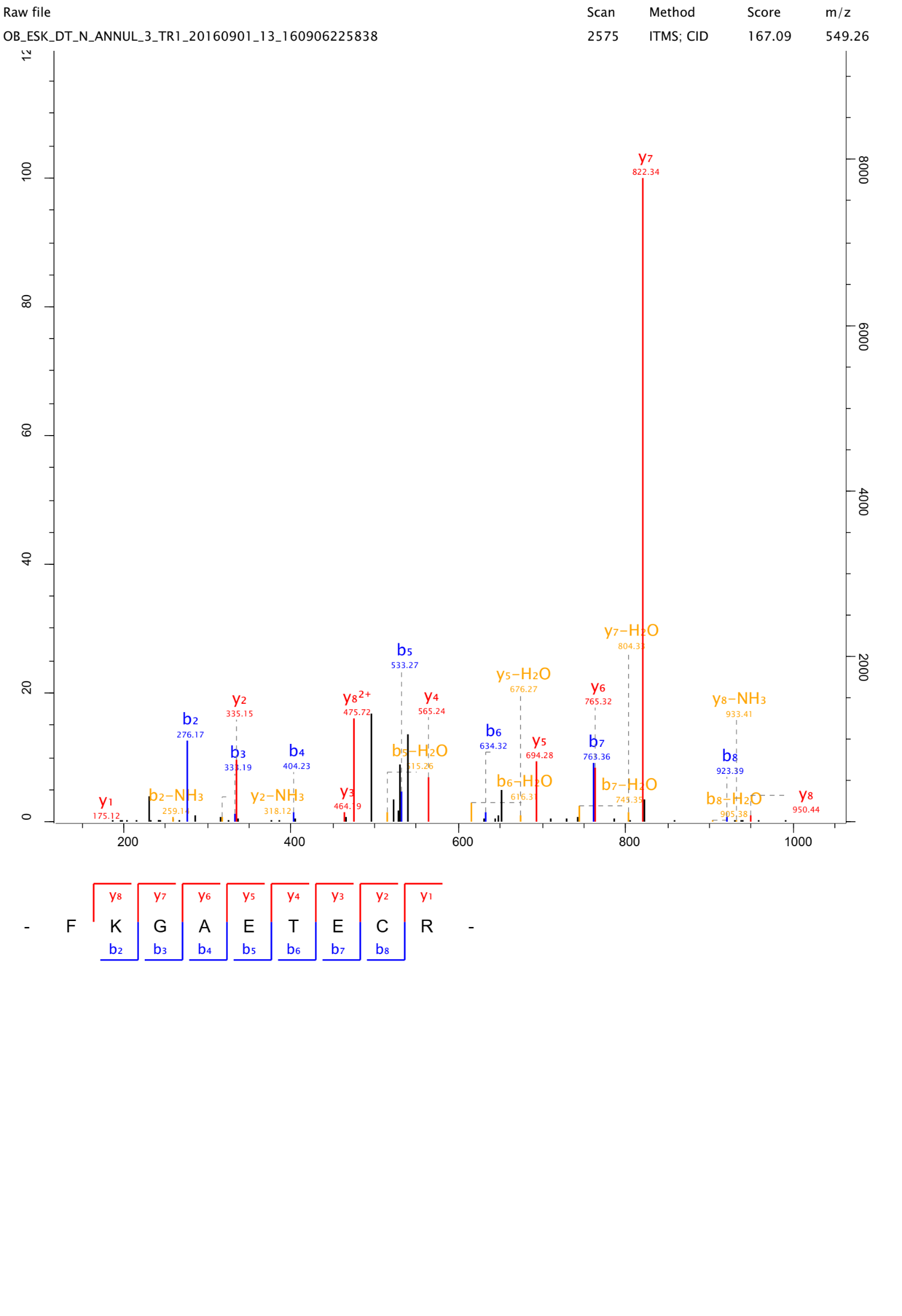
**Spectrum:** 3/6

**Protein ID – D5LMJ3**

**Protein name:** Zinc metalloproteinase-disintegrin-like atrase-A OS=Naja atra OX=8656 PE=2 SV=1

**Number of Unique Peptides:** 6

**m/z:** 749.9

**MS/MS ID:** 594

**Score:** 191.33

**Spectrum:** 4/6


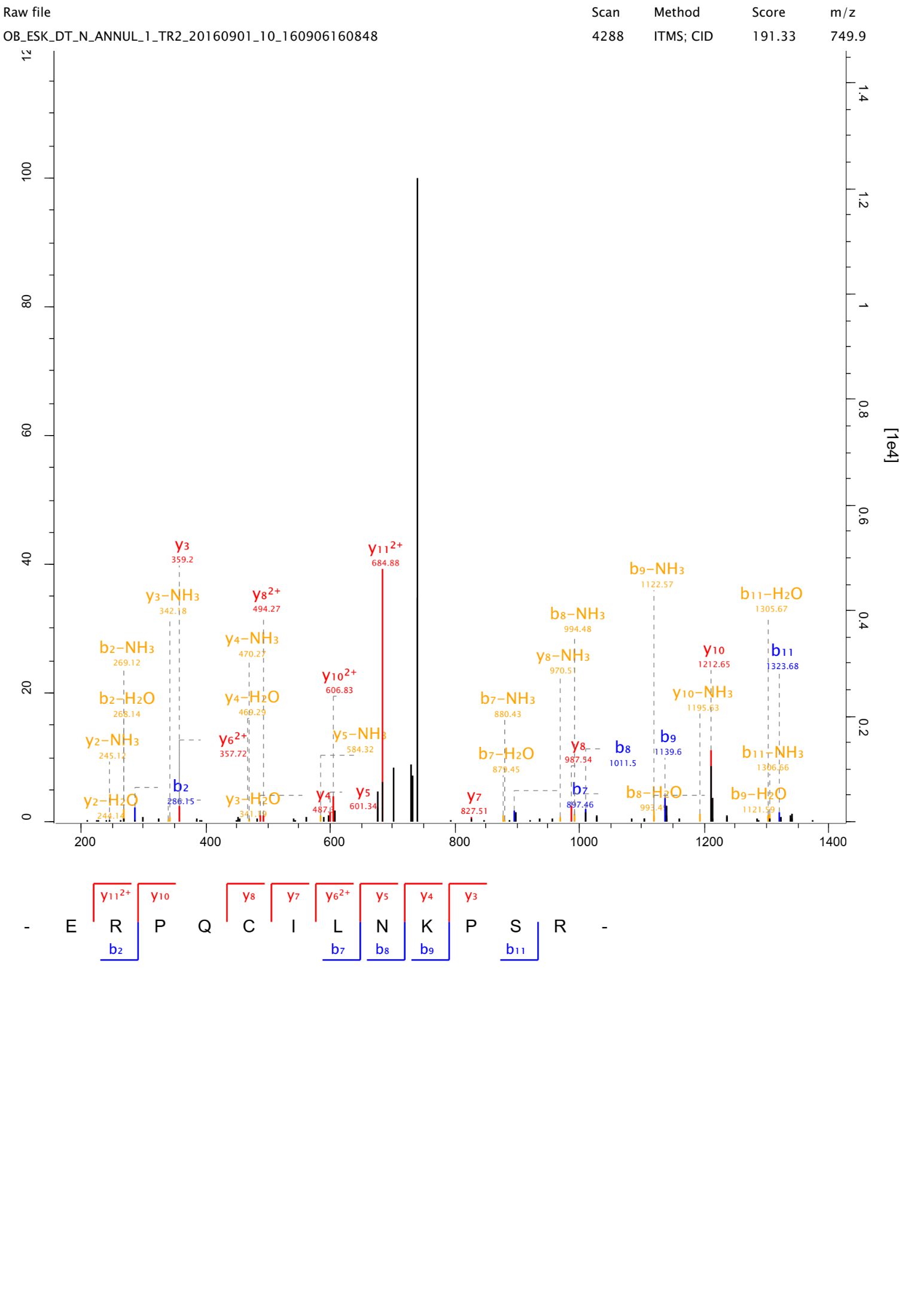


**Protein ID – D5LMJ3**

**Protein name:** Zinc metalloproteinase-disintegrin-like atrase-A OS=Naja atra OX=8656 PE=2 SV=1

**Number of Unique Peptides:** 6

**m/z:** 945.87

**MS/MS ID:** 2453

**Score:** 204.17

**Spectrum:** 5/6


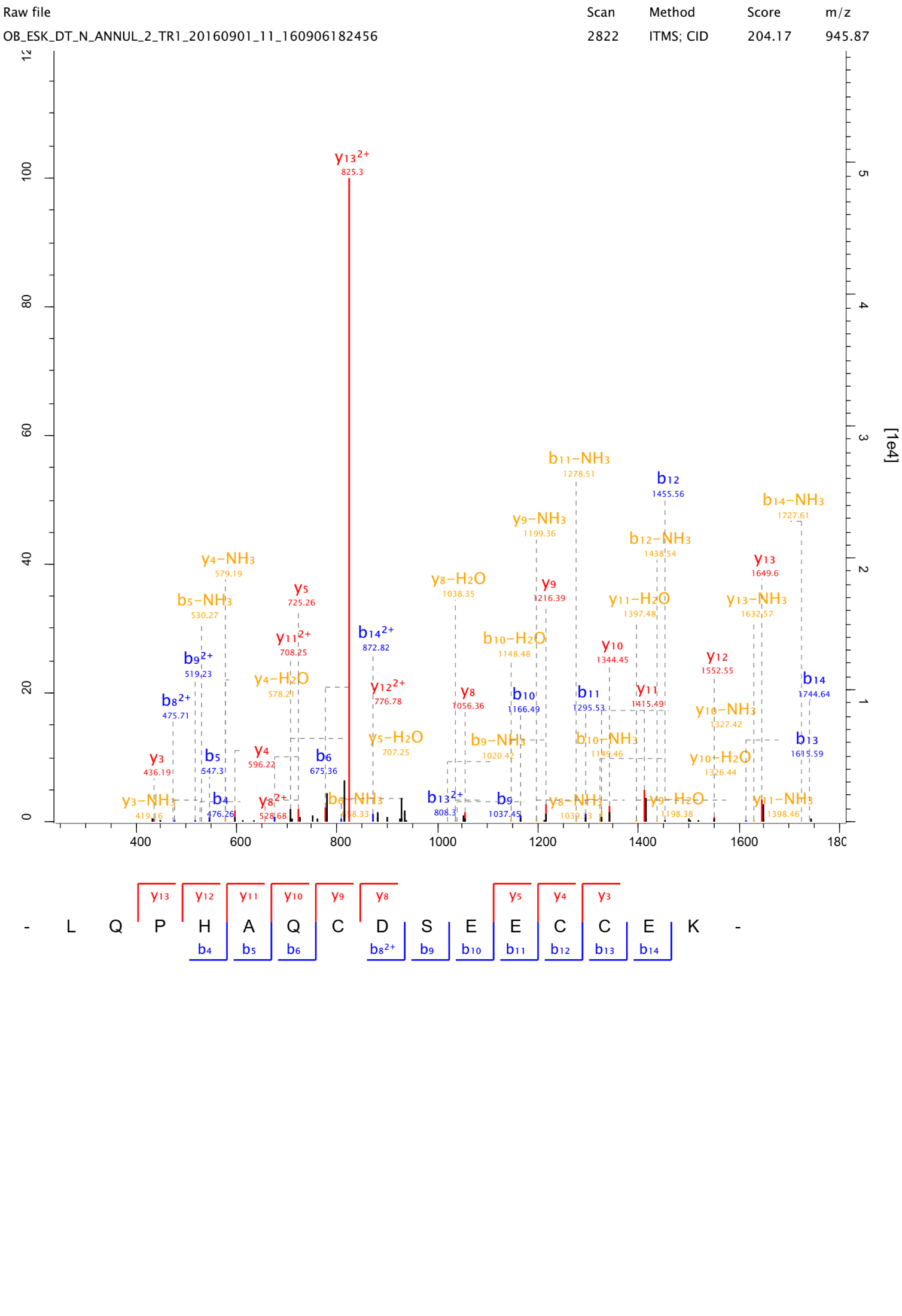


**Protein ID – D5LMJ3**

**Protein name:** Zinc metalloproteinase-disintegrin-like atrase-A OS=Naja atra OX=8656 PE=2 SV=1

**Number of Unique Peptides:** 6

**m/z:** 1090.44

**MS/MS ID:** 2468

**Score:** 159.44

**Spectrum:** 6/6


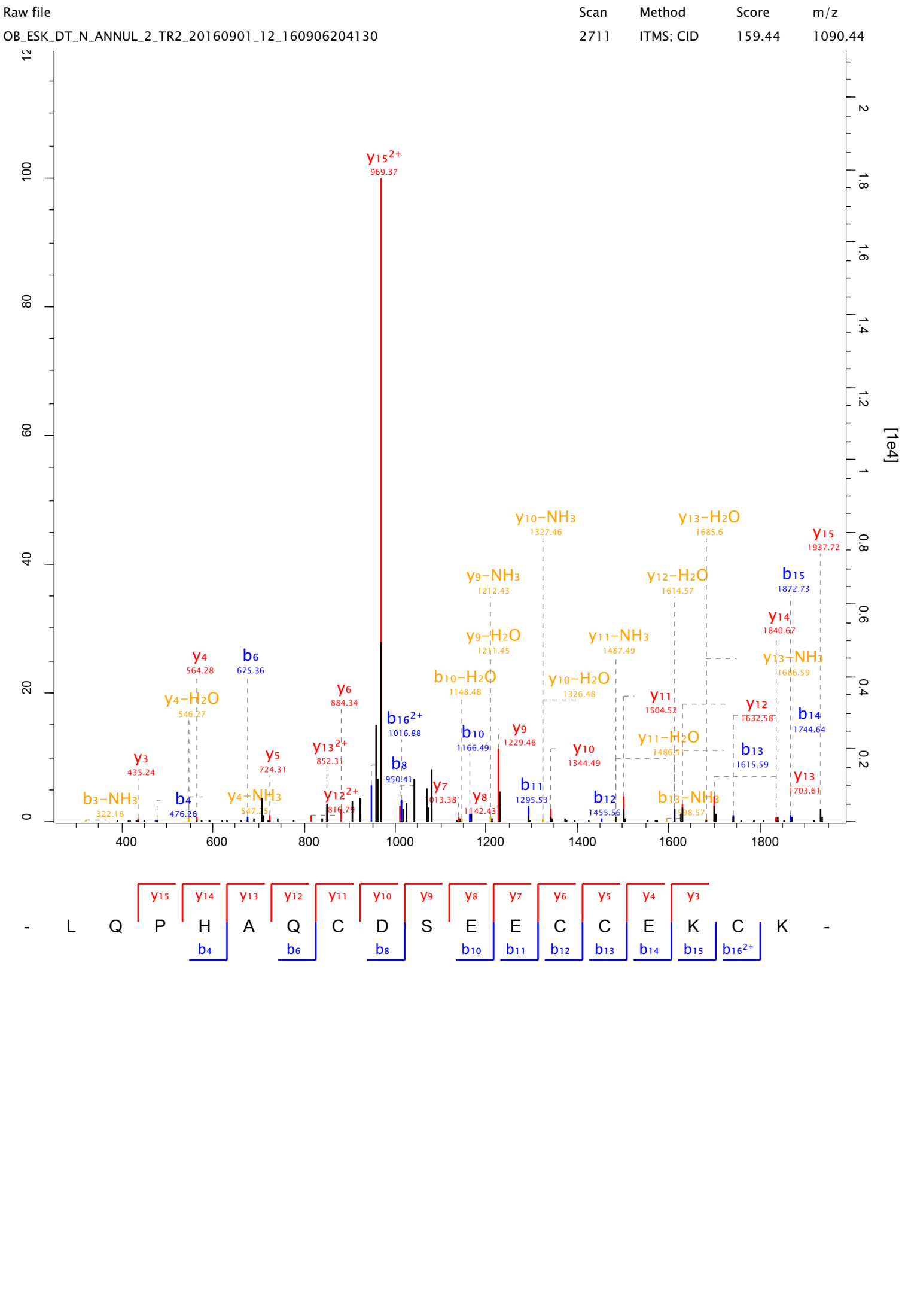


**Protein ID – U3FVL3**

**Protein name:** Vesicular integral-membrane protein VIP36 OS=Micrurus fulvius OX=8637 PE=2 SV=1

**Number of Unique Peptides:** 1

**m/z:** 519.25

**MS/MS ID:** 2805

**Score:** 110.53

**Spectrum:** 1/1


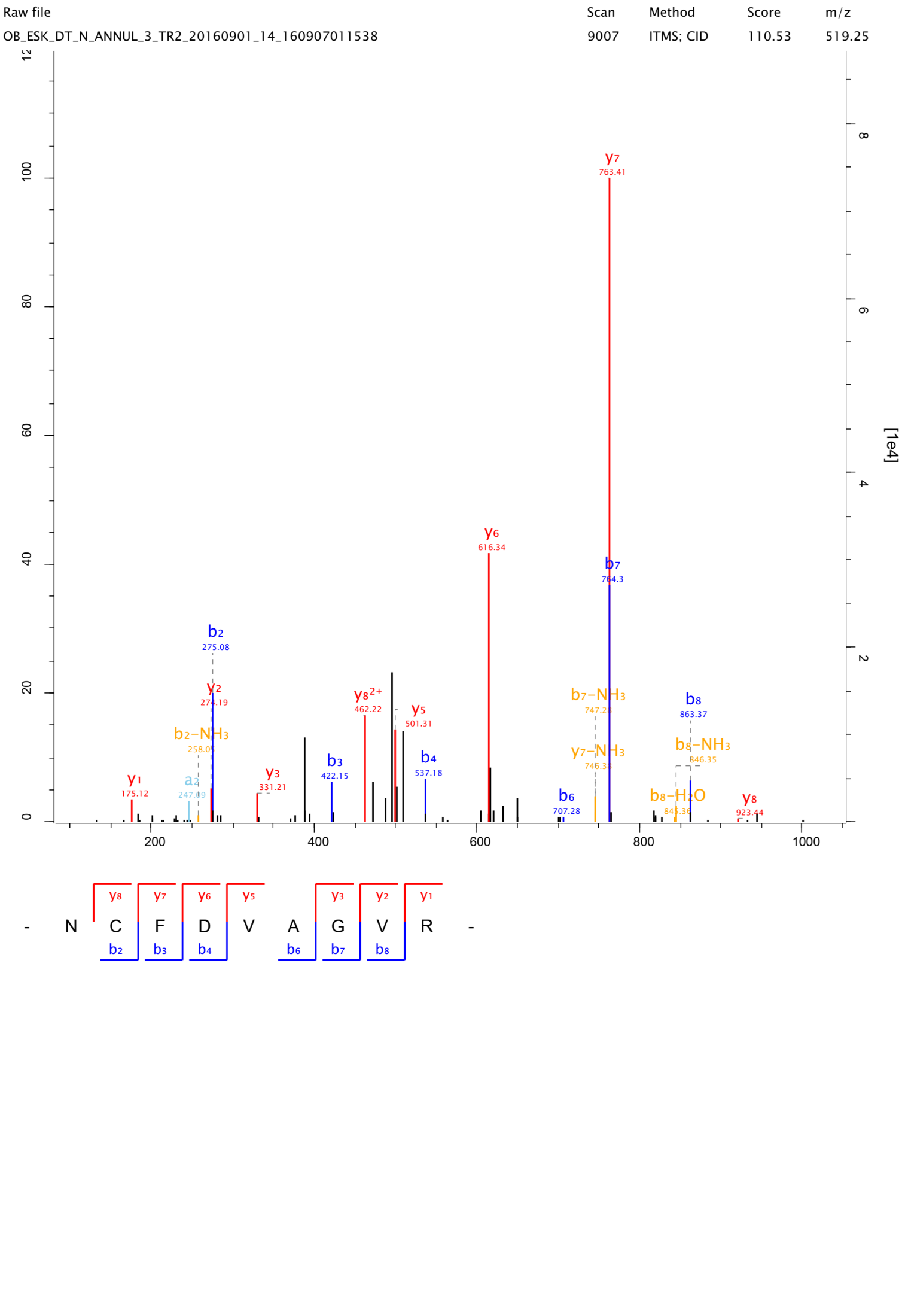


**Protein ID – P00600**

**Protein name:** Acidic phospholipase A2 DE-II OS=Naja melanoleuca OX=8643 PE=1 SV=1

**Number of Unique Peptides:** 1

**m/z:** 723.06

**MS/MS ID:** 316

**Score:** 70.02

**Spectrum:** 1/1


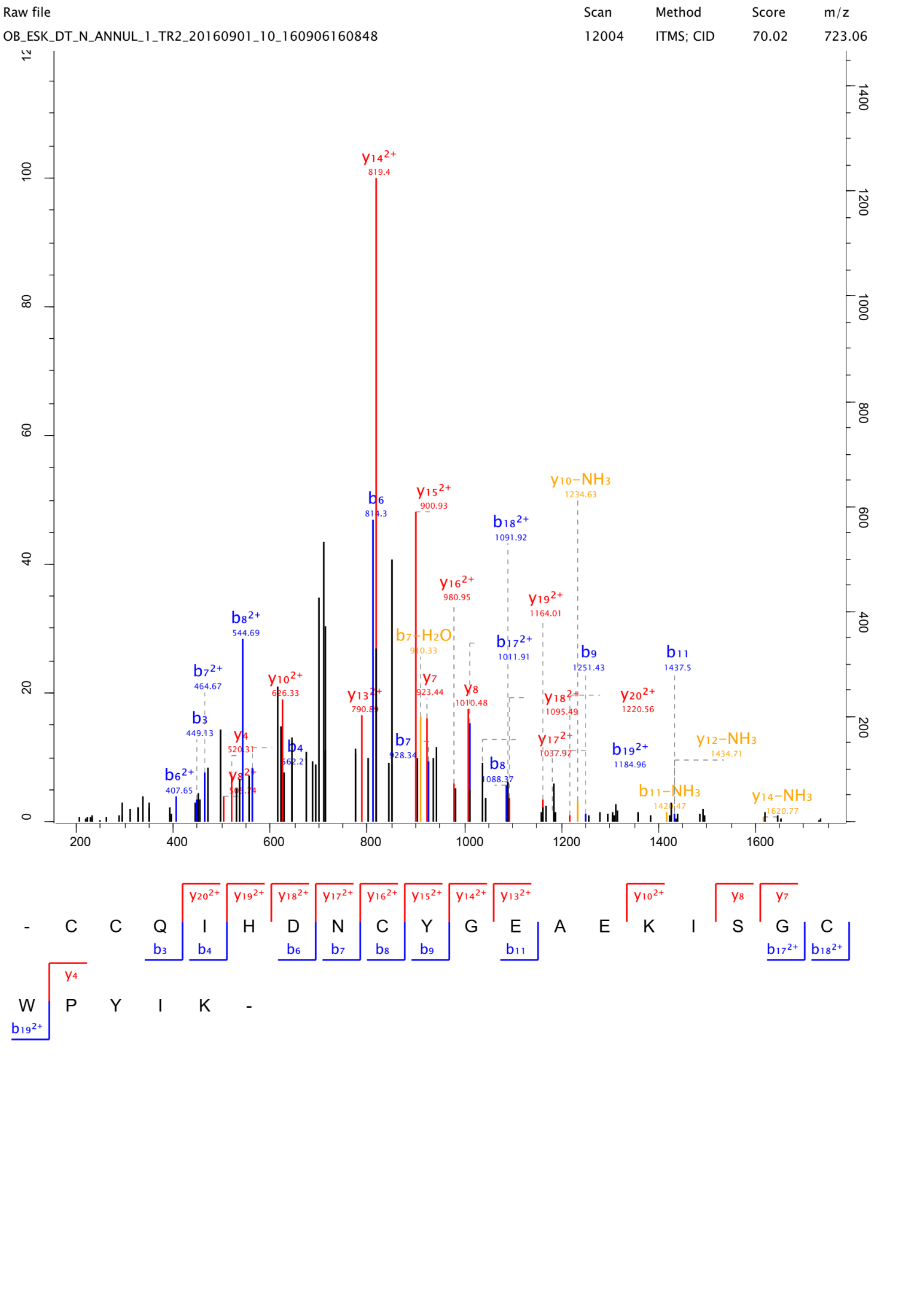


**Protein ID – P00605**

**Protein name:** Phospholipase A2 basic OS=Naja nigricollis OX=8654 PE=1 SV=1

**Number of Unique Peptides:** 1

**m/z:** 768.58

**MS/MS ID:** 2842

**Score:** 52.09

**Spectrum:** 1/1


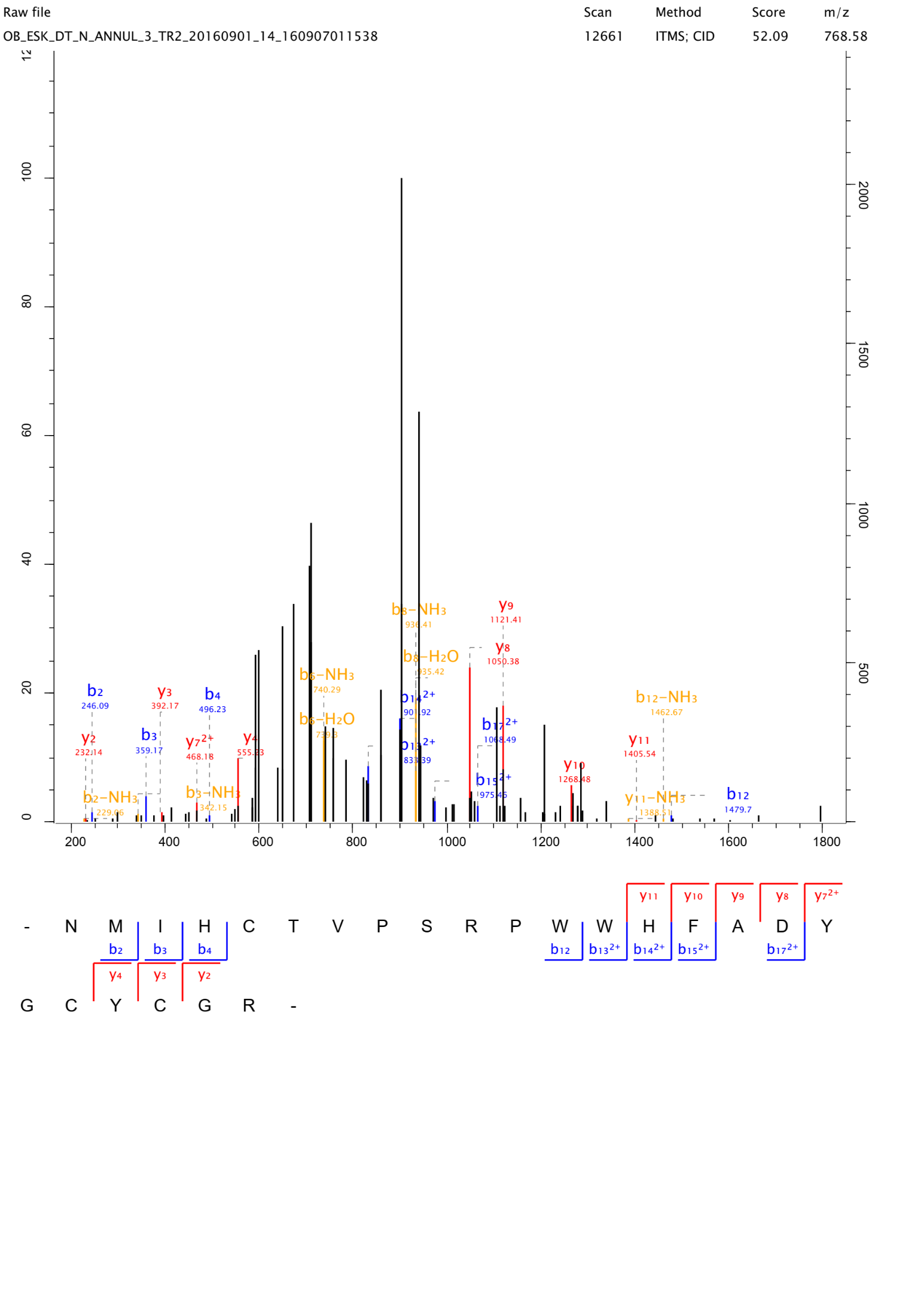


**Protein ID – P00986**

**Protein name:** Kunitz-type serine protease inhibitor 2 OS=Naja nivea OX=8655 PE=1 SV=1

**Number of Unique Peptides:** 4

**m/z:** 712.83

**MS/MS ID:** 626

**Score:** 216.91

**Spectrum:** 1/4


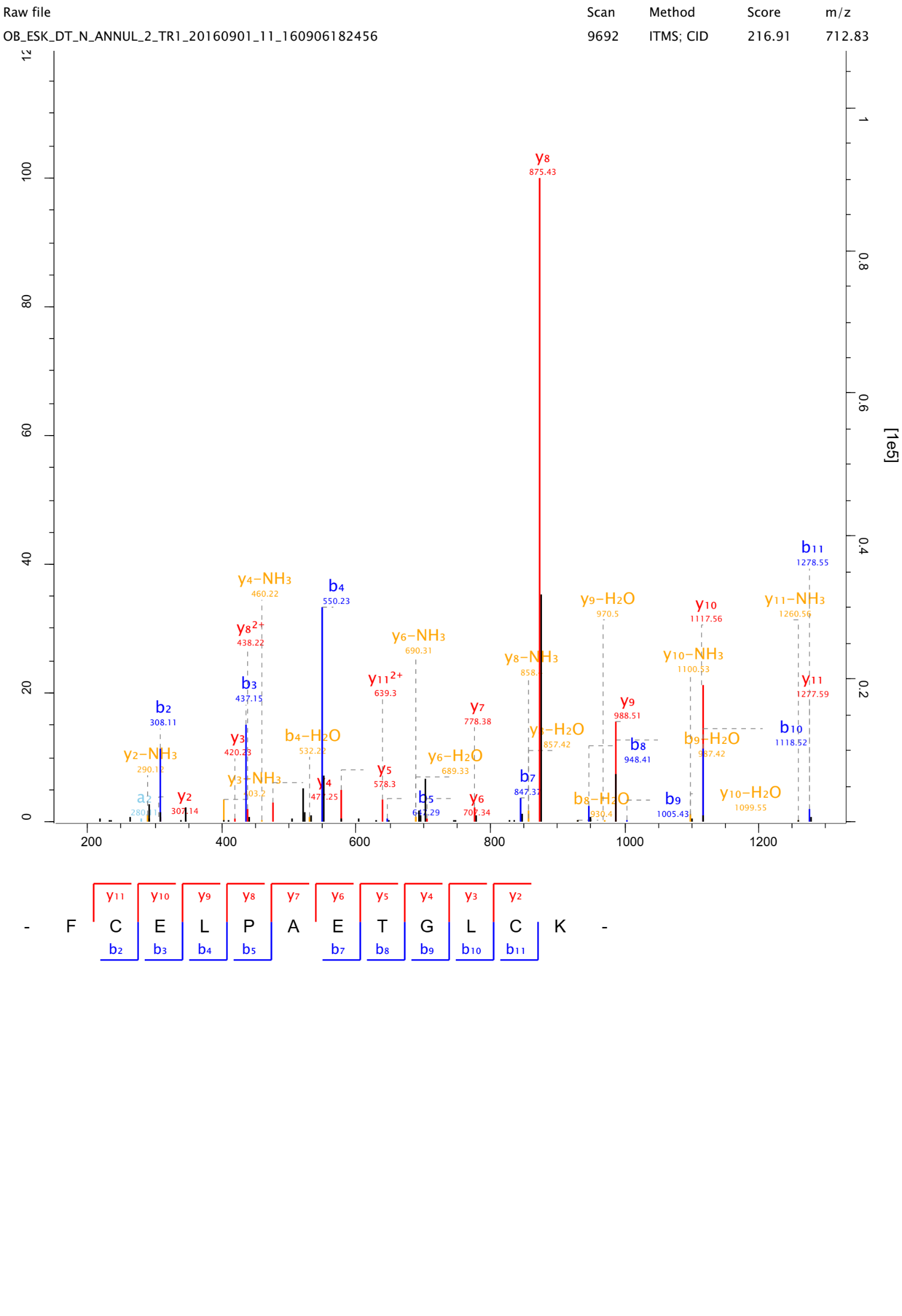


**Protein ID – P00986**

**Protein name:** Kunitz-type serine protease inhibitor 2 OS=Naja nivea OX=8655 PE=1 SV=1

**Number of Unique Peptides:** 4

**m/z:** 674.29

**MS/MS ID:** 3480

**Score:** 175.33

**Spectrum:** 2/4


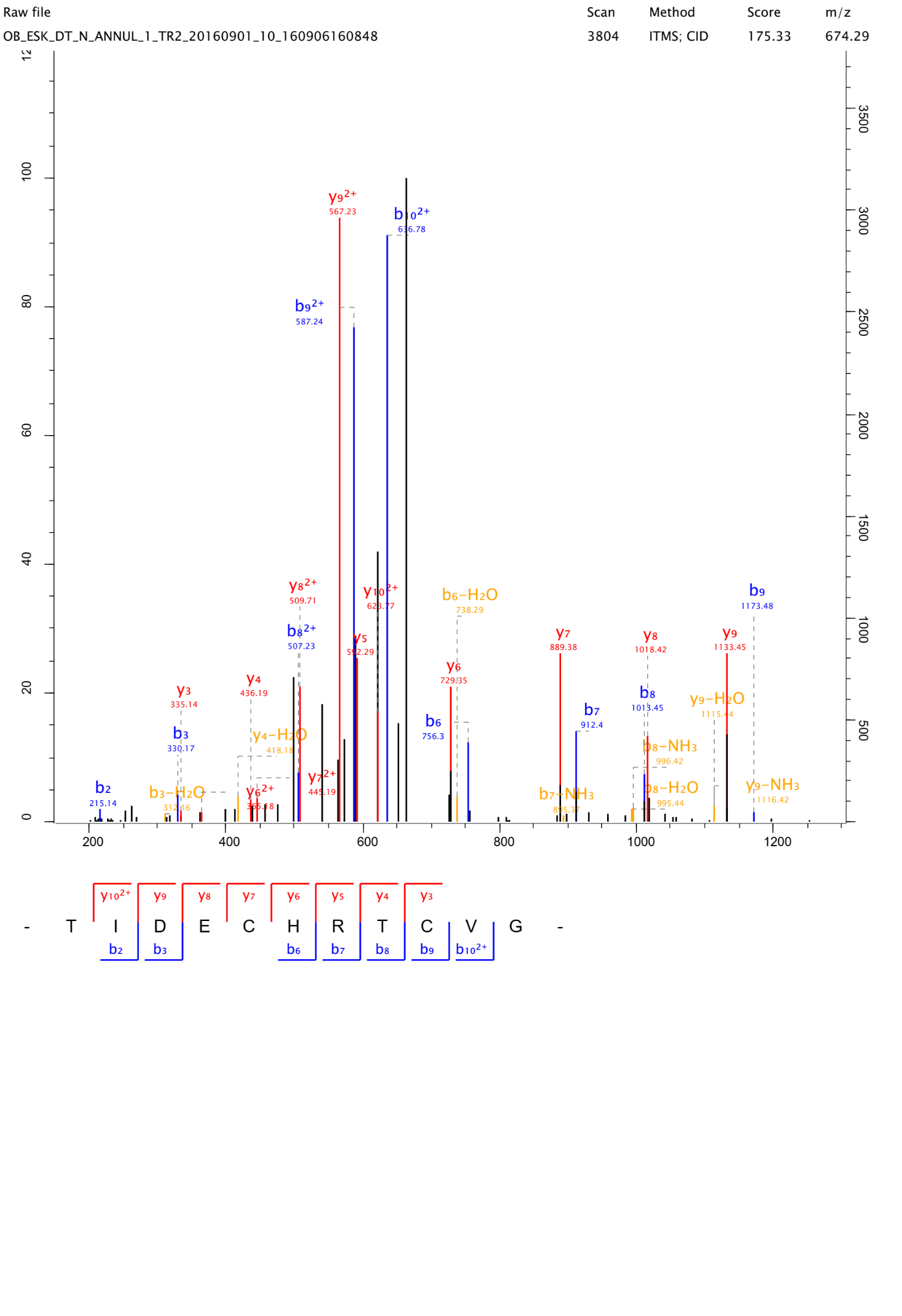


**Protein ID – P00986**

**Protein name:** Kunitz-type serine protease inhibitor 2 OS=Naja nivea OX=8655 PE=1 SV=1

**Number of Unique Peptides:** 4

**m/z:** 541.92

**MS/MS ID:** 695

**Score:** 97.78

**Spectrum:** 3/4


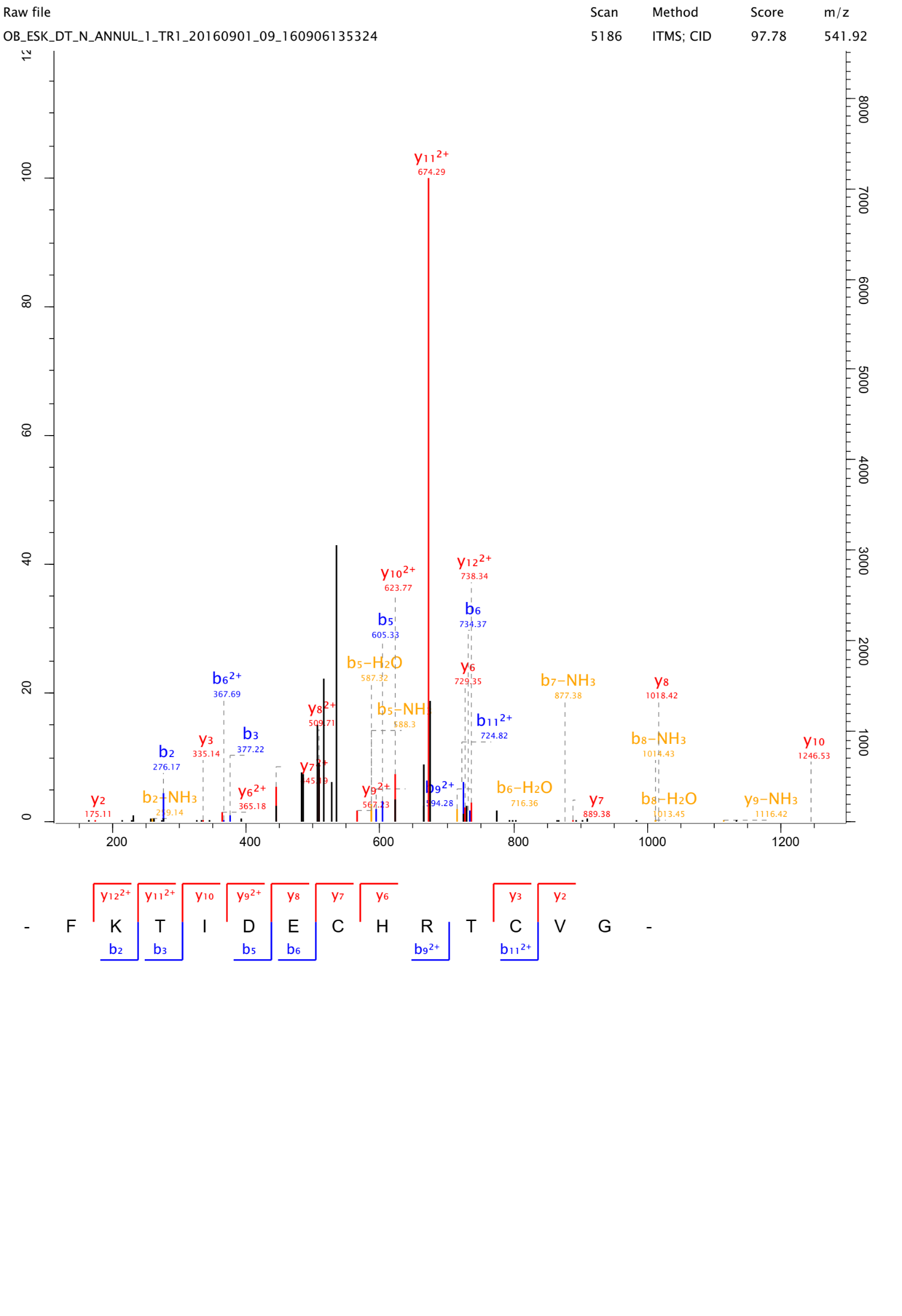


**Protein ID – P00986**

**Protein name:** Kunitz-type serine protease inhibitor 2 OS=Naja nivea OX=8655 PE=1 SV=1

**Number of Unique Peptides:** 4

**m/z:** 603.29

**MS/MS ID:** 686

**Score:** 204.79

**Spectrum:** 4/4


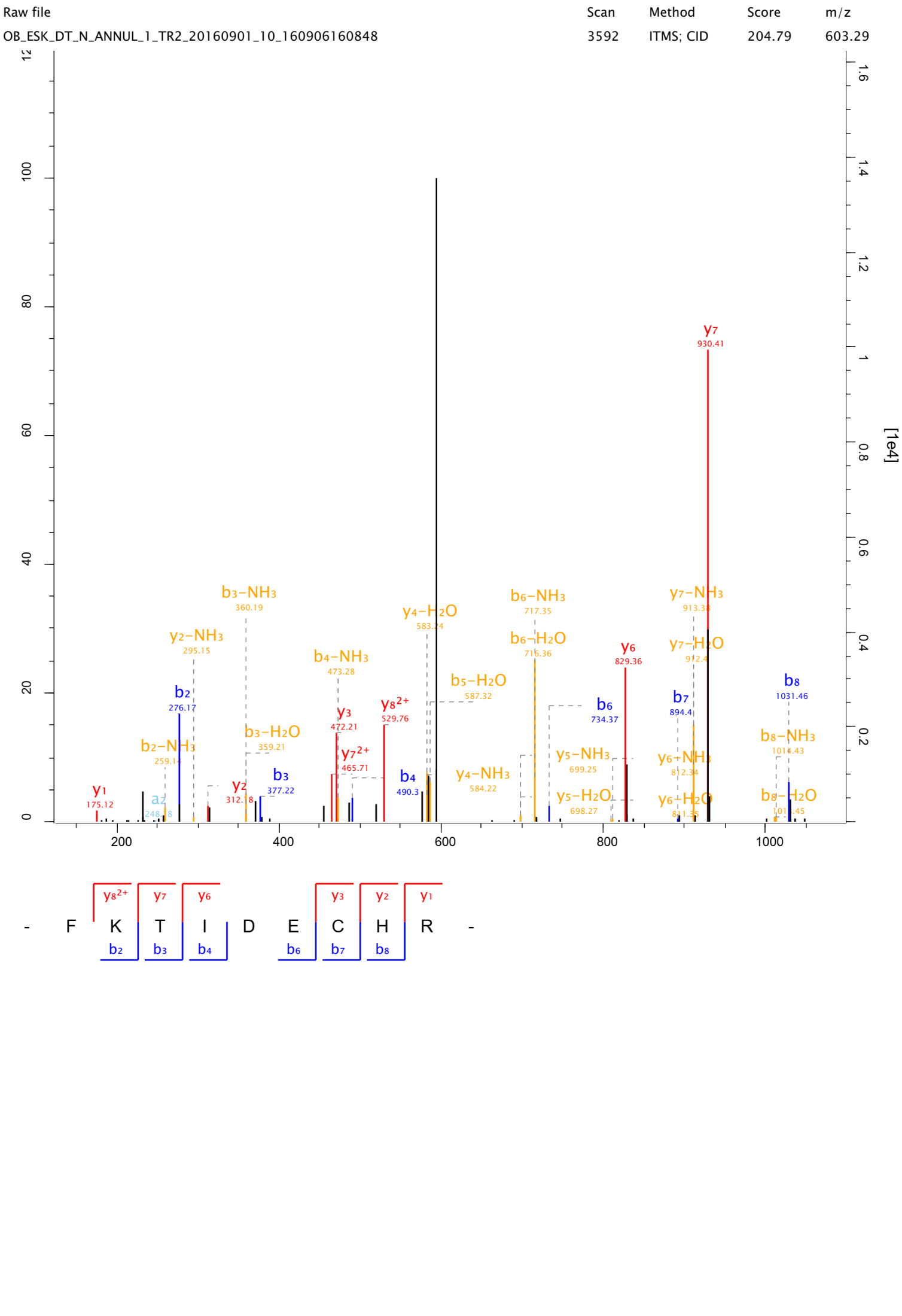


**Protein ID – P01388**

**Protein name:** Long neurotoxin 2 OS=Naja melanoleuca OX=8643 PE=1 SV=1

**Number of Unique Peptides:** 2

**m/z:** 658.76

**MS/MS ID:** 3511

**Score:** 175.74

**Spectrum:** 1/2


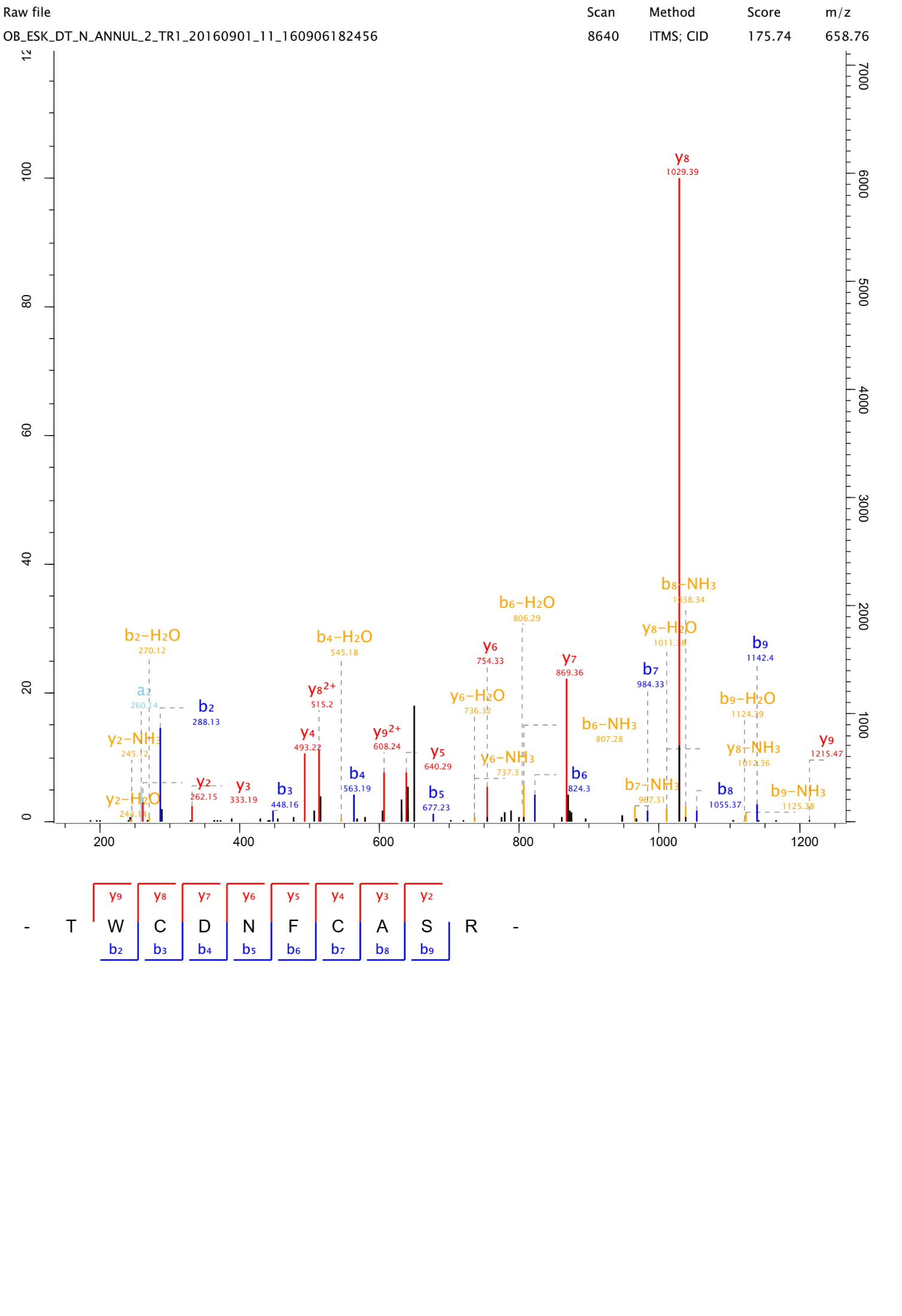


**Protein ID – P01388**

**Protein name:** Long neurotoxin 2 OS=Naja melanoleuca OX=8643 PE=1 SV=1

**Number of Unique Peptides:** 2

**m/z:** 667.68

**MS/MS ID:** 3539

**Score:** 92.95

**Spectrum:** 2/2


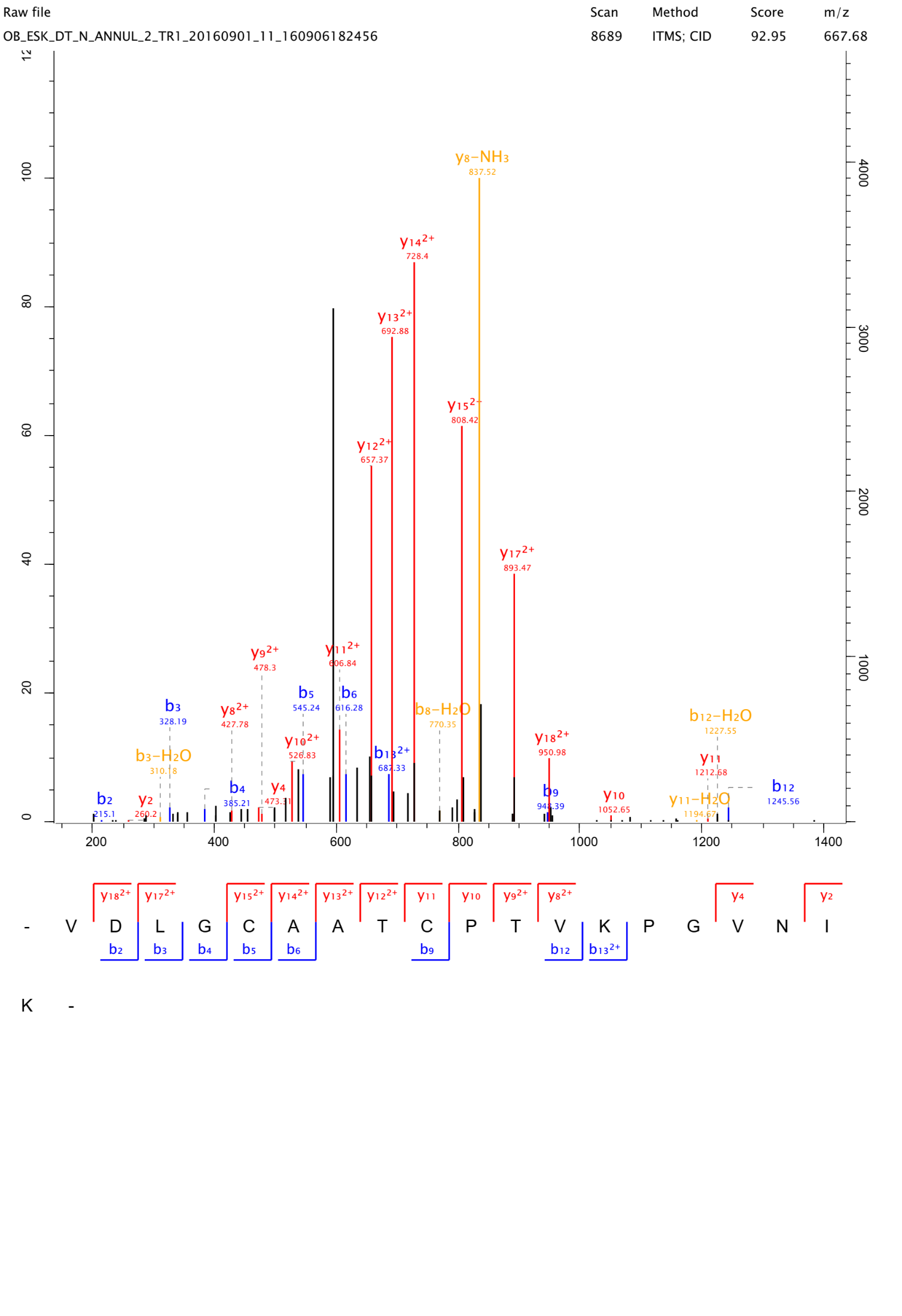


**Protein ID – P01389**

**Protein name:** Long neurotoxin 1 OS=Naja anchietae OX=263737 PE=1 SV=1

**Number of Unique Peptides:** 1

**m/z:** 949.44

**MS/MS ID:** 1845

**Score:** 86.18

**Spectrum:** 1/1


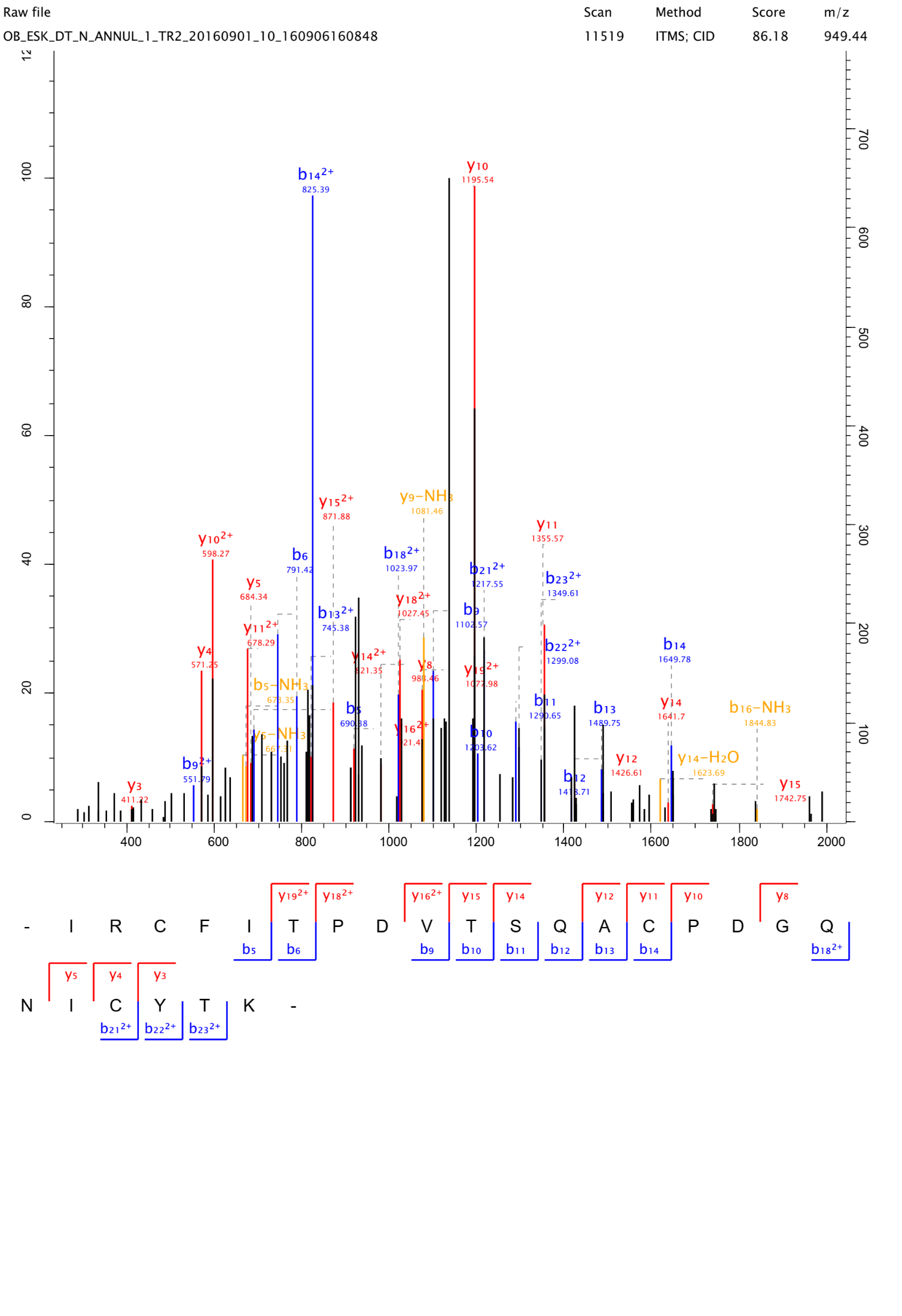


**Protein ID – P01399**

**Protein name:** Weak toxin CM-13b OS=Naja annulifera OX=96794 PE=1 SV=1

**Number of Unique Peptides:** 4

**m/z:** 569.95

**MS/MS ID:** 4024

**Score:** 132.97

**Spectrum:** 1/4


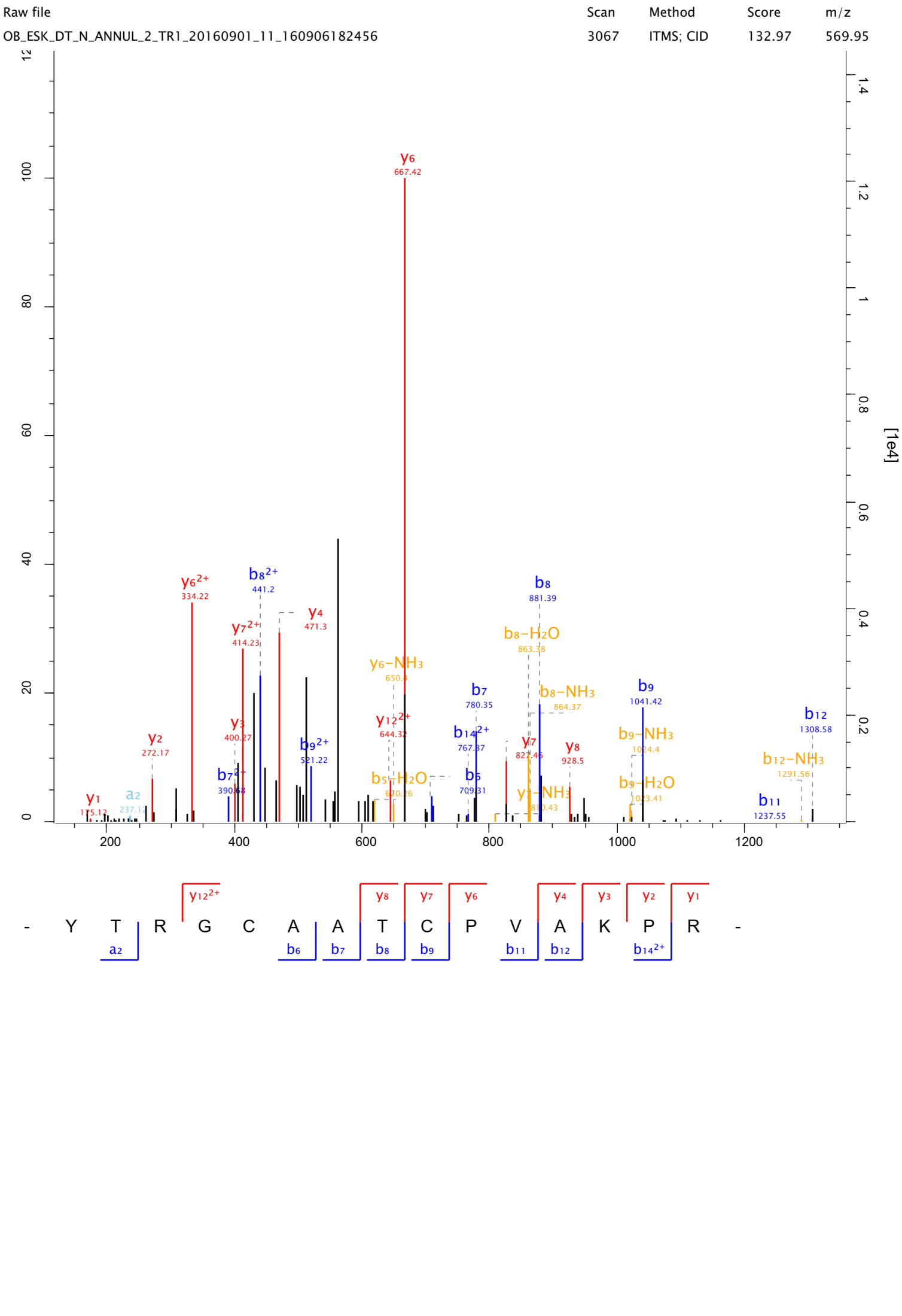


**Protein ID – P01399**

**Protein name:** Weak toxin CM-13b OS=Naja annulifera OX=96794 PE=1 SV=1

**Number of Unique Peptides:** 4

**m/z:** 634.77

**MS/MS ID:** 576

**Score:** 237.51

**Spectrum:** 2/4


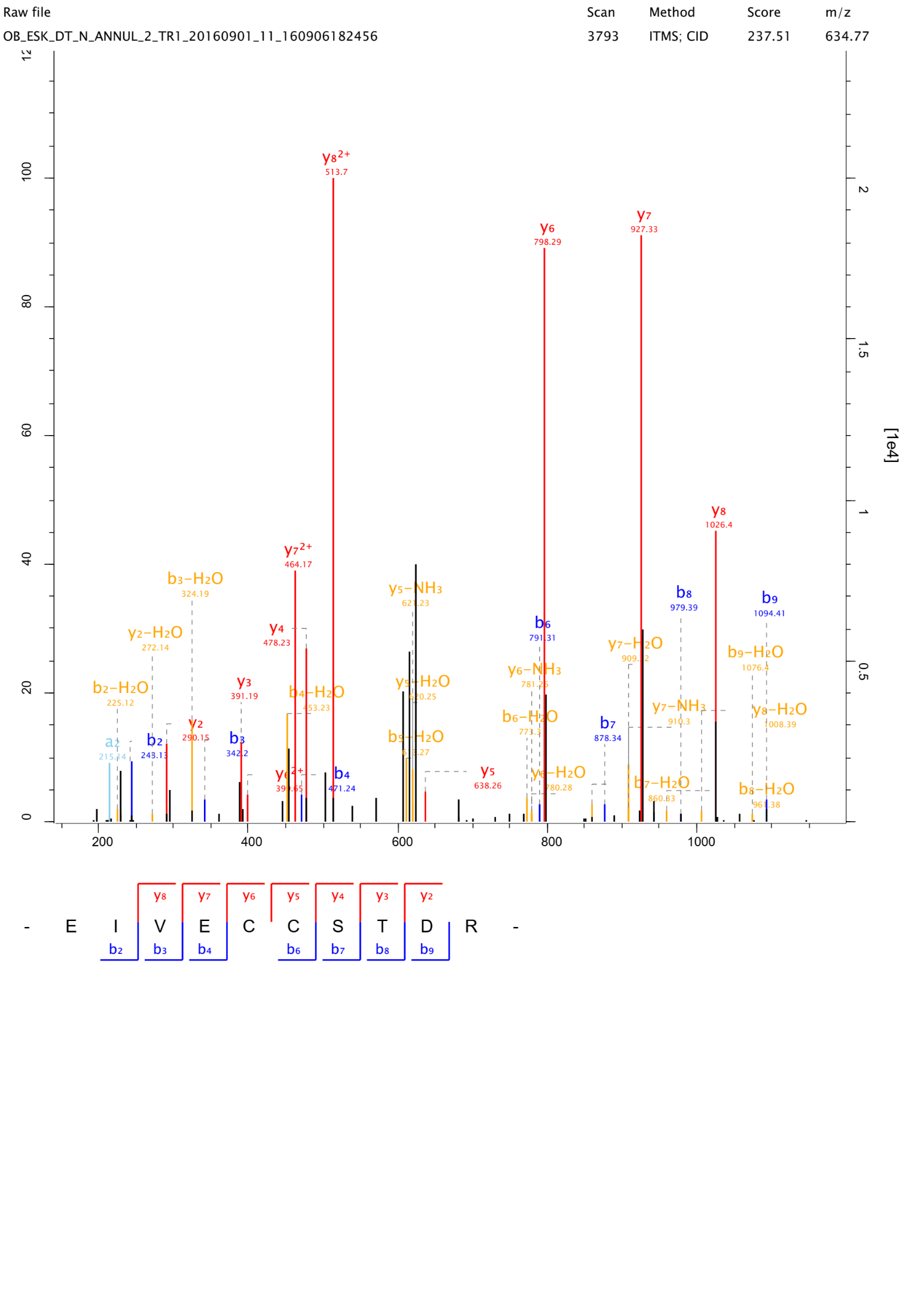


**Protein ID – P01399**

**Protein name:** Weak toxin CM-13b OS=Naja annulifera OX=96794 PE=1 SV=1

**Number of Unique Peptides:** 4

**m/z:** 840.33

**MS/MS ID:** 583

**Score:** 248.19

**Spectrum:** 3/4


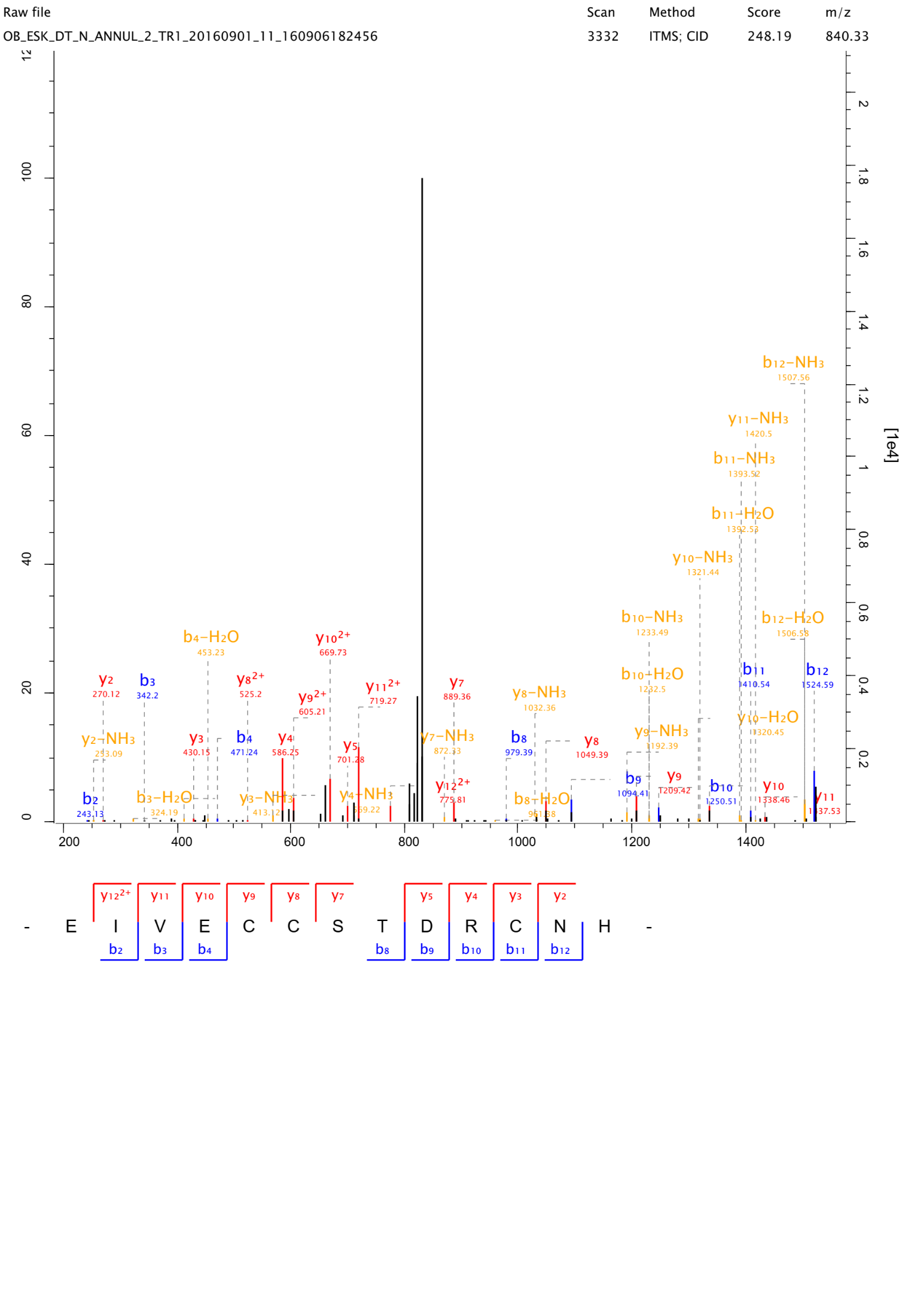


**Protein ID – P01399**

**Protein name:** Weak toxin CM-13b OS=Naja annulifera OX=96794 PE=1 SV=1

**Number of Unique Peptides:** 4

**m/z:** 644.32

**MS/MS ID:** 791

**Score:** 214.67

**Spectrum:** 4/4


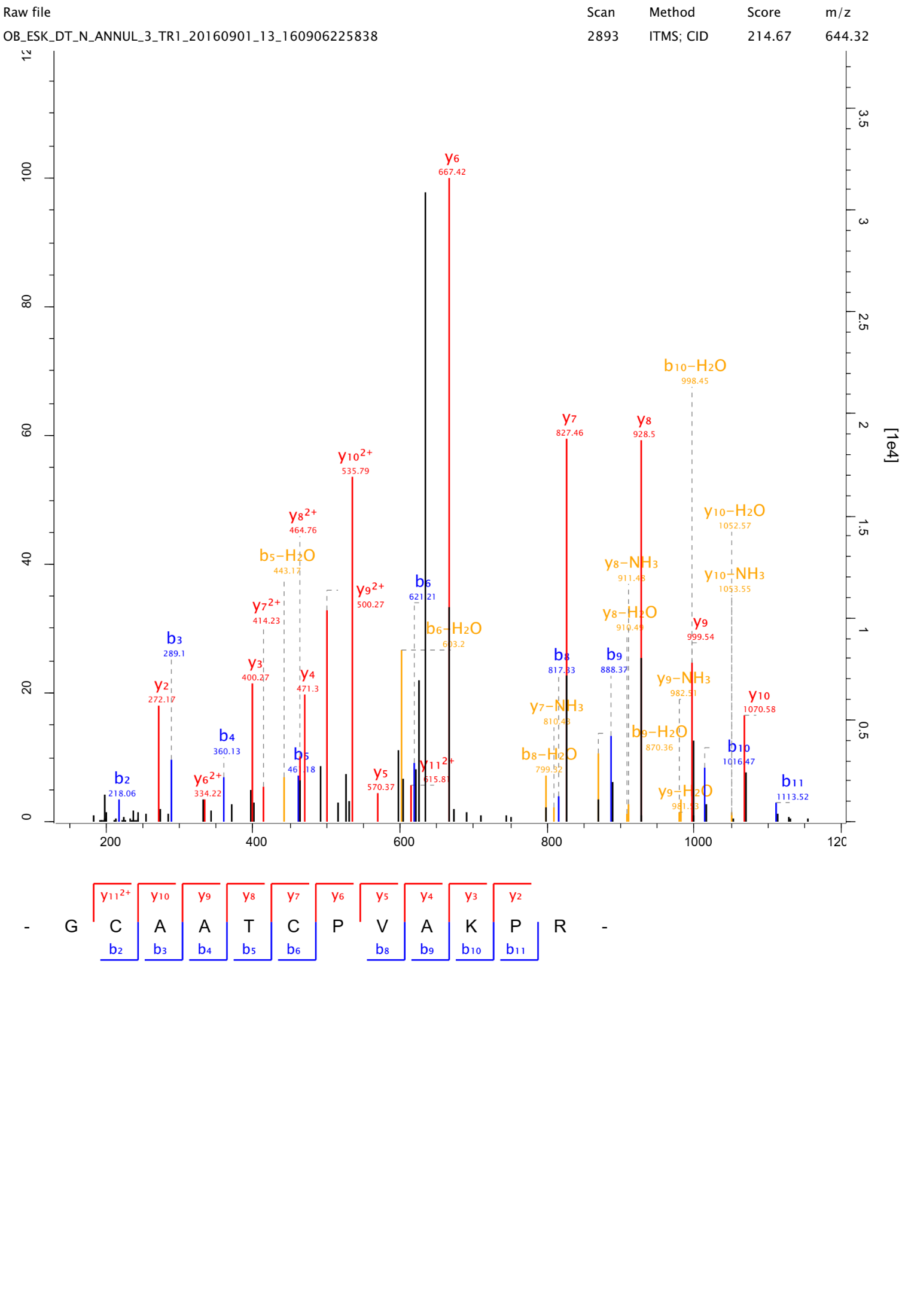


**Protein ID – P01400**

**Protein name:** Weak toxin S4C11 OS=Naja melanoleuca OX=8643 PE=1 SV=1

**Number of Unique Peptides:** 1

**m/z:** 520.77

**MS/MS ID:** 753

**Score:** 173.39

**Spectrum:** 1/1


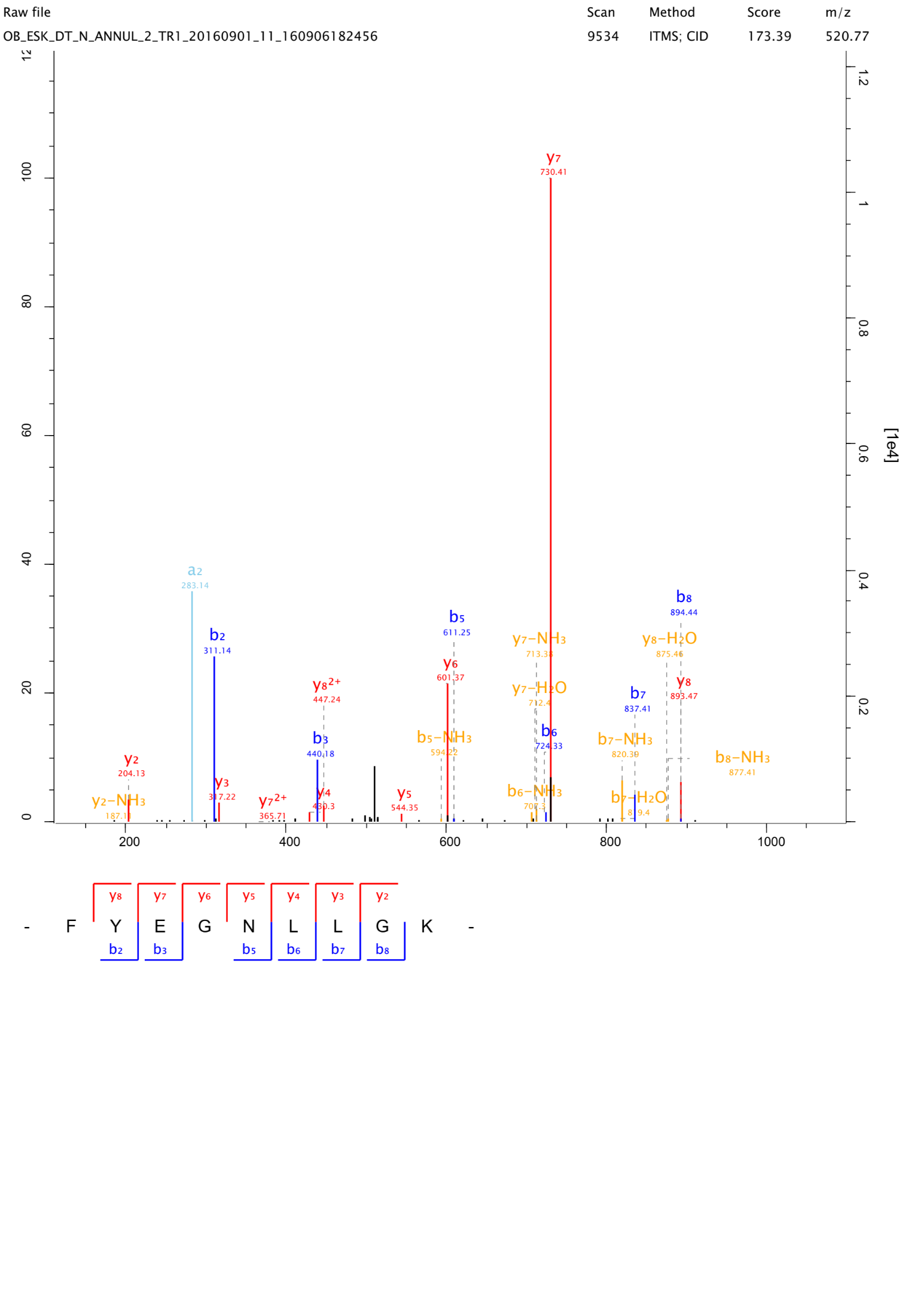


**Protein ID – P01401**

**Protein name:** Weak toxin CM-11 OS=Naja haje haje OX=8642 PE=1 SV=1

**Number of Unique Peptides:** 2

**m/z:** 842.86

**MS/MS ID:** 590

**Score:** 198.32

**Spectrum:** 1/2


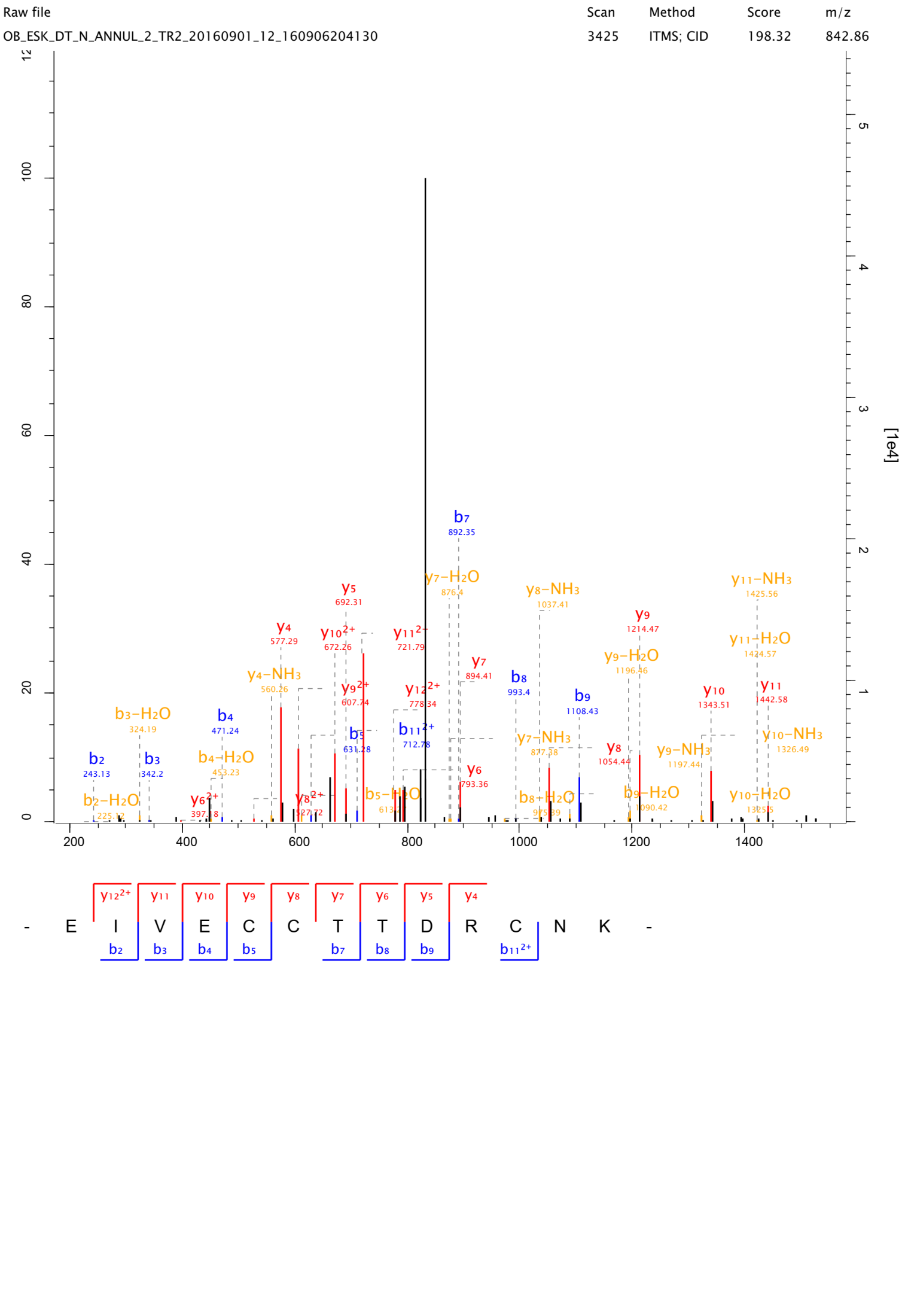


**Protein ID – P01401**

**Protein name:** Weak toxin CM-11 OS=Naja haje haje OX=8642 PE=1 SV=1

**Number of Unique Peptides:** 2

**m/z:** 576.77

**MS/MS ID:** 2809

**Score:** 180.09

**Spectrum:** 2/2


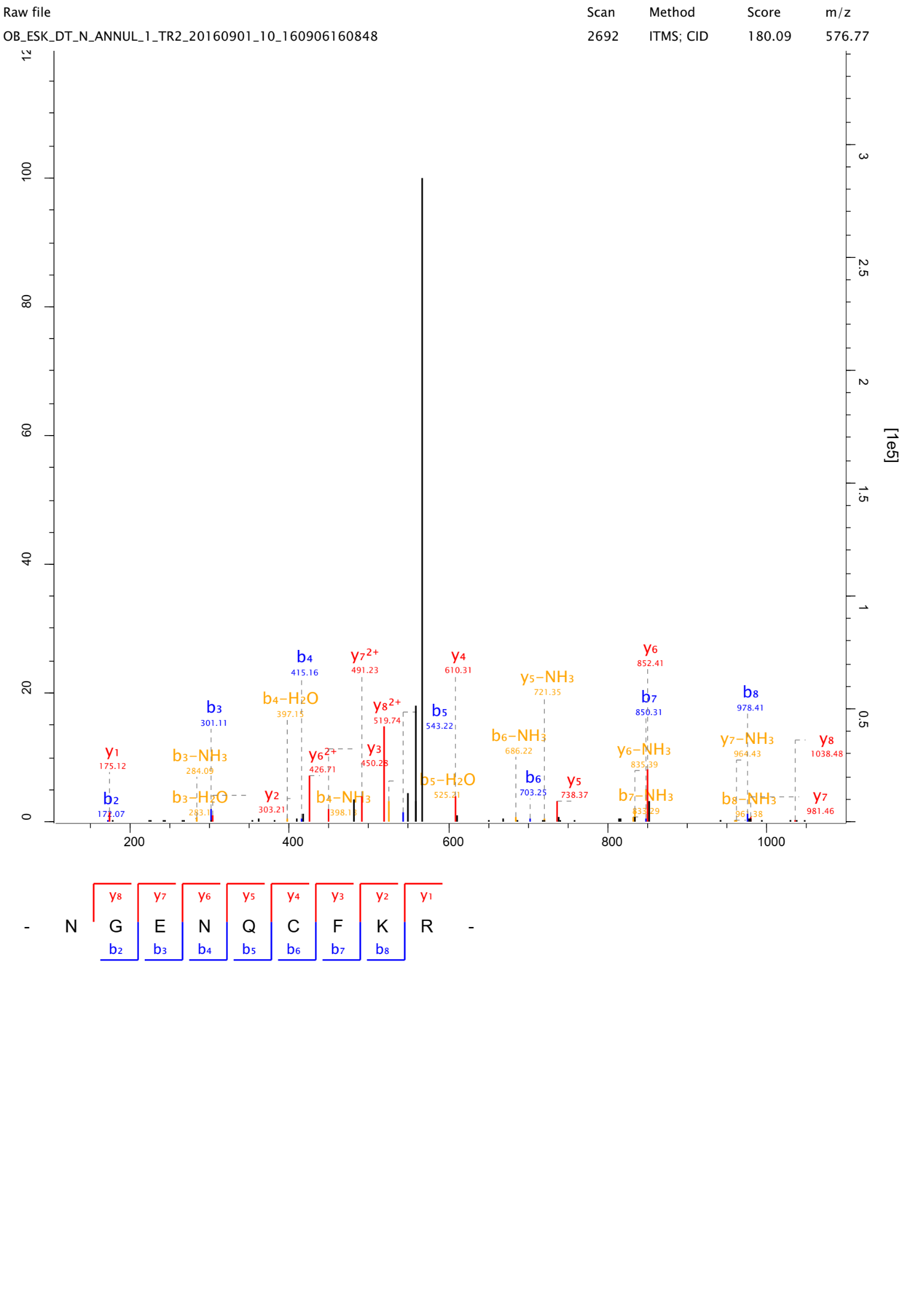


**Protein ID – P01421**

**Protein name:** Short neurotoxin 4 OS=Naja annulifera OX=96794 PE=1 SV=1

**Number of Unique Peptides:** 6

**m/z:** 1072.02

**MS/MS ID:** 3289

**Score:** 294.93

**Spectrum:** 1/6


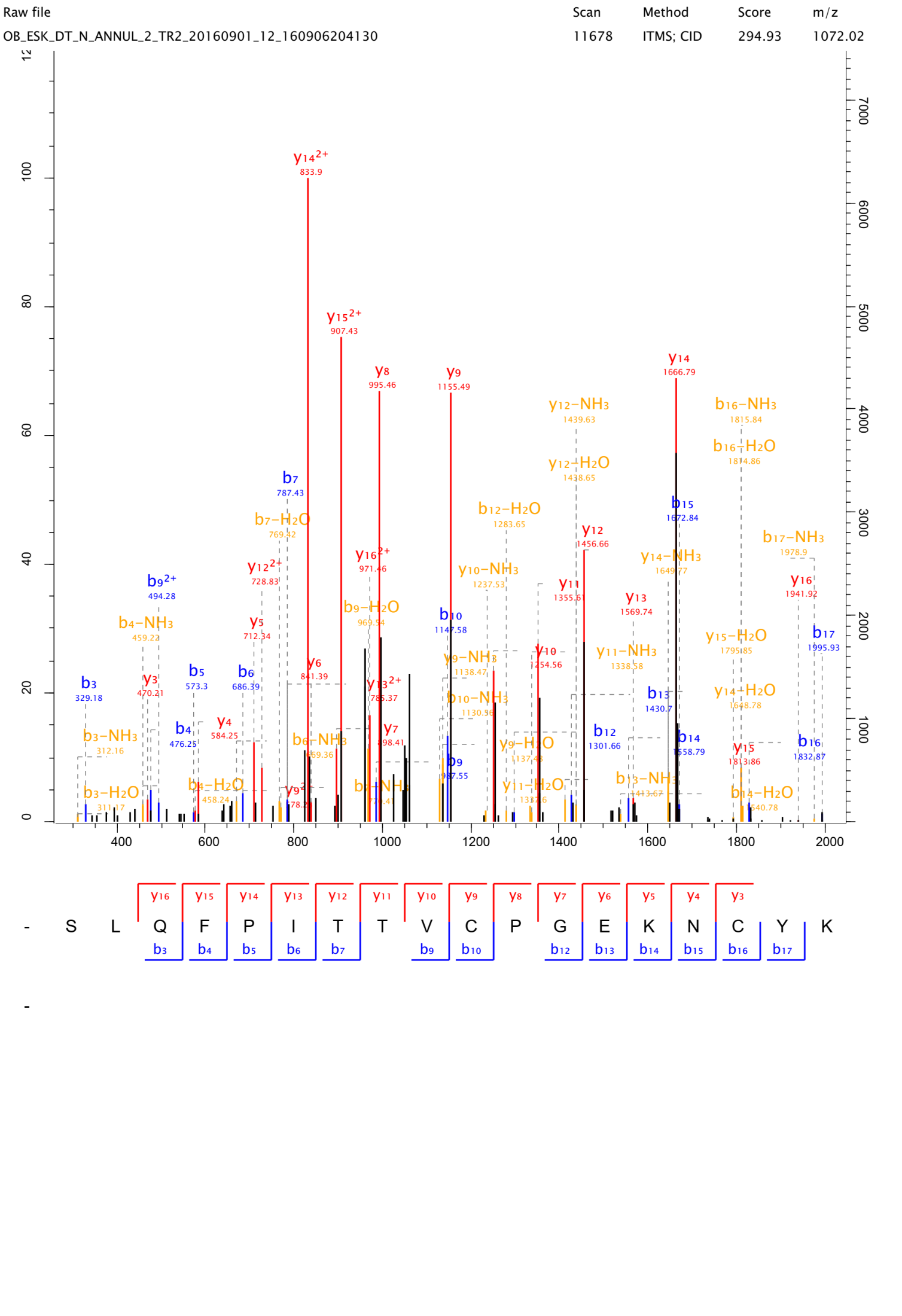


**Protein ID – P01421**

**Protein name:** Short neurotoxin 4 OS=Naja annulifera OX=96794 PE=1 SV=1

**Number of Unique Peptides:** 6

**m/z:** 788.91

**MS/MS ID:** 3198

**Score:** 227.91

**Spectrum:** 2/6


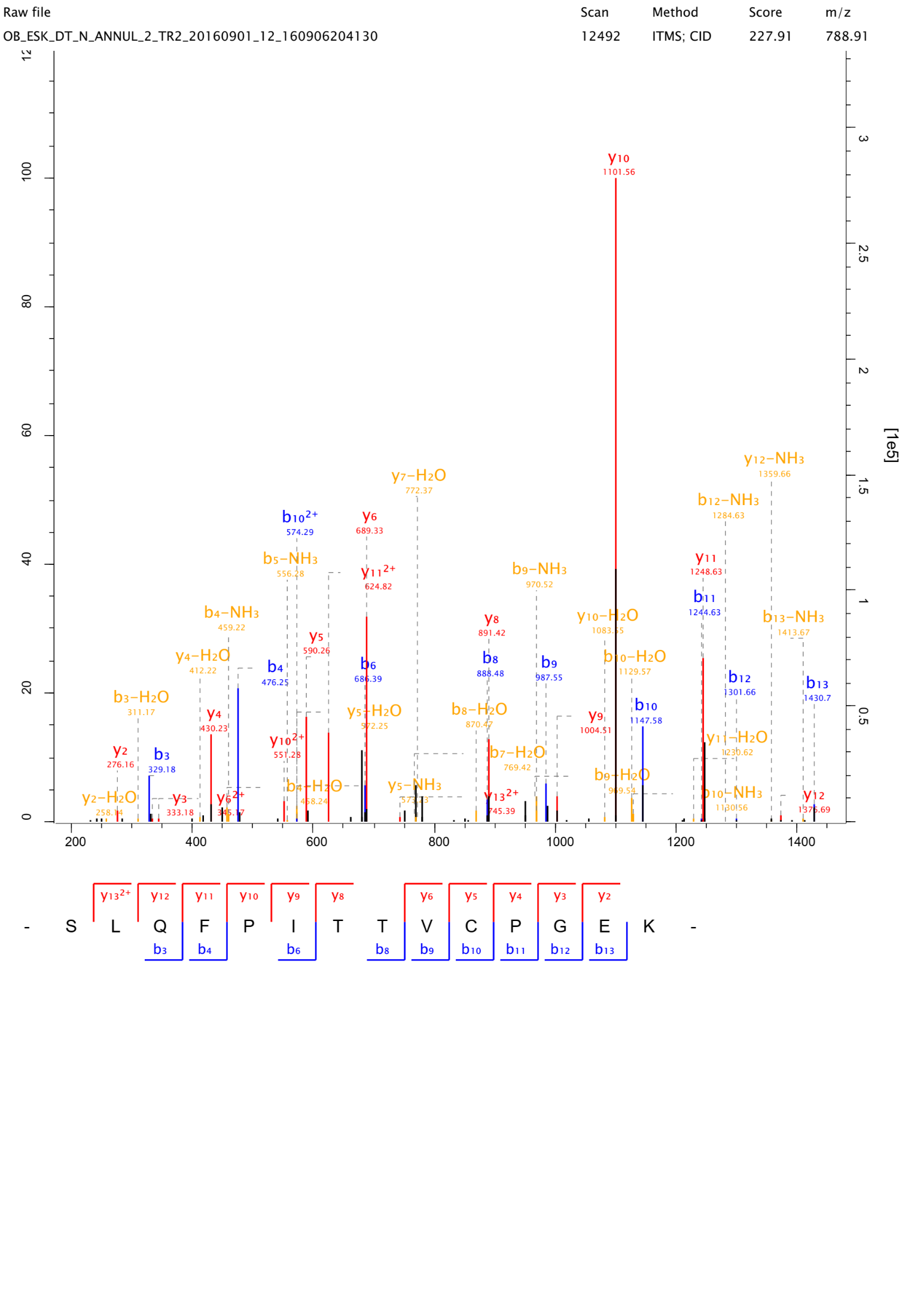


**Protein ID – P01421**

**Protein name:** Short neurotoxin 4 OS=Naja annulifera OX=96794 PE=1 SV=1

**Number of Unique Peptides:** 6

**m/z:** 720.38

**MS/MS ID:** 2949

**Score:** 110.64

**Spectrum:** 3/6


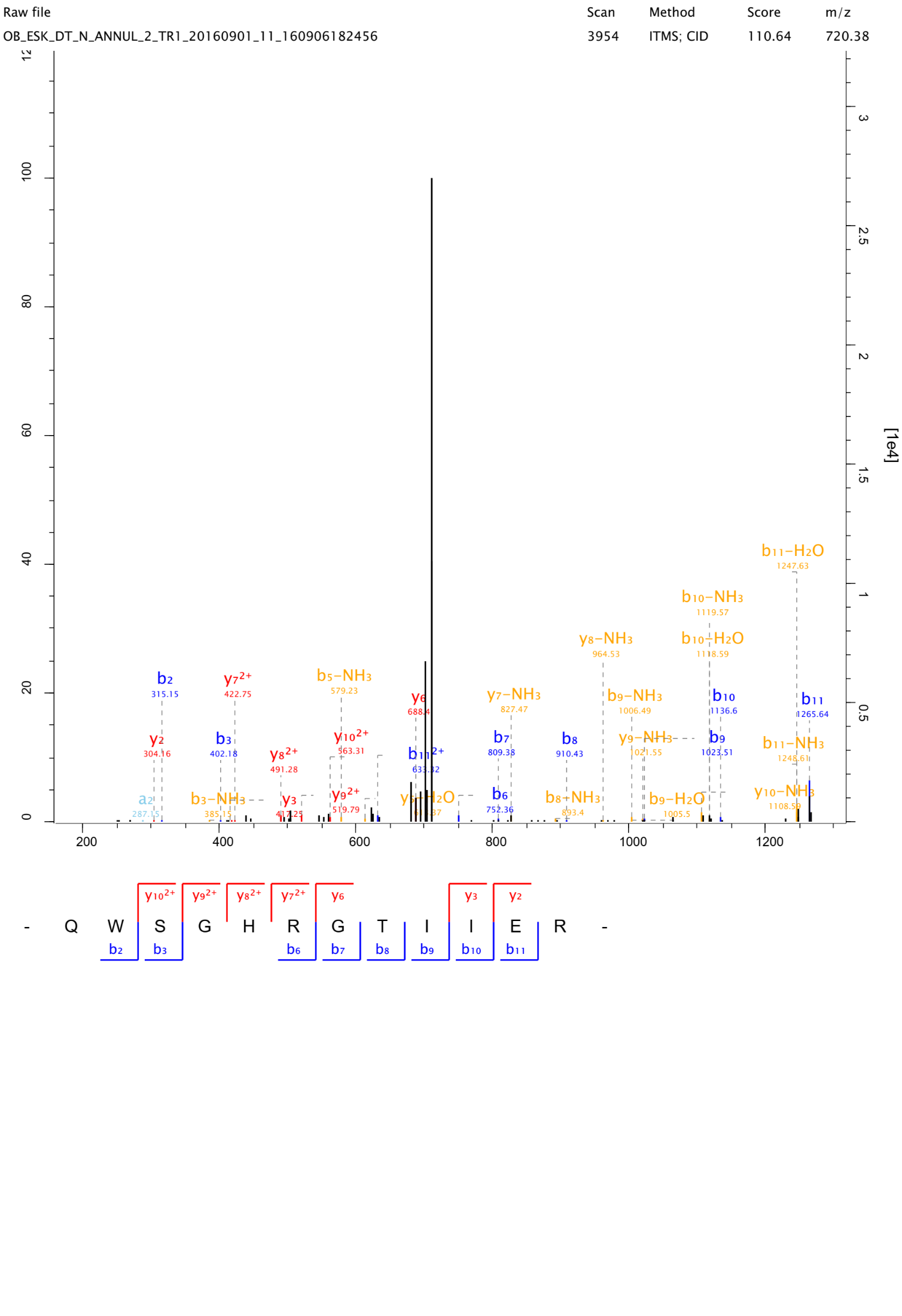


**Protein ID – P01421**

**Protein name:** Short neurotoxin 4 OS=Naja annulifera OX=96794 PE=1 SV=1

**Number of Unique Peptides:** 6

**m/z:** 934.92

**MS/MS ID:** 2086

**Score:** 267.5

**Spectrum:** 4/6


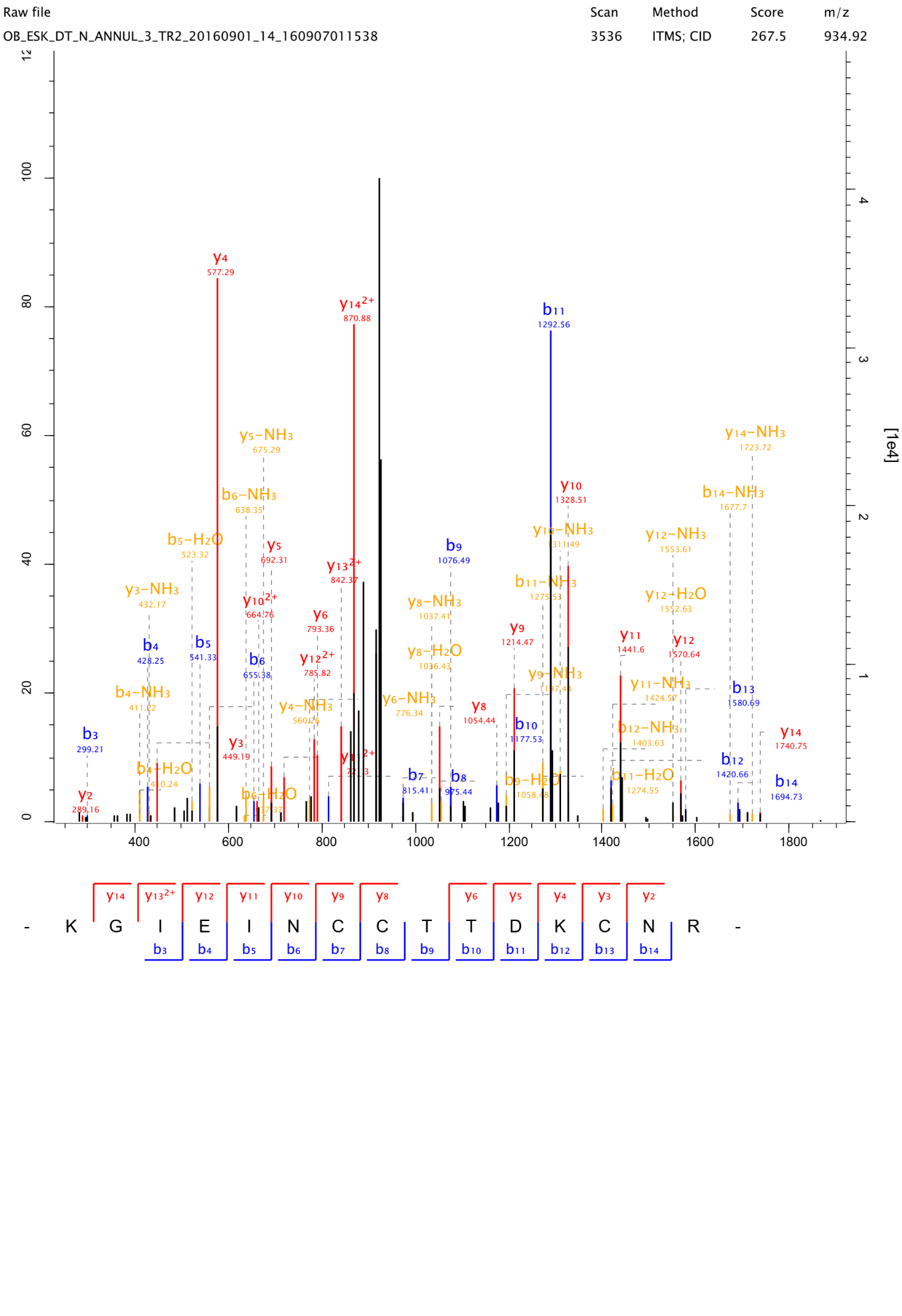


**Protein ID – P01421**

**Protein name:** Short neurotoxin 4 OS=Naja annulifera OX=96794 PE=1 SV=1

**Number of Unique Peptides:** 6

**m/z:** 870.88

**MS/MS ID:** 1727

**Score:** 224.34

**Spectrum:** 5/6


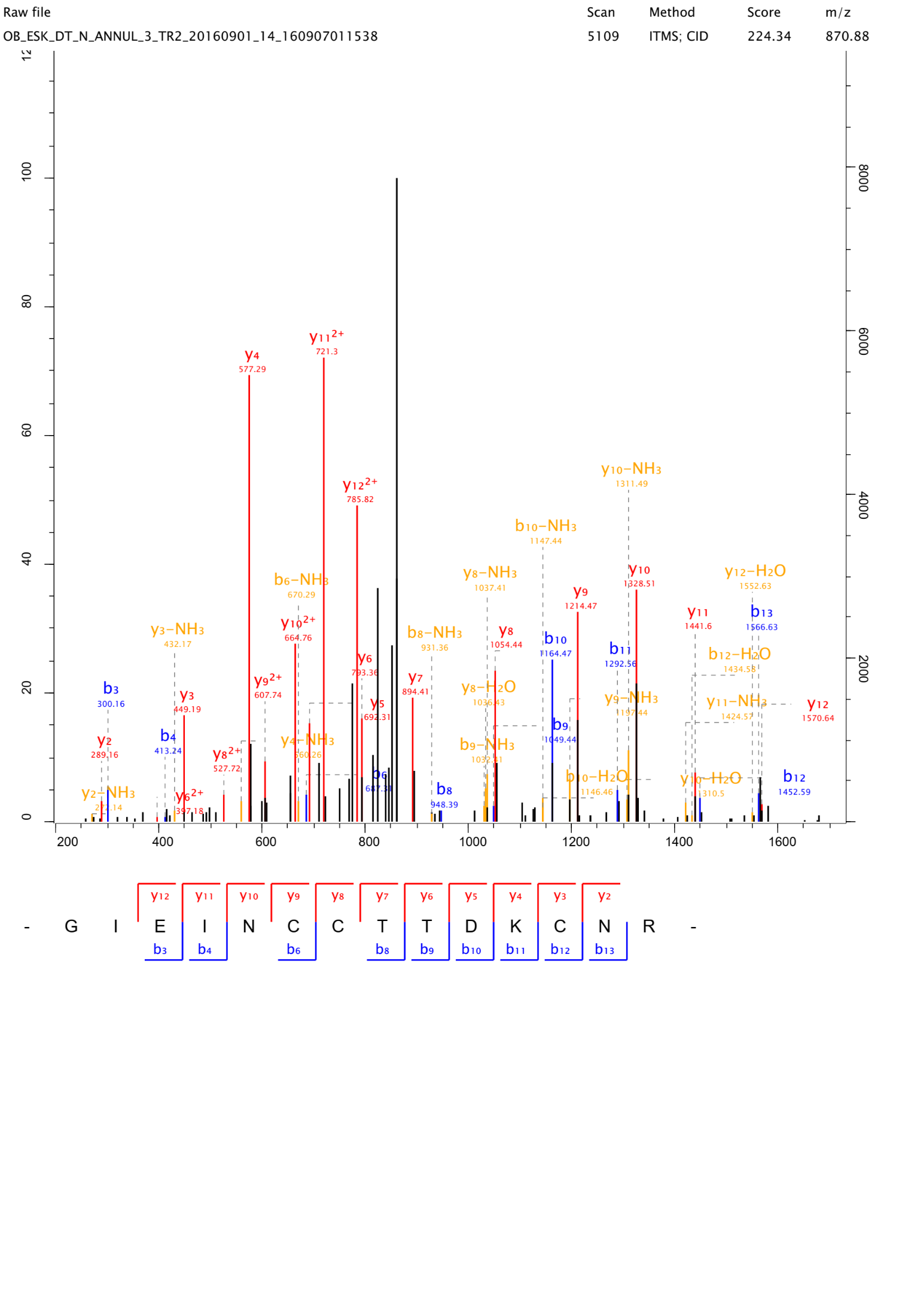


**Protein ID – P01421**

**Protein name:** Short neurotoxin 4 OS=Naja annulifera OX=96794 PE=1 SV=1

**Number of Unique Peptides:** 6

**m/z:** 1135.57

**MS/MS ID:** 3314

**Score:** 202.94

**Spectrum:** 6/6


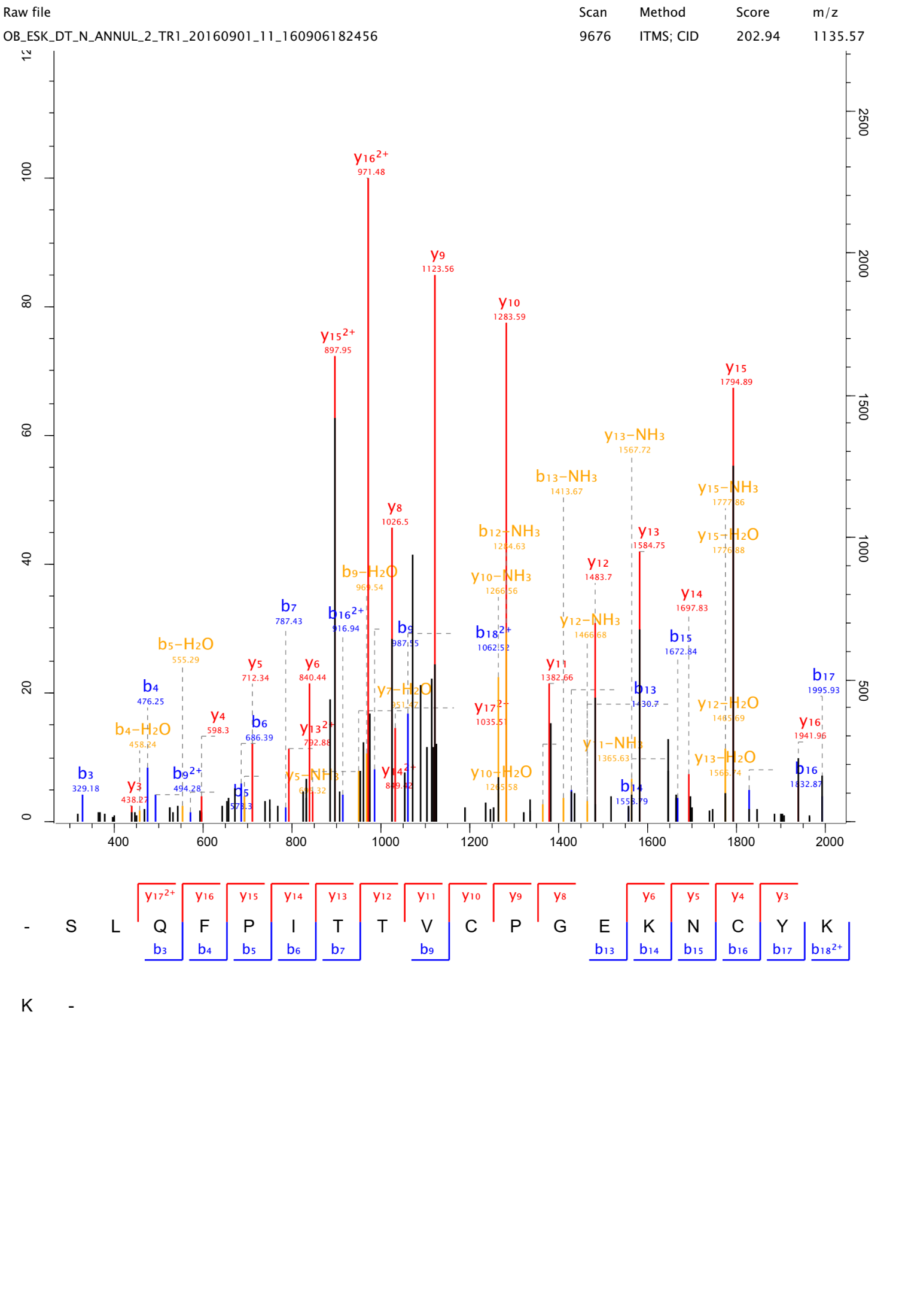


**Protein ID – P01422**

**Protein name:** Short neurotoxin 2 OS=Naja annulifera OX=96794 PE=1 SV=1

**Number of Unique Peptides:** 2

**m/z:** 590.29

**MS/MS ID:** 2757

**Score:** 108.58

**Spectrum:** 1/2


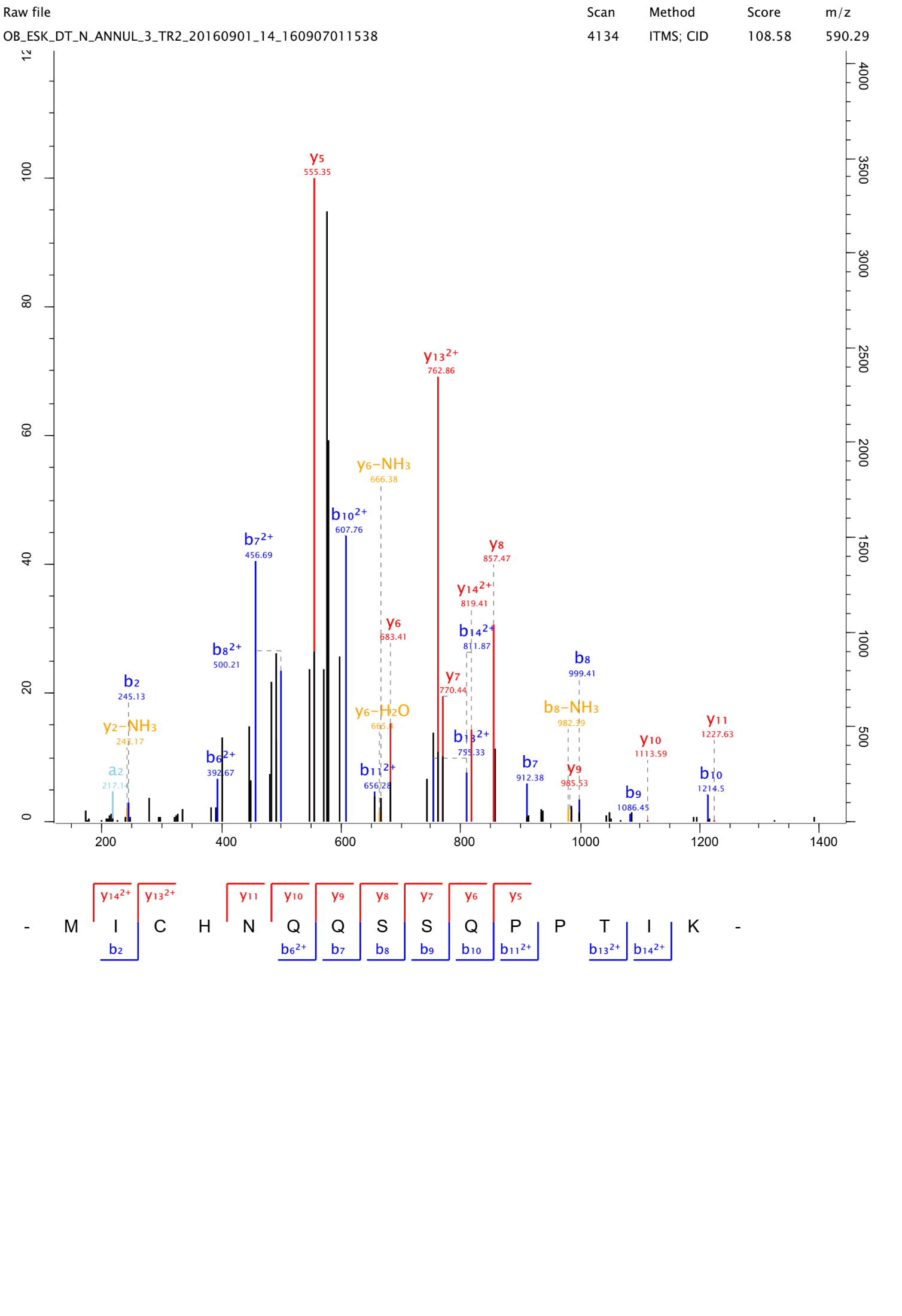


**Protein ID – P01422**

**Protein name:** Short neurotoxin 2 OS=Naja annulifera OX=96794 PE=1 SV=1

**Number of Unique Peptides:** 2

**m/z:** 542.77

**MS/MS ID:** 2087

**Score:** 162.97

**Spectrum:** 2/2


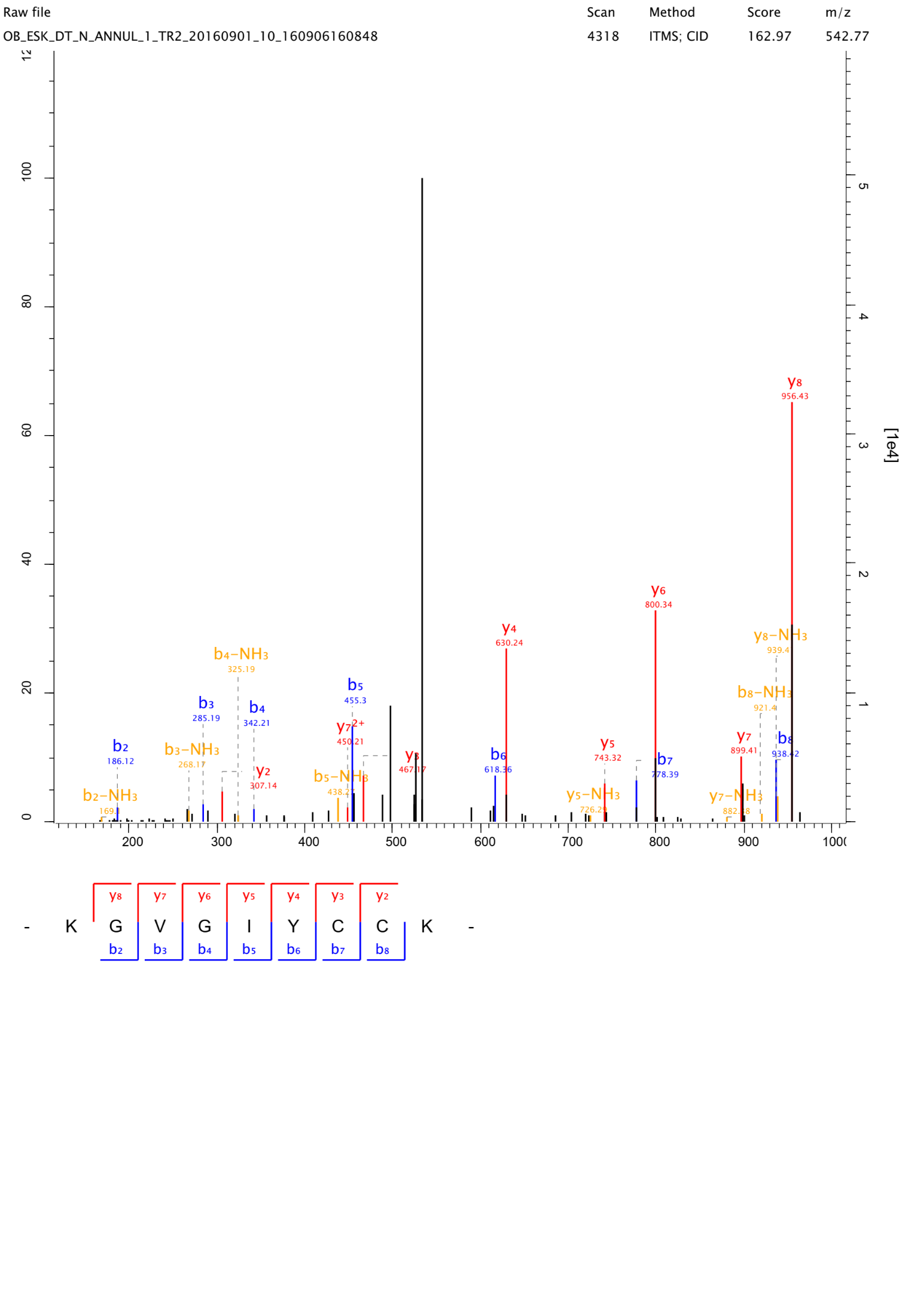


**Protein ID – P01426**

**Protein name:** Short neurotoxin 1 OS=Naja pallida OX=8658 PE=1 SV=1

**Number of Unique Peptides:** 2

**m/z:** 700.27

**MS/MS ID:** 2385

**Score:** 167.18

**Spectrum:** 1/2


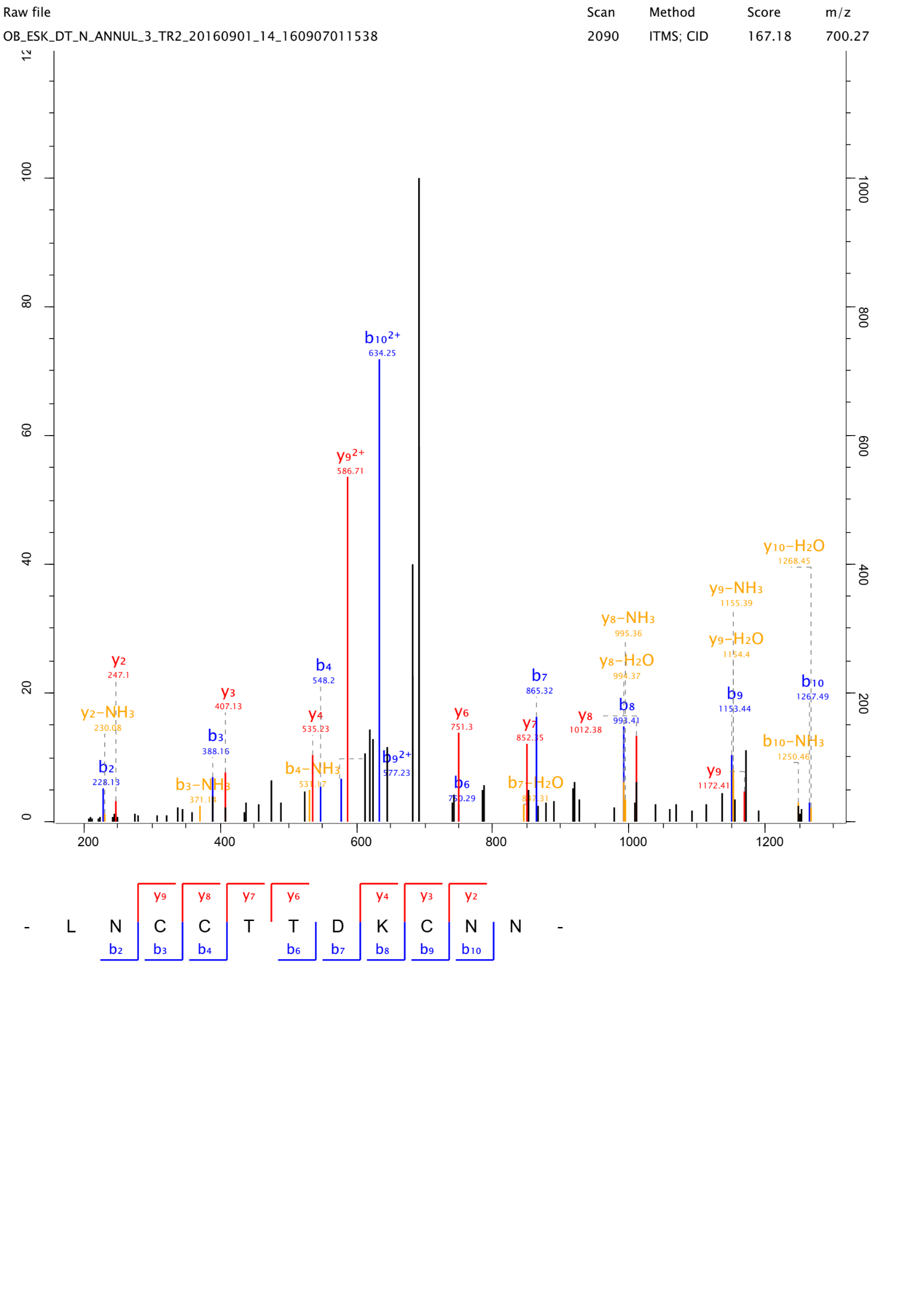


**Protein ID – P01426**

**Protein name:** Short neurotoxin 1 OS=Naja pallida OX=8658 PE=1 SV=1

**Number of Unique Peptides:** 2

**m/z:** 506.22

**MS/MS ID:** 2382

**Score:** 158.76

**Spectrum:** 2/2


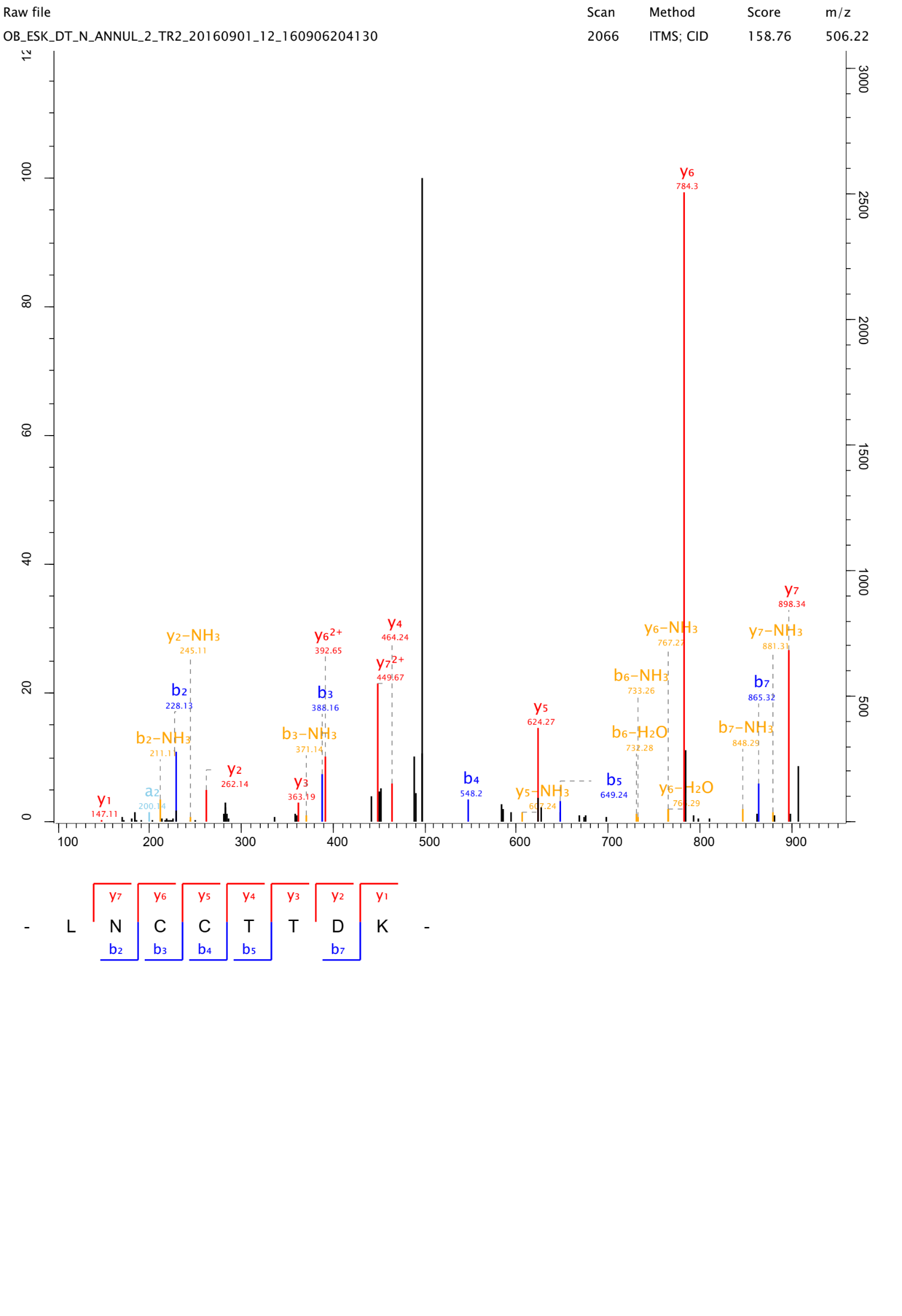


**Protein ID – P01453**

**Protein name:** Cytotoxin 10 OS=Naja annulifera OX=96794 PE=1 SV=1

**Number of Unique Peptides:** 2

**m/z:** 718.89

**MS/MS ID:** 2193

**Score:** 176.87

**Spectrum:** 1/2


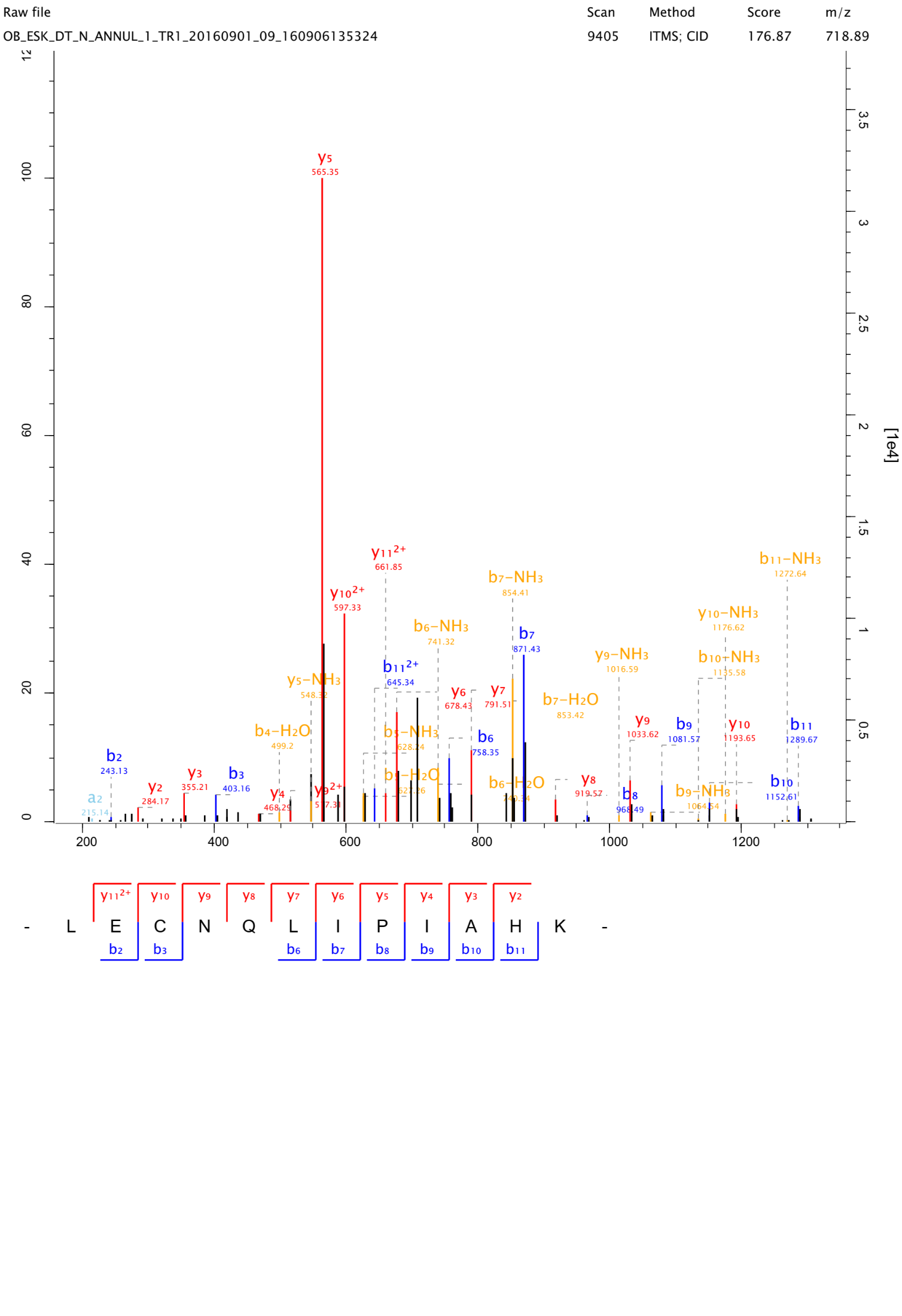


**Protein ID – P01453**

**Protein name:** Cytotoxin 10 OS=Naja annulifera OX=96794 PE=1 SV=1

**Number of Unique Peptides:** 2

**m/z:** 703.69

**MS/MS ID:** 2218

**Score:** 140.49

**Spectrum:** 2/2


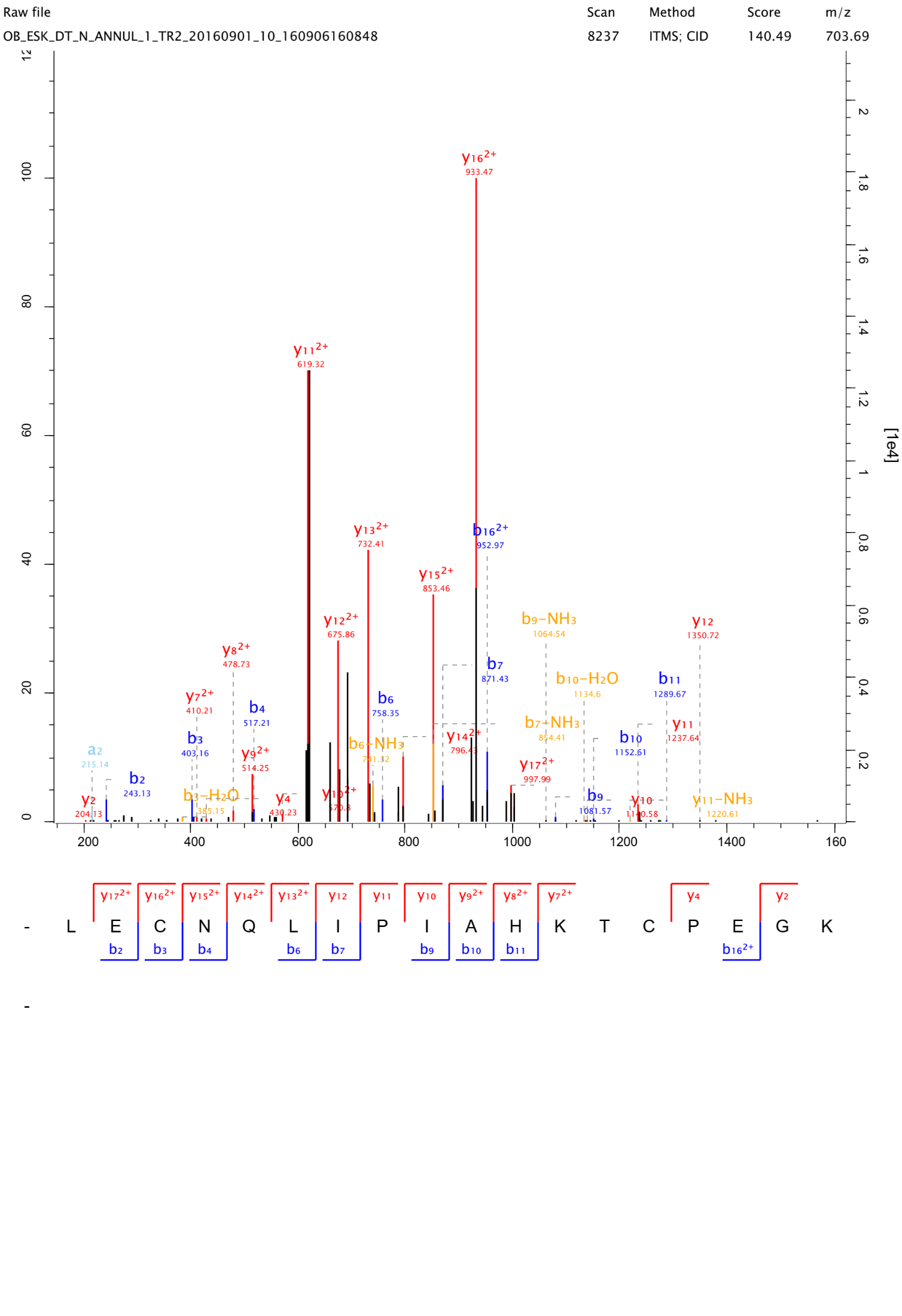


**Protein ID – P01454**

**Protein name:** Cytotoxin 9 OS=Naja annulifera OX=96794 PE=1 SV=1

**Number of Unique Peptides:** 3

**m/z:** 699.03

**MS/MS ID:** 2190

**Score:** 176.28

**Spectrum:** 1/3


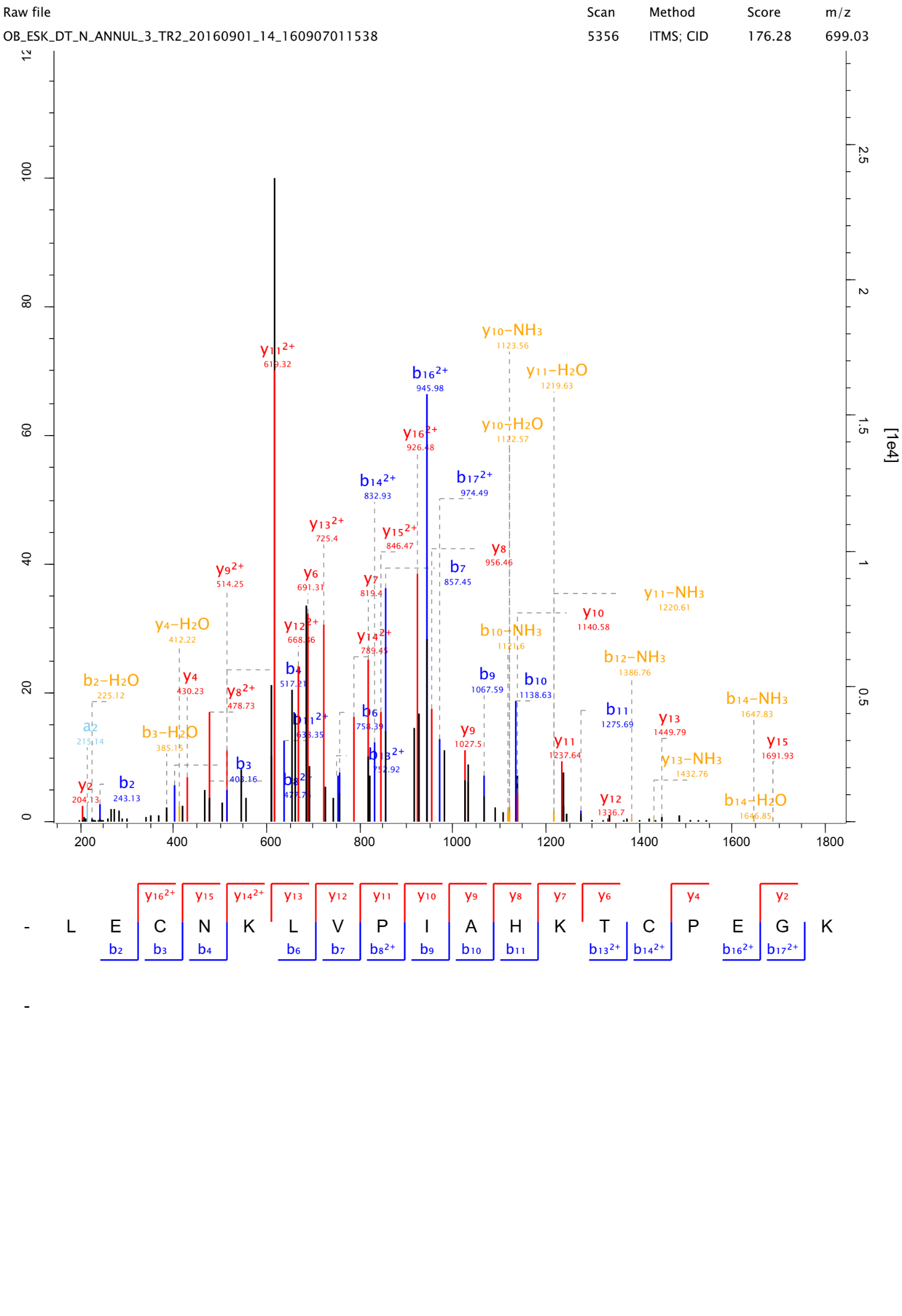


**Protein ID – P01454**

**Protein name:** Cytotoxin 9 OS=Naja annulifera OX=96794 PE=1 SV=1

**Number of Unique Peptides:** 3

**m/z:** 711.4

**MS/MS ID:** 2178

**Score:** 166.24

**Spectrum:** 2/3


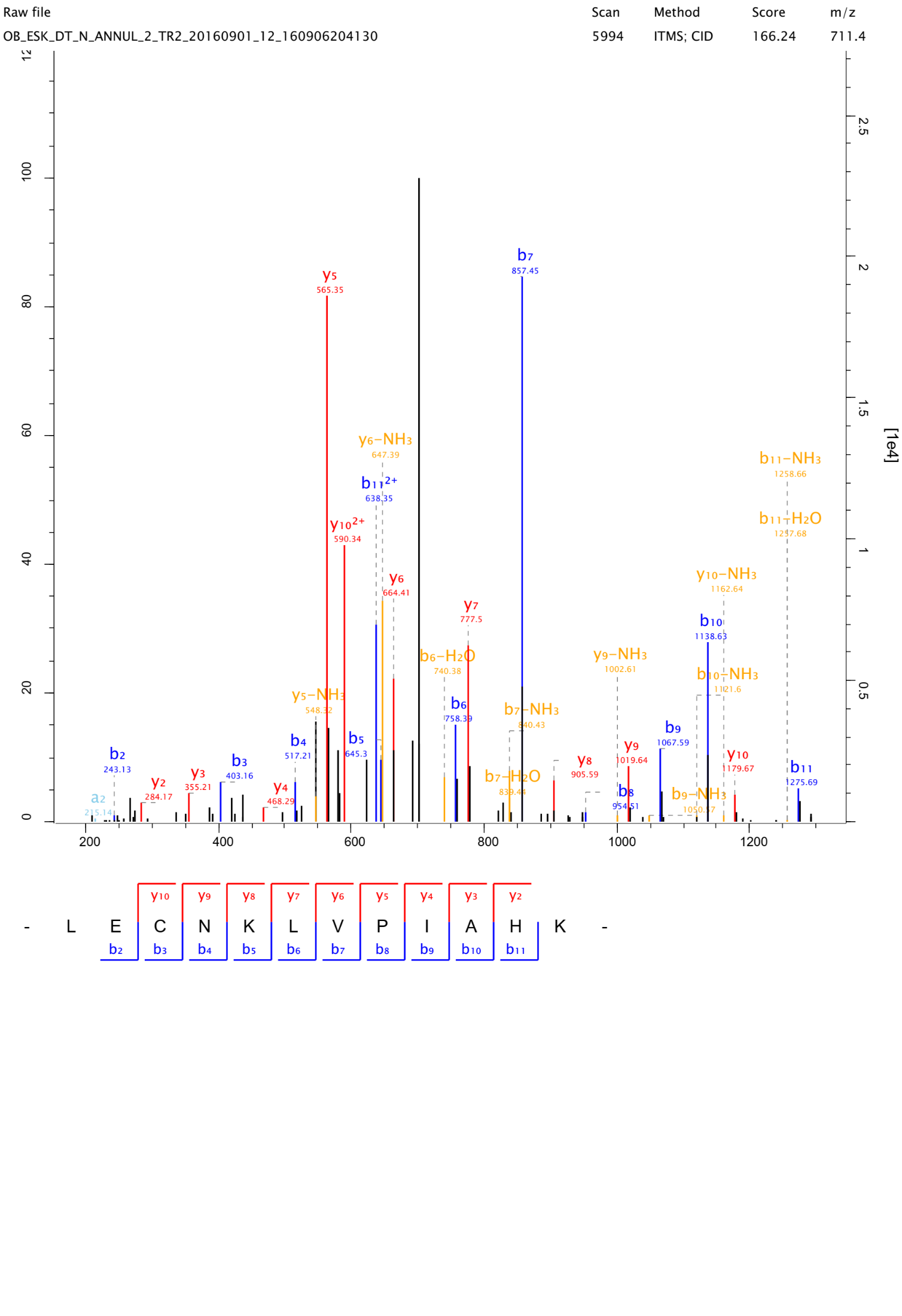


**Protein ID – P01454**

**Protein name:** Cytotoxin 9 OS=Naja annulifera OX=96794 PE=1 SV=1

**Number of Unique Peptides:** 3

**m/z:** 725.4

**MS/MS ID:** 2531

**Score:** 116.9

**Spectrum:** 3/3


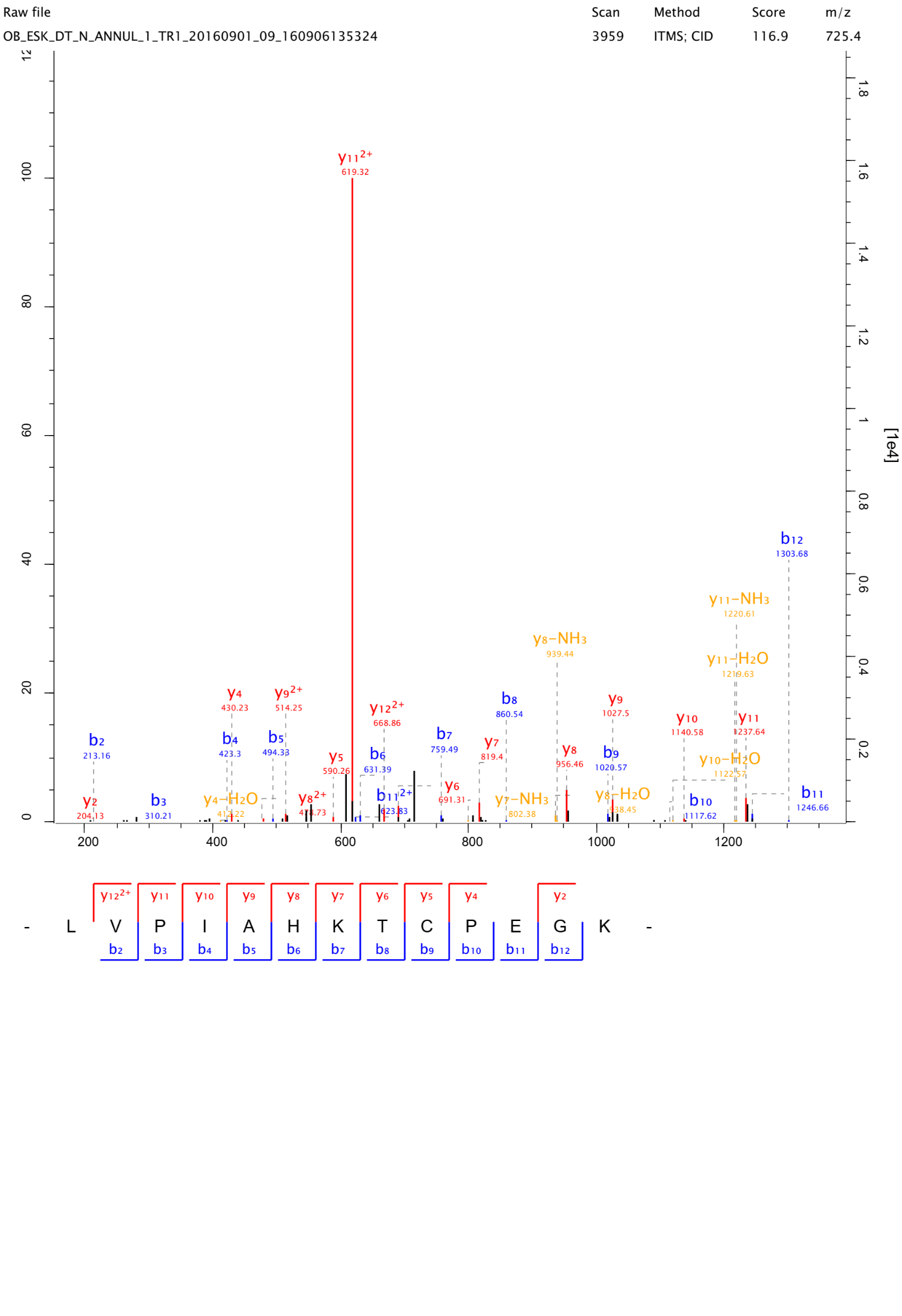


**Protein ID – P01456**

**Protein name:** Cytotoxin 1 OS=Naja nivea OX=8655 PE=1 SV=1

**Number of Unique Peptides:** 3

**m/z:** 632.36

**MS/MS ID:** 373

**Score:** 182.98

**Spectrum:** 1/3


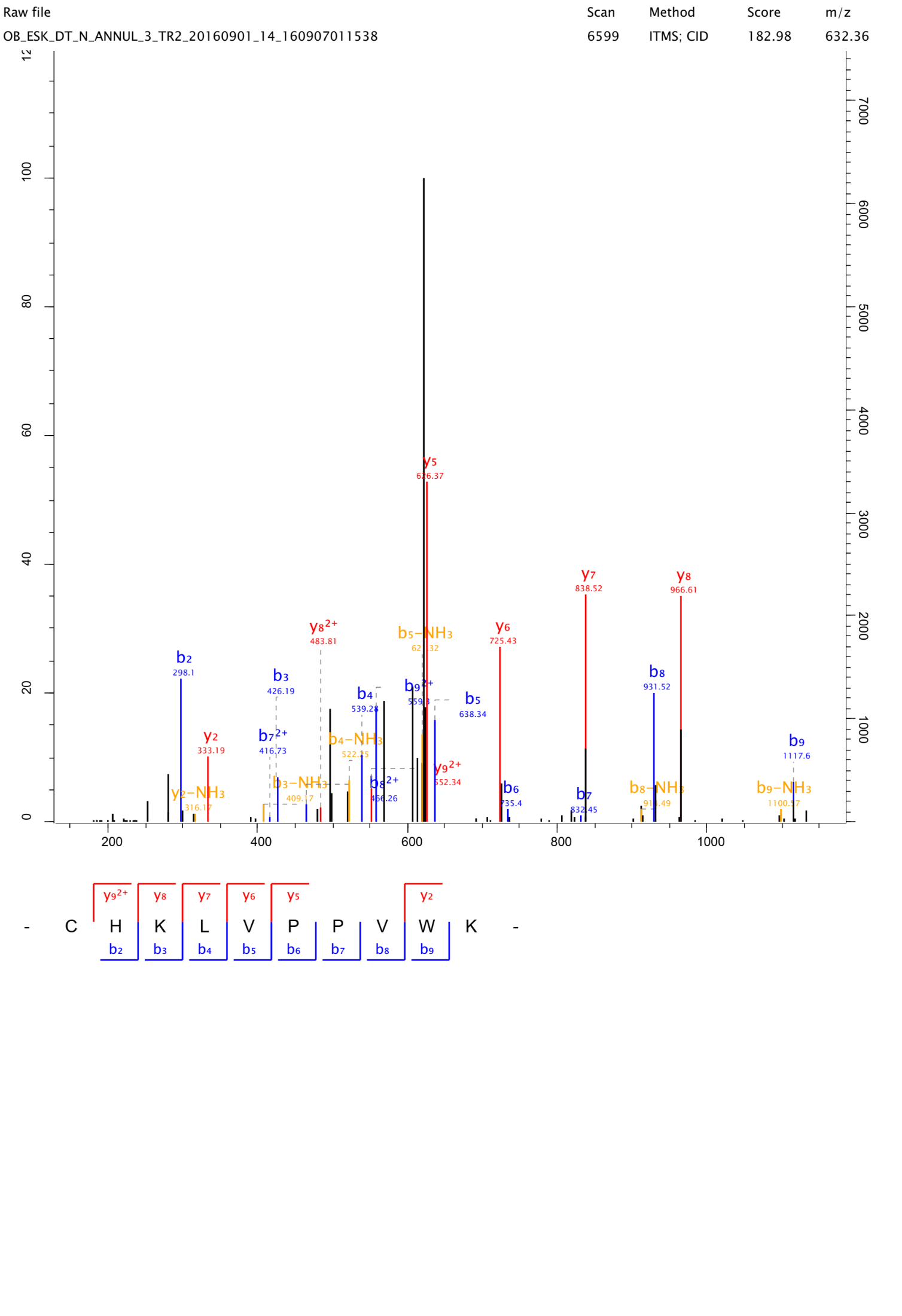


**Protein ID – P01456**

**Protein name:** Cytotoxin 1 OS=Naja nivea OX=8655 PE=1 SV=1

**Number of Unique Peptides:** 3

**m/z:** 960.92

**MS/MS ID:** 520

**Score:** 118.5

**Spectrum:** 2/3


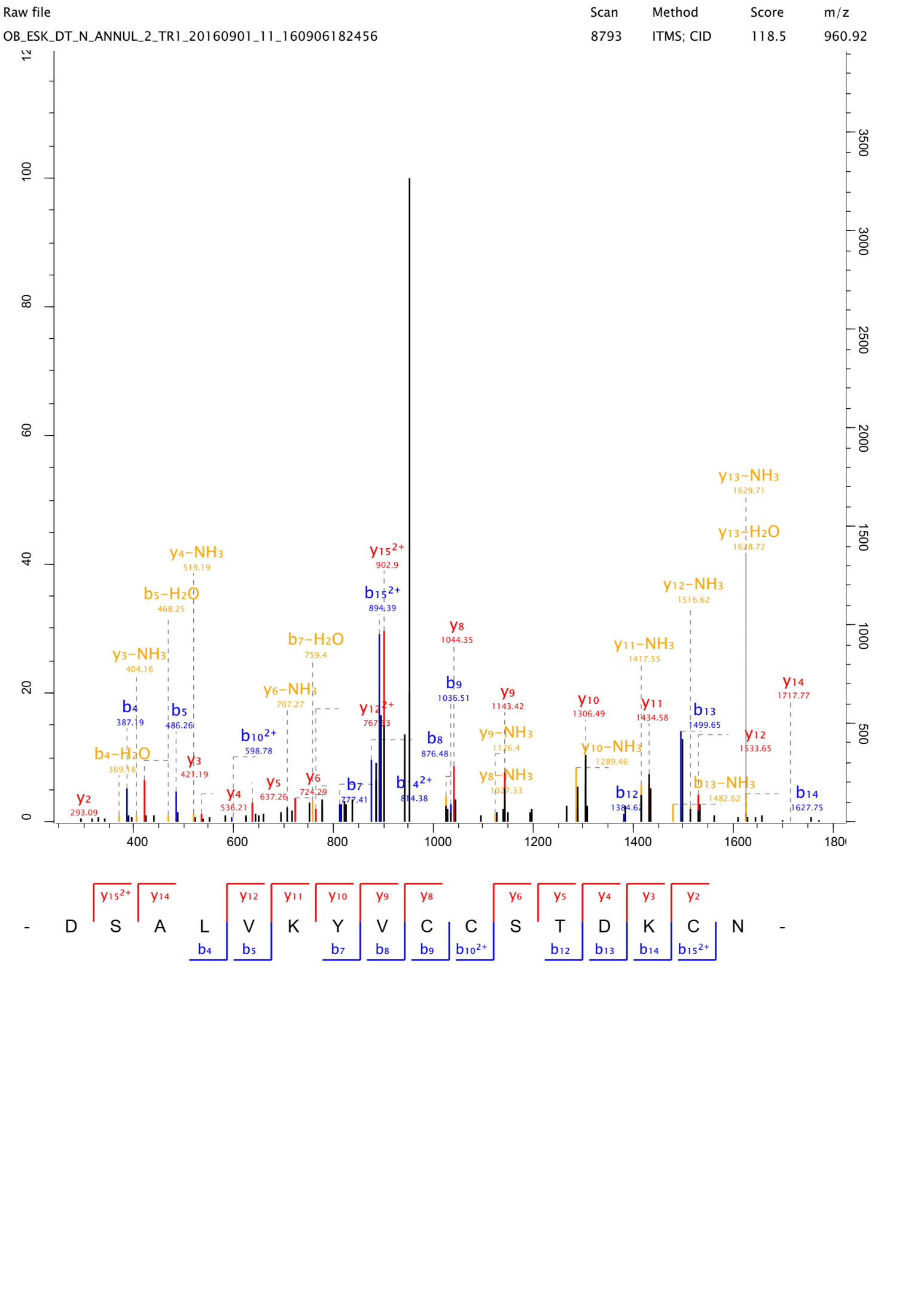


**Protein ID – P01456**

**Protein name:** Cytotoxin 1 OS=Naja nivea OX=8655 PE=1 SV=1

**Number of Unique Peptides:** 3

**m/z:** 419.76

**MS/MS ID:** 2585

**Score:** 103.28

**Spectrum:** 3/3


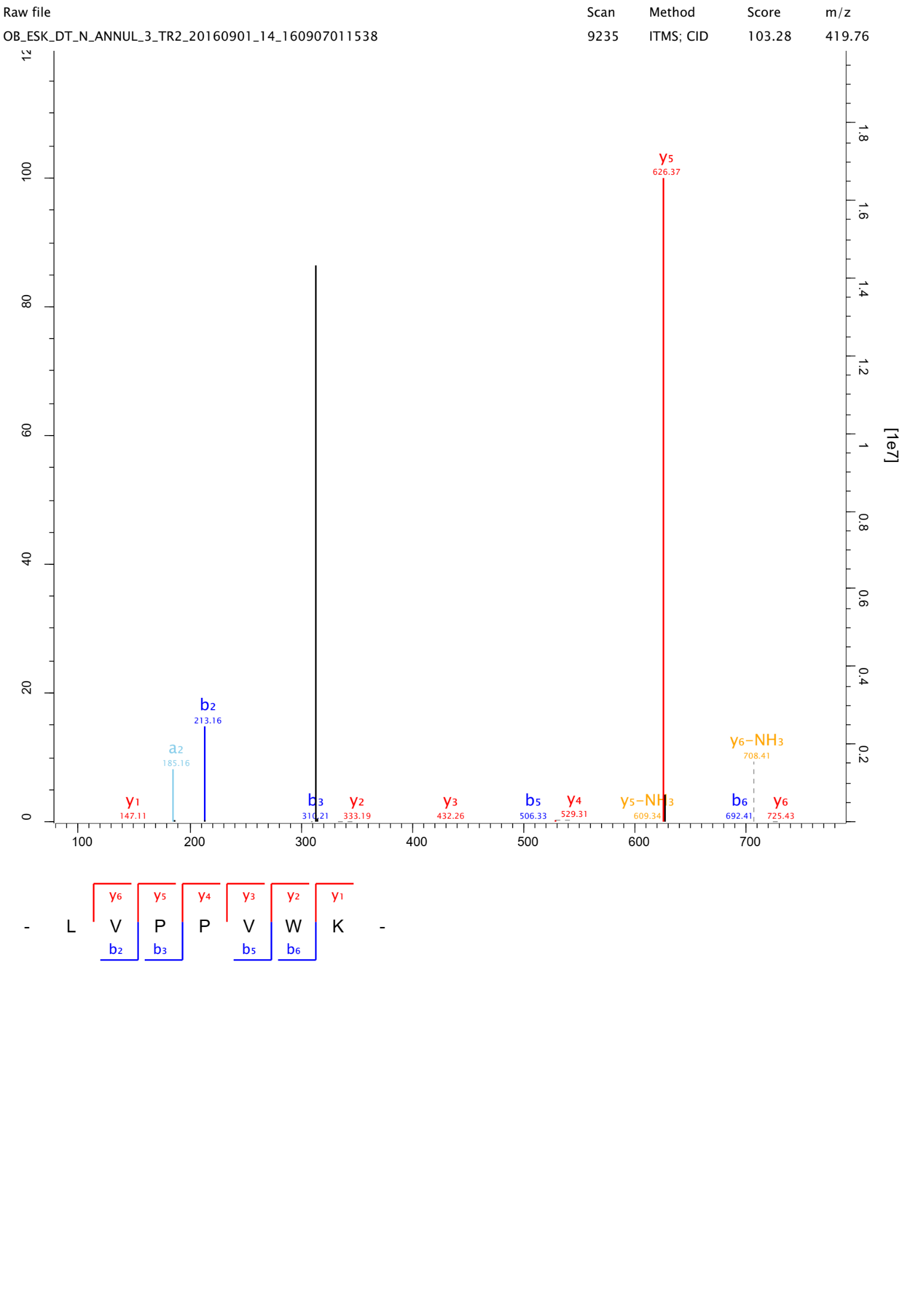


**Protein ID – P01457**

**Protein name:** Cytotoxin 5 OS=Naja haje haje OX=8642 PE=1 SV=1

**Number of Unique Peptides:** 2

**m/z:** 656.34

**MS/MS ID:** 457

**Score:** 145.72

**Spectrum:** 1/2


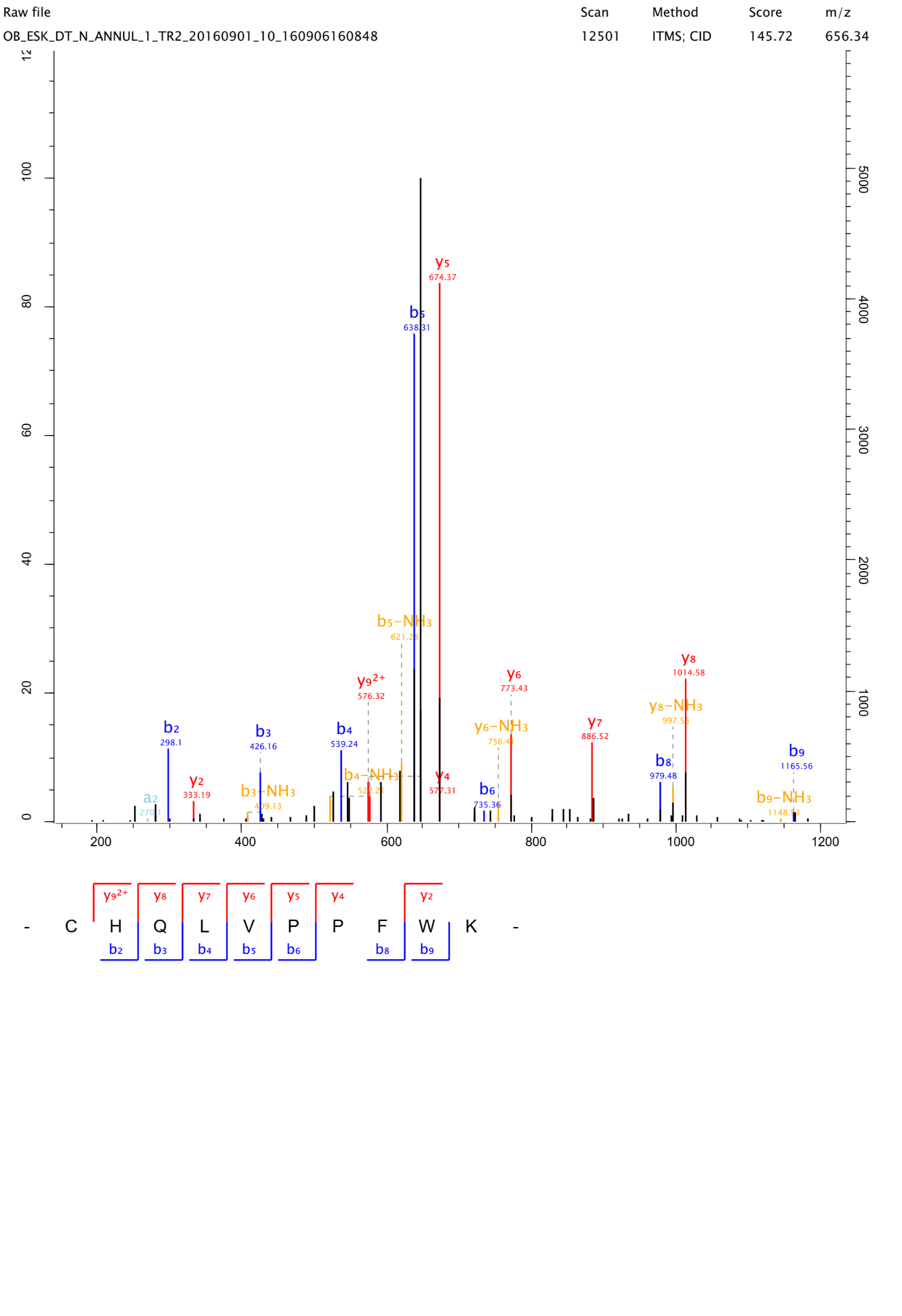


**Protein ID – P01457**

**Protein name:** Cytotoxin 5 OS=Naja haje haje OX=8642 PE=1 SV=1

**Number of Unique Peptides:** 2

**m/z:** 777.43

**MS/MS ID:** 2361

**Score:** 135.8

**Spectrum:** 2/2


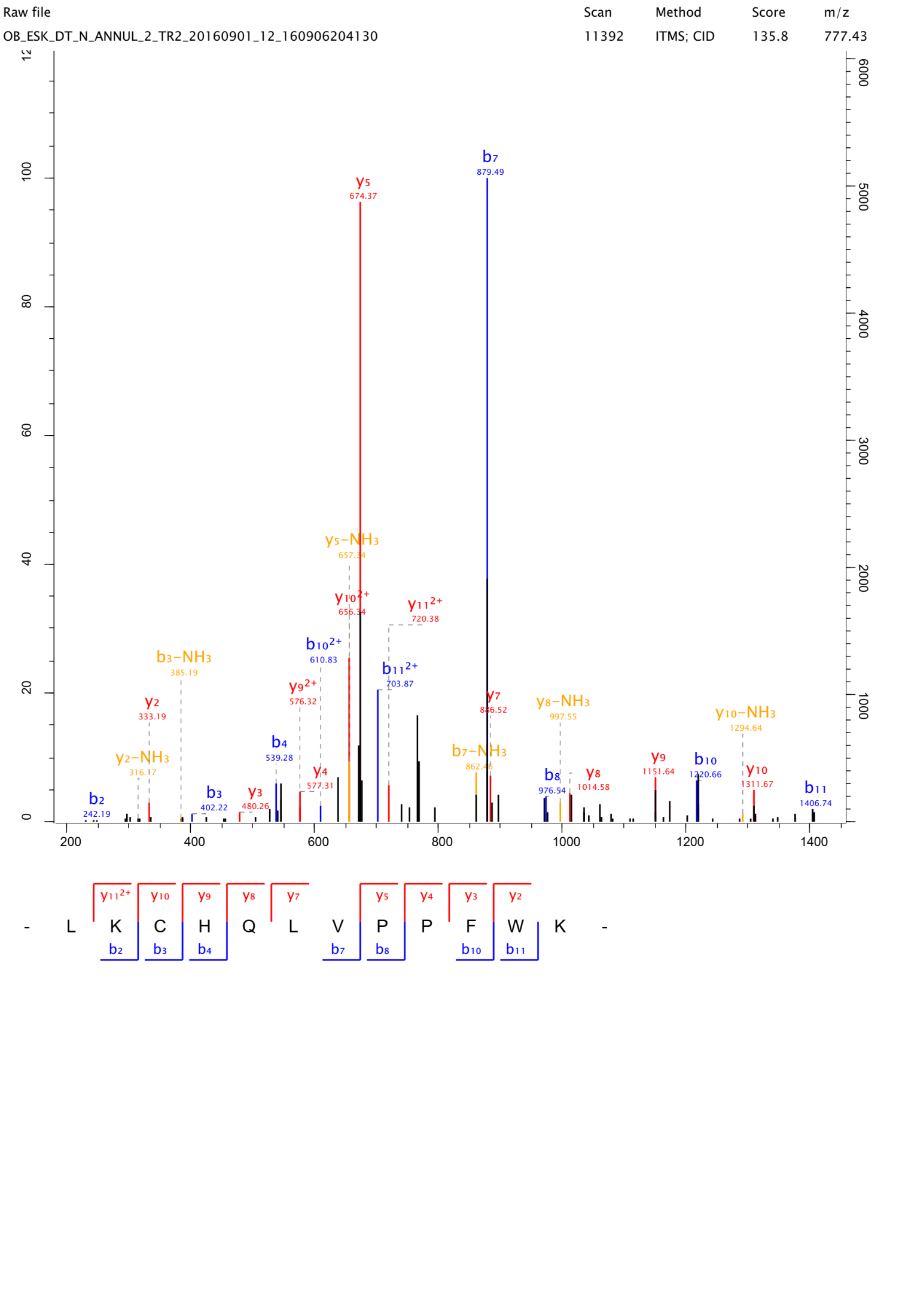


**Protein ID – P01461**

**Protein name:** Cytotoxin 4 OS=Naja annulifera OX=96794 PE=1 SV=1

**Number of Unique Peptides:** 1

**m/z:** 651.85

**MS/MS ID:** 479

**Score:** 140.83

**Spectrum:** 1/1


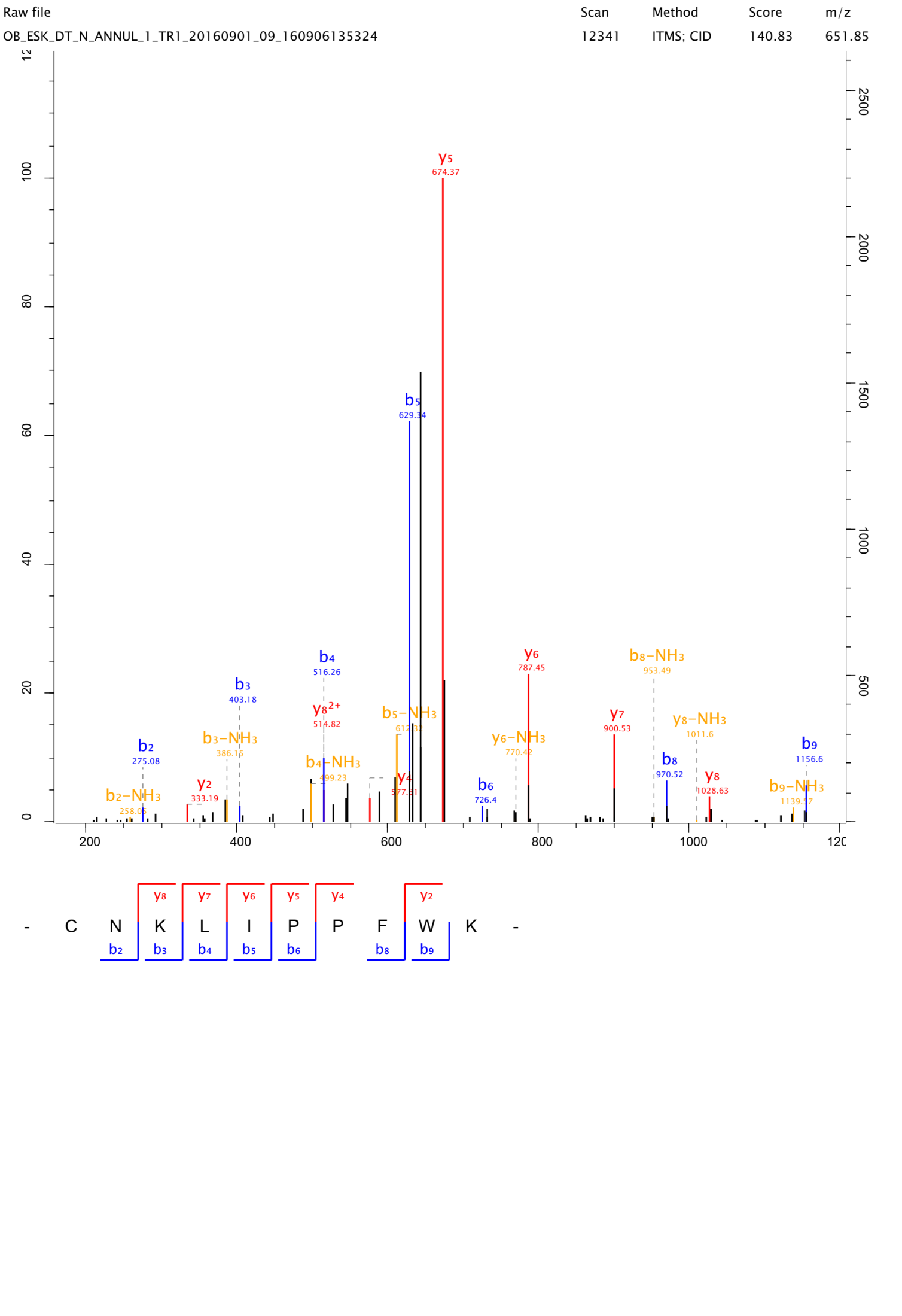


**Protein ID – P01462**

**Protein name:** Cytotoxin 2 OS=Naja annulifera OX=96794 PE=1 SV=1

**Number of Unique Peptides:** 2

**m/z:** 690.86

**MS/MS ID:** 2762

**Score:** 115.29

**Spectrum:** 1/2


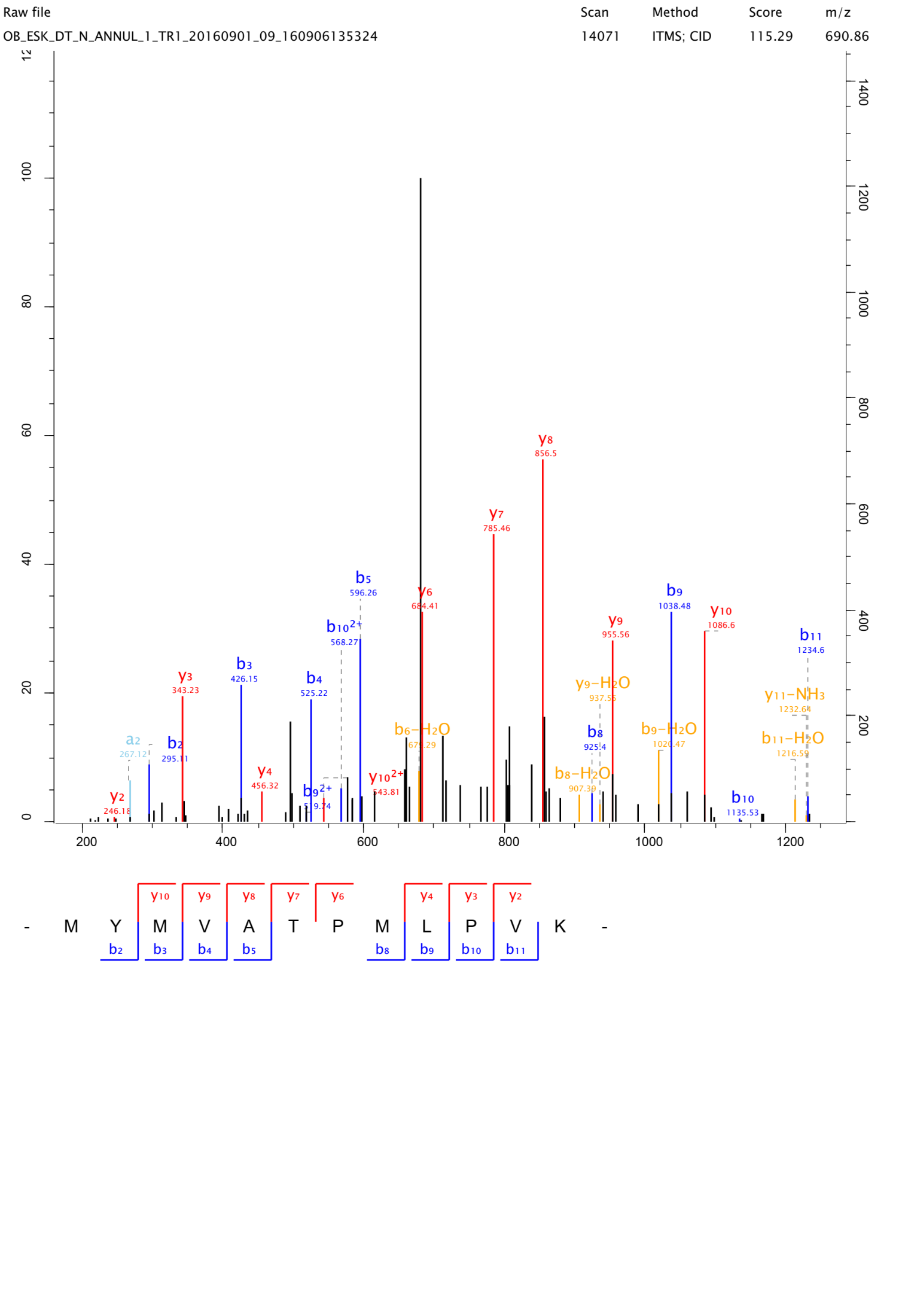


**Protein ID – P01462**

**Protein name:** Cytotoxin 2 OS=Naja annulifera OX=96794 PE=1 SV=1

**Number of Unique Peptides:** 2

**m/z:** 768.91

**MS/MS ID:** 2770

**Score:** 140.78

**Spectrum:** 2/2


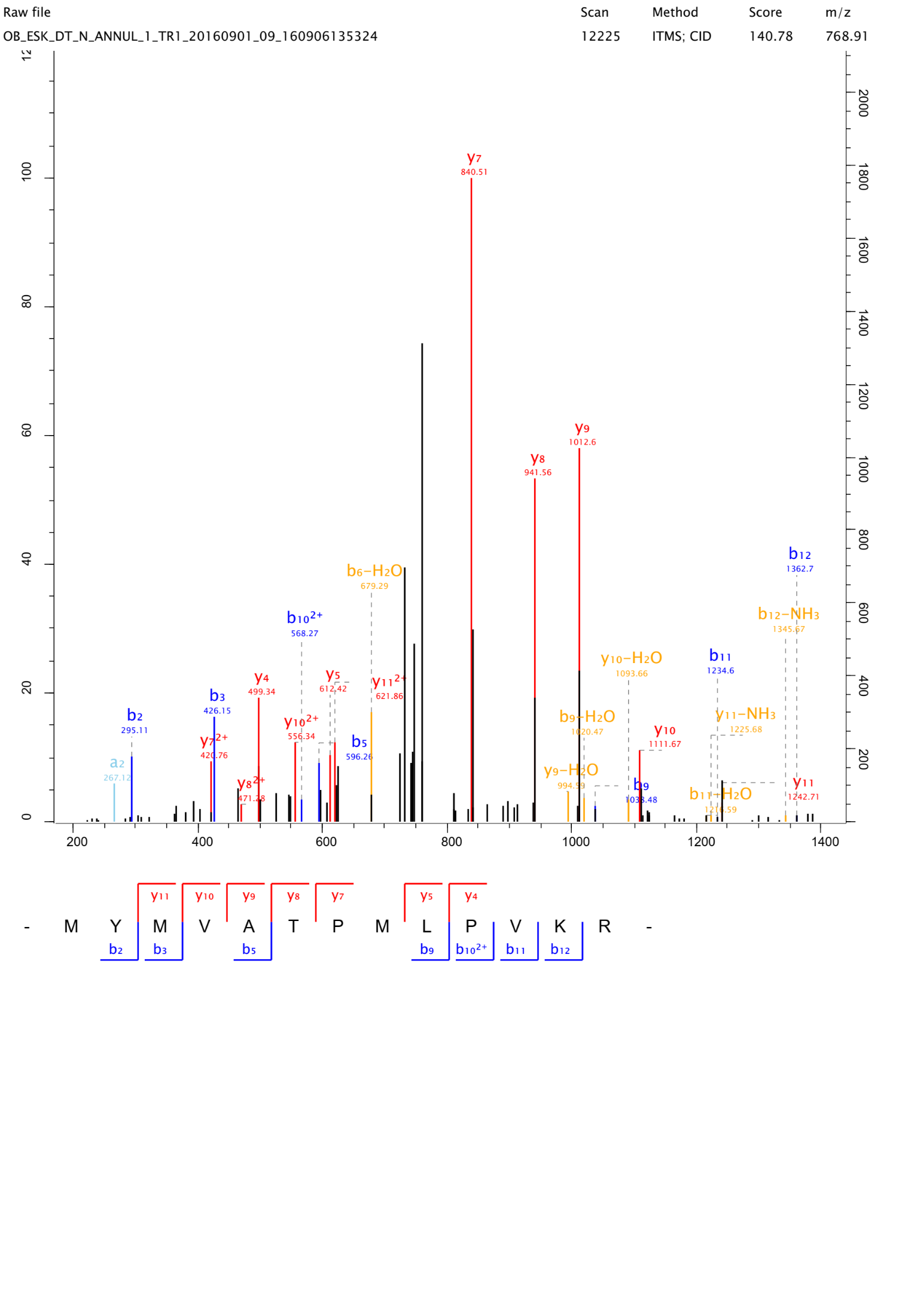


**Protein ID – P01463**

**Protein name:** Cytotoxin 2 OS=Naja nivea OX=8655 PE=1 SV=1

**Number of Unique Peptides:** 2

**m/z:** 663.34

**MS/MS ID:** 454

**Score:** 183.43

**Spectrum:** 1/2


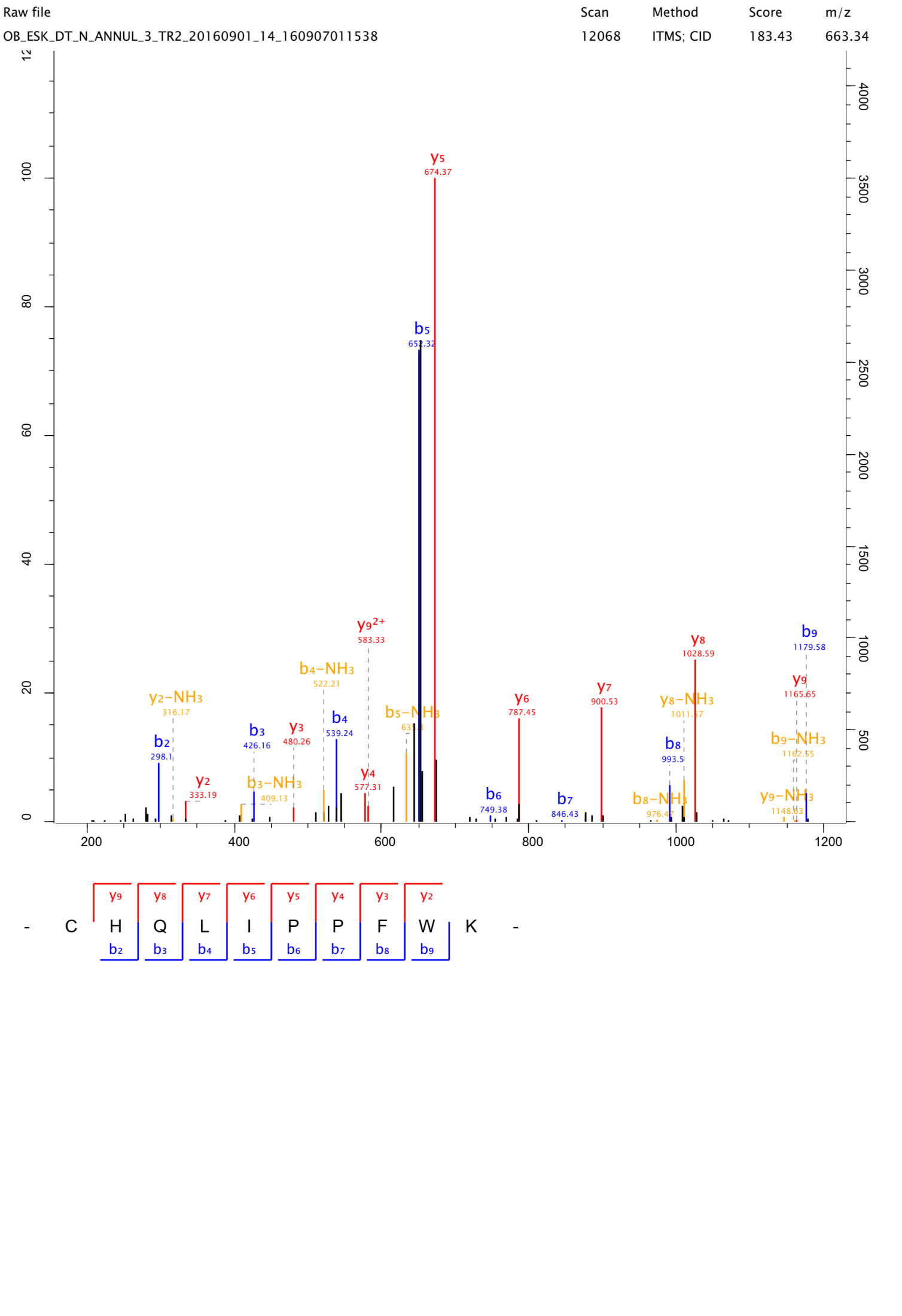


**Protein ID – P01463**

**Protein name:** Cytotoxin 2 OS=Naja nivea OX=8655 PE=1 SV=1

**Number of Unique Peptides:** 2

**m/z:** 783.93

**MS/MS ID:** 2327

**Score:** 160.91

**Spectrum:** 2/2


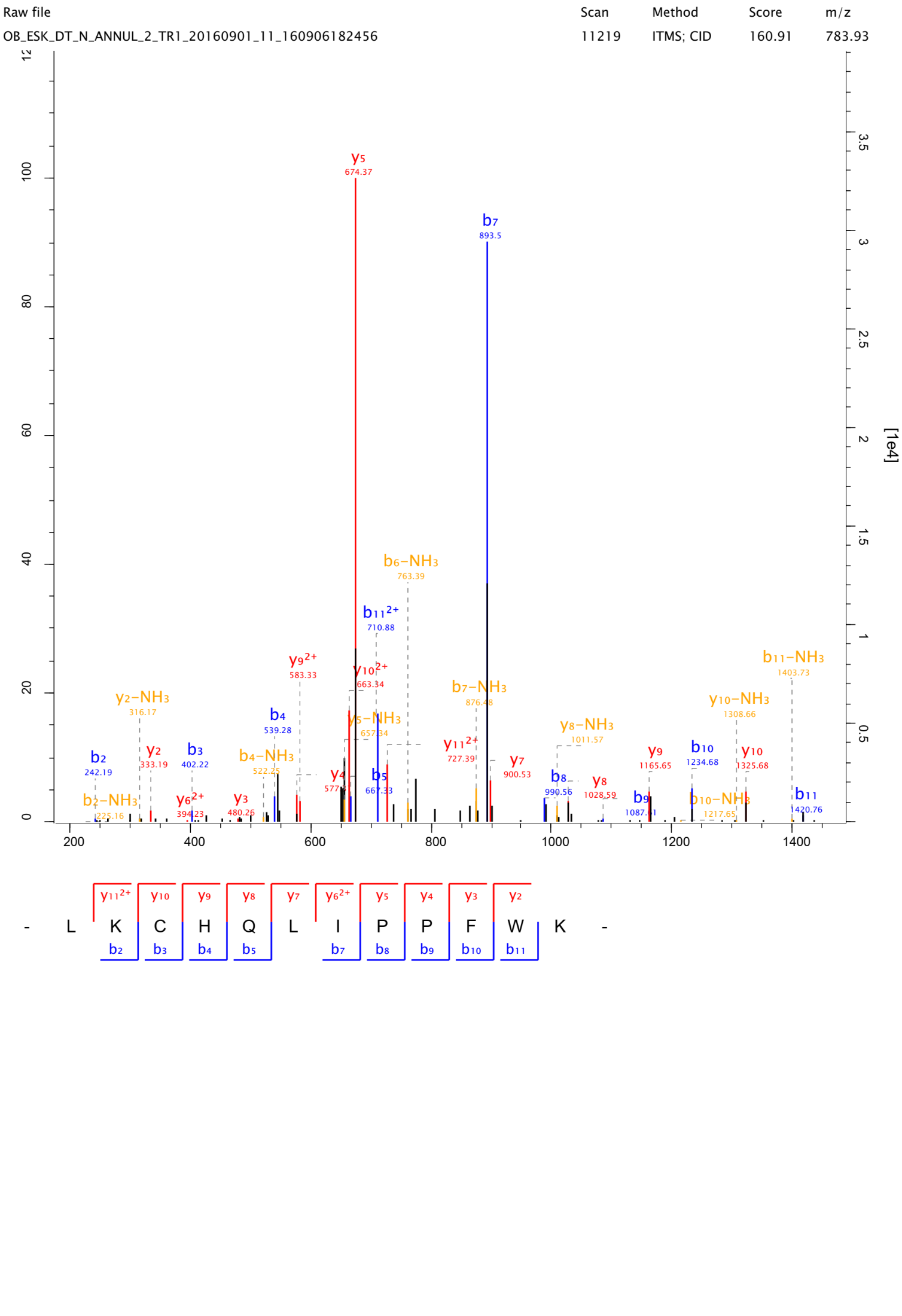


**Protein ID – P01464**

**Protein name:** Cytotoxin 5 OS=Naja annulifera OX=96794 PE=1 SV=1

**Number of Unique Peptides:** 1

**m/z:** 988.93

**MS/MS ID:** 2854

**Score:** 111.88

**Spectrum:** 1/1


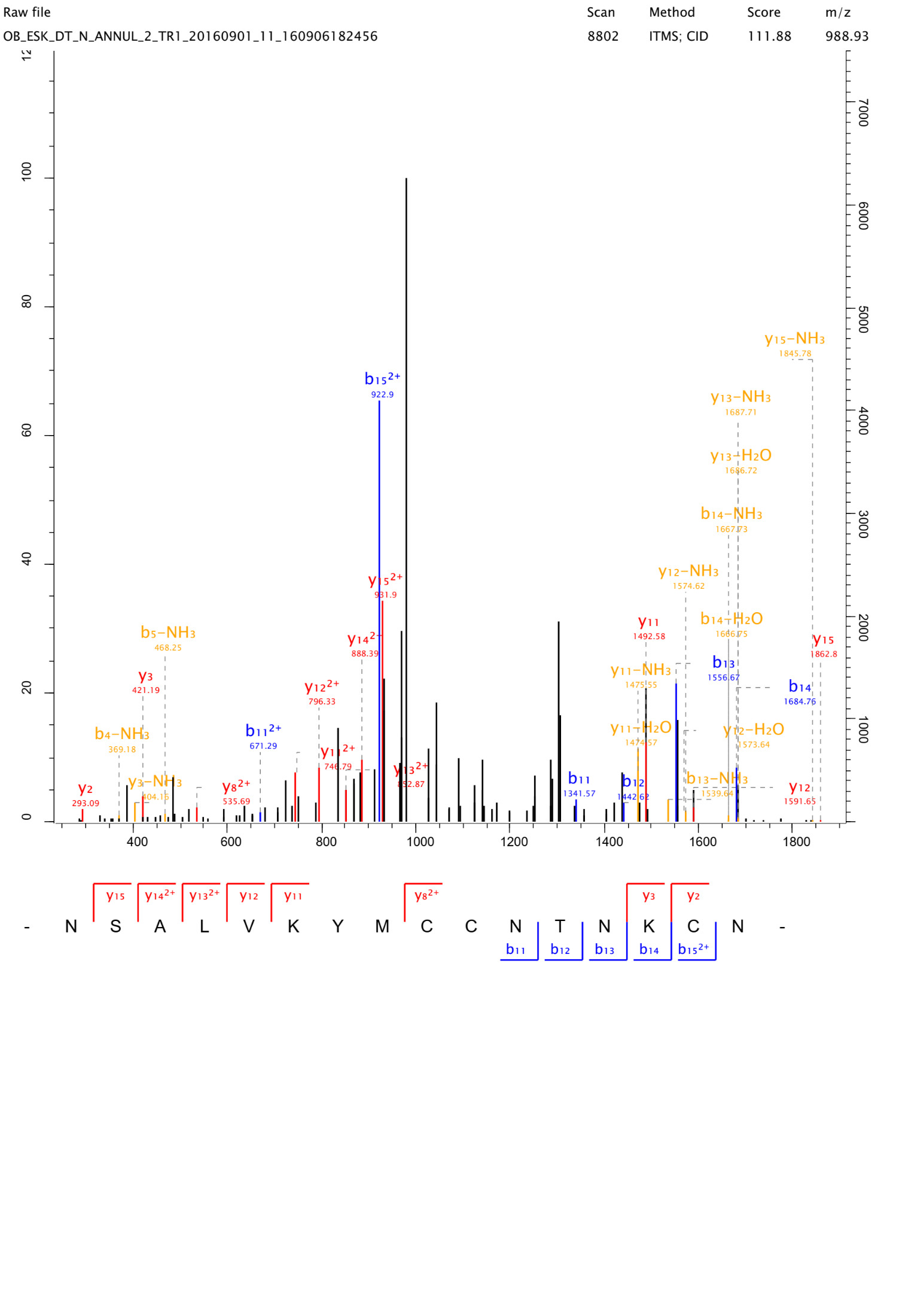


**Protein ID – P0DL15**

**Protein name:** Cysteine-rich venom protein annuliferin-b (Fragment) OS=Naja annulifera OX=96794 PE=1 SV=1

**Number of Unique Peptides:** 1

**m/z:** 565.32

**MS/MS ID:** 2828

**Score:** 157.33

**Spectrum:** 1/1


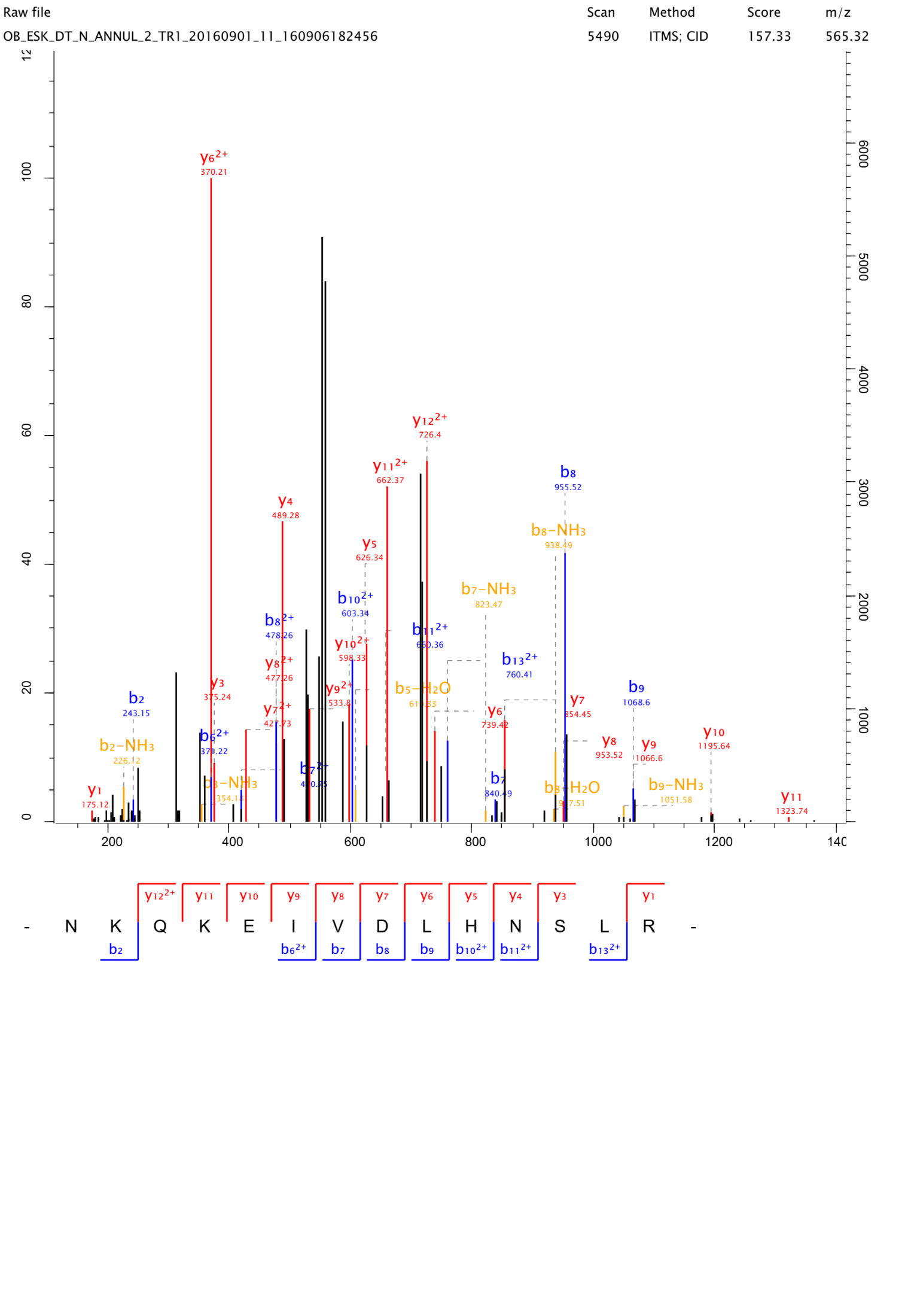


**Protein ID – P25674**

**Protein name:** Long neurotoxin 1 OS=Naja haje haje OX=8642 PE=1 SV=1

**Number of Unique Peptides:** 3

**m/z:** 819.69

**MS/MS ID:** 343

**Score:** 204.23


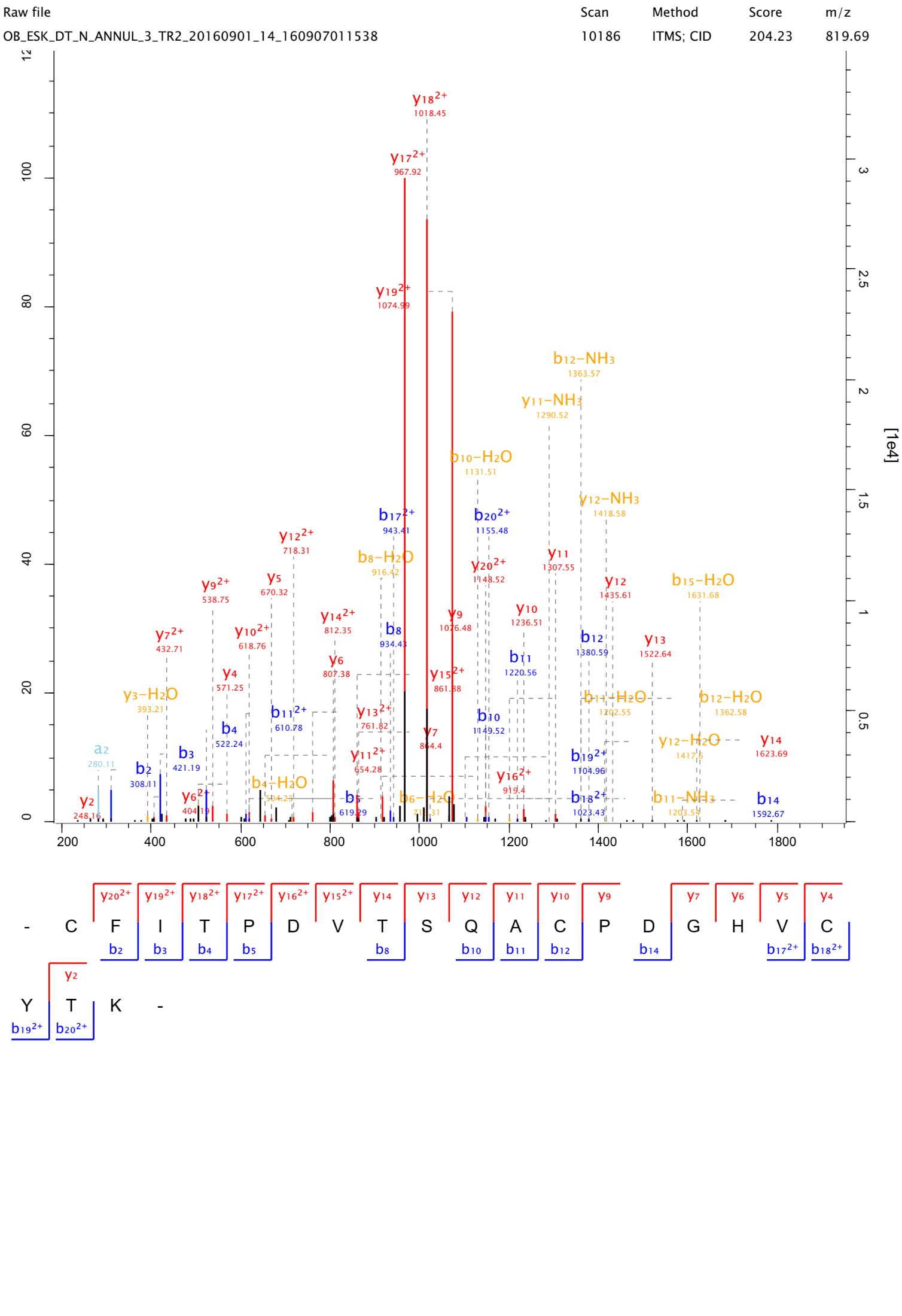
**Spectrum:** 1/1

**Protein ID – P25674**

**Protein name:** Long neurotoxin 1 OS=Naja haje haje OX=8642 PE=1 SV=1

**Number of Unique Peptides:** 3

**m/z:** 909.42

**MS/MS ID:** 1840

**Score:** 113.78

**Spectrum:** 2/3

**Protein ID – P25674**

**Protein name:** Long neurotoxin 1 OS=Naja haje haje OX=8642 PE=1 SV=1

**Number of Unique Peptides:** 3

**m/z:** 688.75

**MS/MS ID:** 2759

**Score:** 150.1

**Spectrum:** 3/3

**Protein ID – P25678**

**Protein name:** Weak toxin CM-2a OS=Naja annulifera OX=96794 PE=1 SV=1

**Number of Unique Peptides:** 2

**m/z:** 529.74

**MS/MS ID:** 2235

**Score:** 166.09

**Spectrum:** 1/2

**Protein ID – P25678**

**Protein name:** Weak toxin CM-2a OS=Naja annulifera OX=96794 PE=1 SV=1

**Number of Unique Peptides:** 2

**m/z:** 764.67

**MS/MS ID:** 2824

**Score:** 69.81

**Spectrum:** 2/2

**Protein ID – P29181**

**Protein name:** Weak neurotoxin 7 OS=Naja naja OX=35670 PE=1 SV=1

**Number of Unique Peptides:** 1

**m/z:** 737.36

**MS/MS ID:** 2980

**Score:** 144.88

**Spectrum:** 1/1

**Protein ID – P61899**

**Protein name:** Venom nerve growth factor OS=Naja kaouthia OX=8649 PE=1 SV=1

**Number of Unique Peptides:** 5

**m/z:** 711.38

**MS/MS ID:** 1779

**Score:** 216.91

**Spectrum:** 1/5

**Protein ID – P61899**

**Protein name:** Venom nerve growth factor OS=Naja kaouthia OX=8649 PE=1 SV=1

**Number of Unique Peptides:** 5

**m/z:** 613.34

**MS/MS ID:** 671

**Score:** 169.98

**Spectrum:** 2/5

**Protein ID – P61899**

**Protein name:** Venom nerve growth factor OS=Naja kaouthia OX=8649 PE=1 SV=1

**Number of Unique Peptides:** 5

**m/z:** 708.31

**MS/MS ID:** 475

**Score:** 145.81

**Spectrum:** 3/5

**Protein ID – P61899**

**Protein name:** Venom nerve growth factor OS=Naja kaouthia OX=8649 PE=1 SV=1

**Number of Unique Peptides:** 5

**m/z:** 647.33

**MS/MS ID:** 1777

**Score:** 188.27

**Spectrum:** 4/5

**Protein ID – P61899**

**Protein name:** Venom nerve growth factor OS=Naja kaouthia OX=8649 PE=1 SV=1

**Number of Unique Peptides:** 5

**m/z:** 570.97

**MS/MS ID:** 663

**Score:** 142.39

**Spectrum:** 5/5

**Protein ID – P62394**

**Protein name:** Cytotoxin 11 OS=Naja haje haje OX=8642 PE=1 SV=1

**Number of Unique Peptides:** 3

**m/z:** 621.75

**MS/MS ID:** 3605

**Score:** 192.12

**Spectrum:** 1/3

**Protein ID – P62394**

**Protein name:** Cytotoxin 11 OS=Naja haje haje OX=8642 PE=1 SV=1

**Number of Unique Peptides:** 3

**m/z:** 359.92

**MS/MS ID:** 2406

**Score:** 116.37

**Spectrum:** 2/3

**Protein ID – P62394**

**Protein name:** Cytotoxin 11 OS=Naja haje haje OX=8642 PE=1 SV=1

**Number of Unique Peptides:** 3

**m/z:** 525.38

**MS/MS ID:** 2104

**Score:** 193.41

**Spectrum:** 3/3

**Protein ID – P68419**

**Protein name:** Short neurotoxin 1 OS=Naja nivea OX=8655 PE=1 SV=1

**Number of Unique Peptides:** 3

**m/z:** 353.93

**MS/MS ID:** 3682

**Score:** 52.39

**Spectrum:** 1/3

**Protein ID – P68419**

**Protein name:** Short neurotoxin 1 OS=Naja nivea OX=8655 PE=1 SV=1

**Number of Unique Peptides:** 3

**m/z:** 357.52

**MS/MS ID:** 518

**Score:** 109.44

**Spectrum:** 2/3

**Protein ID – P68419**

**Protein name:** Short neurotoxin 1 OS=Naja nivea OX=8655 PE=1 SV=1

**Number of Unique Peptides:** 3

**m/z:** 913.89

**MS/MS ID:** 2076

**Score:** 257.8

**Spectrum:** 3/3

**Protein ID – P81782**

**Protein name:** Bucandin OS=Bungarus candidus OX=92438 PE=1 SV=1

**Number of Unique Peptides:** 3

**m/z:** 754.82

**MS/MS ID:** 1901

**Score:** 253.97

**Spectrum:** 1/3

**Protein ID – P81782**

**Protein name:** Bucandin OS=Bungarus candidus OX=92438 PE=1 SV=1

**Number of Unique Peptides:** 3

**m/z:** 509.23

**MS/MS ID:** 354

**Score:** 168.25

**Spectrum:** 2/3

**Protein ID – P81782**

**Protein name:** Bucandin OS=Bungarus candidus OX=92438 PE=1 SV=1

**Number of Unique Peptides:** 3

**m/z:** 572.22

**MS/MS ID:** 326

**Score:** 140.17

**Spectrum:** 3/3

**Protein ID – P82462**

**Protein name:** Muscarinic toxin-like protein 1 OS=Naja kaouthia OX=8649 PE=1 SV=1

**Number of Unique Peptides:** 1

**m/z:** 1092.5

**MS/MS ID:** 701

**Score:** 121.14

**Spectrum:** 1/1

**Protein ID – P82885**

**Protein name:** Thaicobrin OS=Naja kaouthia OX=8649 PE=1 SV=1

**Number of Unique Peptides:** 1

**m/z:** 905.95

**MS/MS ID:** 3504

**Score:** 202.81

**Spectrum:** 1/1

**Protein ID – P82942**

**Protein name:** Hemorrhagic metalloproteinase-disintegrin-like kaouthiagin OS=Naja kaouthia OX=8649 PE=1 SV=1

**Number of Unique Peptides:** 1

**m/z:** 851.75

**MS/MS ID:** 3416

**Score:** 71.64

**Spectrum:** 1/1

**Protein ID – P84807**

**Protein name:** Cysteine-rich venom protein 25-A (Fragment) OS=Naja haje haje OX=8642 PE=1 SV=1

**Number of Unique Peptides:** 1

**m/z:** 585.26

**MS/MS ID:** 529

**Score:** 158.08

**Spectrum:** 1/1

**Protein ID – Q10749**

**Protein name:** Snake venom metalloproteinase-disintegrin-like mocarhagin OS=Naja mossambica OX=8644 PE=1 SV=3

**Number of Unique Peptides:** 2

**m/z:** 399.89

**MS/MS ID:** 2110

**Score:** 120.03

**Spectrum:** 1/2

**Protein ID – Q10749**

**Protein name:** Snake venom metalloproteinase-disintegrin-like mocarhagin OS=Naja mossambica OX=8644 PE=1 SV=3

**Number of Unique Peptides:** 2

**m/z:** 535.29

**MS/MS ID:** 3395

**Score:** 158.08

**Spectrum:** 2/2

**Protein ID – Q5YF89**

**Protein name:** Venom nerve growth factor 2 OS=Naja sputatrix OX=33626 PE=2 SV=1

**Number of Unique Peptides:** 2

**m/z:** 676.85

**MS/MS ID:** 3675

**Score:** 178.46

**Spectrum:** 1/2

**Protein ID – Q5YF89**

**Protein name:** Venom nerve growth factor 2 OS=Naja sputatrix OX=33626 PE=2 SV=1

**Number of Unique Peptides:** 2

**m/z:** 481.73

**MS/MS ID:** 2957

**Score:** 153.04

**Spectrum:** 2/2

**Protein ID – Q7T1K6**

**Protein name:** Cysteine-rich venom protein natrin-1 OS=Naja atra OX=8656 PE=1 SV=1

**Number of Unique Peptides:** 2

**m/z:** 629.74

**MS/MS ID:** 3360

**Score:** 201.09

**Spectrum:** 1/2

**Protein ID – Q7T1K6**

**Protein name:** Cysteine-rich venom protein natrin-1 OS=Naja atra OX=8656 PE=1 SV=1

**Number of Unique Peptides:** 2

**m/z:** 633.78

**MS/MS ID:** 2938

**Score:** 170.89

**Spectrum:** 2/2

**Protein ID – Q7ZT98**

**Protein name:** Cysteine-rich venom protein ophanin OS=Ophiophagus hannah OX=8665 PE=1 SV=1

**Number of Unique Peptides:** 1

**m/z:** 916.95

**MS/MS ID:** 306

**Score:** 93.11

**Spectrum:** 1/1

**Protein ID – Q8JI38**

**Protein name:** Cysteine-rich venom protein latisemin OS=Laticauda semifasciata OX=8631 PE=2 SV=1

**Number of Unique Peptides:** 1

**m/z:** 649.81

**MS/MS ID:** 493

**Score:** 113.38

**Spectrum:** 1/1

**Protein ID – Q91132**

**Protein name:** Cobra venom factor OS=Naja kaouthia OX=8649 PE=1 SV=1

**Number of Unique Peptides:** 7

**m/z:** 748.4

**MS/MS ID:** 4016

**Score:** 106.54

**Spectrum:** 1/7

**Protein ID – Q91132**

**Protein name:** Cobra venom factor OS=Naja kaouthia OX=8649 PE=1 SV=1

**Number of Unique Peptides:** 7

**m/z:** 681.3

**MS/MS ID:** 10

**Score:** 136.53

**Spectrum:** 2/7

**Protein ID – Q91132**

**Protein name:** Cobra venom factor OS=Naja kaouthia OX=8649 PE=1 SV=1

**Number of Unique Peptides:** 7

**m/z:** 527.81

**MS/MS ID:** 1771

**Score:** 125.74

**Spectrum:** 3/7

**Protein ID – Q91132**

**Protein name:** Cobra venom factor OS=Naja kaouthia OX=8649 PE=1 SV=1

**Number of Unique Peptides:** 7

**m/z:** 1052.6

**MS/MS ID:** 290

**Score:** 141.6

**Spectrum:** 4/7

**Protein ID – Q91132**

**Protein name:** Cobra venom factor OS=Naja kaouthia OX=8649 PE=1 SV=1

**Number of Unique Peptides:** 7

**m/z:** 1056.07

**MS/MS ID:** 1798

**Score:** 173.19

**Spectrum:** 5/7

**Protein ID – Q91132**

**Protein name:** Cobra venom factor OS=Naja kaouthia OX=8649 PE=1 SV=1

**Number of Unique Peptides:** 7

**m/z:** 588.82

**MS/MS ID:** 1816

**Score:** 180.66

**Spectrum:** 6/7

**Protein ID – Q91132**

**Protein name:** Cobra venom factor OS=Naja kaouthia OX=8649 PE=1 SV=1

**Number of Unique Peptides:** 7

**m/z:** 669.33

**MS/MS ID:** 3563

**Score:** 178.15

**Spectrum:** 7/7

**Protein ID – Q92035**

**Protein name:** Acetylcholinesterase OS=Bungarus fasciatus OX=8613 GN=ACHE PE=1 SV=2

**Number of Unique Peptides:** 1

**m/z:** 683.98

**MS/MS ID:** 2917

**Score:** 122.63

**Spectrum:** 1/1

**Protein ID – Q92085**

**Protein name:** Neutral phospholipase A2 B OS=Naja sputatrix OX=33626 PE=2 SV=1

**Number of Unique Peptides:** 1

**m/z:** 622.58

**MS/MS ID:** 3404

**Score:** 218.62

**Spectrum:** 1/1

**Protein ID – Q9W6W6**

**Protein name:** Cytotoxin 10 OS=Naja atra OX=8656 PE=3 SV=1

**Number of Unique Peptides:** 1

**m/z:** 789.39

**MS/MS ID:** 1636

**Score:** 129.76

**Spectrum:** 1/1

**Protein ID – R4FID0**

**Protein name:** Amine oxidase OS=Denisonia devisi OX=529689 PE=2 SV=1

**Number of Unique Peptides:** 3

**m/z:** 665.88

**MS/MS ID:** 3017

**Score:** 139.32

**Spectrum:** 1/3

**Protein ID – R4FID0**

**Protein name:** Amine oxidase OS=Denisonia devisi OX=529689 PE=2 SV=1

**Number of Unique Peptides:** 3

**m/z:** 651.88

**MS/MS ID:** 2034

**Score:** 114.97

**Spectrum:** 2/3

**Protein ID – R4FID0**

**Protein name:** Amine oxidase OS=Denisonia devisi OX=529689 PE=2 SV=1

**Number of Unique Peptides:** 3

**m/z:** 587.83

**MS/MS ID:** 2028

**Score:** 116.52

**Spectrum:** 3/3

**Protein ID – R4G7H8**

**Protein name:** 3FTx-Fur-10 OS=Furina ornata OX=529697 PE=2 SV=1

**Number of Unique Peptides:** 1

**m/z:** 670.78

**MS/MS ID:** 3509

**Score:** 106.88

**Spectrum:** 1/1

**Protein ID – R4FK68**

**Protein name:** 3FTx-Pse-105 OS=Pseudonaja modesta OX=340912 PE=2 SV=1

**Number of Unique Peptides:** 1

**m/z:** 1205.19

**MS/MS ID:** 3527

**Score:** 41.92

**Spectrum:** 1/1

**Protein ID – R4FKE6**

**Protein name:** Carboxylic ester hydrolase (Fragment) OS=Suta fasciata OX=529716 PE=2 SV=1

**Number of Unique Peptides:** 2

**m/z:** 878.15

**MS/MS ID:** 1747

**Score:** 87.32

**Spectrum:** 1/2

**Protein ID – R4FKE6**

**Protein name:** Carboxylic ester hydrolase (Fragment) OS=Suta fasciata OX=529716 PE=2 SV=1

**Number of Unique Peptides:** 2

**m/z:** 513.93

**MS/MS ID:** 2851

**Score:** 143

**Spectrum:** 2/2

**Protein ID – V8N4Y2**

**Protein name:** Endonuclease domain-containing 1 protein OS=Ophiophagus hannah OX=8665 GN=ENDOD1 PE=4 SV=1

**Number of Unique Peptides:** 3

**m/z:** 848.47

**MS/MS ID:** 3375

**Score:** 230.69

**Spectrum:** 1/3

**Protein ID – V8N4Y2**

**Protein name:** Endonuclease domain-containing 1 protein OS=Ophiophagus hannah OX=8665 GN=ENDOD1 PE=4 SV=1

**Number of Unique Peptides:** 3

**m/z:** 1056.03

**MS/MS ID:** 1657

**Score:** 169.7

**Spectrum:** 2/3

**Protein ID – V8N4Y2**

**Protein name:** Endonuclease domain-containing 1 protein OS=Ophiophagus hannah OX=8665 GN=ENDOD1 PE=4 SV=1

**Number of Unique Peptides:** 3

**m/z:** 800.4

**MS/MS ID:** 2128

**Score:** 145.91

**Spectrum:** 3/3

**Protein ID – V8N6R7**

**Protein name:** Protein phosphatase 1 regulatory subunit 3A (Fragment) OS=Ophiophagus hannah OX=8665 GN=Ppp1r3a PE=4 SV=1

**Number of Unique Peptides:** 1

**m/z:** 931.68

**MS/MS ID:** 2615

**Score:** 34.04

**Spectrum:** 1/1

**Protein ID – V8NEV9**

**Protein name:** Tudor domain-containing protein 6 (Fragment) OS=Ophiophagus hannah OX=8665 GN=TDRD6 PE=4 SV=1

**Number of Unique Peptides:** 1

**m/z:** 558.81

**MS/MS ID:** 2844

**Score:** 143.7

**Spectrum:** 1/1

**Protein ID – V8NUN2**

**Protein name:** Transmembrane protein 14C OS=Ophiophagus hannah OX=8665 GN=tmem14c PE=4 SV=1

**Number of Unique Peptides:** 1

**m/z:** 633.82

**MS/MS ID:** 2510

**Score:** 108.31

**Spectrum:** 1/1

**Protein ID – V8NWT6**

**Protein name:** Ankyrin repeat domain-containing protein 34B (Fragment) OS=Ophiophagus hannah OX=8665 GN=ANKRD34B PE=4 SV=1

**Number of Unique Peptides:** 1

**m/z:** 327.86

**MS/MS ID:** 3027

**Score:** 104.2

**Spectrum:** 1/1

**Protein ID – V8P0T5**

**Protein name:** Tumor necrosis factor receptor superfamily member 11B OS=Ophiophagus hannah OX=8665 GN=TNFRSF11B PE=4 SV=1

**Number of Unique Peptides:** 2

**m/z:** 614.31

**MS/MS ID:** 3941

**Score:** 155.06

**Spectrum:** 1/2

**Protein ID – V8P0T5**

**Protein name:** Tumor necrosis factor receptor superfamily member 11B OS=Ophiophagus hannah OX=8665 GN=TNFRSF11B PE=4 SV=1

**Number of Unique Peptides:** 2

**m/z:** 595.92

**MS/MS ID:** 504

**Score:** 98.59

**Spectrum:** 2/2

**Protein ID – V8P2C8**

**Protein name:** Pyruvate kinase OS=Ophiophagus hannah OX=8665 GN=PKM2 PE=3 SV=1

**Number of Unique Peptides:** 1

**m/z:** 620.97

**MS/MS ID:** 648

**Score:** 111.81

**Spectrum:** 1/1

**Protein ID – V8P2C8**

**Protein name:** Glutathione peroxidase (Fragment) OS=Ophiophagus hannah OX=8665 GN=Gpx3 PE=3 SV=1

**Number of Unique Peptides:** 10

**m/z:** 680.38

**MS/MS ID:** 648

**Score:** 175.88

**Spectrum:** 1/10

**Protein ID – V8P2C8**

**Protein name:** Glutathione peroxidase (Fragment) OS=Ophiophagus hannah OX=8665 GN=Gpx3 PE=3 SV=1

**Number of Unique Peptides:** 10

**m/z:** 592.78

**MS/MS ID:** 223

**Score:** 155.33

**Spectrum:** 2/10

**Protein ID – V8P2C8**

**Protein name:** Glutathione peroxidase (Fragment) OS=Ophiophagus hannah OX=8665 GN=Gpx3 PE=3 SV=1

**Number of Unique Peptides:** 10

**m/z:** 928.47

**MS/MS ID:** 1670

**Score:** 174.39

**Spectrum:** 3/10

**Protein ID – V8P2C8**

**Protein name:** Glutathione peroxidase (Fragment) OS=Ophiophagus hannah OX=8665 GN=Gpx3 PE=3 SV=1

**Number of Unique Peptides:** 10

**m/z:** 445.76

**MS/MS ID:** 2237

**Score:** 148.21

**Spectrum:** 4/10

**Protein ID – V8P2C8**

**Protein name:** Glutathione peroxidase (Fragment) OS=Ophiophagus hannah OX=8665 GN=Gpx3 PE=3 SV=1

**Number of Unique Peptides:** 10

**m/z:** 665.35

**MS/MS ID:** 1781

**Score:** 208.84

**Spectrum:** 5/10

**Protein ID – V8P2C8**

**Protein name:** Glutathione peroxidase (Fragment) OS=Ophiophagus hannah OX=8665 GN=Gpx3 PE=3 SV=1

**Number of Unique Peptides:** 10

**m/z:** 734.74

**MS/MS ID:** 2241

**Score:** 87.82

**Spectrum:** 6/10

**Protein ID – V8P2C8**

**Protein name:** Glutathione peroxidase (Fragment) OS=Ophiophagus hannah OX=8665 GN=Gpx3 PE=3 SV=1

**Number of Unique Peptides:** 10

**m/z:** 1005.86

**MS/MS ID:** 2525

**Score:** 88.63

**Spectrum:** 7/10

**Protein ID – V8P2C8**

**Protein name:** Glutathione peroxidase (Fragment) OS=Ophiophagus hannah OX=8665 GN=Gpx3 PE=3 SV=1

**Number of Unique Peptides:** 10

**m/z:** 746.9

**MS/MS ID:** 2517

**Score:** 113.38

**Spectrum:** 8/10

**Protein ID – V8P2C8**

**Protein name:** Glutathione peroxidase (Fragment) OS=Ophiophagus hannah OX=8665 GN=Gpx3 PE=3 SV=1

**Number of Unique Peptides:** 10

**m/z:** 770.9

**MS/MS ID:** 2891

**Score:** 172.85

**Spectrum:** 9/10

**Protein ID – V8P2C8**

**Protein name:** Glutathione peroxidase (Fragment) OS=Ophiophagus hannah OX=8665 GN=Gpx3 PE=3 SV=1

**Number of Unique Peptides:** 10

**m/z:** 622.66

**MS/MS ID:** 3456

**Score:** 113.76

**Spectrum:** 10/10
