# Supplementary figures and images for: Naja annulifera Snake: New insights into the venom components and pathogenesis of envenomation
Source: PLoS Negl Trop Dis. 2019 Jan 18;13(1):e0007017. doi: 10.1371/journal.pntd.0007017 (PMC6338361; doi:10.1371/journal.pntd.0007017)

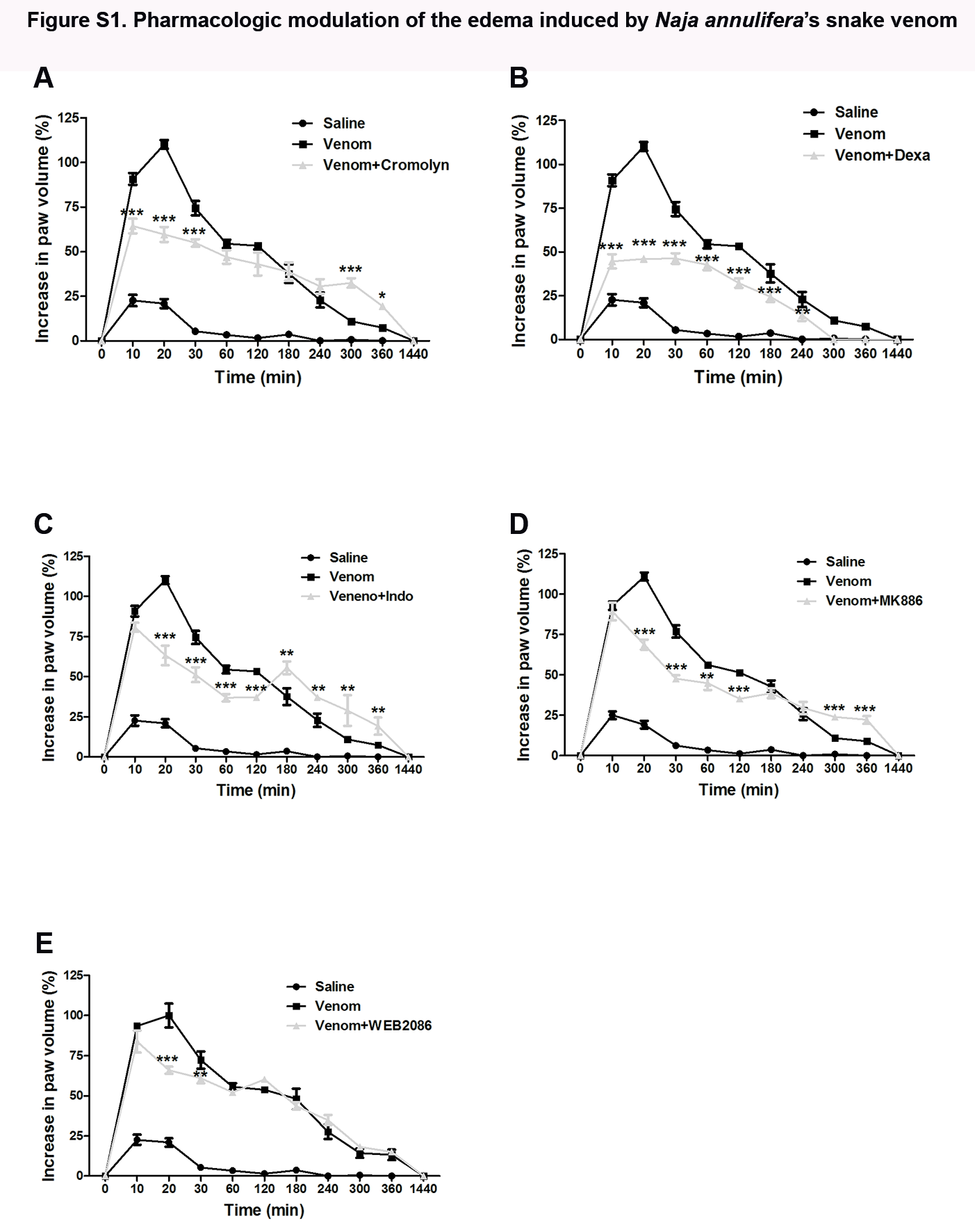

Supplement: S1 Fig — To evaluate the contribution of the different inflammatory mediators classes in hindpaw edema induced by N. annulifera’ s venom, Balb/c mice groups were treated with [A] Cromolyn, a mast cell degranulation inhibitor (10 mg/kg, three days consecutively before the edema, i.p. route); [B] dexamethasone (Dexa), a cPLA2 inhibitor (2 mg/kg, 2 hours before the edema, i.p. route); [C] indomethacin (Indo), a COX isoform inhibitor (10 mg/kg, 30 minutes before the edema, i.p. route); [D] MK886, a 5-lipoxygenase-activating protein inhibitor (5 mg/kg, 30 minutes before the edema, i.p. route) and [E] WEB2086, a PAFR antagonist (5 mg/kg, 1 hour before the edema, s.c. route). After treatment, edema was induced, and the paws were measured with a caliper rule at several time points. The increased paw volume was expressed as percentage. Statistical analysis was performed using two-way ANOVA, followed by the Bonferroni multiple comparison test (***p≤ 0.05) ± SD. (TIF) [file pntd.0007017.s004.tif]
